# Abstracts from the 25th European Society for Animal Cell Technology Meeting: Cell Technologies for Innovative Therapies: Lausanne, Switzerland. 14-17 May 2017

**DOI:** 10.1186/s12919-018-0097-x

**Published:** 2018-03-15

## O-001 CRISPR-Cas based synthetic transcription factors: A strategy for improving bioproduction in CHO cells

### Si Nga Sou, Dirk-Jan Kleinjan, Susan J. Rosser

#### Institute of Quantitative Biology, Biochemistry, and Biotechnology, School of Biological Sciences, University of Edinburgh, Edinburgh, EH9 3FF, UK

##### **Correspondence:** Si Nga Sou (si.sou@ed.ac.uk)

**Background**

Despite advances in Chinese hamster ovary (CHO) cell bioprocess optimisation, production of large complex proteins remains costly and high degree of variability among final products is problematic. Novel strategies that target molecular pathways for high product yield and consistency are vital. To overcome this bottleneck, we developed a CRISPR-dCas based synthetic transcription factors (sTF) system that modulates expression of endogenous mRNA and miRNA targets involved in protein transport and glycosylation.

**Materials and methods**

sTF utilises two forms of Cas9 proteins: Endonuclease inactive ‘dead’ Cas9 (dCas9) with trans-activator domain (VPR) attached and native cutting Cas9 (Fig. 1a). In Herceptin® expressing CHO-K1, we transiently expressed dCas9-VPR with sgRNAs against upstream of protein transport-related gene promoters (Napg, Rab5A & Aprc1b) for transcriptional activation, or transfecting dCas9 with sgRNAs against their promoter regions for suppression (Vamp4). To lower galactosyltransferase (β1,4-GalT)-associated miRNA expression (cgr-miR-181d-5p, cgr-miR500 & cgr-miR501-5p), CHO cells were co-expressed with dCas9 and sgRNAs against miRNA promoters; or with native Cas9 and sgRNAs against mature miRNA sequences [1]. mRNA and miRNA levels of target genes were quantified by q-rt-PCR, protein level of β1,4-GalTs by western blot, and secreted IgG yield by IgG-ELISA.

**Results**

The dCas9 approach receives up to 60% increase in IgG expression, along with 1.2 to 2.5-fold rise in Napg, Rab5A and Aprc1b mRNA levels. While repressing Vamp4 transcription leads to a negative effect on IgG yield (Fig. 1b - c). Our results show positive correlation between pathways involved in protein transport and recycling, and recombinant protein (rProtein) yield. Both Cas9 and dCas9 approaches reduce miR-181d-5p, miR500 & miR501-5p by around 35-50%, this simultaneously enhances β1,4-GalT1 & 4 expression by up to 2-fold, which could be useful in future engineering of rProtein glycosylation profiles for specific function. This system also provides a platform for concurrent manipulation of multiple mRNA and miRNA with dCas9, where dCas9 expression can be further controlled via AID- or ecDFR-Degron technology [2].

**Conclusions**

Our works here present the potential of the CRISPRa/i system to easily reengineer or to study CHO cell metabolic pathways for more efficient rProtein production. The chemical inducible Cas9/dCas9 protein expression offers further control over multiple endogenous gene manipulation.

**Acknowledgements**

Authors thankfully acknowledge the Biotechnology and Biological Sciences Research Council for funding this research work. SNS thanks ESACT 2017 for providing her with the opportunity to present her work at the meeting.

**References**

1. Chang H, Yi B, Ma R, Zhang X, Zhao H, Xi Y. **CRISPR/cas9, a novel genomic tool to knock down microRNA in vitro and in vivo.** *Scientific Reports* 2016. 6:22312.

2. Kleinjan D, Wardrope C, Sou S, Rosser S. **A Toolkit of Tunable, Degron-tagged dCas9/Cpf1 Effectors for Multi-directional Drug-inducible control of Synthetic Gene Regulation.** *Nat Commun* 2017 (In press).

**Fig. 1 (abstract O-001).**
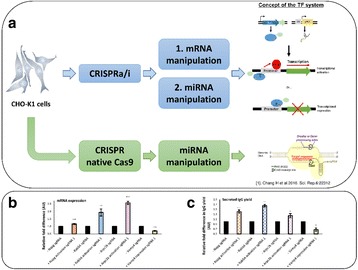
**a** Schematic representation of CRISPR based synthetic transcription factor technology. **b** mRNA expression levels of protein transport related genes (Napg, Rab5A and Arpc1b). **c** Quantification of secreted IgG production when CHO cells were transfected with dCas9-VPR/dCas9 and different sgRNAs

## O-002 Degradation of recombinant proteins of diverse formats by CHO host cell proteases is circumvented via knock-out of CHO matriptase

### Holger Laux^1^, Sandrine Romand^1^, Joel Tapparel^1^, Sandro Nuciforo^1^, Stine Buechmann-Moller^2^, Guelay Dogrusoez^1^, Sandra Haas^1^, Benjamin Sommer^1^, Edward J. Oakeley^2^, Ursula Bodendorf^2^

#### ^1^Novartis (BTDM), Basel, 4056, Switzerland; ^2^Novartis (NIBR), Basel, 4056, Switzerland

##### **Correspondence:** Holger Laux (holger.laux@novartis.com)

**Background**

An increasing number of biologics are entering the development pipelines of pharmaceutical companies [1]. Today, the preferred production host for therapeutic proteins is the CHO cell line. However one of the major hurdles, especially for the production of non-antibody glycoproteins, is host cell-related proteolytic degradation which can drastically impact developability and timelines of pipeline projects.

**Material and methods**

**Spike-in:** CHO cells were cultivated in a chemically defined culture medium at 36.5°C/10% CO_2_ in shake-flasks. When the cells reached their maximum viable density, they were removed by centrifugation and the conditioned medium was collected. A model mAb was spiked into the conditioned medium and incubated at 37°C ± protease inhibitors. The amount of proteolytic degradation was analysed by western blot and LC-MS.

**Transcriptomics:** Total RNA was extracted after 3 days of cell cultivation. RNA sequencing libraries were constructed and processed on the HiSeq 2000 platform from Illumina.

**Generation of matriptase knockout:** CHO-K1 cells were transfected with mRNA encoding “transcription activator-like effector nucleases” or “zinc finger nucleases” targeting matriptase exon 2. The transfected cells were subsequently sorted into single cells and analysed for frameshift mutations in both alleles via Sanger sequencing.

**Cell cultivation:** Fed batch cultivation was performed in 15-mL miniaturized bioreactors (AMBR15).

**Results**

Approximately 700 proteases are known in rodents. To reduce the number of candidate proteases we showed first that a model mAb (prone to proteolytic degradation) incubated in conditioned medium of CHO-K1 cells resulted in clipping of the mAb, demonstrating the involvement of secreted/shedded proteases (Fig. 1a). Broad spectrum inhibitors of the different protease classes revealed that only serine protease inhibitors prevented clipping. Serine protease inhibitors of higher specificity highlighted the group of “S1A trypsin-like proteases” (Fig. 1a).

Comparison of the proteolytic degradation profile of several therapeutic proteins between CHO-K1 with another CHO cell line (CHO-A) revealed less degradation in CHO-A. Therefore expression of the involved protease(s) is likely lower in CHO-A. Gene expression profile analysis of both cell lines showed five secreted/shedded “S1A trypsin–like serine proteases” more than 1.5 fold lower expressed in CHO- CHO-A versus CHO-K1 (Fig. 1b). Surprisingly, siRNA knockdown experiments of these five candidates identified “Matriptase” as the major protease involved in degradation of recombinant proteins expressed in CHO-K1 cells (Fig. 1c upper panel).

Next, we generated a CHO-K1 matriptase knockout (KO) cell line. No proteolytic degradation product was detected when the model mAb was spiked into conditioned medium of the KO cell line (Fig. 1c lower panel). Also, stable expression of the model mAb in the KO cell line resulted in no/significantly less clipping (Fig. 1e). The protein titer and the cell growth behaviour of the matriptase KO cells were similar to the corresponding wildtype (wt) cells (Fig. 1d) as shown by comparative cultivation in AMBR system.

**Conclusions**

One major challenge for the production of recombinant proteins is CHO host cell mediated proteolytic degradation which can negatively impact or even result in termination of projects [2; 3].

Using a variety of techniques such as applying protease inhibitors, transcriptomics and siRNA mediated knock-down we were able to identify “matriptase” as the major protease involved in degradation of recombinant proteins expressed in CHO-K1 cells. Subsequently we generated a matriptase deficient CHO cell line. Protein candidates of diverse formats, severely degraded in wt CHO-K1 cell line, were not or significantly less cleaved in the matriptase KO cell line. Furthermore cell growth, viability and productivity levels were comparable between the wt and the matriptase KO cell line. In summary, we have generated a superior platform-compatible CHO production host cell line with the same favourable productivity properties as the parental host cell line [4; 6], allowing expression of complex glycoproteins prone to clipping.

**Acknowledgements**

We would like to thank Moritz Frei for his support for the generation of the NGS transcriptomics data.

**References**

1. Walsh (2014) **Biopharmaceutical benchmarks 2014.** *nature biotechnology*. 32: 11.

2. Dorai, H., J. F. Nemeth, E. Cammaart, Y. Wang, Q. M. Tang, A. Magill, M. J. Lewis, T. S. Raju, K. Picha, K. O'Neil, S. Ganguly, and G. Moore (2009) **Development of mammalian production cell lines expressing CNTO736, a glucagon like peptide-1-MIMETIBODY: factors that influence productivity and product quality.** *Biotechnol Bioeng*. 103: 162-176.

3. Robert, F., H. Bierau, M. Rossi, D. Agugiaro, T. Soranzo, H. Broly, and C. Mitchell-Logean (2009) **Degradation of an Fc-fusion recombinant protein by host cell proteases: Identification of a CHO cathepsin D protease.** *Biotechnol Bioeng*. 104: 1132-1141.

4. Ritter, A., B. Voedisch, J. Wienberg, B. Wilms, S. Geisse, T. Jostock, and H. Laux (2016) **Deletion of a telomeric region on chromosome 8 correlates with higher productivity and stability of CHO cell lines.** *Biotechnol Bioeng*. 113: 1084-1093.

5. Ritter, A., T. Rauschert, M. Oertli, D. Piehlmaier, P. Mantas, G. Kuntzelmann, N. Lageyre, B. Brannetti, B. Voedisch, S. Geisse, T. Jostock, and H. Laux (2016) **Disruption of the gene C12orf35 leads to increased productivities in recombinant CHO cell lines.** *Biotechnol Bioeng*. 113: 2433-2442.

6. Ritter, A., S. Nuciforo, A. Schulze, M. Oertli, T. Rauschert, B. Voedisch, S. Geisse, T. Jostock, and H. Laux (2016) **Fam60A plays a role for production stabilities of recombinant CHO cell lines.** *Biotechnol Bioeng*.

**Fig. 1 (abstract O-002).**
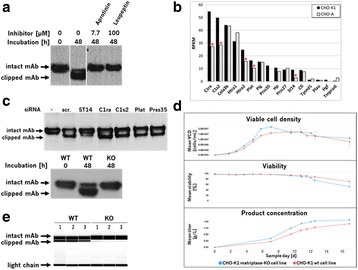
Matriptase knock-out in CHO cells prevents clipping of recombinant proteins. **a** Serine protease inhibitors protect model mAb from proteolytic degradation in CHO-K1 cell derived conditioned medium. The model mAb was incubated in conditioned medium for 0h or 48h at 37°C, subsequently samples were analyzed by western blot. Broad spectrum serine protease inhibitors (Aprotinin, Leupetin) were added during incubation. Aprotinin and Leupetin are inhibiting proteolytic degradation. The intact mAb (upper band) and the clipped mAb (lower band) are indicated by arrows. **b** Gene expression profiling of CHO-K1 versus CHO-A by NGS. Shown is the gene expression profile of “secreted/shedded members of the S1A trypsin–like serine protease family” for CHO-K1 and CHO-A cell lines using next generation sequencing. The gene expression analysis highlights that five proteases were more than 1.5 fold higher expressed in CHO-K1 cells (labelled with a red asterix). The y-axis shows the transcript abundance as RPKM (Reads Per Kilobase of exon model per Million mapped reads). **c** siRNA knock-down identifies matriptase as major clipping protease and CHO matriptase KO clone shows no detectable clipping activity. Upper figure: siRNAs directed against the five protease genes and scrambled (scr.) siRNA were transfected and conditioned medium was collected three days after transfection. The model mAb was incubated in fresh medium as control (first lane) and conditioned medium from the siRNA transfected cells. Samples were analyzed by western blot. Only siRNA targeting matriptase (ST14) showed reduced proteolytic degradation. The intact mAb (upper band) and the clipped mAb (lower band) are indicated by arrows. Lower figure: The model mAb was incubated for 48h in conditioned medium collected from wt CHO-K1 as well as the matriptase knockout clone. Samples were analyzed by western blot. The intact mAb (upper band) and the clipped mAb (lower band) are indicated by arrows. No proteolytic degradation could be detected in the samples originating from the matriptase KO clone. **d** Cell growth, viability and productivity in AMBR (fed batch with temperature shift). Cell growth, viability and volumetric productivity profiles of wt CHO-K1 (red circles, N=2) and matriptase KO clone (blue squares, N=1) cultivated in 15-mL AMBR. No significant differences were seen between WT and matriptase KO clone regarding cell growth and viability. Comparable or slightly higher productivity was detected for the matriptase KO clone compared to the WT. **e** Significant reduced proteolytic clipping applying matriptase KO clone. The model mAb was stable expressed in CHO-K1 (WT) as well as the CHO-K1 matriptase KO clone. Samples were analyzed by western blot. The intact mAb (upper band) and the clipped mAb (lower band) are indicated by arrows. Significant reduced proteolytic degradation could be detected in the samples originating from the matriptase KO clone (3 samples each is shown for wt and KO cells)

## O-010 Lipidomics for robust high performance process development

### Andréa McCann, Gregory Mathy, Laetitia Malphettes

#### UCB pharma, Braine l’Alleud, Belgium

##### **Correspondence:** Andréa McCann (andrea.mccann2@ucb.com); Gregory Mathy

**Background**

CHO cell lines are common hosts for the production of biopharmaceutical proteins. So far, considerable progress has been made increasing productivity of cell culture to meet the rapidly growing demand for antibody biopharmaceuticals through increased cell densities and longer culture times. The downside is the increase of the process related impurities, bringing new challenges for process and harvest development. Among the process related impurities such as host cell proteins (HCPs) or DNA the potential impact of lipids production and release during cell culture is still poorly understood due to the complex nature and diversity of this class of molecules. Thanks to recent advances in analytical tools especially mass spectrometry, the advent of lipidomics offers now the feasibility to study several thousands of lipid species thus unraveling the possibility to understand and potentially control the interactions between high performance bioreactor processes, harvest conditions and purification.

**Materials and methods**

In order to analyze and quantify lipids, we developed a three steps method. In a first step, lipids were extracted with Methyl tert-butyl ether (MTBE) according to Matyash method [1]. Lipids were then separated by liquid chromatography using either HILIC of reverse phase column prior to detection and quantification by mass spectrometry. All lipid classes were detected by ESI-MS/MS excepted cholesterol (APCI-MS/MS). Finally we applied this method to analyze the lipid content of different cell lines each expressing a different recombinant protein, during a 14 days fed batch process.

**Results**

Lipid from CHO cells were successfully extracted with a yield between 80% and 95% depending on the different lipid classes. Stable isotope labeled lipids were used as internal standard in order to have comparable results between batches. The obtained results (Fig. 1) show that for a given cell line, lipid distribution is changing over the process. Moreover, this distribution may vary significantly depending on the cell line: CL-1 and in a lower extend, CL-3, show an accumulation of triglycerides from day 6 to the end of the process, while CL-2 doesn’t seems to follow this trend.

**Conclusion**

Interestingly, in some cell lines/experimental conditions, we highlighted an overproduction of triglycerides and cholesterol leading to the accumulation of lipid droplets known as energy storage sink. At the metabolic level, these findings suggest a relative overflow of the carbon metabolism. From a process development perspective these findings can be considered on the one hand as a resource waste since the stored energy is not used for protein/biomass biosynthesis and on the second hand as the root cause of additional process challenges especially during the harvest and the first capture steps given the hydrophobic nature of these molecules. Implementation of lipidomics analysis enables us to highlight a new type of process variability and to anticipate potential problems for the downstream steps. The application of this methodology on our platform has helped us to design tailor made solutions (pretreatment selection, filter selection,…) at the clarification step which are now implemented in our harvest development platform approach.

**Acknowledgments**

Many thanks to Valentine Chevallier for her precious advices, to Stefanos Grammatikos for his support and to the whole Upstream Process Sciences team.

**Reference**

1. Matyash V, Liebisch G, Kurzchalia TV, Shevchenko A, Schwudke D: **Lipid extraction by methyl-tert-butyl ether for high-throughput lipidomics** J Lipid Res. 2008, **49**(5):1137-46.

**Fig. 1 (abstract O-010).**
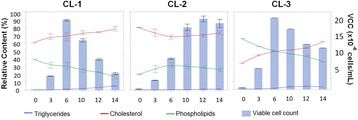
Lipid distribution for 3 different cell lines over the process

## O-020 High throughput analysis of antibody glycosylation in cell culture samples

### Sebastian Giehring, Kristina Lechner, Christian Meissner, Anna Johann, Christine Wosnitza

#### PAIA Biotech GmbH, Cologne, D-51105, Germany

##### **Correspondence:** Sebastian Giehring (sebastian.giehring@paiabio.com)

**Background**

The glycosylation of therapeutic proteins is a critical quality attribute (CQA) and needs to be analyzed during cell line and bioprocess development. The current methods for analyzing glycosylation are mainly based on the enzymatic release of glycans. They are tedious and offer only limited throughput, which makes them unsuitable for cell line development work. In this study we evaluated a novel PAIA assay for measuring intact glycoproteins with capture beads and fluorescence labeled plant lectins to analyze glycans in a high throughput 384-well plate format.

**Material and methods**

Analytes: Erbitux^©^, Mabthera^©^, Arzerra^©^ and Avastin^©^. Two glyco-engineered variants of one IgG were kindly provided by Merck (Vevey, Switzerland). All analytes were spiked into CHO-K1 cell culture supernatant or buffer, diluted 1:1 with a denaturation solution and incubated at 65 or 70°C for 20 minutes to expose the Fc glycans. Erbitux samples were analyzed under denaturing conditions to detect Fab- and Fc-glycosylation and in native conditions for Fab glycosylation only. 10μL of pretreated sample was added to each well of the special 384-well PAIAplate, containing labeled lectin and capture beads. The microplate was incubated for 45 minutes at 1800 rpm on an orbital shaker at room temperature and spun down at 500 xg. The read-out was done on a fluorescence microscope (SynenTec, Elmshorn, Germany) in less than five minutes.

**Results**

**Figure 1a: Lectin binding profiles of different IgGs.** The analysis of different IgG results in lectin binding profiles which show the different degrees in glycosylation. High abundance of sugars leads to high binding rates of the lectin for the respective sugar. Avastin has a very low degree of galactosylation and high mannose species compared to Mabthera and Arzerra (Fig. 1a). Only Arzerra is carrying glycans with 2-6 linked sialic acids. These findings are in line with results from literature [1].

**Figure 1b: Distinction between Fc and Fab glycosylation in Erbitux.** Without denaturation only the Fab glycans are detectable in Erbitux. Denaturation leads to additional exposure of the Fc glycans and thus higher lectin binding rates compared to native Erbitux. GNA and NPL only bind to denatured Erbitux indicating that the high mannose glycans are only present on the Fc part. The equal SNA binding rates for both conditions confirm that the 2-6 linked sialic acids are almost exclusively found on the Fab part. This is in agreement with published data [2].

**Figure 1c: Lectin binding rates correlate with the levels of galactosylation and fucosylation.** Increasing degrees of glycosylation in the mixtures of the glycan variants from Merck lead to higher lectin binding rates for all galactose and fucose markers proving that quantitative analysis can be performed with these assays. The ConA lectin which binds to the common core mannose glycan motive remains at the same level, suggesting that the Fc glycans were similarly exposed in all samples.

**Conclusions**

The results demonstrate that PAIA assays are capable of quickly detecting differences in glycan patterns of different antibodies. In addition it was shown that glycan variants of the same IgG can be analyzed quantitatively. And finally we could confirm the differences in Fab and Fc glycosylation in Erbitux. We believe that bead-based assays with lectins have a great potential for monitoring product quality early in the development process.

**Acknowledgements**

We thank David Bruehlmann and Thomas Vuillemin from Merck (Vevey, Switzerland) for providing the IgG glycan variants and the 2-AB-UPLC glycan data.

**References**

1. Fuller, S, et al.: **Assessing the Variability of an Innovator Molecule N-Glycan Profile**, Poster Prozyme Inc. 2012.

2. Ayoub D, et al.: **Correct primary structure assessment and extensive glyco-profiling of cetuximab by a combination of intact, middle-up, middle-down and bottom-up ESI and MALDI mass spectrometry techniques.** *Mabs 2013* Vol 5:5, 699-710.

**Table 1 (abstract O-020).** Specificity of lectins

Lectin	Specificity
LCA	Core Fucose
PSA	Core Fucose
ECL	Terminal β-Gal
RCA	Terminal β-Gal
GNA	High Mannose
NPL	High Mannose
SNA	Sia α 2-6 Gal
MAL-II	Sia α 2-3 Gal
ConA	Core Mannose

**Fig. 1 (abstract O-020).**
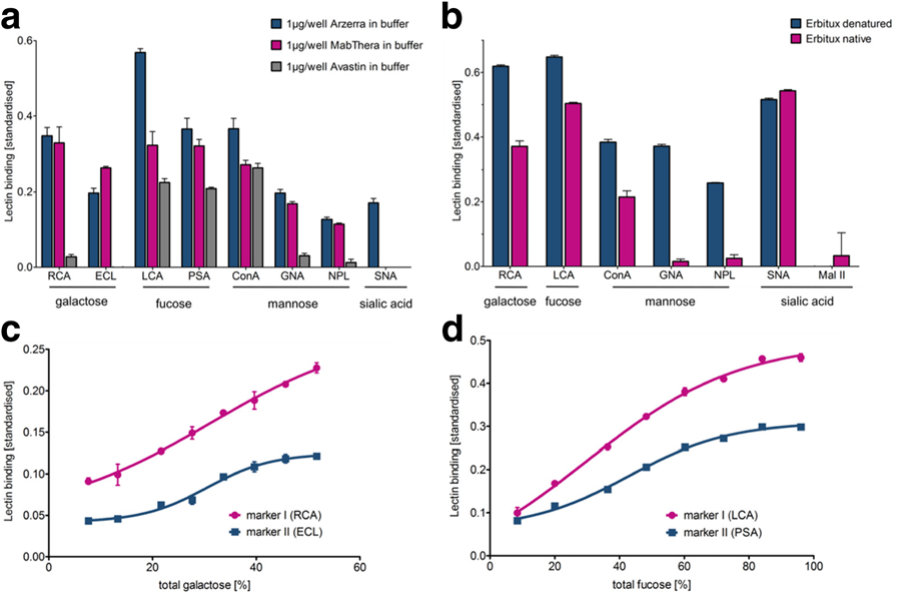
**a** Lectin binding profiles of different IgGs. **b** Distinction between Fc and Fab glycosylation in Erbitux. **c** Lectin binding rates correlate with the levels of galactosylation and fucosylation. **a** Lectin binding profiles of different therapeutic IgGs Arzerra, Mabthera and Avastin solutions of 200 μg/mL in CHO-K1 cell culture supernatant were denatured and the IgG glycans were characterized using nine different lectins. The lectin binding profiles match well with the glycan profiles reported in the literature. **b** Distinction between Fc and Fab glycosylation in Erbitux. Erbitux was diluted to a concentration of 200 μg/mL in TRIS buffer and measured in native and denatured conditions to distinguish Fab glycosylation (native Erbitux) from Fab and Fc glycosylation (denatured Erbitux). It could be confirmed that sialic acids are almost exclusively present on the Fab part of Erbitux and that the high mannose glycans are only found in the Fc part. **c** Lectin binding rates correlate with the levels of galactosylation and fucosylation. Two glycan variants samples of the same IgG from Merck were mixed in different ratios to yield glycosylation rates of 9 to 55% in terminal β-galactose and 8 to 100% in core-fucose, based on data from 2-AB UPLC analysis. The mixtures all contained 0.5 μg IgG per well. The measured lectin binding rates for all galactose and fucose markers correlate very well with the respective degree of glycosylation in the mixtures. All measurements were performed in triplicates

## P-004 Optimized PEI-based transfection reagents for production of clinical grade viral vectors

### Geraldine Guerin-Peyrou, Jelena Vjetrovic, Valérie Kédinger, Alain Cuzange, Alengo Nyama’antu, Patrick Erbacher

#### Polyplus-transfection, Bioparc,850 Boulevard S. Brant, 67400 Illkirch, France

##### **Correspondence:** Valérie Kédinger (vkedinger@polyplus-transfection.com)

**Background**

Gene- and cell therapy-based medicines are experiencing resurgence due to the introduction of “next generation” transfer viral vectors, which have demonstrated improved safety and efficacy. Adeno associated viruses (AAV) and lentiviruses are very commonly used in therapeutics and often produced by transient gene expression, using PEI-mediated transient transfection in HEK-293 or HEK-293T cells [1]. The critical raw materials needed for cGMP vector production must be sourced from approved suppliers and should have gone through a rigorous testing program to reduce the risk of introducing adventitious agents into the production process. Correspondingly, the PEI transfection reagent must also be sourced from a qualified supplier, and have gone through rigorous testing to ensure reliable transfection efficiencies, and hence reproducible virus production yields.

Here, we present PEIpro® and PEIpro®-HQ, the unique PEI-based transfection reagents suitable for use in process development and in cGMP biomanufacturing, respectively. Unlike commercially available PEIs, PEIpro® benefits from extensive research and development in polymer chemistry and formulation for mammalian cell transfection. We further demonstrate that PEIpro® and PEIpro®-HQ are the reagents of choice for virus production runs in most cell culture systems, hence facilitating the transition from initial optimization during process development up to large-scale therapeutic viral vector production in adherent or suspension cells.

**Materiel and methods**

*Manufacturing process of PEIpro® and PEIpro®-HQ re*agents**.**

PEIpro® and PEIpro®-HQ are fully synthetic reagents, free of any animal-origin components. In comparison to PEIpro®, a more extensive number of Quality Controls are performed on PEIpro®-HQ to enable its use as a qualified raw material in GMP processes for the manufacturing of clinical batches of therapeutic products.

*Lentivirus and AAV production* **.**

Irrespective of the cell culture vessel type, transfection using PEIpro® was performed following our recommandations. As an example, HEK-293T (lentivirus) and HEK-293 (AAV) cells were thawed directly into each medium and passaged every 3 to 4 days before going into a 2 Liter benchtop bioreactor. Cells were resuspended and cultured for 3 days before transfection with PEIpro®. HEK-293T cells were transfected with a third-generation system (four plasmids) for lentivirus production. HEK-293 cells were co-transfected with three plasmids for AAV production. Lentiviral and AAV titers were measured 48 and 72 hours post-transfection (Data kindly provided by Généthon).

**Results**

PEIpro® is the reagent of choice for virus production runs in most adherent and suspension cell culture systems from process development up to large scale clinical-grade virus production. Irrespective of the cell culture-based system and production scale, PEIpro® and PEIpro®-HQ have led to efficient viral vector yields in standard laboratory cell systems, such as in flasks, cell factories, and roller bottles, as well as in multilayers flasks or fixed-bed culture systems that take into account time and space concerns for the scaling-up process (Table 1).

For example, high viral vector yields superior to 10^7^ IG/mL and 10^11^-10^12^ VG/mL were obtained respectively for lentiviruses and AAVs in suspension HEK-293T and HEK-293 cells cultured in one of the commercially available synthetic cell culture medium BalanCD® HEK293 (Irvine Scientific®).

**Conclusion**

PEIpro® and its higher quality grade PEIpro®-HQ are the unique PEI suitable for efficient and reproducible production of therapeutic viral vectors. Efficient viral vector production yields can be achieved in most cell culture systems, irrespective of the production scale. With appropriate and advanced quality controls, the highest quality grade PEIpro®-HQ is commercially available to accompany academics and biopharmaceutical companies in terms of qualified raw material for their GMP-grade viral vector production needs.

**Acknowledgments**

Polyplus-transfection would like to thank Généthon for their kindly provided data.

**Reference**

1. Merten et al. Methods & Clinical Development 3:16017 (2016).

**Table 1 (abstract P-004).** PEIpro®, the reagent of choice for virus production runs in most cell culture systems in both adherent and suspension cells. Irrespective of the cell culture-based system and production scale, PEIpro® and PEIpro®-HQ have led to efficient viral vector yields superior to 10^7^ IG/mL and 10^9^ VG/mL, respectively for lentiviruses and AAVs

Cell culture system	Vector	Cells	Titer
CS10®/CF10®	AAV	Adherent HEK-293, HEK-293T	10^11^-10^13^ VG/ml
Fixed-bed bioreactor (iCELLIS®)	AAV	Adherent HEK-293T	10^14^-10^16^ VG/ml
Shaker Flask	AAV	Suspension HEK-293, HEK-293T	10^9^-10^10^ VP/ml
Bioreactor	AAV	Suspension HEK-293, HEK-293T	0.8-1.5 x10^9^-10^10^ VG/ml
10 cm dish/75 cm^2^	Lentivirus	Adherent HEK-293, HEK-293T	1-2 x10^8^ TU/ml
HYPERflask®/HYPERstack®	Lentivirus	Adherent HEK-293, HEK-293T	1-2 x10^8^ TU/ml
Shaker Flask	Lentivirus	Suspension HEK-293F, HEK-293T	2x 10^7^-10^10^ VP/ml
Bioreactor	Lentivirus	Suspension HEK-293, HEK-293T	10^7^ IG/ml

**Fig. 1 (abstract P-004).**
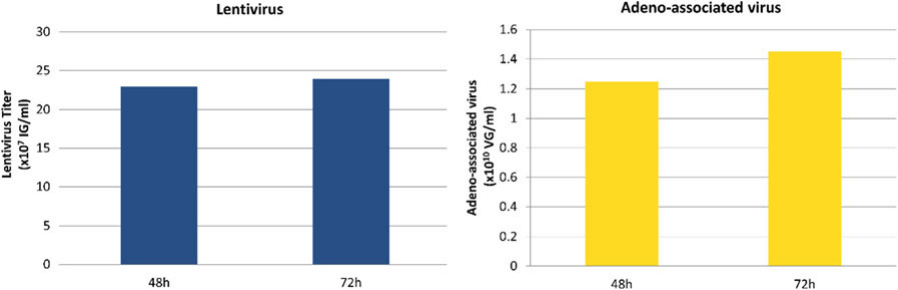
Lentivirus and AAV production in HEK-293T and HEK-293 cells grown in suspension in BalanCD® HEK293 (Irvine Scientific®). HEK-293T (lentivirus) and HEK-293 (AAV) cells were thawed directly into each medium and passaged every 3 to 4 days before going into a 2 Liter benchtop bioreactor. Cells were seeded and cultured for 3 days before being transfected by PEIpro® (Polyplus). For transfection, four plasmids were used for lentivirus and three plasmids were used for AAV. Lentiviral and AAV titer were measured 48 and 72 hours post transfection (Data kindly provided by Généthon)

## P-014 Inoculum performance under different culture conditions: “One clone, multiple behaviour”

### Chandrashekhar K. Nanjegowda, Sasikumar Karunakaran, Kumaran Sivalingavasu, Ritika Lakhotia, Sohini Jana, Reetesh P. Mahalingannanavar, Dinesh Baskar, Saravanan Desan, Ankur Bhatnagar, Anuj Goel

#### Cell Culture Lab, Biocon Research Limited, Biocon Special Economic Zone, Plot No. 2 & 3, Bommasandra Jigani Link Road, Bangalore 560 099, India

##### **Correspondence:** Chandrashekhar K. Nanjegowda (chandrashekhar.kuravangi@biocon.com)

**Background**

In mAb manufacturing processes, production bioreactor step is considered to be crucial as the product is produced in this step. However, the cells spend a significant portion of process time in the inoculum stage starting from vial thaw to expansion in shake flasks and seed bioreactors. During these stages, the cells encounter environmental changes in parameters like pH, Osmolality, concentration of nutrients and waste metabolites. These small changes can have a profound impact on the cells when experienced over multiple passages. The effect of this cumulative impact is not apparent during the seed stage(s) but can be evident in the production step.

In this work, we present the data from experiments with cells maintained under different inoculum culture conditions and evaluating its behaviour in terms of growth performance in production bioreactor.

The following case studies were conducted where the changes were made in the inoculum step. The cells from the inoculum step which were exposed to these changes over multiple passages were used to run production batches with control conditions and the impact in the production step was observed as a response.

Case Study 1: Effect of incubation at different CO_2_ levels

Case Study 2: Effect of incubation at different Temperature

Case Study 3: Modification of seed medium buffer composition

**Materials and methods**

Table 1

**Results**

Results indicated that when the cells were subjected to various culture conditions for multiple passages (like different temperature, CO_2_ levels and medium composition), the growth in inoculum stages (doubling time and viability) did not get impacted. However, when these cells were used in the production bioreactor, significant impact was observed on process performance.

As illustrated in the Fig. 1a, the cells which were maintained at higher CO_2_ levels and lower temperature during inoculum stages showed improved cell concentration in the production bioreactor. However the cells sub-cultured in higher temperature showed reduced growth performance. When the cells were maintained in the same media with two different buffering components (Fig. 1b), a shift in lactate production was observed in the inoculum stage itself. These effects became predominant in production culture.

The ability to modulate the inoculum conditions was exploited to improve cell line stability. In one of the clone, cell growth and viability in the production bioreactor was observed to decrease with the increase in age of inoculum (Fig. 1c & d). However with the use of alternate medium (buffering, medium components and culture conditions) used for sub-culturing, improvement in cell line stability was achieved.

**Conclusions**

The results of our experiments indicate that cells when maintained under different inoculum culture conditions can behave significantly different when used in the production process. We also identified that these conditions impact the overall cell line stability of a clone. Hence a thorough understanding of the cells and its metabolism is required. The parameters which predict its behavior must be controlled as per the requirement of the process. Manipulation of culture conditions suitably in the early inoculum propagation steps enables improving performance and stability of a cell line, thereby increasing process robustness. Also, we propose that the model to study the inoculum parameters effect should include production run where the impact of the inoculum parameter will be more pronounce.

**Acknowledgements**

Cell Culture Team, Biocon

**Table 1 (abstract P-014).** Material and methods used for the work described

Parameter	Description
Cell lines	Recombinant CHO Cells expressing monoclonal antibody
Media	Chemically defined cell culture media
Sub-Culturing	Every 2-3 days, cells are sub-cultured with fresh medium and incubated in CO_2_ shaker incubator
Production Run	Production runs were conducted in 1L scale bioreactor in fed batch mode
Analysis	Cell Concentration & Viability (Cedex HiRes), Product titer using Protein A HPLC, Lactate (YSI)

**Fig. 1 (abstract P-014).**
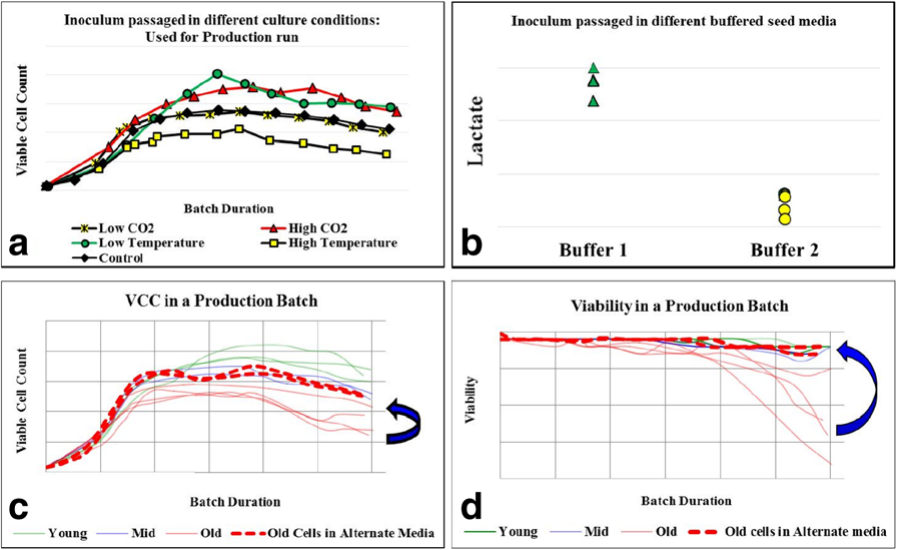
Data from control production experiments started with inoculum maintained in different conditions (**a**) Temperature and CO_2_ levels, (**b**) The Lactate levels observed when cells maintained in media with different buffering components, (**c & d**) represents the improvement in cell line stability with altered medium

## P-015 Advantages and challenges of a continuous upstream process

### Janani Kanakaraj, Mandeep Kaur, Abdul Waheed, Bala Murugan S., Dinesh Baskar, Saravanan Desan, Ankur Bhatnagar, Anuj Goel

#### Cell Culture Lab, Biocon Research Limited, Biocon Special Economic Zone, Plot No. 2 & 3, Bommasandra Jigani Link Road, Bangalore 560 099, India

##### **Correspondence:** Janani Kanakaraj (janani.kanakaraj@biocon.com)

**Background**

Continuous perfusion process is making a comeback as a competing upstream manufacturing technology for the production of Biopharmaceuticals compared to the standard fed batch processes. This is primarily because of cost advantages such as reduced capital cost and increased product yield. The change in status of perfusion process from older perfusion to the new-age perfusion is due to availability of better cell retention devices leading to more efficient processes, improved cell lines, cell culture medium capable of supporting high cell densities and better bioreactor control strategies. In this work, we present the advantages, limitations and challenges of fed batch and perfusion type of processes through case studies.

**Materials and methods**

Table 1

**Results**

**Case 1: Comparison between fed-batch and perfusion process**

The Perfusion run yielded 5-fold higher titer compared to Fed batch run (Fig. 1a). Considering the number of runs that could be executed in a manufacturing facility within the same calendar days, about 1-fold increase in product output can be achieved with the perfusion process (Fig. 1a). This difference is attributed to higher IVCC, higher PCD and longevity of cells because of decreased level of toxic metabolite concentrations such as lactate and ammonia.

**Case 2: Understanding product retention in perfusion process**

The new-age perfusion processes utilize hollow fiber filters. This has been observed to cause retention of product within the bioreactor especially towards the end of the production run. Two types of experiments were conducted to study the factors contributing to product retention:

- Spiking studies:
  - Role of product titer: product was spiked into chemically defined media
  - Role of different cell viability: different broths with varying viability spiked with same product titer
- Evaluation of different hollow fiber membrane (M1, M2 and M3) on product retention.

From spiking studies, it was evident that cell debris and poor quality cell broth (lower viability) were the major factors contributing to product retention (Fig. 1c). From the different membranes experiments, it was identified that at Pilot scale, M1 showed much higher retention from the first perfusion cycle itself and it increased to more than 75% towards the end of the batch. However, with M2 membrane, product retention started only late (after 50% of batch duration) and it remained low (~20-40%). On the contrary, this difference was not observed at 1L scale due to the usage of membranes with larger filter area (2-3 folds higher compared to pilot scale). When the filter area per unit volume of perfusate was decreased by half (M2_Batch 4) for the pilot scale, even M2 showed retention profile similar to M1 (Fig. 1d).

**Conclusions**

We presented data to show that perfusion process has 5-fold increase in product yield on a per-batch basis and a 3-fold increase when facility throughput is considered. Product retention is a technical challenge that requires optimization (perfusion rates and filter membrane types). We believe it is imperative that labs that develop processes for biologics can now consider both perfusion and fed-batch based processes as both these technologies can now closely compete with each other. The choice of the process format going forward should now solely be dependent on the requirement for the biologic rather than the earlier perception that fed-batch is the preferred choice because of its simplicity.

**Acknowledgements**

Asmita Mukerji, Reetesh PM, Sasi Kumar K, Pilot plant team

**Table 1 (abstract P-015).** Material and methods used for the work described

Parameter	Description
Cell lines	Recombinant CHO cells expressing monoclonal antibody
Media	Chemically defined cell culture media
Process mode	Fed-batch and Perfusion
Analysis	Cell concentration and viability were determined using automated cell counters (Cedex HiRes). Product titer of perfusate and harvest were measured using Protein A HPLC

**Fig. 1 (abstract P-015).**
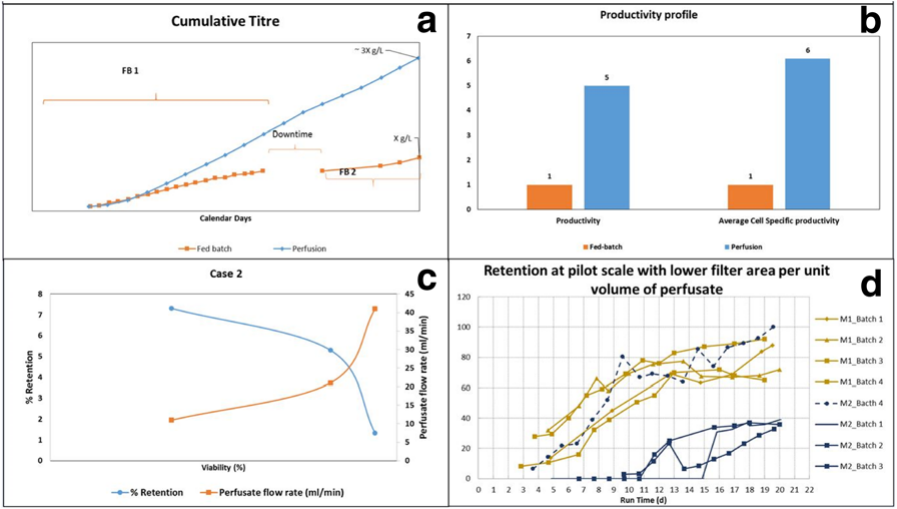
**a** Product output comparison of perfusion and fed batch, **b** Comparison of fed-batch and continuous processes - productivity and Average cell specific productivity, **c** Cell viability affecting product retention **d** Product retention with different hollow fibre membrane types

## P-023 Differential analysis of IgG product quality by intact mass analysis for fed-batch-cultivated CHO cells under glucose limitation

### Benjamin Müller^1^, Anica Schmidt^2^, Christoph Heinrich^3^, Heino Büntemeyer^1^

#### ^1^Biofidus AG, Bielefeld, 33613, Germany; ^2^Institute of Cell Culture Technology, Bielefeld University, Bielefeld, 33615, Germany; ^3^Xell AG, Bielefeld, 33689, Germany

##### **Correspondence:** Benjamin Müller (benjamin.mueller@biofidus.de)

**Background**

Chinese hamster ovary (CHO) cell culture has been widely used for production of monoclonal antibodies in the pharmaceutical industry. Previous studies have shown that the cell specific productivity in CHO cells can be increased by glucose limitation [1]. Introducing a productivity enhancing effect it is possible that this also affects the quality of the product such as glycosylation or other posttranslational modifications. In this work, we are focusing on the impact of glucose limitation and increased productivity on the product quality of a monoclonal antibody produced in a fed-batch cultivation of CHO cells.

**Materials and methods**

CHO cells were cultivated both under limiting and non-limiting nutrient conditions in fed-batch. For fed-batch cultivation the reduced range for glucose concentration was chosen between 0.2 and 0.5 g/L. Reference cultivation was performed between 1.5 and 3.0 g/L. Both cultures were fed with similar volumes of a complex nutrient supplement. All cultivations were performed in chemically-defined, animal-component free CHO growth media (Xell AG). Viable cell density and viability were determined using the automated cell counting system CEDEX (Roche Diagnostics), glucose and lactate concentrations were detected via YSI (YSI life sciences). Amino acid were quantified using HPLC-FLD, vitamins were quantified using reversed phase chromatography coupled to a triple quadrupol mass spectrometer (Varian 320, selected reaction monitoring). Amounts of IgG1 were quantified via Protein A HPLC, mAb purified from another CHO cell clone was used as a standard. The analysis of product quality was performed by intact mass analysis using reversed phase chromatography coupled to a microOTOF-Q II mass spectrometer (Bruker Daltonik).

**Results**

The CHO cell culture cultivated under low nutrient conditions reached a 54% higher viable cell density than the reference culture (Fig. 1a). The product titer was even increased by 109% (Fig. 1b). The spent media analysis shows that some amino acids and vitamins were present at presumably limiting concentrations after day 5/6, mostly in the low nutrient level culture (down to 40 to 190 μM for TYR, GLN, ARG, and ASN, below 1 μM for pyridoxine, data not shown). The product quality showed significant changes for the changed feeding strategy (Fig. 1c and d). As expected, the glycation level decreased from 3% to 1% compared to the reference culture. The truncation level of C-terminal lysine at the heavy chain of the mAB increased from 79% to 88%. The glycosylation was also significantly influenced by the low nutrient level (Fig. 1e): The non-fucosylated variants increased from 3% to 6% (Fig. 1f), the degree of galactosylation increased from 31% to 39% (Fig. 1g).

**Conclusions**

Cultivation under low nutrient level led to 54% higher viable cell density and a product titer increased by 109% when compared to reference culture grown under non-limiting nutrient conditions. The analysis of product quality reveals 75% less glycation of light chain for CHO cells grown under low nutrient conditions (0.7% vs 2.7% in reference culture). The truncation of C-terminal lysine decreased by 10% (from 88% to 79%), the degree of galactosylation increased by 23% (from 31% to 39%, also observed by Takuma et al. [2]) and non-fucosylated glycans increased by 105% (from 2.8% to 5.8%) under low nutrient conditions. The product quality analysis by intact mass proved to be highly robust (average CV for four replicates = 2%).

In summary, cultivation with alternative feed led to higher IgG product titer and better product quality (glycation unwanted, higher amount of non-fucosylated glycans leads to higher antibody-dependent cell-mediated cytotoxicity (ADCC), higher amount of galactosylation to higher complement-dependent cytotoxicity (CDC) and ADCC [3, 4]).

**References**

1. Wingens M, Gätgens, J, Albaum, S, Büntemeyer, H., Noll, T., Hoffrogge, R.: **2D-DIGE screening of high productive CHO cells under glucose-limitation – basic changes in the proteome equipment and hints for epigenetic effects**. *Journal of Biotechnology 2015*, 201: 86-97.

2. Takuma, S, Hirashima, C, Piret, J.M.: **Dependence on Glucose Limitation of the pCO2 Influences on CHO Cell Growth, Metabolism and IgG Production.** *Biotechnology and Bioengineering 2007*, 97, 6: 1479-1488.

3. Thomann, M, Reckermann, K, Reusch, D, Prasser, J, Tejada, M.L.: **Fc-galactosylation modulates antibody-dependent cellular cytotoxicity of therapeutic antibodies.** *Molecular Immunology 2016*, 73: 69-75.

4. Reusch, D, Tejada, M.L.: **Fc glycans of therapeutic antibodies as critical quality attributes.** *Glycobiology 2015*, 25, 12: 1325-1334.

**Fig. 1 (abstract P-023).**
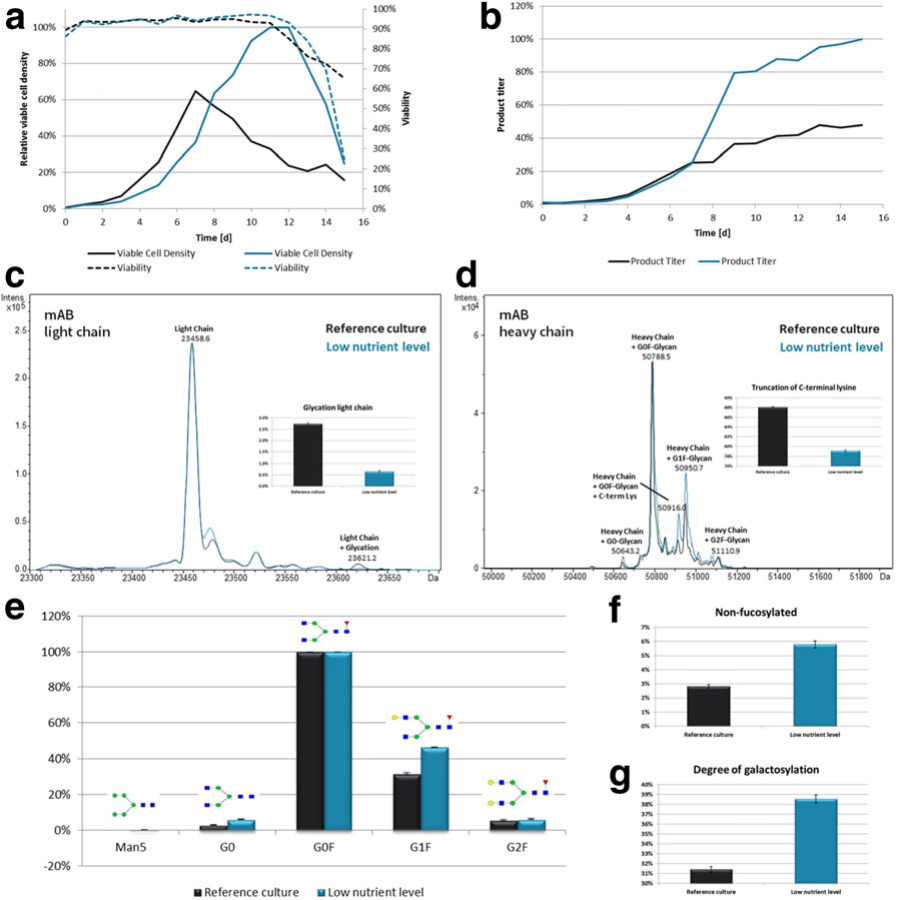
**a** Growth curve of reference culture vs. low nutrient level; **b** Product titer of reference culture vs. low nutrient level; **c** and **d** Analysis of product quality of reference culture vs. low nutrient level by intact mass measurement; **e** Comparison of glycosylation profile of IgG product for reference culture vs. low nutrient level; **f** and **g** Comparison of product quality for reference culture vs. low nutrient level

## P-031 How cell culture automation benefits upstream process development

### Carsten Musmann (carsten.musmann@roche.com)

#### Roche Diagnostics GmbH, Pharma Biotech Production and Development, Penzberg, 82377, Germany

**Background**

We developed an automated, multiwell plate (MWP) based screening system for suspension cell cultures (Fig. 1a) which is now routinely used in cell culture process development. It is characterized by a fully automated workflow with integrated analytical instrumentation. It uses shaken 6-24 well plates as bioreactors which can be run in batch and fed-batch mode with a capacity of up to 768 reactors in parallel [1-3]. A wide ranging analytical portfolio to monitor cell culture processes and also a cooperation with internal high throughput (HT) analytic groups to characterize product quality are available. In addition the use and the benefits of spectroscopic methods for cell culture automation were shown in the past [4, 5].

**Materials and methods**

Automated cell culture systems enable broader screening within a shorter time frame for many applications in upstream process development. The higher degree of parallelization and automation helps to screen for most promising parameters in a shorter time. The use of broad DoE screening design allows in addition the identification of parameters that support high titers while keeping high product quality (multiple factors at the same time). The illustration (Fig. 1b) shows an example how this combination can speed up process development steps. Main applications of the cell culture automation are for example the identification of product quality levers and media or feed optimization.

**Results**

The application of the cell culture automation is shown for two examples. The goal in the first application was to identify levers to reduce trisulfides. By a screening of 39 conditions in parallel (in 4-fold replication, 158 wells in sum) the reduction of Trisulfides by 97.5 % (normalized to start level) was possible. In addition the levers for Trisulfide reduction were identified. The best and start conditions were verified in bioreactor scale (Fig. 1c).

The goal in the second application was to increase product concentration without an impact on product quality. By a screening of 54 conditions in parallel (in 4-fold replication, 216 wells in sum) the increase of titer from 1.5 g/L to 3.7 g/L (> factor 2) was possible by media platform change and media optimization. An impact on product quality could not been shown. The best conditions were also verified in bioreactor scale (Fig. 1d).

**Conclusion**

The benefits of using cell culture automation in late stage process development were shown based on two examples of current applications. For this purpose the experimental results of the development work of two late state projects using the in-house developed automated cell culture system were shown. The first example shows the capability of the automated cell culture system by reducing trisulfides significantly in just one experiment. For the other project the final product concentration could be increased by factor 2.5 by a media screening and changing to the in-house media platform. These two examples show the potential of cell culture automation as a routine tool in process development.

**Acknowledgements**

The author would like to thank the cell culture automation team (J. Hoffmann, G. Pechmann, C. Schuster), all internship and diploma students (S. Spielmann, K. Müller, B. Frommeyer, J. Wisbauer, A.Gutknecht), former members of the cell culture automation team (K. Joeris, S. Markert), the Roche Penzberg pilot plant team and all Roche Penzberg portfolio project teams.

**References**

1. Markert, S.,Joeris, K.: **Development of an automated, multiwell plate based screening system for suspension cell culture.** BMC Proc. 2011, 5(Suppl 8): O9.

2. Markert, S., Musmann, C.,Joeris, K.: **Development and application of an automated, multiwell plate based screening system for suspension cell culture.** BMC Proc. 2013, 7(Suppl 6): P113.

3. Markert, S.,Joeris, K.: **Establishment of a fully automated microtiter plate-based system for suspension cell culture and its application for enhanced process optimization.** Biotechnol Bioeng. 2017, 114(1): 113-121.

4. Musmann, C., Joeris, K.,Markert, S.: **Spectroscopic tools for an automated suspension cell culture screening system.** BMC Proc. 2015.

5. Musmann, C., Joeris, K., Markert, S., Solle, D.,Scheper, T.: **A review of spectroscopic methods for high-throughput characterization of mammalian cell cultures in automated cell culture systems.** Engineering in Life Sciences. 2015.

**Fig. 1 (abstract P-031).**
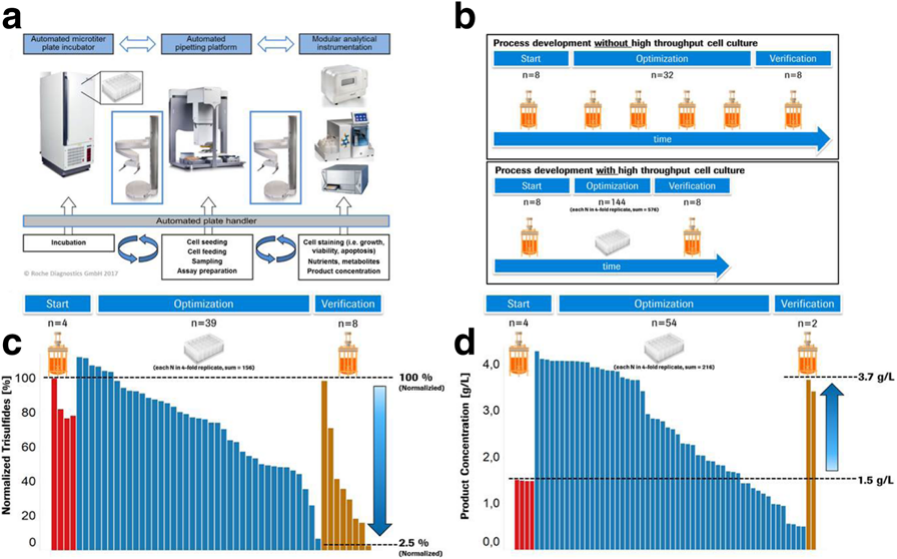
**a** Schematic illustration of the automated cell culture system. Only the core system is shown with a robotic plate handler as key device connecting cultivation, processing and analytical parts. **b** Illustration of an example how cell culture automation can speed up process development steps. **c** Application in the identification of product quality levers. **d** Application in titer optimization

## P-038 A novel platform for high throughput cell line screening and development

### Christoph Freiberg^1^, Lukasz Gricman^1^, Gian Andrea Signorell^1^, Lukas Flueck-Kabay^1^, Amanda Fitzgerald^2^, Maria Wendt^1^, Yang-Chieh Chou^3^, Hans Peter Fischer^1^

#### ^1^Genedata AG, Basel, Switzerland; ^2^Genedata Inc., Boston, MA, USA; ^3^Genedata Inc., San Francisco, CA, USA

##### **Correspondence:** Christoph Freiberg (bioprocess@genedata.com)

**Background**

The cell line development process has become faster and is simultaneously generating more clone- and product-related analytical data. In order to select the best producer cell line, extremely heterogeneous data types need to be systematically compared. The timely availability of all data needed to decide which cell line to pursue has become a bottleneck in the cell line development workflow. To ensure sound decision making, new integrated workflow support and data analysis methods are needed.

**Materials and Methods**

We have developed a new end-to-end platform for bioprocess development, which includes a cell line development workflow system supporting seeding, selection, passaging, analyzing, cryo-conservation, and processing in (micro-) bioreactors. This platform, Genedata Bioprocess™, enables partially or fully automated cell line selection and assessment processes, and it increases process efficiency and quality. The system tracks the full history of all clones - from initial transfection all the way to their evaluation in bioreactor runs - and combines this information with analytics data on molecules, clones, and product quality. It can directly integrate with all instruments, such as pipetting robots, bioreactors, and bioanalyzers. The system is designed for a wide range of biologic molecules, including antibodies (IgGs, novel formats) and other therapeutic proteins (e.g., fusion proteins).

**Results and Conclusions**

Highlighted use cases describe the identification of top producer cell lines, decision making support, bioreactor data management, and full clone history report documentation (Fig. 1). Genedata Bioprocess, which was developed in collaboration with top pharmaceutical companies, can flexibly support various (non-linear) workflows and structure the collected information in a way that fosters collaboration across an organization. While increasing throughput is crucial to ensure the timely availability of optimal producer cell lines, high-throughput is only possible when automated processes in the laboratory and the resulting data collection and aggregation can be streamlined. Genedata Bioprocess helps to establish more productive processes by offering support and integration for automation stations and measurement devices. Thanks to the comprehensive workflow support and the possibility to integrate results from cell line stability experiments, product quality assessment, and bioreactor suitability tests, Genedata Bioprocess provides a unique way to evaluate cell lines. Comprehensive analysis of all data collected in the process helps to ensure the highest possible quality and minimize the time and resources needed for data analysis and management. Integration of bioreactor data analysis and visualization with other parameters measured in cell line development, streamlines clone evaluation in micro-bioreactors and supports high-throughput operations. Genedata Bioprocess comprehensively tracks the full clone history from the origin of the host cell line to the generation of the validated monoclonal producer cell line. For promising clones, the clone history report can be generated with one click. Besides supporting cell line development, Genedata Bioprocess is a comprehensive platform capable of tracking the complete bioprocess development process.

**Acknowledgements**

Allison Kurz, Genedata AG, Basel, Switzerland

**Fig. 1 (abstract P-038).**
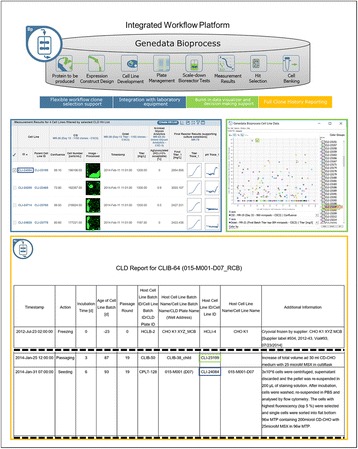
Scheme of the complete cell line development workflow support in Genedata Bioprocess. Showcasing integration of data from diverse measurement instruments, data visualization for decision making support as well as, tracking of full clone history

## P-043 Clarification of Oncolytic Measles Virus suspension using Charged Depth Filters

### Daniel Loewe^1^, Tanja A. Grein^1^, Hauke Dieken^1^, Tobias Weidner^1^, Denise Salzig^1^, Peter Czermak^1,2,3^

#### ^1^Institute of Bioprocess Engineering and Pharmaceutical Technology, University of Applied Sciences Mittelhessen, Wiesenstraße 14, 35390 Giessen, Germany; ^2^Department of Chemical Engineering, Kansas State University, Manhattan, KS, USA; ^3^Fraunhofer Institute for Molecular Biology and Applied Ecology (IME), Project group Bioresources, Winchesterstr. 3, 35394 Giessen, Germany

##### **Correspondence:** Daniel Loewe (daniel.loewe@lse.thm.de)

**Background**

In 2012, 14.1 million people suffered from cancer [1] making it to a major concern of our society. Since common cancer treatment is limited and not effective for late stage carcinoma, alternative methods are needed to reduce the high mortality rate of cancer patients. One alternative approach is the application of the oncolytic Measles virus (OMV), because OMV has a natural affinity against cancer cells. The major drawbacks of OMV is to produce the extremely high amount of at least 10^11^ TCID_50_ (50 % tissue culture infective dose) per dose [2] which is needed. To solve this problem, a high titer process must be established including an efficient downstream processing (DSP). We developed an appropriate upstream processing and are able to produce 10^10^-10^11^ TCID_50_ mL^-1^ in a bioreactor with 0.5 L working volume [3]. Now, we focus on the DSP part. The following study tested the application of charged depth filters for the OMV clarification. In contrast to common DSP schemes, a depletion of virus particles or a loss of infectivity is not desired. The aim is a reduction of protein content and DNA with minimal loss of infective OMV. Further, we investigated the influence of the cell culture medium on the depth filtration process.

**Material and Methods**

To explore the influence of the surrounding cell culture medium on the depth filtration performance, OMV was either produced in serum-free medium (VP-SFM) or serum-containing medium (DMEM + 10% FCS). The production was done in a STR with a working volume of 0.5 L as described in [3]. Cells and carriers were separated with an Opticap XL 1-module (Polygard-CR; 5 μm; Merck).

For the depth filtration Millistak+ CE50 filters (Merck) were used. The filter material was autoclaved and rinsed with 25 mL of 20 mM Tris-HCl (pH=7.4). The virus suspension was filtered with a load of 50 L m^-2^ using a peristaltic pump (ISM931C; Ismatec) applying a flux of 150 L m^-2^ h^-1^ (Fig. 1). Samples were collected at the beginning and end of a filtration run.

The OMV titer (TCID_50_ mL^-1^) of the samples was determined according to Kärber and Reed [4, 5]. Protein content was measured with the Pierce BCA protein assay kit (ThermoFisher Scientific) according to the manufacturer’s instructions. DNA was measured by a microtiter assay using Quant-iT PicoGreen dsDNA reagent (ThermoFisher Scientific) according to the manufacturer’s instructions.

**Results**

We found that positively charged depth filters were suitable to clarify OMV suspensions. The cell culture medium, in which the OMV was produced, influenced the outcome of the depth filtration. A log reduction value (LRV) of 0.87 was determined for OMV present in serum-containing medium (SCM), whereas the titer of OMV in serum-free medium (SFM) was reduced 1.63 log levels. This indicates that without serum in the surrounding liquid, OMV will adsorb to the filter material. However, we must evaluate if the missing serum or other components present in SFM are responsible for this effect.

Total protein was not relevantly reduced by the clarification using charged depth filters. For OMV present in SCM, the residual protein content was slightly less compared to OMV present in SFM (Table 1). In contrast, host cell DNA (hcDNA) was bound to the filter material. We achieved a 33% reduction of hcDNA for an OMV suspension in SFM. After clarifying an OMV suspension in SFM, the remaining hcDNA content was even lower being only 42 %.

**Conclusions**

Charged depth filters are suitable for the first clarification step of OMV downstream processing. Residual protein could pass the depth filter almost unhindered, whereas the hcDNA content was already reduced to 42% at maximum. However, the OMV titer was also reduced by the depth filtration. This undesired effect was stronger for the OMV present in SFM. Because the agencies require avoiding serum in clinical-grade production processes, this is disadvantageous. Nonetheless, because SFM will be soon standard for OMV production, further experiments have to be done preventing the OMV reduction during clarification. One option can be to reduce the adsorption strength of the virus to the filter material by the addition of salt. Moreover, it is important to establish a standardized protocol for the upstream processing. We determined batch-to-batch variations within the clarification indicating a strong impact of upstream processing (USP) on the outcome of the DSP. Therefore, further studies must investigate the influence of USP parameter e.g. time of harvest and pH of the harvest solution on the OMV.

**References**

1. Torre LA, Bray F, Siegel RL, Ferlay J, Lortet-Tieulent J, Jemal A. **Global cancer statistics, 2012**. CA Cancer J Clin. 2015;65:87–108. doi:10.3322/caac.21262.

2. Russell SJ, Federspiel MJ, Peng K-W, Tong C, Dingli D, Morice WG, et al. **Remission of disseminated cancer after systemic oncolytic virotherapy.** Mayo Clin Proc. 2014;89:926–33. doi:10.1016/j.mayocp.2014.04.003.

3. Grein TA, Schwebel F, Kress M, Loewe D, Dieken H, Salzig D, et al. **Screening different host cell lines for the dynamic production of measles virus.** Biotechnol Prog. 2017;33:989–97. doi:10.1002/btpr.2432.

4. Kärber G. **Beitrag zur kollektiven Behandlung pharmakologischer Reihenversuche.** Archiv f. experiment. Pathol. u. Pharmakol. 1931;162:480–3. doi:10.1007/BF01863914.

5. Reed LJ, Muench H. **A simple method of estimating fifty per cent endpoints.** American Journal of Epidemiology. 1938;27:493–7. doi:10.1093/oxfordjournals.aje.a118408.

**Fig. 1 (abstract P-043).**
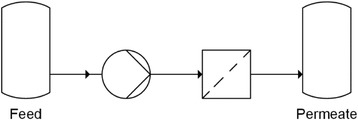
Experimental setup of depth filtration – OMV, produced in SCM or SFM, was filtered through a positively charged depth filter (Millistak+ CE50; Merck) (Load: 50 L m^2^ and flux: 150 L m^-2^ h^-1^)

**Table 1 (abstract P-043).** Virus titer of OMV present in serum-containing (SCM) or serum-free medium (SFM), before (feed) and after (permeate) depth filtration. Additionally, total protein and host cell DNA (hcDNA) were determined

Medium	Sample	Log. virus titer (-)	Residual total protein (%)	Residual hcDNA (%)
SCM	Feed	6.39 ± 0.23	100 ± 5	100 ± 13
	Permeate	5.52 ± 0.48	92 ± 4	67 ± 4
SFM	Feed	6.16 ± 0.55	100 ± 10	100 ± 9
	Permeate	4.53 ± 0,23	96 ± 7	42 ± 3

## P-047 Development of DoE based fed-batch strategies for high-producing CHO cell cultures

### David Reinhart^1^, Andreas Castan^2^, Lukas Damjanovic^1^, Barbara Holub^3^, Renate Kunert^1^

#### ^1^Vienna Institute of BioTechnology, Department of Biotechnology, University of Natural Resources and Life Sciences, Vienna, Muthgasse 11, 1190 Vienna, Austria; ^2^GE Healthcare Bio-Sciences AB, Björkgatan 30, 751 84 Uppsala, Sweden; ^3^GE Healthcare Bio-Sciences AB, Kremplstraße 5, 4061 Pasching, Austria

**Background**

Fed-batch culture is commonly employed to maximize cell and product concentrations in upstream mammalian cell culture processes. Typical standard platform processes rely on fixed-volume bolus feeding of concentrated feed supplements at regular intervals. However, such static approaches might result in over- or underfeeding. To mimic more closely the dynamics of a fed-batch culture, we developed a dynamic feeding strategy responsive to the actual nutrient needs of a mAb-producing recombinant CHO cell line.

**Materials and methods**

Model cell line was a mAb-expressing CHO DG44 (licensed from Cellca GmbH) cultivated in HyClone™ CDM4NS0 (GE Healthcare) supplemented with glutamine. Feed media were HyClone Cell Boost™ 1, 2, 3, 4, 5, 6, 7a and 7b feed supplements (all GE Healthcare). Analytics comprised cell concentration, viability, mAb titer, selected metabolites, osmolality and amino acids. For fed-batch development, we applied a Design of experiments (DoE) approach using MODDE™ statistical software (Umetrics AB) combined with a three-step strategy, as follows:

- Step 1: Selection of the best performing Cell Boost supplements (batch)
- Step 2: Fine-tuning of feed ratio of selected Cell Boost supplements (fed-batch)
- Step 3: Feed strategy development: constant vs. dynamic (fed-batch)
- Bioreactor: Verification of the best results (step 3) under controlled conditions (fed-batch)

**Results and discussion**

Improvements made at different steps during fed-batch development are shown in Fig. 1. At step 1, all eight Cell Boost supplements were added to CDM4NS0 according to a DoE approach, and batch cultures were performed. This evaluation allowed us to select only those Cell Boost supplements that were beneficial to the overall culture performance. Non-performing Cell Boost supplements were removed and not considered further.

At step 2, the selected Cell Boost supplements were added daily to the cultures at different ratios according to a DoE approach, and fed-batch cultures were conducted. As expected, daily feed additions to replenish consumed nutrients substantially improved mAb and peak cell concentrations as well as viable cumulative cell days (VCCD) compared to batch cultivation. Further, the results enabled us to fine-tune the feed ratio of selected Cell Boost supplements.

At step 3, we further optimized the best performing feed ratio by investigation of static and dynamic feed protocols. Most fed-batch protocols rely on constant feed additions on distinct days. However, these approaches often lead to substantial over- or underfeeding during bioprocessing. To improve such “static” protocols, we investigated three different “dynamic” approaches as shown in Table 1 by applying the selected Cell Boost supplements with the optimized feed ratio. This investigation allowed us to further improve the bioprocess performance.

The best performing approaches, constant and retrospective feed, were further investigated in fully automated bioreactors under controlled conditions. In general, constant cultivation parameters in the bioreactor slightly enhanced mAb titers compared to shake flask cultivation. The retrospective feed strategy yielded 10% higher titers than the constant strategy.

Overall, the established methodology for fed-batch development allowed us to obtain 2.5× higher mAb titers (batch mean: 1.9 g/L vs. fed-batch 4.9 g/L) in a short time and three simple steps. In addition, the product quality was investigated. Compared to the legacy fed-batch process, fed-batches that were conducted with the newly selected basal and feed media altered the distribution of charge and glycan variants. The amount of aggregated product was not altered.

**Conclusions**

The established methodology for fed-batch development is a rapid protocol to select well-performing feed supplements and optimize their ratio to the culture requirements. In three steps, mAb titers were boosted 2.5x from 1.9 g/L to 4.9 g/L. Product glycosylation and charge variants could be influenced by the newly selected basal and feed media compared to a legacy fed-batch process. The amount of aggregated product was not altered.

**Fig. 1 (abstract P-047).**
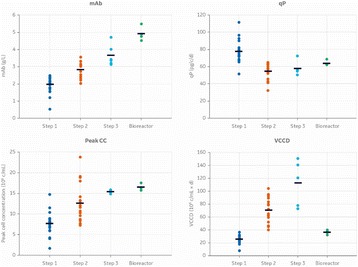
Overview of improvements made for **a** mAb titer, **b** cell-specific mAb production rates (qP), **c** peak cell concentrations, and **d** the integral over the viable cell concentration (VCCD; viable cumulative cell days) at different steps during fed-batch strategy development. Each experimental result is shown by a coloured dot. Mean values of all experiments performed at one step are indicated by a black line

**Table 1 (abstract P-047).** Strategies for static and dynamic fed-batch development

Fed-batch protocol	Fundamentals for daily feed additions
Static	Constant, daily feed additions
Dynamic – Concentration	Key substrate kept at target concentration
Dynamic – Predictive	Prediction of integrated viable cell count and cell-specific consumption rates of key substrate
Dynamic – Retrospective	Based on mean daily gains of viable cell concentration and mean cell-specific consumption rates of previous experiments

## P-048 Transcriptome analysis in high-producing CHO cell cultures: strategies to design high-performing cell culture media

### David Reinhart^1^, Andreas Castan^2^, Lukas Damjanovic^1^, Wolfgang Ernst^1^, Renate Kunert^1^

#### ^1^Vienna Institute of BioTechnology, Department of Biotechnology, University of Natural Resources and Life Sciences, Vienna, Muthgasse 11, 1190 Vienna, Austria; ^2^GE Healthcare Bio-Sciences AB, Björkgatan 30, 751 84 Uppsala, Sweden

**Background**

The present study investigates the beneficial effect of spiking HyClone™ ActiPro™ basal medium with HyClone Cell Boost™ 7a and Cell Boost 7b feed supplements on growth and productivity of a recombinant CHO cell line. To evaluate the impact of feed-spiking compared with cultivation in basal medium only, the cell line was grown in bioreactors under controlled conditions to determine cell-specific metabolic rates, nutrient consumption, and byproduct accumulation over the process time. Transcriptome analysis of the cultivated cells, using microarrays on four consecutive days to investigate differential gene expression, revealed the beneficial effect of feed-spiking compared with cells grown in basal medium.

**Materials and methods**

Model cell line was a mAb-expressing CHO DG44 (licensed from Cellca GmbH) cultivated either in ActiPro basal medium only (GE Healthcare) or in ActiPro basal medium feed-spiked with additional supplementation with 7% Cell Boost 7a and 0.7% Cell Boost 7b (GE Healthcare). Both cultures were grown in batch mode using DASGIP™ CellFerm-Pro™ stirred-tank bioreactors (Eppendorf). The beneficial effect of feed-spiking was analysed by transcriptome analysis using microarray technology (8×60 k design, Agilent).

**Results and discussion**

Both basal and feed-spiked processes lasted for seven days with viabilities above 95% until Day 6. On day seven, a sharp decline in viability indicated the end of the batch process (Fig. 1a). In feed-spiked medium, cells initially grew slower but reached almost twice as high peak cell concentrations (17.6 × 10^6^ c/mL) than in basal medium only (9.79 × 10^6^ c/mL). Remarkably, the integral of the viable cell concentration over the total process time (viable cumulative cell days [VCCD]) was similar between both process strategies (Fig. 1c). While mAb production plateaued after Day 4 in basal medium only (final titer 0.8 g/L), a continuous increase to three-fold higher final titers (2.4 g/L) was observed in feed-spiked medium (Fig. 1b). The higher titers could be attributed to generally higher cell-specific productivities (qP), which remained rather constant (~70 pg/cell/day) in feed-spiked cultures. In basal medium, the qP continuously dropped by 20% (Day 0 to 3), 50% (Day 4), and > 90% (Day 5 to 7) from 70 to 10 pg/cell/day in basal medium cultures. In average, the qP was 70% higher in feed-spiked cultures (Fig. 1d).

Transcriptome analysis of differentially expressed genes between cells grown in basal medium or feed-spiked medium were used to identify relevant GO terms that indicated a more active proliferative state for feed-spiked cultures (data not shown). The top GO terms significantly related to cell cycle and primary metabolism, cellular division, as well as nucleobase formation or regulation. Furthermore, GSEA revealed several significantly enriched set of genes related to gene transcription, DNA replication and repair, cell growth and proliferation, as well as inhibition of apoptosis in feed-spiked cultures. Thus, feed-spiking increased the proliferative activity of cultivated cells. Several of the identified genes appear as promising targets for cell line engineering, but have not yet been described in relation to high-producing recombinant cell lines and will need to be evaluated in future studies.

**Conclusions**

Feed-spiking of basal medium is a convenient and easy way to considerably increase product concentrations in a simple batch culture. Differential gene expression revealed genes that appear important for high cell-specific production rates, and this knowledge can be leveraged into cell line engineering approaches or the design of high producing CHO cell media. In the latter case, a maximized supply of nutrients enabled a self-fueling energy metabolism (data not shown) and allowed mAb expression at constantly high rates. The described results were shown to be applicable to three additional recombinant mAb-expressing cell lines including CHO DG44, CHO-S and CHO-K1 (data not shown).

**Fig. 1 (abstract P-048).**
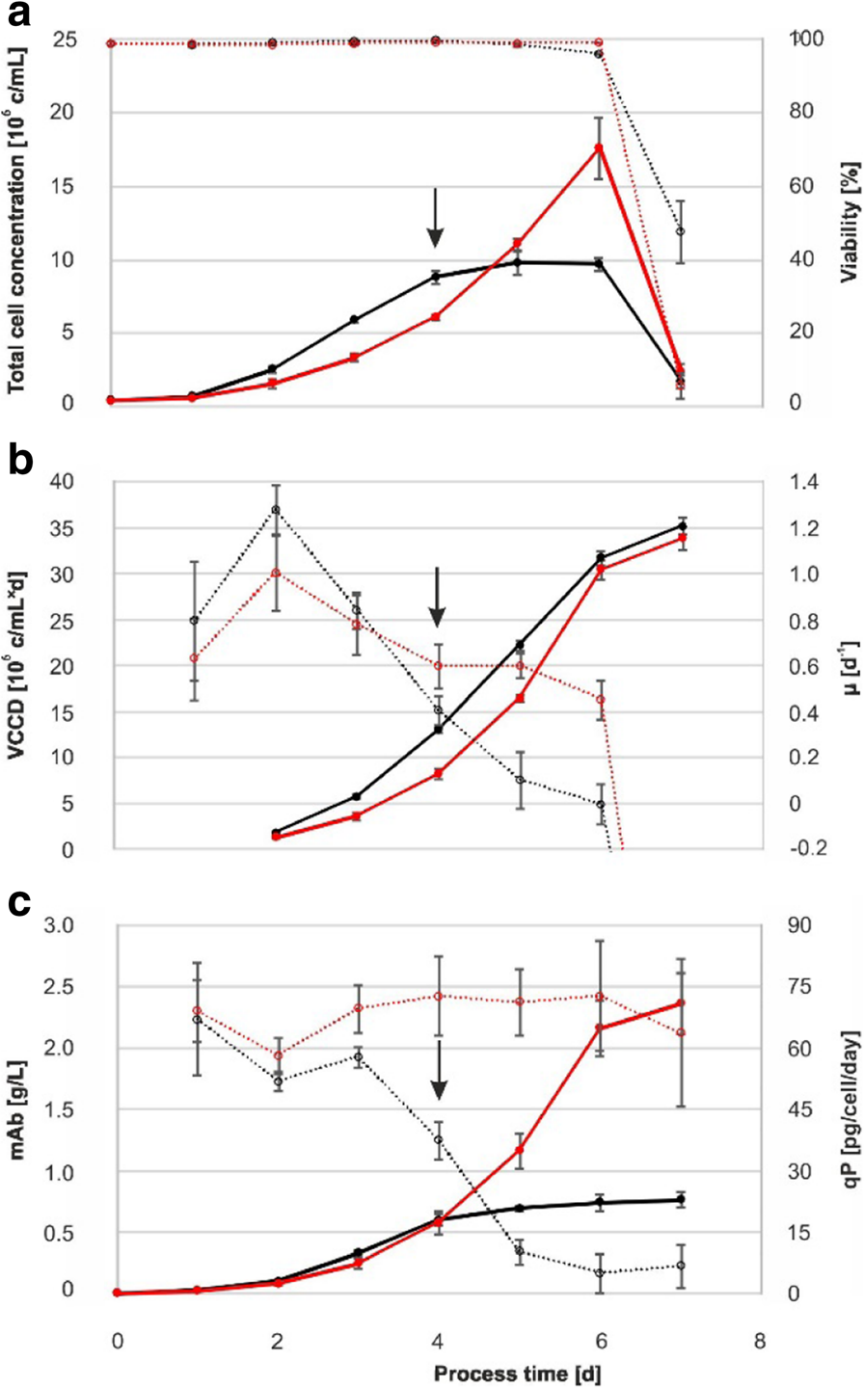
Process performance of basal medium (black) and feed-spiked (red) bioreactor batch cultures: **a** cell concentrations and viability, **b** viable cumulative cell days and specific growth rate, and **c** antibody concentrations and cell-specific productivity. Error bars indicate standard deviation from three independent experiments. The black arrows on Day 4 indicate the beginning of decreasing cell-specific productivities and lower cell-specific growth rates in basal medium cultures

## P-049 Physicochemical and functional characterization of a candidate adalimumab biosimilar TUR01

### A. Emin Atik^1^, Zeynep Yildirim Keles^1^, Yigit Erdemgil^1^, Deniz Baycin Hizal^1^, Ozge Can^2^, R. Serdar Alpan^1^

#### ^1^Biotechnology Development Center, Turgut Ilaclari, Istanbul, Turkey; ^2^Department of Medical Engineering, Acibadem University, Istanbul, Turkey

##### **Correspondence:** Deniz Baycin Hizal (dhizal@turgutilac.com.tr)

**Background**

TUR01 has been developed as a candidate biosimilar of adalimumab (Humira®). A number of originator molecules have been analyzed to determine the ranges of the critical quality attributes (CQAs). In order to obtain high similarity; a variety of upstream and downstream process development strategies were applied to assure that the CQAs fall within the ranges determined from the originator. After drug substance formulation, state-of-art analytical methods have been used to demonstrate the similarity between TUR01 and originator adalimumab.

**Materials and methods**

TUR01 was produced in a genetically engineered Chinese hamster ovary (CHO) cell lines. All mass spectrometry experiments (intact & reduced mass, peptide mapping, glycan analysis) were carried out on a Xevo G2-XS QToF mass spectrometer equipped with a lock spray ion source (Waters Corp., Milford, MA, USA). The kinetic rate constants and binding of TUR01 to TNFα was measured by Biacore T200 (GE Healthcare). The data were evaluated using the 1:1 fitting model. The charge heterogeneity (acidic and basic variants) and size heterogeneity (aggregate, monomer and fragment levels) were determined by capillary isoelectric focusing (cIEF, PA800 Plus Beckman Coulter) and size-exclusion chromatography (SEC, Waters Corp., Milford, MA, USA), respectively. Additionally, CE-SDS was used for impurity analysis. The secondary structure was obtained by FTIR experiments (Bruker Tensor 27 FTIR, Bruker Optics GmbH, Ettlingen, Germany).

**Results**

High biosimilarity must be demonstrated by physicochemical and functional characterization for approval requirements of phase I and phase III studies in terms of efficacy, safety and immunogenicity. In this study, rounds of upstream and downstream processes were run to reach the CQA limits of the originator molecule. After conducting many different development strategies, the mirror plot images of the intact deconvoluted mass were found to be identical corresponding to similar levels of glycoforms. The UV chromatogram of reversed phase ultra-performance liquid chromatography (RP-UPLC) of tryptic peptide mapping demonstrated that the primary structure of TUR01 is identical to the originator as shown in Fig. 1a. Post-translational modifications (PTMs) such as oxidation, deamidation, N-terminal pyroglutamic acid, C-terminal lysine truncation levels were also comparable for two products. The glycosylation site (HC-Asn301) was confirmed by peptide mapping analysis and 100% glycan site occupancy was proven for TUR01 and originator. The glycosylation pattern for two products were highly similar in terms of major glycans (G0F, G1F, G0F-GN and etc.). Man5 level was lower in TUR01 compared to the originator product which may not have any clinical effect on the molecule. The secondary structure was determined by ATR-FTIR spectroscopy. Absorption bands (Amide I and Amide II) were overlapped completely and amounts of *α*-helix and *β*-sheet structures were comparable. Furthermore; size-exclusion chromatography (SEC) analysis revealed that both products have the same level of purity (>99%) and aggregate (<1%) levels. The level of impurities were determined as below 4% by CE-SDS. The capillary isoelectric focusing (cIEF) experiments showed that the charge variant profiles of two products are indistinguishable and the isoelectric point of main peak is observed at 8.3 for both products. The association/dissociation rate constants and binding affinity for both TUR01 and originator were highly similar and similarity score was calculated greater than 99%, as shown in Fig. 1b.

**Conclusions**

In this study, state-of-art analytical techniques were used to assess the biosimilarity of TUR01 to the originator adalimumab. Head-to-head comparison data clearly demonstrated that TUR01 is highly similar to the originator adalimumab in terms of physicochemical and functional characteristics. Based on the analytical similarities, we believe that TUR01 will have comparable PK/PD, potency, and efficacy results to the originator adalimumab.

**Acknowledgements**

The authors thank upstream and downstream development teams of Turgut. Ilaclari, Merck Life Sciences and Covance.

**Fig. 1 (abstract P-049).**
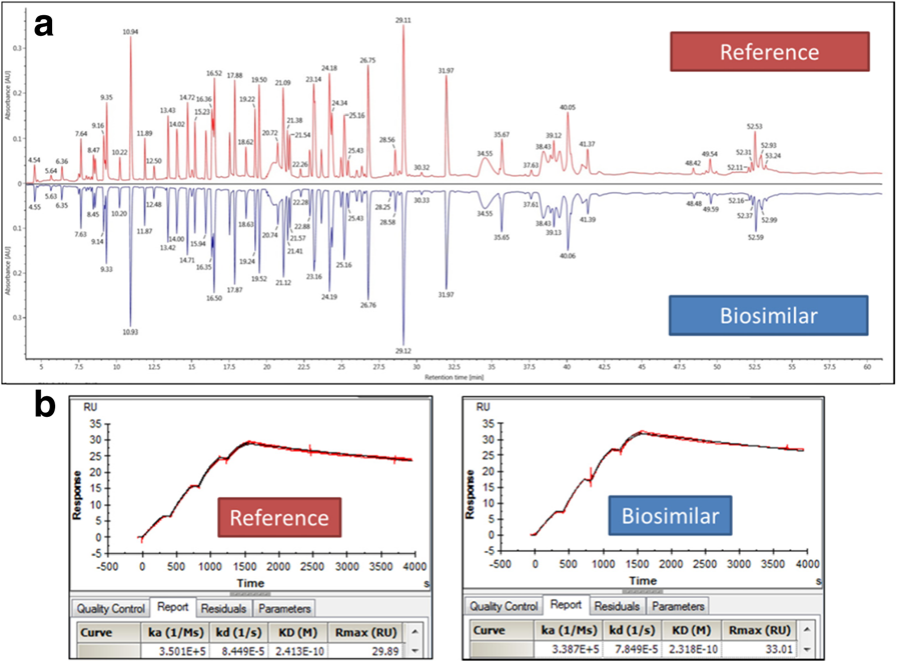
**a** UV chromatogram of reference and biosimilar peptide mapping comparison. **b** Binding kinetics sensorgram of reference and biosimilar

## P-054 Perfusion media development using cell settling in automated cell culture system

### Dustin Davis, Jeremiah Riesberg, Delia Lyons

#### MilliporeSigma, Saint Louis, MO, 63103, USA

##### **Correspondence:** Dustin Davis (dustin.davis@sial.com)

**Background**

The expanded interest in intensified continuous bioprocessing has highlighted the need to develop a small scale model for perfusion cell culture. The direction in the industry has been to increase target cell densities to ≥50x10^6^vc/mL and decrease perfusion rates to ≤3vvd. In order to increase the throughput of our perfusion media development capabilities we sought to develop a small scale model of perfusion using the ambr®15 instrument (Sartorius, Germany). We used a modified cell settling model from the previously published by Kreye et. al. to achieve the cell retention necessary to reach perfusion relevant viable cell concentrations [1]. In this work, we will show the application of this small scale model for: (1) Identification of specific productivity performance over a steady-state for tested media, (2) Identification of CSPR_min_ for a specific cell line and medium combination, and (3) Confirmation of consistent product quality profiles between the small scale model and benchtop perfusion (data not shown).

**Materials and methods**

A CHOZN® cell line producing an IgG1 was evaluated in several proprietary chemically defined media prototypes generated during the development of the catalog EXCELL® Advanced HD Perfusion medium: “Fed Batch Medium”, “Early Prototype”, “Mid Prototype”, “Intermediate Prototype” and “Late Prototype” [2]. Small scale simulation of perfusion experiments were run in ambr®15. Media exchange was performed 3 times per day in equal amounts. Agitation, gassing, and liquid handling were stopped for an optimized period of time to allow cells to settle to the bottom. Spent media was removed in an amount proportional to 1/3^rd^ daily exchange volume. Agitation, gassing, and liquid handling were resumed and fresh media was added back to the vessels. For benchtop perfusion, cells were inoculated in 3L Applikon bioreactors (Applikon, Netherlands). At a concentration of ~6.0x10^6^vc/mL, perfusion was initiated using the ATF2 (RepliGen, Massachusetts). Perfusion rate was limited at 1.2vvd during steady-state.

**Results**

Using the cell settling method described above we have been able to achieve ≥90% cell retention efficiency. All media tested in this work were able to reach and maintain the 30x10^6^ vc/mL target cell density at 1vvd (Fig. 1). Performance of each media formulation was ranked based on specific productivity (Table 1). Using “Intermediate Prototype”, minimum steady-state CSPR was determined to be 33.3pL/c/d for this cell line. N-glycan analysis of ambr®15 and bioreactor samples via intact mass spectrometry displayed only slight differences in product quality profile (data not shown).

**Conclusions**

Our work has shown a clear distinction between various prototype perfusion media and demonstrated a 50% increase in specific productivity over “Fed Batch Medium” used in perfusion. Additionally, we have shown the application to further characterize the process using this model to determine CSPR_min_ for a given medium and cell line.

**Acknowledgements**

Thank you to the bioreactor team of Irfan Hodzic, Amer Al-Lozi, and Jana Mahadevan.

**References**

1. Kreye S, Zoro B: **Webinar: ambr15 as a sedimentation-perfusion model for cultivation characteristics and product quality prediction.** 2016.

2. Riesberg J, Hodzic I, Lyons D: **“De novo” high density perfusion medium: increased productivity and reduced perfusion rates.** PO083. ESACT 2017.

**Table 1 (abstract P-054).** Average specific productivity and cell specific perfusion rates for media formulations tested

	Fed-Batch Medium	Early Prototype	Mid Prototype	Late Prototype
Average qp (pg/c/d)	19.01±0.65	22.98±1.29	26.26±1.69	31.80±1.07
Average CSPR (pL/c/d)	35.17±3.90	31.30±2.34	34.51±6.49	35.37±1.34

**Fig. 1 (abstract P-054).**
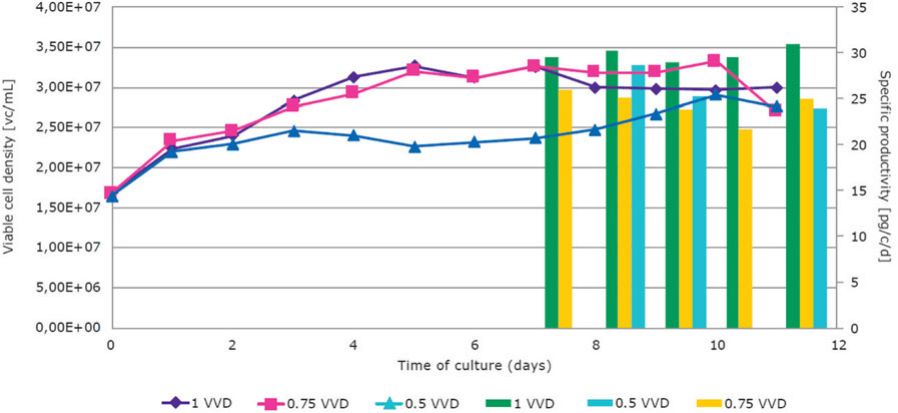
Growth and specific productivity at various perfusion rates

## P-061 An ‘Industry first’ 500L Bioreactor CHO Transient Culture: Development of large scale transient expression capabilities

### Emma Tyzack, Gary Pettman, Lekan Daramola

#### Biopharmaceutical Development, MedImmune, Cambridge, CB21 6GH, UK

##### **Correspondence:** Lekan Daramola (daramolal@medimmune.com)

**Background**

MedImmune has developed a proprietary high yielding, scalable and easy to use Chinese Hamster ovary (CHO) cell based transient expression system. The system is used routinely for early stage material supply of a variety of biologic protein formats for projects within R&D at both AstraZeneca and MedImmune.

The transient process has been successfully operated at 500L in a Sartorius Biostat Single Use Bioreactor (SUB), yielding 0.4kg of crude product from a two-week expression culture (Table 1). Successful scale up of the process to 500L creates the potential to supply transiently expressed products to support toxicology studies or even early GMP clinical supply, enabling accelerated biopharmaceutical development project timelines.

The scale up from rocking bioreactors (RBr) to SUB scale identified some scalability issues. Lower specific productivity due to increased cell growth and decreased titres were observed in the SUB (Fig. 1 iii & iv). To improve the predictability of scale up, a new process was developed and evaluated in the SUB vessels utilising a modified transfection method, which resulted in comparable expression levels and specific productivity between RBr and SUB scales.

**Materials and methods**

Two sets of expression vectors comprising heavy chain and light chain plasmids expressing a human IgG1 kappa mAb, as previously described [1,2] were used in the process optimisation study. The cell line used for transient expression and the PEI mediated transfection method has been described previously [1]. Transfected cultures were run under fed batch conditions for 14 days in 22L GE Healthcare Wave bioreactors (RBr), Hyclone SUB using 50L and 250L Hyclone bioreactor bags (Thermo Scientific).

**Results**

The transfection process was modified to address the reduced titres and higher viable cell density (VCD) seen in the SUB cultures. Shake flask cultures were used to assess the standard (A) and modified transfection processes (B and C) (Fig. 1, i & ii). Process C was identified as the process to be studied at SUB scale, offering the potential to mitigate the high viable cell densities (VCD) observed. Scaling up process C to 50L and 250L SUB resulted in cultures producing titres exceeding 1g/L with desired cell growth profiles.

**Conclusions**

Scale up of process A into SUB vessels resulted in decreased productivity compared to the RBr scale. After optimisation, the SUB process C yielded increased specific productivities and expression titres comparable to those seen at RBr scale (Table 1).

MedImmune has successfully completed the first known successful CHO transient culture at 500L scale producing > 800mg/L of mAb at harvest. Process optimisation has subsequently demonstrated reproducible titres at 50L to 250L scale exceeding 1g/L with comparable glycosylation profiles between SUB and RBr cultures across scales.

**Acknowledgements**

Steve Ruddock, Richard Lugg, Ken Lee, Rob Stadelman, Ruchika Bandekar, Karen Dickson, Faisal Uddin, Jake Warrington, Nick Myatt, Claire Pearce, Andy Smith, Chris Sellick

**References**

1. Daramola O, Stevenson J, Dean G, Hatton D, Pettman G, Holmes W, Field R. **A high-yielding CHO transient system: Co-expression of genes encoding EBNA-1 and GS enhances transient protein expression.** Biotechnol Prog. 2014; 30:132-141; DOI: 10.1002/btpr.1809.

2. Persic L, Roberts A, Wilton J, Cattaneo A, Bradbury A, Hoogenboom HR. **An integrated vector system for the eukaryotic expression of antibodies or their fragments after selection from phage display libraries.** Gene 1997; 187:9-18.

**Fig. 1 (abstract P-061).**
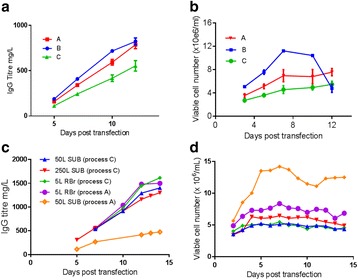
The standard transfection process (A) and modified processes (B&C) were evaluated in shake flask cultures (a & b) and SUB and RBr scale (c & d). Process B was noted to increase cell growth (b) whilst process C reduced cell growth and resulted in a slight titre drop (a). At SUB scale Process C reduced cell growth to levels comparable to the RBr culture, an effect that was scalable from 50L to 250L (d) and produced titres that were comparable to the RBr cultures (c)

**Table 1 (abstract P-061).** qP and titre benefits of the optimised SUB transfection process C when compared to process A across scales

Process Scale	Specific productivity (pg/cell/day)	Day 14 Titre mg/L
500L SUB Process A	6.54	831
250L SUB Process A	5.52	838
50L SUB Process A	3.22	440
5L RBr Process A	16.35	1499
250L SUB Process C	17.31	1300
50L SUB process C	21.70	1402
5L RBr Process C	23.64	1614

## P-064 Comprehensive analysis of the impact of trace elements in media on clone dependent process performance and product quality

### Fabian Stiefel^1^, Melanie Oesterle^1^, Frederick Rudolph^1^, Martin Pauers^2^, Jochen Schaub^1^, Jan Bechmann^1^

#### ^1^Bioprocess Development Biologicals, Boehringer Ingelheim, Germany; ^2^Pharmaceutical Development Biologicals, Boehringer Ingelheim, Germany

##### **Correspondence:** Fabian Stiefel (fabian.stiefel@boehringer-ingelheim.com)

**Background**

State-of-the-art biopharmaceutical processes are accounting concomitantly for process performance and product quality. Even though high yielding, robust processes are the cornerstones of any process development, product quality parameters such as structural integrity, charge variances and post-translational modifications are progressively becoming the focus of the developmental work. In conjunction with host cell line selection and process performance parameters, media components are crucial for the continued progress in rational modulation of product quality attributes affecting biological activity, immunogenicity, half-life or stability. Among media components, trace elements (TE) are of particular interest as they play a pivotal role in various cell metabolism pathways. Based on a comprehensive DOE approach, extensive process performance- and product quality evaluation combined with metabolic flux analysis, the impact of several trace elements on the biopharmaceutical process is assessed.

**Material and methods**

In a comprehensive I-optimal DOE approach (Fig. 1), the effect of six TE in various concentration levels and combinations in serum-free media was studied for four different CHO-K1 cells lines in an ambr® 15 setup. A scrutiny of the process performance parameters such as cellular growth, productivity, amino acids and vitamins consumptions rates for each of the conditions was performed. The process performance evaluation was accompanied by extensive product quality analysis including size and charge variants, glycosylation patterns, oxidation and methylation. Furthermore, a metabolic flux analysis was performed based on the nitrogen balance.

**Results**

Based on extensive analytical data, the obtained response surface model provides a clear insight into the impact of particular TE and their combinations on process performance and product quality. The high model quality enables discriminations between clone dependent and clone independent effects. With an elevation in titer up to 25% in the best condition of the cell lines clearly show, that even state-of-the-art media can be outperformed by trace element rebalancing. Analyzing specific rates in combination with metabolic flux analysis improves the understanding of metabolic restructuring of the cell lines under distinct TE levels and combinations. Modulation of trace elements levels had a tremendous effect on the charge heterogeneity and glycosylation structure of the different proteins. This provides a toolbox for the fine tuning and control of product quality parameters.

**Conclusion**

Taken together, the data further paves the way to the rational fine tuning of process performance and product quality attributes.

**Fig. 1 (abstract P-064).**
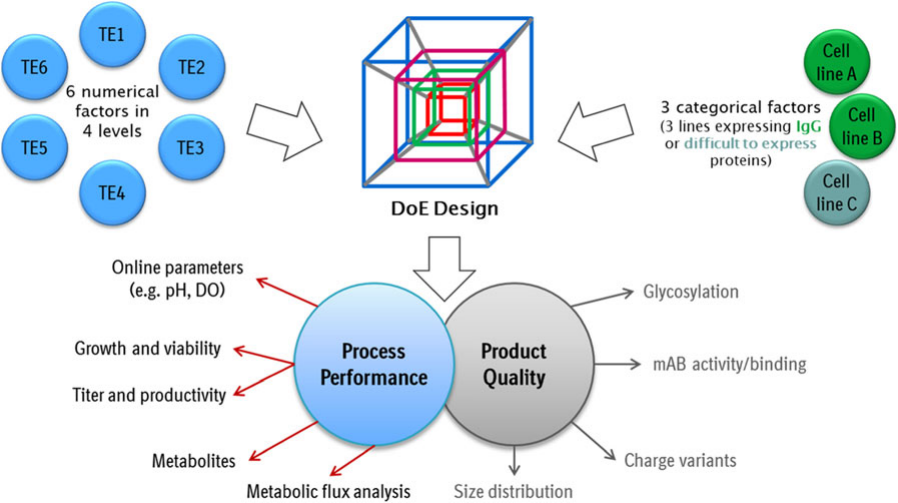
Experimental design of the DoE and analyzed parameters

## P-065 Development of an analytical approach for on-line monitoring and control of monoclonal antibodies quality

### Florian Cambay^1,2^, Olivier Henry^1^, Yves Durocher^2,3^, Gregory de Crescenzo^1^

#### ^1^Department of chemical engineering, Ecole Polytechnique de Montreal, Montreal, QC, Canada; ^2^Human Health Therapeutics Portfolio, National Research Council, Montreal, QC, Canada; ^3^Department of biochemistry, Université de Montreal, Montreal, QC, Canada

##### **Correspondence:** Gregory de Crescenzo (gregory.decrescenzo@polymtl.ca)

**Background**

Due to regulatory concerns and economic impact, ensuring product quality and consistency is now one of the main challenge faced by the biopharmaceutical industry. For monoclonal antibodies (mAb), glycosylation is one of the most important quality attributes as it impacts on mAb structure integrity, and ultimately on both clinical efficacy and safety. Many factors affect mAb glycosylation and its inherent heterogeneity, including the host cell, the culture medium, the mode of operation and the operating conditions. In this context, the capacity to monitor and control on-line the antibody glycosylation, from early- to late-stage process development, would be of salient interest to reduce the time and cost to market.

**Materials and methods**

In order to address this unmet need, we have designed an improved SPR biosensor assay to measure the kinetics of interaction between a mAb and the extracellular domain of the FcγRIIIa receptor bound at the biosensor surface [1,2].

**Results**

Of salient interest, we also demonstrated that various binding kinetic signatures, especially different dissociation kinetics could be correlated with distinct mAb glycosylation patterns and with therapeutic efficacies, as deduced from mass spectrometry and a surrogate ADCC assay, respectively. In parallel, we have also harnessed a SPR biosensor directly to a bioreactor, which permitted the at-line determination of the concentration of antibodies by hybridoma cells during a bioreactor culture.

**Conclusions**

We now plan on combining both approaches to determine on-line the glycosylation profile of the produced mAbs. Our ultimate goal is to design a unique and highly innovative bioprocess control tool that can be readily applied in an industrial bio-manufacturing setting.

**Acknowledgements**

This work was supported by the Canada Research Chair on Protein-Enhanced Biomaterials and the National Research Council Canada: Human Health Therapeutics portfolio. We thank Gilles Saint-Laurent and Christian Gervais for technical support and fruitful discussions.

**References**

1. Dorion-Thibaudeau, J., C. Raymond, E. Lattova, H. Perreault, Y. Durocher and G. De Crescenzo: **Towards the development of a surface plasmon resonance assay to evaluate the glycosylation pattern of monoclonal antibodies using the extracellular domains of CD16a and CD64.** *Journal of Immunological Methods* 2014, **408**: 24-34.

2. Dorion-Thibaudeau, J., G. St-Laurent, C. Raymond, G. De Crescenzo and Y. Durocher: **Biotinylation of the Fc gamma receptor ectodomains by mammalian cell co-transfection: application to the development of a surface plasmon resonance-based assay.** *Journal of Molecular Recognition* 2016, **29(2)**: 60-69.

## P-066 Development and assessment of a robotic highthroughput platform for antibody minipurification

### Frédéric Delouvroy, Cyrielle Calmels, Grégory Mathy, Laetitia Malphettes

#### Upstream Process Sciences, Biotech Sciences, UCB Pharma S.A., Chemin du Foriest, Braine l’Alleud, Belgium

##### **Correspondence:** Frédéric Delouvroy (frederic.delouvroy@ucb.com)

**Introduction**

Reducing timelines and costs are key factors for bio-pharmaceutical industries to accelerate process development and drug delivery to patients. Enhancing throughput of bioprocess development has become increasingly important for the screening and optimization of cell culture processes. This challenge requires high throughput tools. In a previous study [1], we showed that ambr® 15, a robotically driven mini-bioreactor system developed by TAP-Sartorius, could be advantageous to accelerate process development. The use of ambr® 15 system allows us to test a large number of experimental conditions in a single experiment. Therefore, the large amount of production samples to be characterized for Product Quality Attributes (PQA) increases as well: the bottleneck has moved from the generation of samples at the production bioreactor step to in-process analysis.

For product quality attribute analysis at lab scale, protein purification is generally carried out on >5mL columns which is incompatible with the size of ambr® 15 bioreactors. Moreover, the applied methods are relatively low throughput. The development of a high binding capacity resin (up to 70 mg/mL) [2], combined with high performing new cell lines which are able to produce up to 5 g/L of recombinant monoclonal antibodies, allow require the development of an efficient and high throughput (HTS) purification method robot. The use of robotic equipment for small scale purification purposes is a great opportunity for us to tackle this bottleneck, by enabling highthroughput sample purification at smaller scale (200μL).

**Materials and methods**

Recombinant monoclonal antibodies were produced by a genetically engineered Dihydrofolate Reductase (DHFR)-/- DG44 Chinese Hamster Ovary (CHO) cell line. Clarified cell culture fluid (CCCF) was obtained from 2 and 2K liter bioreactors after three filtration steps. Minipurifications were performed on Tecan Freedom EVO® robot with PreDictor RoboColumns® containing 200μL Mabselect SuRe® resin. Larger scale purification were executed using an AktaXpress using Hitrap column ProtA.

To assess monoclonal antibody purification at small scale, we first tested the repeatability of the minipurification, purifying the samples 8 times on the same columns and using different columns, focusing on the yield of the purification and the impact on Product Quality Attributes, especially the HMWS. Then, we compared those results to those obtained with the AktaXpress at larger scale purification, comparing the yield of the purification and the PQA of the Protein-A eluates obtained with both purification systems. Finally, we assessed the capability of the robot to perform HTS of buffer and purification conditions, evaluating three different buffers at different concentrations and pH values, and also testing different loading column capacities.

**Results and discussion**

In this study we established that the Tecan can be used as a robust platform for purification at small scale. We observed similar purification yields, intra and inter run. The analysis of the PQA1a level showed there is also very high reproducibility. And the pH of the eluate showed as well strong comparability as well. Table 1 shows the coefficient of variation (CV) of the yield, HMWS and eluate pH are low, demonstrating the good reproducibility of the purification.

The strong reproducibility obtained between the different purifications showed that the Tecan and the AktaXpress are similar in terms of purification performance and PQA (Fig. 1a, b). The Tecan is a versatile automated liquid handler allowing the screening of huge purification conditions (Fig. 1c), the possibility to purify large quantities of samples, while the samples amount is limited. The Tecan has the potential to purify more than 150 samples/day, reducing timelines and allowing us to deliver faster to the patients.

**References**

1. F. Delouvroy et al.: **ambr™ Mini-bioreactor as a High-Throughput Tool for Culture Process Development to Accelerate Transfer to Stainless Steel Manufacturing Scale: Comparability study from Process Performance to Product Quality Attributes.** ESACT 2015 Barcelona.

2. S. Ghose, et al.: **Maximizing binding capacity for protein A chromatography.** Biotechnol. Prog. (2014).

**Table 1 (abstract P-066).** Reproducibility data summary

Bioreactor	Yield (%)		PQA1.a (%)		pH of the eluate	
	Average	CV (%)	Average	CV (%)	Average	CV (%)
2000L bioreactor	76.73	0.13	1.06	0.09	3.93	0.1
2L Bio 1 (n=8)	66.68	0.02	1.03	0.13	4.07	0.01
2L Bio 2 (n=8)	65.53	1.52	1.2	0.28	3.56	1
2L Bio 3 (n=8)	67.69	0.05	1.05	0.28	4.1	0.03
2L Bio 4 (n=8)	64.3	0.06	1.09	0.23	4.07	0.04
2L Bio 5 (n=8)	66.49	0.06	0.59	0.11	3.94	0.04
2L Bio 6 (n=8)	67.99	0.03	0.63	0.05	3.94	0.02
2L Bio 7 (n=8)	61.62	0.04	0.68	0.05	3.95	0.02
2L Bio 8 (n=8)	67.18	5.35	0.65	0.06	3.93	0.03

**Fig. 1 (abstract P-066).**
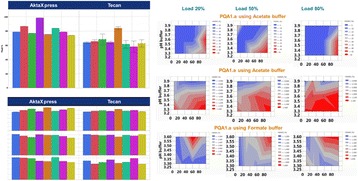
Comparison between both purification systems and the ability of the system to be used as a high throughput tools for buffers screening. **a** Purification yield (%). **b** PQA1.a (normalized). **c** Impact of the pH and buffer concentration on the PQA1.a

## P-067 Viable cell density monitoring in bioreactor with lensless imaging

### Geoffrey Esteban^1^, Martin Pisaneschi^1^, Jérémie Cubeta^2^, David Sergeant^2^

#### ^1^R&D, Iprasense, Clapiers, France; ^2^R&D, Ipratech, Mons, Belgium

**Background**

Monitoring cell density and viability of mammalian cell culture bioreactors is a necessary task that presents today a number of remaining challenges. The traditional measurement for bioreactor cell count and viability rely on using the Trypan Blue exclusion method once a day. While automatic cell counters have reduced the statistical manual error, sampling the bioreactor remains a contamination risk and is prohibiting process control as the sampled volume becomes siginficant. Lensless Imaging Technology is a new method for accurately determining cell concentration and viability without staining. This technique directly acquires the light diffraction properties of each individual cells through their holograms images without any objective, lens or focus settings. Living and dead cells have significant holographic patterns that can be distinguished and precisely counted.

**Material and method**

Lensless imaging technique directly acquires the light diffraction properties of each individual cells through their holograms images without any objective, lens or focus settings. Living and dead cells have significant holographic patterns that can be distinguished and precisely counted. We compare cell counts and viability between the reference method and our Lensless Imaging device, the Cytonote counter. Measures are performed once a day on samples from 12 bioreactors, from the inoculation to the end of the culture. We also assessed the repeatability of our method. Another Lensless Imaging prototype is setup as a measurement chamber directly connected to a perfusion bioreactor, for continuously receiving the bioreactor broth, and therefore reproducing an in situ measure.

**Results and discussion**

With a concentration range up to 40x10^6^ cells/ml (Fig. 1) and viability range at 75-100%, we obtained a correlation factor of 0.98 between the two compared methods. The large field of view allows the analyze of several thousand cells within a single image, keeping the statistical variability of the measure as low as 3%.

Our measurement chamber prototype has demonstarted its capability for continuous Viable Cell Density and viability monitoring. We are now working at designing a steam strerilizable probe, and we envision Lensless Imaging to become the future method of choice for on-line monitoring of suspension cells cultures.

**Conclusion**

Lensless Imaging Technology is capable of accurately and precisely monitoring Viable Cell Density and Viability with a combination of significant advantages starting from low sample volume use, label free detection, quick measure, simple device, to high number of cell analyzed which let us think that it is a good candidate for very small-scale bioreactor and high-throughput measures. Its high repeatability is also a key parameters in the effort to narrow batch to batch deviations. In addition we demonstrate that this technique is potentially powerful for in-line and continuous monitoring of a lab bioreactor. We envision Lensless Imaging to become the future method of choice for on-line and in-situ monitoring of suspension cells and a perfect tool for process control in fed-batch or perfusion mode in single-use bioreactors or traditional steam sterilized vessels. It can certainly become the first VCD measurement technique to work from cell line engineering, to process development, pilot scale, and up to manufacturing scale.

**Acknowledgements**

Acknowledgment to Cedric Allier from CEA Leti, Grenoble, France.

**Fig. 1 (abstract P-067).**
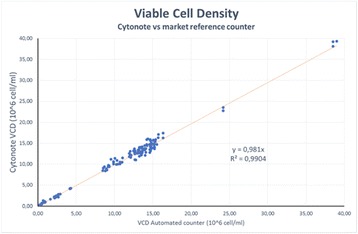
Viable cell count correlation between the lensless imaging technique and the trypan blue reference instrument

## P-068 Time-dependent product heterogeneity in mammalian cell fermentation processes

### K. Grunwald^1,2^, T. Noll^1^, H. Büntemeyer^1^

#### ^1^Cell Culture Technology, Bielefeld University, 33615 Bielefeld, Germany; ^2^Biofidus AG, 33613 Bielefeld, Germany

##### **Correspondence:** H. Büntemeyer (heino.buentemeyer@uni-bielefeld.de)

**Background**

A consistent product quality is a major goal in the production of bio-therapeutics, especially recombinant glycoproteins. Whereas it is unlikely that the polypeptide chain changes during a production process, posttranslational modifications and protein folding are sensitive to fluctuations in parameters and conditions.

Here we focus on protein glycosylation as one important indication for product quality [1]. During a batch process conditions change continuously. At the beginning, the supply situation for the cell is excellent, but the secreted material stays a long time in the culture fluid. Later during cultivation substrate provision decreases, whereas the exposition time of the protein to the culture fluid is much shorter. Altogether this leads to product heterogeneity of the secreted protein during a batch culture.

**Materials and methods**

Four different cell lines, two of human origin and two CHO clones, producing four different recombinant glycoproteins were investigated in this study. Together with their respective parental cell line the clones were cultured in three replicates in shakers. Supernatant from the cultures were harvested at four time points. The removed culture volume was replaced by culture supernatant of the identically cultured corresponding parental cell line. The product was isolated from the supernatant and the glycans were released. One part of the released glycans was labeled with 2-AB and separated by HILIC-FLD. The other part of the glycans was permethylated and analyzed by MALDI-ToF mass spectrometry (Fig. 1a). The investigated proteins were Antibody, Antithrombin III from CHO clones and α1-Antitrypsin, C1-Inhibitor from human clones.

**Results and conclusions**

The antennarity of the glycans is quite stable in all production phases. The degree of core fucosylation is very high in all products. A low fucosylation degree of antibodies may be favorable for a higher ADCC performance [2]. Some of the products showed an antennary fucosylation, which seemed to change not very much in different cultivation phases. Nevertheless, this might be an issue due to an antigenic impact in the patient.

The antennary galactosylation changes noticeable for the antibody and α1-Antitrypsin. In both cases the degree is highest in the first phase. An incomplete galactosylation leads to truncated glycans. This leads inevitable to undersialylated antennas to be seen for α1-Antitrypsin. The sialylation is the highest in the early phases and decreases during cultivation time. Sialylation of a therapeutical protein is important for the half-life in the patient. Therefore highly sialylated products are desired [3].

In further studies the consistency of the galactosylation and the sialylation will investigated for fed batch and long term continuous cultures in comparison to batch cultures. Due to the feed solution or the fresh media being present during such processes, the supply situation should be excellent for the whole cultivation time.

The differences between the MALDI-ToF and HILIC-FLD data originate from complex and unresolved chromatograms (Fig. 1b, chromatograms not shown). For that reason coupling of HILIC-FLD and MS is very much recommended.

**Acknowledgments**

We would like to thank A. Schemel and A. Ehrlich for technical assistance.

**References**

1. Parodi, Armando: **Protein Glycosylation and its Role in Protein Folding**. Annual Review of Biochemistry, 2000, 69:69-93.

2. Liu, Chalouni, Young, Junttila, Sliwkowski, Lowe: **Afucosylated antibodies increase activation of FcγRIIIa-dependent signaling components to intensify processes promoting ADCC.** Cancer Immunol Research, 2015, 3(2): 173-183.

3. Varki, Schnaar, Schauer: **Sialic Acids and other Nonulosonic Acids**. In *Essentiels of Glycobiology* [Internet] 3^rd^ edition. Edited by Varki. New York, USA:Cold Spring Harbor Laboratory Press; 2017.

**Fig. 1 (abstract P-068).**
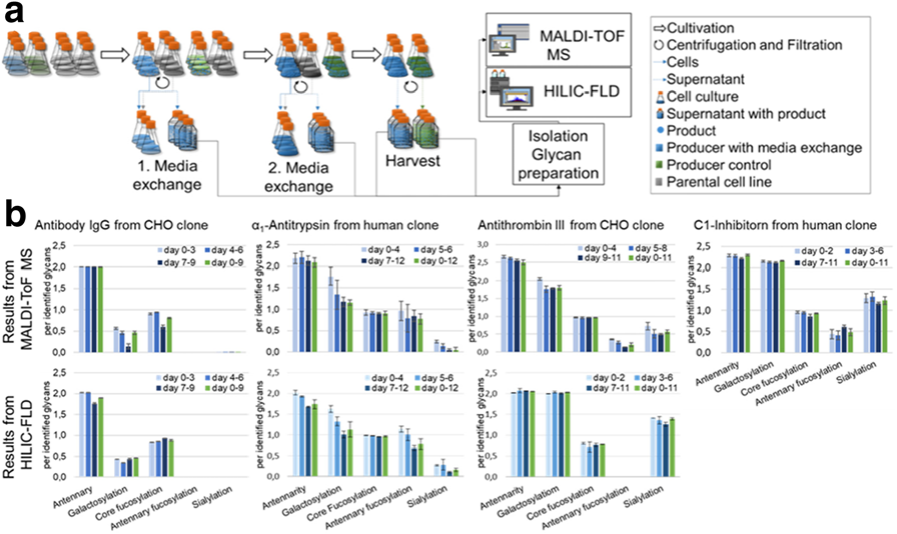
**a** Experimental process, **b** Overview of glycan appearance. The columns each represent the portion based on all identified glycans in the particular sample. The shown standard deviation results from three biological replicates

## P-071 Efficient protein production by transient gene expression using insect cells

### Hideki Yamaji^1^, Keita Mori^1^, Hirotsugu Hamada^1^, Yuki Ohmuro-Matsuyama^2^, Tomohisa Katsuda^1^

#### ^1^Department of Chemical Science and Engineering, Graduate School of Engineering, Kobe University, 1–1 Rokkodai, Nada, Kobe 657–8501, Japan; ^2^Present address: Laboratory for Chemistry and Life Science, Institute for Innovative Research, Tokyo Institute of Technology, Yokohama 226–8503, Japan

##### **Correspondence:** Hideki Yamaji (yamaji@kobe-u.ac.jp)

**Background**

Novel biologics are often selected from a large library of lead candidates in the initial stage of preclinical and clinical developments. For this selection, there is a demand for high-throughput production of recombinant proteins of high quality and in sufficient quantity. Transient gene expression offers a rapid approach to the production of numerous recombinant proteins for the initial-stage developments of biologics. Mammalian cells are major host cells for transient gene expression, but they have the disadvantages of complicated operations and high cost of culture. Insect cells are easy to handle and can be grown to a high cell density in suspension with a serum-free medium. Insect cells can also produce large amount of recombinant proteins through post-translational processing and modifications of higher eukaryotes. Hence, insect cells have been recognized as an excellent platform for the production of functional recombinant proteins [1,2]. In the present study, the production of an antibody Fab fragment through transient gene expression in lepidopteran insect cells was examined.

**Materials and methods**

The DNA fragments encoding the heavy chain (Hc) and light chain (Lc) genes of an Fab fragment of mouse anti-bovine RNaseA [3] were respectively cloned into the plasmid vector pIHAneo, which contained the *Bombyx mori* actin promoter downstream of the *B. mori* nucleopolyhedrovirus (BmNPV) IE-1 transactivator and the BmNPV HR3 enhancer for high-level expression [4]. *Trichoplusia ni* BTI-TN-5B1-4 (High Five) cells were co-transfected with the resultant plasmid vectors using linear polyethyleneimine (PEI; Mw 40,000). Before transfection, the plasmids and PEI were prepared in 150 mM NaCl, pH 7.0 and incubated at room temperature for 5 min. When the transfection efficiency was checked, a plasmid vector encoding the enhanced green fluorescent protein (EGFP) gene was also co-transfected. Transfected cells were incubated with a serum-free medium in a static or shake-flask culture. Culture supernatants were analysed by western blotting and enzyme-linked immunosorbent assay (ELISA). The numbers of green fluorescent cells and total cells in culture broth was determined using a flow cytometer.

**Results and discussion**

Western blot analysis and ELISA of culture supernatants showed that transfected High Five cells secreted the Fab fragment with antigen-binding activity. In static cultures, transfection and culture conditions, such as Hc:Lc gene ratio, a serum-free medium, DNA:PEI ratio, and DNA amount per cell, were successfully optimized by flow cytometry of EGFP expression in transfected cells and the yield of the secreted Fab fragment measured by ELISA. The effects of culture temperature and initial cell density were also examined by comparing the cell growth and the production of Fab fragments in shake-flask cultures. Under optimal conditions (medium, PSFM-J1 (Wako Pure Chemical Industries, Japan); Hc:Lc gene ratio, 3:7; DNA, 5 μg/(10^6^ cells); PEI, 10 μg/(10^6^ cells); initial cell density, 1 x 10^6^ cells/cm^3^; temperature, 24°C), the yield of more than 100 mg/L of Fab fragment was achieved in 5 days in a shake-flask culture (Fig. 1). Transfection did not significantly affect the growth of High Five cells as compared with untransfected cells. Transient gene expression using insect cells may offer a promising approach to high-throughput production of candidate proteins for the development of biologics.

**References**

1. Yamaji H: **Production of antibody in insect cells**. In *Antibody expression and production. Cell engineering. Volume 7*. Edited by Al-Rubeai M. Dordrecht, Netherlands: Springer Science + Business Media; 2011 53–76.

2. Yamaji H: **Suitability and perspectives on using recombinant insect cells for the production of virus-like particles.** *Appl Microbiol Biotechnol* 2014, **98**:1963–1970.

3. Katakura Y, Kobayashi E, Kurokawa Y, Omasa T, Fujiyama K, Suga K-I: **Cloning of cDNA and characterization of anti-RNase A monoclonal antibody 3A21**. *J Ferment Bioeng* 1996, **82**:312–314.

4. Yamaji H, Manabe T, Watakabe K, Muraoka M, Fujii I, Fukuda H: **Production of functional antibody Fab fragment by recombinant insect cells**. *Biochem Eng J* 2008, **41**:203–209.

**Fig. 1 (abstract P-071).**
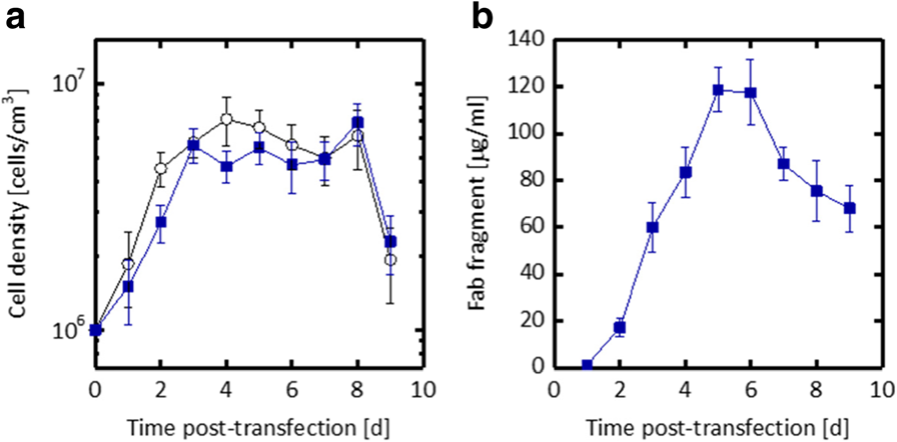
Production of Fab fragment under optimal conditions in a shake-flask culture. Cells at a density of 1 x 10^6^ cells/cm^3^ were transfected with 5 μg/(10^6^ cells) of DNA (Hc:Lc gene ratio = 3:7) using 10 μg/(10^6^ cells) of PEI in the serum-free medium PSFM-J1.Transfected cells were incubated at 24 °C. **a** Density of viable cells. Density of untransfected cells (open circles) is also shown. **b** Concentration of Fab fragments in the culture supernatant. Bars represent the means ± S.D. obtained from eight (**a**) or three (**b**) different determinations

## P-072 Implementation of different culture strategies for increasing cell density and titer in HEK293 bioreactor cultures

### Iván Martínez-Monge^1^, Pere Comas^1^, Joan Triquell^1^, Marc Camps^2^, Jordi Prat^1^, Antoni Casablancas^1^, Martí Lecina^1,3^, Jordi J Cairó^1^

#### ^1^Department of Chemical, Biological and Environmental Engineering, Autonomous University of Barcelona, Bellaterra (Cerdanyola del Vallès), 08193, Spain; ^2^ Department of Biotechnology, Farmhispania S.A., Montmeló, 08160, Spain; ^3^Bioengineering Department, IQS-Universitat Ramon Llull, Barcelona, 08017, Spain

##### **Correspondence:** Iván Martínez-Monge (ivan.martinez.monge@uab.cat)

**Background**

The increasing demand for biopharmaceuticals produced in mammalian cells has led industries to increase volumetric productivity of bioprocesses through different strategies [1,2,3]. In this context, fed-batch and perfusion cultures have attracted more interest than conventional batch processes. The efficient application of such alternative processes requires the availability of reliable on-line measuring tools for cell density and cell metabolic activity estimation [4]. The comparison of different culture strategies for HEK293 cell line producing IFN-γ are presented below: batch, fortified batch and fed-batch. In this context, a new robust feeding strategy based on the monitoring of alkali buffer addition was applied for the estimation of nutrient requirements. This method allows to increase cell density and product titer compared with the other strategies assessed.

**Materials and methods**

Three different culture strategies were carried out in 2-litre Biostat B-DCU II bioreactor. First, a reference batch and a batch using fortified medium (nutrient enriched medium) were run and assessed in terms of Viable Cell Density (VCD) and product titer, and set as initial references. Then, a fed-batch was performed applying a feeding strategy based on the nutrient requirements estimation by monitoring the alkali buffer addition used for the control of pH.

**Results**

VCD and product titer achieved for the different culture strategies assessed (batch, fortified-batch and fed-batch) are presented in table 1. In fortified batch an increase in VCD of 145% and also 350% in product titer were obtained compared with batch.

In the fed-batch culture carried out (Fig. 1), we observed that alkali buffer addition profile matched the VCD evolution trend. Thus, the monitoring of alkali buffer addition was used for estimating the nutrients requirements (i.e. the volume of feeding medium) at any time during the fed batch phase. The feeding strategy based on alkali buffer addition enabled to maintain glucose concentration set point therein a narrow range during fed-batch phase (around 20 mM). As a result, higher VCD (16.6·10^6^cells/mL) was obtained when compared with both batch references: VCD was enhanced to 241% and 39% and an increase up to 381% and 7% in product titer in respect to batch and fortified batch respectively.

The results prove that fed-batch strategy based on the alkali buffer addition is a robust on-line monitoring method that enables to optimize the feeding strategy in a fed-batch cultures.

**Conclusions**

Three different culture strategies have been tested in bioreactor with a HEK293 cell line producing IFN-γ. Results show as the higher VCD is reached, the higher product concentration is achieved. Therefore, from bioprocess development point of view, it is very interesting to implement strategies with higher VCD outcome, such as fed-batch operation mode.

In this context, a new robust method for VCD estimation in fed-batch was applied. The alkali buffer addition necessary for maintaining the pH set-point is an on-line reliable and easy measuring variable that provides information about by-products formation (mainly lactic acid). The monitoring of this variable can provide information about the cell concentration, activity and metabolism, to detect changes in culture. Besides that, a relationship between alkali buffer addition and VCD can be established since the first is strongly correlated with cell growth and metabolites consumption/formation.

The application of alkali buffer addition measure to implement an optimal feeding strategy in fed-batch permits to enhance VCD and product titer when comparing with batch strategies.

**Acknowledgements**

The authors would like to mention that this research was supported by the FI-DGR (2017) from Spanish Government and the project was led by Prof. Jordi Joan Cairó Badillo.

**References**

1. Gálvez, J., Lecina, M., Solà, C., Cairó, J. J., & Gòdia, F. (2012). **Optimization of HEK-293S cell cultures for the production of adenoviral vectors in bioreactors using on-line OUR measurements.** *Journal of biotechnology*,*157*(1), 214-222.

2. Román, R., Miret, J., Scalia, F., Casablancas, A., Lecina, M., & Cairó, J. J. (2016). **Enhancing heterologous protein expression and secretion in HEK293 cells by means of combination of CMV promoter and IFNα2 signal peptide.** *Journal of biotechnology*, *239*, 57-60.

3. Liste-Calleja, L., Lecina; M. & Cairó, J.J. (2014). **HEK293 cell culture media study towards bioprocess optimization: animal derived component free and animal derived component containing platforms*.*** *Journal of Bioscience and Bioengineering,* 117(4), 471-477.

4. Casablancas, A., Gámez, X., Lecina, M., Solà, C., Cairó, J. J., & Gòdia, F. (2013). **Comparison of control strategies for fed‐batch culture of hybridoma cells based on on‐line monitoring of oxygen uptake rate, optical cell density and glucose concentration.** *Journal of Chemical Technology and Biotechnology*,*88*(9), 1680-1689.

**Table 1 (abstract P-072).** Summary of the VCD and product titer for the three culture strategies performed with HEK293 in bioreactor

	VCD_max_ (x10^6^ cells/mL)	Product titer fold increase (relative to Batch)
Batch	4,88	1,00
Fortified-Batch	12,00	3,50
Fed-Batch	16,64	3,81

**Fig. 1 (abstract P-072).**
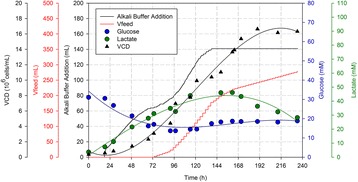
VCD, alkali buffer addition, feed volume, glucose and lactate concentration in HEK293 fed-batch performed based alkali buffer addition

## P-078 A novel approach to high throughput screening for perfusion

### Jean-Marc Bielser^1,2^, Jakub Domaradzki^1^, Jonathan Souquet^1^, Massimo Morbidelli^2^, Hervé Broly^1^

#### ^1^Biotech Process Sciences, Merck KGaA, Corsier-sur-Vevey, Switzerland; ^2^Institute of Chemical and Bioengineering, ETH Zürich, Zürich, Switzerland

##### **Correspondence:** Jean-Marc Bielser (jean-marc.bielser@merckgroup.com)

**Background**

Perfusion systems for suspended mammalian cells raise growing interest in the biomanufacturing industry. Continuous manufacturing is growing in the field and is encouraged by health authorities [1, 2, 3]. This work addresses scale down limitations inherent to continuous media exchange and cell retention by using a semi-continuous system. Data was generated with a set of different clones that were previously studied in fed-batch mode [4].

**Materials and methods**

4 CHO-K1 cell lines expressing the same monoclonal antibody (mAb) and issued from the same transfection were used as models. 3.5L bioreactors (Sartorius) were used for fed-batch and perfusion production runs. The perfusion bioreactors were run using an alternating tangential flow filtration device (Repligen, XCell™ ATF 2 System). The cell biomass was controlled by removing cells through a bleed line and was controlled using a biocapacitance probe (Hamilton, Incyte). The perfusion rate (D) was fixed to one vessel volume a day (vvd^-1^). The semi-continuous runs were made in 50 mL shake tube (TPP, TubeSpin® Bioreactor 50). Once a day, the tubes were centrifuged (5 min, 200 g), the supernatant removed (to mimic a perfusion rate of 1 vvd^-1^), replaced with fresh media and cells were re-suspended.

**Results**

The clone’s growth potential were preserved across the systems (Fig. 1). Clone #3 always reached the highest viable cell density (VCD), followed by clone #1. Clone #2 and #4 showed similar growth characteristics. It is interesting to note that in the perfusion bioreactor different patterns in terms of VCD were observed although the cell biomass signals were similar for all 4 runs. This reflects the fact that the capacitance measures the biomass and not the absolute cell count [5].

To estimate the minimum cell specific perfusion rate (CSPR_min_) in the semi-continuous experiment, the perfusion rate was divided by the maximum viable cell density (VCD_max_). This value was compared to the CSPR obtained during the 4^th^ set-point (SP4) of the perfusion runs. As expected, the bleed fraction decreased when the capacitance set-point was increased and went down to 5% or less of the total perfusion rate (data not shown). Since the bleed removes the excess biomass, it is an indication of how close to a limitation the system is. Therefore, the CSPR calculated at SP4 was considered as the minimum CSPR. The CSPR_min_ obtained in both systems were very close (Table 1). The semi-continuous system can therefore be used to identify the CSPR_min_ before running a continuous bioreactor, it therefore facilitates the decision making early in the development (to define the target cell density for a defined perfusion rate).

The specific productivity (Q_p_) of the 4 clones was quantified at the maximum VCD (semi-continuous) or at SP4 (perfusion). Absolute values are not representative since the cell environment is so different in both systems. Nevertheless, a relative ranking proved to be indicative of the respective performances (Table 1). The maximum cell growth in fed-batch, semi-continuous and perfusion were also compared, their ranking was always preserved. Both indications can be used to assess a performance ranking for different clones.

**Conclusions**

The performance of 4 clones was studied in 3 different cultivation systems: fed-batch/perfusion bioreactors and semi-continuous shake tube. The semi-continuous system was able to precisely predict the CSPR_min_, an important process parameter for perfusion. Specific productivity and maximum cell density ranking was preserved across the systems, therefore the scale down experiment can be used to assess a performance ranking for perfusion clone screening.

**Acknowledgments**

The authors want to thank the Biotech Process Sciences team at Merck in Corsier-sur-Vevey for their support and also the members of the Morbidelli group at ETH Zürich for their input and collaboration.

**References**

1. S. S. Farid, B. Thompson, and A. Davidson: **Continuous bioprocessing: The real thing this time?** *mAbs* 2014, **6**:1357-1361.

2. K. B. Konstantinov, C. L. Cooney: **White paper on continuous bioprocessing.** *J. Pharm. Sci.* 2015 *J. Pharm. Sci.*, **104**:813-820.

3. S. Chatterjee: **FDA Perspective on Continuous Manufacturing.** *IFPAC Annu. Meet.* 2012.

4. Rouiller Y, Bielser J-M, Brühlmann D, Jordan M, Broly H, Stettler M: **Screening and Assessment of Performance and Molecule Quality Attributes of Industrial Cell Lines across Different Fed-Batch Systems.** *Biotechnol. Prog.* 2015, **32**:160-170.

5. C. F. Opel, J. Li, A. Amanullah: **Quantitative modeling of viable cell density, cell size, intracellular conductivity, and membrane capacitance in batch and fed-batch CHO processes using dielectric spectroscopy.** *Biotechnol. Prog*. 2010, **26**:1187–1199.

**Fig. 1 (abstract P-078).**
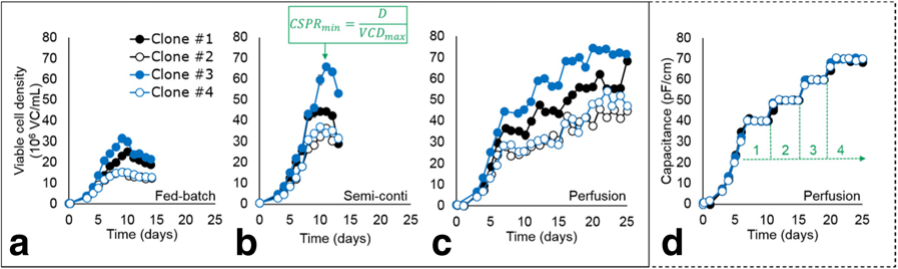
Viable cell density of the 4 clones in **a** fed-batch bioreactors **b** semi-continuous shake tubes **c** perfusion bioreactors and **d** on-line capacitance signal used to increment the biomass set-point (SP) progressively (SP 1 to 4)

**Table 1 (abstract P-078).** Summary of CSPR, Q_p_ (relative to clone #1) and maximum growth in all the systems. Clone #1 was by far the best candidate for perfusion by reaching a productivity of about 1 g/L/day at SP4. The three other clones, despite their different growth and specific productivities all performed a productivity close to 0.5 g/L/day on SP4

	Semi-conti	Perfusion	Semi-conti	Perfusion	Ranking	Fed-batch	Semi-conti	Perfusion
Clone	CSPR_min_	CSPR_SP4_	Q_p_	Q_p-SP4_	-	VCD_max_	VCD_max_	VCD_SP4_
-	pL/cell/day	pL/cell/day	-	-	-	10^6^·VC/mL	10^6^·VC/mL	10^6^·VC/mL
**#1**	**23.5**	**21.7 (±0.8)**	**1**	**1**	**2**	**25.3**	**44.5**	**55.4 (±0.1)**
#2	27.5	28.3 (±0.3)	0.82	0.67	4	15.4	36.4	42.7 (±1.1)
#3	15.2	17.3 (±0.3)	0.28	0.31	1	31.6	70.0	72.7 (±0.7)
#4	27.1	24.8 (±1.3)	0.67	0.59	3	15.5	36.9	44.7 (±5.5)

## P-079 Modulating antibody galactosylation through cell culture medium for improved function and product quality

### Jenny Y. Bang, James-Kevin Y. Tan, Catherine Nguyen, David T. Ho, David E. Ho, Jessie H.-T. Ni

#### Research & Development Department, Irvine Scientific, Santa Ana, CA, 92705, USA

##### **Correspondence:** Jenny Y. Bang (jbang@irvinesci.com)

**Background**

The production of therapeutic antibodies (Abs) requires high titers and excellent product quality to ensure efficient manufacturing and potent drug efficacy. Glycosylation, or the attachment of sugars to organic molecules, is a critical quality aspect that can significantly alter Ab binding, function, and therapeutic effect [1]. Galactose is a key sugar of interest due to its significant impact on Ab function and the ability to control galactosylation through cell culture medium. Herein, Irvine Scientific assessed the ability of media components to modulate galactose levels on a model therapeutic Ab. Various media compositions were able to modulate galactosylation levels without compromising cell growth and Ab titers. In addition, an *in vitro* assay was utilized to evaluate the functional ability of Abs to bind and activate complement-dependent cytotoxicity (CDC). Differences in galactosylation significantly altered the Abs’ ability to induce cell cytotoxicity. Furthermore, design of experiment analysis determined the optimal ratio of supplements to maximize galactosylation. This “Optimized Supplement” was verified and evaluated against other suppliers’ galactosylation supplements in terms of growth, titer, glycan analysis, and Ab function. The Optimized Supplement outperformed all other suppliers‘ supplements and resulted in the best overall cell growth, titer, galactosylation, and Ab function.

**Materials and methods**

Fed-batch cultures of Chinese hamster ovary cells expressing an IgG1 Ab against CD20 were grown in BalanCD® CHO Growth A and were fed with BalanCD® CHO Feed 4 on days 3-7 of the cultures. Viable cell density and cell viability were assessed by a Beckman Coulter Vi-Cell XR, Ab titer was assessed by a Pall FortéBio QK^e^, and glycan analysis was assessed by a PerkinElmer LabChip GXII. For the functional CDC assay, Abs were incubated with Daudi B lymphoblast cells and normal human complement serum. Cell cytotoxicity was assessed with a Promega CytoTox-Glo kit.

**Results**

Various supplements were evaluated in fed-batch cultures and resulted in 15-45% Ab galactosylation without compromising cell growth and Ab titers. Design of experiment analysis determined an optimal composition, deemed “Optimized Supplement,” which was evaluated against a panel of galactose-modulating supplements from other suppliers. The Optimized Supplement resulted in a similar viable cell density (VCD) and cell viability compared to the fed-batch culture Control which had no supplements (Fig. 1a). Supplements from Supplier 1 resulted in similar to half the VCD of the Control while supplements from Supplier 2 resulted in very low VCD and percent viability. All of the supplements except those from Supplier 2 resulted in Ab titers similar to the Control (Fig. 1b). Due to the poor growth and subsequently low titer from Supplier 2’s supplement, Supplier 2 was not further evaluated. The glycan profiles were analyzed and are presented in (Fig. 1c). All the evaluated supplements were able to raise galactosylation; however, only the Optimized Supplement and the 2X Supplier 1 Supplement resulted in over 40% galactosylation. The function of the Abs was further evaluated in a CDC assay (Fig. 1d). Abs from the Optimized Supplement were more effective than the Control Abs and had a significantly lower half-maximal effective concentration (EC_50_, 1.19 μg/mL) than the Control (1.71 μg/mL). Abs from the 2X Supplier 1 Supplement had a similar EC_50_ to the Control which may be due to the higher Man5% of the Abs.

**Conclusions**

An Optimized Supplement was produced through fed-batch evaluation and design of experiment analysis. The Optimized Supplement outperformed all other supplenments from other suppliers and resulted in the best overall cell growth, glycan profile, and functional Ab activity (Table 1).

**Reference**

1. Hossler P, Khattak SF, Li ZJ: **Optimal and consistent protein glycosylation in mammcalian cell culture.** *Glycobiology* 2009, **19**: 936-949.

**Fig. 1 (abstract P-079).**
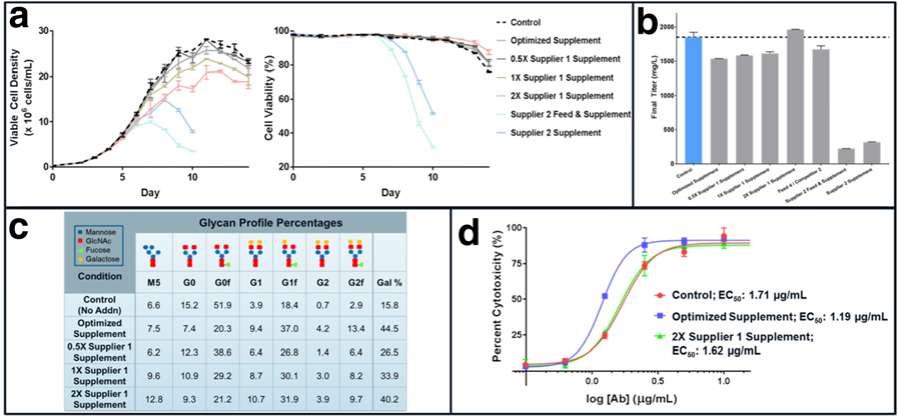
Comparison of galactose-modulating supplements. Irvine Scientific’s Optimized Supplement and various supplements from other suppliers were evaluated in their ability to affect **a** cell growth, **b** titer, **c** glycan profile, and **d** Ab function. The Optimized Supplement was able to maintain cell growth, sustain high titers, increase Ab galactosylation, and improve Ab function

**Table 1 (abstract P-079).** Summary of key parameters from top-performing supplements

	Max Viable Cell Density (x 10^6^ cells/mL)	Estimated Cumulative Cell Density (x 10^6^ cells/mL)	Final Titer (mg/L)	Percent Galactosylation (%)	Man 5 (%)	CDC EC_50_ (μg/mL)
Control, No Supplements	28.2	198	1850	15.8	6.6	1.71
Optimized Supplement	25.7	185	1538	44.5	7.5	1.19
2X Supplier 1 Supplement	21.1	148	1964	40.2	12.8	1.62

## P-082 Development of a high-throughput platform to support cell culture media and feed screening

### Jente Lu, Luke Wang, Crystal Lee, Julie Gardin, Yvette Tang

#### Cell Culture Process Development, BioMarin Pharmaceutical Inc., Novato, CA 94949, USA

##### **Correspondence:** Jente Lu (jente.lu@bmrn.com)

**Background**

Industry practice for mammalian cell culture media and feed development typically employs a high-throughput screening (HTS) platform along with large sets of experiments [1]. Modern HTS systems often include robotic liquid handlers to replace labor intensive steps. To align with advancements in the field, a semi-automated HTS platform was developed to facilitate in-house media and feed development for early stage biologics projects.

**Materials and methods**

Selecting appropriate instruments and integrating them into a seamless system are the keys to a HTS platform. The developed HTS platform uses 24 deep well plates (DWPs) for culture vessels, the liquid handler of the Advance Microscale BioReactor (AMBR15) for media/feed formulation preparation in an aseptic environment, NyONE cell imager for viability and cell growth analysis, TECAN Freedom EVO‘s liquid handler for activity assay sample preparation, and Cedex BioHT for high-throughput metabolite analysis. 24 DWPs offer comparable cell growth to shake flasks and compatible layout to AMBR15, which makes the 24 DWP an ideal candidate for the platform. In addition, the user friendly Design of Experiments (DoEs) interface and liquid handler function of the AMBR15 expediates the formulation preparation of varying DoE conditions [2]. A macro program was written and developed in Excel to enable the easy import of DoEs design from major statistics software packages, such as JMP and Simca, into AMBR15.

**Results**

Performance qualification of each component were performed prior to implementing the HTS platform. Comparable cell growth profile and productivity were achived between shake flasks and 24 DWPs (Fig. 1a), indicating compatable cell culture environment for the cells. Cell counts using NyONE gave identical cell growth ranking as the traditional count from Vi-Cell XR (Fig. 1b). Freedom EVO’s liquid handler was optimized to produce comparable activity results to manual operation while expediting the sample preparation with improved consistency (Fig. 1c). Finally, implementing the liquid handler function of AMBR15 to support media and feed formulation significantly reduced the labor for each experiment. Summary of the capability comparison between the HTS platform and the traditional method are listed in Table 1.

A case study of a complex feed screening with definitive screening design was completed using the semi-automatic HTS platform. This experiment, containing more than 60 feed formulations in duplicates, was handled by one operator and delivered a 40% improvement in productivity within a 4 week period (Data not shown). In addition, implementing the HTS platform for this study also resulted into ~80% reduction in labor while improving the traceability of formulation preparation.

**Conclusion**

A semi-automated HTS platform was developed to support media and feeds screening and development for early stage biologics projects. The platform utilizes 24 DWPs, NyONE cell imager, AMBR15, Freedom EVO liquid handler system, and BioHT metabolic analyzer to accelerate the screening process. This screening platform not only improves process throughput, operational precision, and traceability of formulation preparation, but also reduces the labor for the media and feed formulation preparation.

**Acknowledgements**

Authors would like to thank Dr. Benjamin Youn in Manufacturing Science and Technology (MSAT) at BioMarin for his help on coding the Excel Macro program for AMBR15, and Dr. Donald L. Traul from TAP Biosystems (now part of the Sartorius Stedim Biotech Group) for his assistance on AMBR15 operations.

**References**

1. Radu C, Adrar HS, Alamir A, Hatherley I, Trinh T, Djaballah H: Journal of laboratory automation. **Designs and Concept-Reliance of a Fully Automated High Content Screening Platform.** 2012;17(5):359-369.

2. Delouvroy F, Siriez G, Tran A-V, Mukankurayija L, Kochanowski N, Malphettes L: BMC Proceedings. **ambr**^**TM**^ **Mini-bioreactor as a high-throughput tool for culture process development to accelerate transfer to stainless steel manufacturing scale: comparability study from process performance to product quality attributes.** 2015;9(Suppl 9):P78.

**Fig. 1 (abstract P-082).**
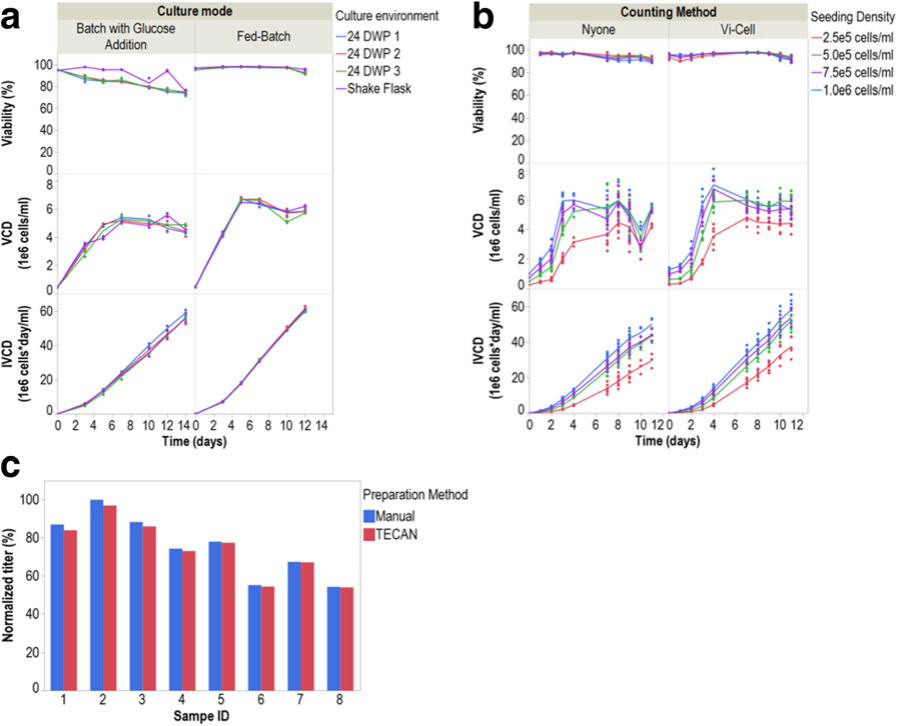
Performance qualification of each component. **a** Shake Flask and 24DWPs exhibit comparable growth profile in both culture modes. **b** NyONE and Vi-Cell both has sufficient resolution to distinguish growth profiles with 4 distinct inoculation cell density. **c** Assay performed using samples prepared by TECAN Freedom EVO produces comparable titer ranking (sample ID #2 as the highest sample and sample ID #6 and #8 as the lowest two samples) as samples prepared by manual operation with faster turnaround

**Table 1 (abstract P-082).** Comparison of throughput between the traditional and high-throughput screening method

	Condition per Incubator ^a^	Formulation Preparation	Cell Counting ^b^	Titer Assay
HTS platform	384 conditions	Fully automated	0.625 min/ sample	15 min/ 24 samples
Traditional Method	~100 conditions	Manual preparation	3 mins/ sample	1 hour/24 samples

^a^Based on max capacity of the incubator. ^b^ HTS platform perform cell counts at 30 mins/plate with 28 samples on one plate

## P-083 “De novo” high density perfusion medium: increased productivity and reduced perfusion rates

### Jeremiah Riesberg, Irfan Hodzic, Delia Lyons

#### MilliporeSigma, St. Louis, MO, 63109, USA

##### **Correspondence:** Jeremiah Riesberg (jeremiah.riesberg@sial.com)

**Background**

A perfusion medium requires high concentrations of specific nutrients while balancing other components to support intensified perfusion processes. Using a combination of design of experiment (DOE), multivariate analysis (MVA), and spent media analysis, we developed a catalog “de novo” perfusion medium by working with multiple CHO cell lines and proteins. The optimization of the medium in bioreactors using alternating tangential flow (ATF) cell retention devices reduced the minimum CSPR from over 80pL/cell/d to under 35pL/cell/d for most cell lines while increasing specific productivity during 30 day steady states with stable growth rates, viability, volumetric productivity and product quality.

**Materials and methods**

High Throughput Screening (HTS) was performed with seven cell lines, while four were used in bioreactors: CHO-S, DG44, and two CHOZN® GS lines, each producing different monoclonal antibodies and include a fusion protein. For HTS experiments, cells were inoculated at 2.0x10^6^vc/mL with a 30 mL working volume in 50 mL TPP® tubes and cultured for 7 days in a Multitron shaken at 200rpm, 37°C, 80% RH, and 5% CO_2_.

For benchtop perfusion, cells were inoculated at 0.4-2.0x10^6^vc/mL in 3L Applikon bioreactors (Applikon, Netherlands) with a 2L working volume. Bioreactors were operated at 350 rpm, 37°C, 40% DO, and a pH of 6.9 or 7.1±0.05 depending on the cell line. Oxygen was supplied through an L- sparger or microsparger as needed, and Excell® antifoam (MilliporeSigma, Germany) was added at a maximum rate of 0.25% v/v to control foam. At a cell concentration of ~6.0x10^6^vc/mL, perfusion was initiated using the ATF2 (RepliGen, Massachusetts), with a bleed set to maintain cell concentrations at 50 or 80*10^6^vc/mL.

**Results**

Two “de novo” prototype media were developed using DOE and MVA in HTS with TPPs and an ambr®15 [1] and one was chosen for further development after comparing to a basal medium enriched with feed in bioreactors. Eleven components were identified as significant effectors of critical parameters for perfusion processes across evaluated cell lines. DOE central composite experiments were run and component concentrations were optimized in the selected prototype.

In parallel, amino acid specific consumption rates were calculated from bioreactor spent media samples and used to adjust the concentration of amino acids to target a reduced CSPR. Increasing specific amino acids concentrations resulted in a significant reduction of the minimum CSPR across all tested cell lines - for example the CSPR of CHO-S was reduced from 60 to 39pL/cell/day (Table 1). However, even at the lower CSPR, spent media analysis revealed excess concentration of some amino acids, so specific accumulating amino acids were reduced and components were streamlined for the final medium: EXCELL® Advanced HD Perfusion Medium.

Using this medium, a CHO-S and a CHOZN® GS cell line producing a fusion protein were cultured at a CSPR of less than 40pL/cell/day with a VCD of 50*10^6^vc/mL. Metabolic profile, productivity, and product quality were constant over the 30 day steady state. The CHOZN® GS cell line was also tested at 80*10^6^vc/mL with a CSPR of 33pL/cell/day (Fig. 1).

**Conclusions**

We have developed a catalog perfusion medium from first principles, ensuring broadness of application by using seven cell lines in scaled-down systems and four in perfusion bioreactors. The final catalog medium showed significant improvements in productivity across all cell lines, with reduced CSPRs when compared to enriched fed-batch medium or initial prototypes (Table 1).

**Acknowledgments**

Thanks to the bioreactor team of Dustin Davis, Amer Al-Lozi, and Jana Mahadevan.

**Reference**

1. Davis D, Riesberg J, Lyons D: **Perfusion media development using cell settling in automated cell culture system.** PO054. ESACT 2017.

**Table 1 (abstract P-083).** CSPR and q_p_ improvements from early prototype to EXCELL® Advanced HD Perfusion Medium with three cell lines. Final catalog perfusion medium requires greatly reduced CSPR with higher specific productivity

	CSPR		q_p_		VCD
	Prototype	Final	Prototype	Final	Max
CHOS IgG	74	39	8.9	10.5	50*10^6^
CHOZN® GS Fusion	72	39	8.7	9.6	50*10^6^
		33		8.7	80*10^6^
CHOZN® GS IgG	80	59	20	28.5	50*10^6^

**Fig. 1 (abstract P-083).**
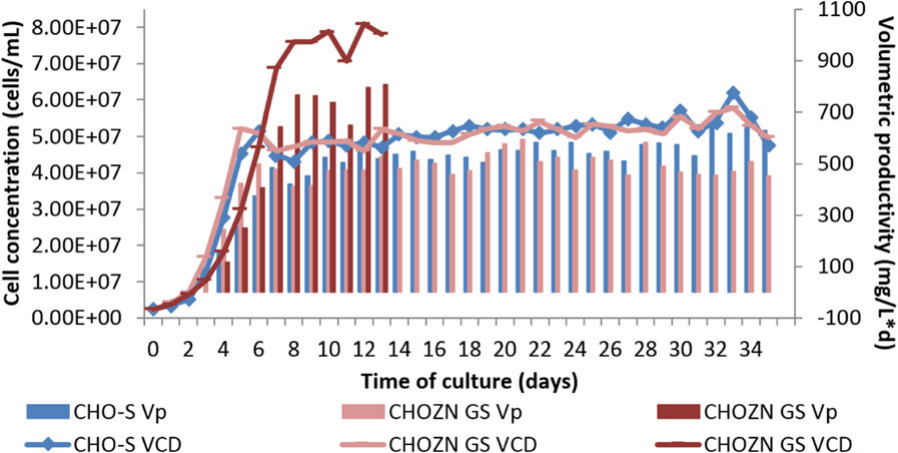
At a target cell density of 50*10^6^vc/mL, volumetric productivity was stable for a 30 day steady state with EXCELL® Advanced HD Perfusion Medium. Shorter steady states were tested at 80*10^6^vc/mL

## P-087 Improving mammalian cell culture process development by model-assisted design of experiments

### Johannes Möller, Tobias Steinmetz, Marius Braakmann, Ralf Pörtner

#### Institute of Bioprocess and Biosystems Engineering, Hamburg University of Technology, Hamburg, D-21073, Germany

##### **Correspondence:** Ralf Pörtner (poertner@tuhh.de)

**Background**

There is a rising demand for accelerated process development, increased efficiency and economics for biopharmaceutical production processes. Furthermore, increased process understanding have evolved from the Process Analytical Tool initiative (PAT) and the Quality by Design (QbD) methodology. In contrast to one-factor-at-a-time methods, statistical Design of Experiment (DoE) methods are widely used to develop biopharmaceutical processes. Even if high-throughput systems can handle these numbers of experiments in parallel, the heuristic restriction of boundaries and the high number of factors results in stepwise iterations with multiple runs. Therefore, the combination of model-based simulations with DoE methods (mDoE) for the development of sophisticated cell culture processes is a novel tool for process development [1]. It is used to reduce the number of experiments during DoE and the time needed for the development of more knowledge-based cell culture processes. This concept was applied to the optimization of the initial glutamine and glucose concentrations of a CHO batch process.

**Material and methods**

A mechanistic model was adapted and modified from [2] and used to describe the dynamics of cell metabolism and antibody production of an IL-8 antibody producing CHO cell line (see abbreviation of Fig. 1 for cultivation details). Experiments were simulated and compared to a fully experimental DoE. As can be seen from Table 1, user defined constraints were chosen to get a stable and reproducible process with the aim of maximizing the cell density but decreased lactate and ammonia production.

**Results**

At first, the experimental space was estimated by simulating the responses for broad concentration ranges and calculating the multiple response desirability function (Fig. 1a). This results in a small area (turquoise) suggested as experimental space. Experiments were planned within these boundaries and responses were either simulated (Fig. 1b, 4 cultivations for fitting the model) or compared with the purely experimental responses (Fig. 1c, 16 cultivations). Optimal concentrations for glutamine and glucose with respect to the constraints are in the lower right corner and similar for both methods (red frame, Fig. 1).

**Conclusion**

Compared with the fully experimental design, mDoE results in a reduction of 75 % in the number of experiments (4 experiments for modelling vs. 16 experiments in experimental DoE). The method is intended to optimize cultivation strategies for mammalian cell lines and evaluated these before experiments have to be performed in laboratory scale. This results in a significant time and cost reduction during process development and process establishment. The strategy is especially intended for the use in multi-single-use-devices to speed up process development.

**Acknowledgements**

We kindly acknowledge the Cell Culture Technology group (Bielefeld University, Prof. Dr. Thomas Noll) for providing the cell line used in this work and the Federal Ministry of Education and Research for funding (Grant no. 031B0305).

**References**

1. Möller J and Pörtner R: **Model-based Design of Process Strategies for Cell Culture Bioprocesses: State of the Art and New Perspectives**. New Insights into Cell Culture Technology, 1^st^ edition, InTech. doi:10.5772/67600, 2017 157-172.

2. Kern S, Platas Barradas O, Pörtner R, Frahm B: **Model-based strategy for cell culture seed train layout verified at lab scale**. Cytotechnology, 2016 Aug; 68(4):1019-32. doi: 10.1007/s10616-015-9858-9. Epub 2015 Mar 21.

**Table 1 (abstract P-087).** Constraints for medium optimization

Concentration	Constraints
Viable cell density	> 10^7^ cells/ml
Antibody	maximize
Lactate	<30 mM
Ammonium	minimize

**Fig. 1 (abstract P-087).**
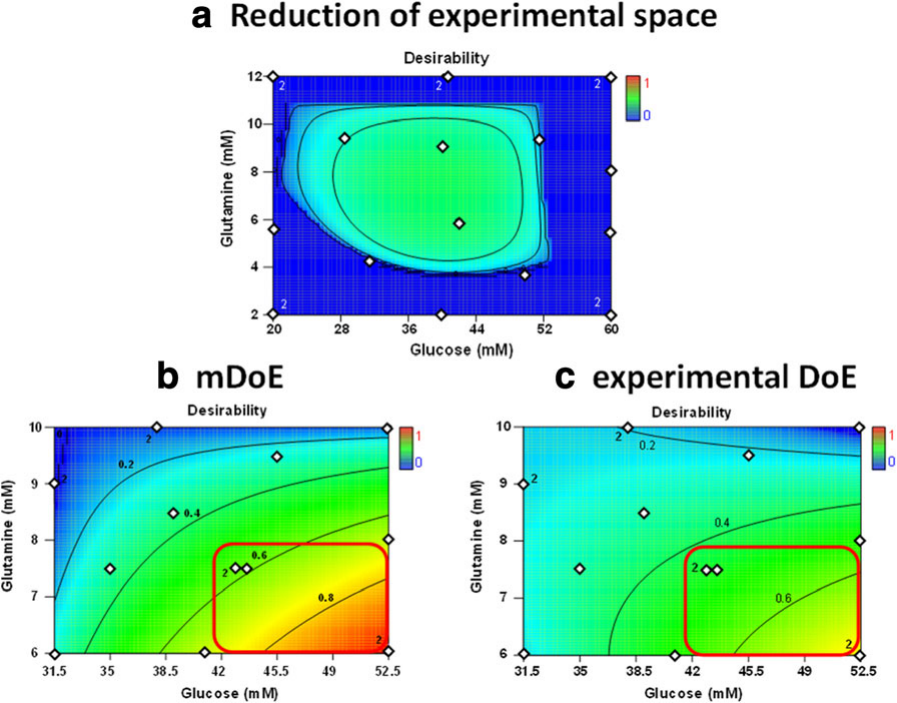
Reduction of experimental space and comparison of simulated and experimental medium optimization, experimental design was done in Design Expert 9 (I-optimal Design, 5 lack-of-fit, 5 replicate points)); **b** model was fitted to mean of 4 parallel shaking flask cultivations (80 ml working volume, MATLAB 2015a), **c** cultivation was done in 16 parallel shaking flasks (40 mL working volume each), Incubator: Kuhner LT-XC (37°C, 5% CO2, 85% humidity, 12,5 mm shaking diameter, 200 rpm), Medium: TC-42, Xell AG

## P-092 Understanding of decreased sialylation of Fc-fusion protein in hyperosmotic recombinant Chinese Hamster Ovary cell culture: *N*-glycosylation gene expression and *N*-linked glycan antennary profile

### Jong Hyun Lee^1^, Yeong Ran Jeong^1^, Yeon-Gu Kim^2,3^, Gyun Min Lee^1^

#### ^1^Biological Sciences, KAIST, Daejeon, 305-701, Republic of Korea; ^2^Biotechnology Process Engineering Center, KRIBB, Cheongju, 363-883, Republic of Korea; ^3^Department of Bioprocess Engineering, UST, Daejeon, 305-350, Republic of Korea

##### **Correspondence:** Gyun Min Lee (gyunminlee@kaist.ac.kr)

**Background**

For the large-scale production of therapeutic glycoproteins, fed-batch culture has been widely used for its operational simplicity and high titer. However, repeated feeding of medium concentrates and/or addition of a base to maintain optimal pH during fed-batch culture lead to increase in osmolality. The hyperosmolality affects glycosylation in a protein-specific manner. However, the mechanism behind such osmolality-dependent variations in glycosylation in recombinant Chinese hamster ovary (rCHO) cells remains unclear.

**Materials and methods**

In this study, to better understand the effect of hyperosmolality on the glycosylation of a protein produced from rCHO cells, we investigated 52 *N*-glycosylation-related gene expression and *N*-linked glycan structure in Fc-fusion protein-producing rCHO cells exposed to hyperosmotic conditions. Furthermore, to validate the effect of hyperosmolality on protein glycosylation, we performed hyperosmotic culture supplemented with betaine, an osmoprotectant, and then analyzed the *N*-linked glycan structure and mRNA levels of *N*-glycan branching/antennary genes.

**Results**

After three days of hyperosmotic culture, nine genes (*ugp*, *slc35a3*, *slc35d2*, *gcs1*, *manea*, *mgat2*, *mgat5b*, *b4galt3*, and *b4galt4*) were differentially expressed over 1.5-fold of the control, and all these genes were down-regulated. *N*-linked glycan analysis by anion exchange and hydrophilic interaction HPLC showed that the proportion of highly sialylated (di-, tri-, tetra-) and tetra-antennary *N*-linked glycans was significantly decreased upon hyperosmotic culture. Addition of betaine, an osmoprotectant, to the hyperosmotic culture significantly increased the proportion of highly sialylated and tetra-antennary *N*-linked glycans (P ≤ 0.05), while it increased the expression of the *N*-glycan branching/antennary genes (*mgat2* and *mgat4b*). Thus, decreased expression of the genes with roles in the *N*-glycan biosynthesis pathway correlated with reduced sialic acid content of Fc-fusion protein caused by hyperosmolar conditions.

**Conclusions**

Taken together, the results obtained in this study provide a better understanding of the detrimental effects of hyperosmolality on *N*-glycosylation, especially sialylation, in rCHO cells. The identified genes, particularly *mgat2* and *mgat4b*, are potential targets for engineering in CHO cells to overcome the impact of hyperosmolality on glycoprotein sialylation.

**Acknowledgements**

This research was supported in part by a grant from the Bio & Medical Technology Development Program of the NRF funded by the Korean government (2013M3A9B6075931, 2016R1A2B4014133).

**References**

1. Pfizenmaier J, Junghans L, Teleki A, Takors R: **Hyperosmotic stimulus study discloses benefits in ATP supply and reveals miRNA/mRNA targets to improve recombinant protein production of CHO cells.** *Biotechnol J* 2016, 11(8):1037-1047.

2. Schmelzer AE, Miller WM: **Effects of osmoprotectant compounds on NCAM polysialylation under hyperosmotic stress and elevated pCO**_**2**_**.** *Biotechnol Bioeng* 2002, 77(4):359-368.

## P-093 Disruptive cost-effective antibody manufacturing platform based on cutting-edge purification process

### V. Medvedev, M. Duyck, T. Albano, J. Castillo

#### Univercells SA, Gosselies, Belgium

##### **Correspondence:** V. Medvedev (v.medvedev@univercells.com)

**Background**

Demand for high-quality monoclonal antibodies is growing exponentially, calling for new production capacities. Overcoming current limitations of conventional manufacturing strategies, namely the high capital investment and production cost, can only be achieved through innovative process designs based on the latest technologies.

This study presents a process design combining batch-fed technology with continuous multi-column capture. An advanced cell culture clarification method was introduced to simplify downstream operations and increase overall cost-effectiveness of the process, for an optimized production of recombinant proteins.

**Materials and methods**

This study was performed with CHO cells expressing a monoclonal antibody targeted against the Coronoavirus responsible for the Middle East Respiratory Syndrome (MERS), developed by *Organic Vaccines*^*TM*^ and the NIH, kindly provided to Univercells.

Upstream process:

- Fed-batch, 12 days culture at 10L scale with CD-CHO chemically defined media and feeds.

Harvest treatment:

- Precipitation of impurities in the production bioreactor using organic compounds (<1% v/v) and flocculation by electropositive organics (<0.1% w/v).
- Acidic pH and physiological conductivity.

Harvest clarification:

- Depth filtration with coarse and fine grade depth filters followed by sterilising-grade filtration.
- Primary depth filters: D0HC, D0SP, Clarisolve 20 (Merck), Sartoclear Dynamics (Sartorius).
- Secondary depth filters: X0HC (Merck), DL20 (Sartorius).
- Sterilizing grade filters: Express SHC (Merck), Sartopore 2 XLG (Sartorius).

Capture of antibody:

- Affinity capture: CaptivA PriMab (Repligen), Mab Capture A Select (Thermofisher), Amsphere A3 (JSR), Toyopearl AF-rProteinA HC-650F (Tosoh).
- Cation exchange capture: Gigacap S-650M (Tosoh).

**Results**

**Upstream processing and harvest treatment:** Culture reached 0.5 g/L (8x10^6^ cells/mL; 90% viability), harvest treatment was found to be very effective in terms of impurities clearance.

**Capture:** Capture strategies were evaluated from the point of view of simplification of downstream operations, with HCP impurities content monitored as a key performance indicator.

- Protein A affinity chromatography: Advanced harvest clarification enabled major improvements in affinity capture, in terms of eluate purity and reduction of host cell impurities (<35 ppm in all conditions tested). (Fig. 1).
- Cation exchange chromatography: CEx allows higher capacities (>100 g/L) than protein A, whilst being more affordable (from 2- to 6-fold cheaper). Low residual HCP (<500 ppm) was observed with all CEx resins tested. Without harvest treatment and clarification preceding the capture studies (either affinity or CEx), results showed a lower binding capacity of the resin, a higher content of HCP in the eluate (up to 2000 ppm), a higher content of HMW species in the elution fraction (up to 3-fold higher) and a significant turbidity of the neutralized eluate.
- Continuous multicolumn chromatography: Further options to increase cost efficiency include using a continuous multicolumn setup (Table 1). Two models were assessed based on two different static binding capacities (SBC), demonstrating that 4 to 6 columns of 100ml were able to process a 200L production in less than 24h.

**Conclusion**

This method provides a great opportunity for designing simplified and low footprint Mabs DSP processes, while maintaining similar or achieving superior quality profile compared to standard approaches:

- Harvests treatments followed by depth filtration proved to be a cost-efficient way to obtain pretreated feed and minimize the burden on downstream operations.
- Protein A resins exhibited advantages of extracting key contaminants during harvest treatment, while CAEX confirmed to be a competitive capture strategy.
- Switching from batch to continuous multicolumn mode allowed to process a complete batch in less than 24 hours, requiring lower media and resins volumes. Followed by a single polishing step, such process set-up strongly supports the reduction of operations required to deliver a high-quality product.

**Acknowledgements**

Organic Vaccines^TM^ and the NIH, who kindly provided to Univercells.

**Fig. 1 (abstract P-093).**
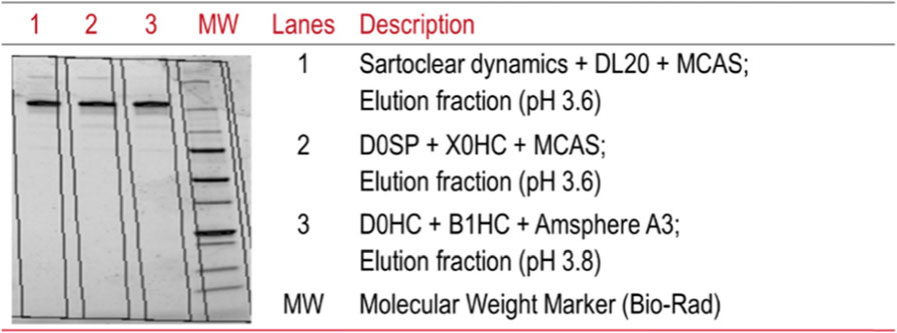
Capture on protein A

**Table 1 (abstract P-093).** Capture results with multicolumn chromatography

200L Feed at 4g/L	4 columns		6 columns	
	72 mg/mL SBC	30 mg/mL SBC	72 mg/mL SBC	30 mg/mL SBC
	5x5=98.2mL	5x5=98.2mL	5x5=98.2mL	5x5=98.2mL
Residence time (min)	0.5	1.2	0.5	0.5
Feed velocity (cm/h)	600	250	600	600
Max velocity (cm/h)	891	891	600	614
Max flow (ml/min)	291.6	291,6	196.4	196.4
Productivity (SS)	96.2	40.1	64.2	50.2
Number of cycles	32.0	76.0	21.6	50.9
**Process Time (h)**	**21.6**	**51.2**	**21.8**	**27.4**

## P-096 Analyses of product quality of complex polymeric IgM produced by CHO cells

### Julia Hennicke, David Reinhart, Renate Kunert

#### University of Natural Resources and Life Sciences, Vienna, 1190, Austria

##### **Correspondence:** Julia Hennicke (julia.hennicke@boku.ac.at)

**Background**

Immunoglobulin M (IgM) antibodies are secreted by B cells as the first defense against invading pathogens during primary immune response. Some IgM antibodies already gained the orphan drug status, which shows their unique capability in therapy of rare diseases. Potential fields for applications are discovered with increasing knowledge about these molecules. It seems that the most active forms are pentameric and hexameric IgMs. Unfortunately, recombinant production of IgMs is rather difficult as secretion and correct polymer formation results in low expression yields and mixtures of polymers.

**Materials and methods**

We established stable producing Chinese hamster ovary (CHO) DG44 cell lines to analyze cellular and extracellular factors that influence quantity and quality of the produced recombinant polymeric IgM in future studies [1]. One quality parameter is polymer distribution, which can be measured directly in cell culture supernatant using densitometric analyses [2]. Additionally, we developed a very efficient single-step-affinity purification strategy using the POROS CaptureSelect IgM Affinity Matrix to analyze pure IgMs. For more precise measurements of the IgM isoform distribution we separated the purified polymers by high performance liquid size exclusion chromatography (SEC HPLC).

**Results**

Our CHO DG44 cell lines grow to peak cell concentration of 4.5x10^6^ cells/mL in Erlenmeyer flasks and 4.0x10^6^ cells/mL in bioreactors. Similar productivity of approximately 50 mg/L was observed for cells cultivated in both cultivation vessels in a non-optimized batch culture using chemically defined media. Analysing how cultivation conditions affect the fraction of polymers may offer clues about the assembly of polymers and the challenges of IgM production. We quantified polymeric distribution of IgM directly in the supernatant using a densitometric method [2]. Cultivated under standard conditions (37°C, pH 7) IgM012 is produced as 90% pentamers, whereas IgM012_GL only consists of approximately 80% pentamers. The purified IgM012_GL was analysed with SEC-HPLC and contained 81 % pentamer and 19 % dimer, which is comparable to the results achieved with densitometry.

The purification of the IgM antibodies was quite challenging as the manufacturer recommend acidic elution, which led to aggregation and inefficient elution of our model IgMs. Therefore, we screened for different elution buffers that prevent denaturation and aggregation. By combining high salt concentrations with moderate pH reduction we optimized elution conditions to 88-99% IgM recovery, which corresponded to a five to six fold improvement compared to the manufacturers’ conditions. SDS-PAGE analysis and SEC-HPLC showed that our elution strategy resulted in a very pure product after a single chromatographic step. The purification strategy was verified with the IgM103, IgM104 and IgM617.

**Conclusions**

Our model IgMs were produced in a ratio of approximately 4:1 pentameric to dimeric IgM, measured concordantly with both analytical methods. Process development on IgM purification using the POROS Capture Select human IgM affinity matrix enabled the recovery of highly pure fractions. Through optimization, by combining mild pH and high salt concentrations, the relatively low elution yields were increased by a factor of 5-6. Applying densitometry and SEC-HPLC we will investigate how culture conditions influence polymer formation in future.

**Acknowledgements**

We thank Polymun Scientific Immunbiologische Forschung GmbH for providing the antibodies IgM103, IgM104 and IgM617 as a kind gift. This work was supported by the PhD program BioToP (Biomolecular Technology of Proteins) funded by the Austrian Science Fund (FWF Project W1224).

**References**

1. Chromikova, Mader, Steinfellner, Kunert: **Evaluating the bottlenecks of recombinant IgM production** **in mammalian cells.** *Cytotechnology* 2015, **67**:334-356.

2. Vorauer-Uhl, Wallner, Lhota, Katinger, Kunert: **IgM characterization directly performed in crude culture supernatants by a new simple electrophoretic method.** *J immunol Methods* 2010, **359**:21-27.

## P-097 Development of a high-throughput scale down model for a high cell density PER.C6®-based adenovirus perfusion production process

### Julia Meijer, Iris van Hoorn, Matthijs van Duijvenboden, Jeroen de Lozanne, Perrine Rouel, Bas Diepenbroek

#### Vaccine Process and Analytical Development, Janssen Vaccine and Prevention B.V., Leiden, Netherlands

##### **Correspondence:** Julia Meijer

**Background**

Currently, no small scale (<0.5L) cell culture system is commercially available for high cell density perfusion cultivations to use in high throughput screening studies. To increase throughput for process characterization activities at Janssen Vaccines and Prevention, a shaker flask-based scale down model was developed. Though, the control possibilities of shaker flask cultures are technically very limited and different compared to a bioreactor controlled process. In addition, the sensitivity of the shaker flask model should allow the detection of the effects of process parameters on critical quality attributes (CQAs) of the vaccine produced at large scale.

**Material and methods**

Iterative experiments were performed in shake flasks to evaluate the influence of cultivation parameters such as shaking speed, working volume, CO2% in the incubator and daily base additions on cultivation parameters (as cell growth, pH and DO). In addition, a medium exchange was tested to mimic the perfusion mode used in the bioreactor process. The PreSens shake flask reader was implemented to allow for pH and DO monitoring. The conditions for which the performance as reflected in specific virus titer showed the best fit were selected. At these conditions, a series of parallel shaker flask infections were conducted to demonstrate statistical equivalence of performance parameter and CQAs (as cell specific IU titer and VP/IU ratio) between the production scale and reduced scale processes and thus to qualify the shake flask as a scale-down model.

**Results**

A daily medium exchange by centrifugation was implemented and cultivation parameters for shake flasks were identified. Based on performance parameter (cell specific VP titer) and the CQAs of the vaccine (cell specific IU titer and VP/IU ratio), equivalence between the production-scale and scale down systems was confirmed. The scale down model data fall into the 95% prediction intervals calculated on manufacturing data whereas scale down model data from batch mode experiments (using non optimized cultivation conditions) do not.

**Conclusions**

The shaker flask as a scale down model for the 10L bioreactor perfusion process was qualified. This model is a tool to screen a subset of process parameters at a higher throughput, thereby reducing process characterization timelines.

**Fig. 1 (abstract P-097).**
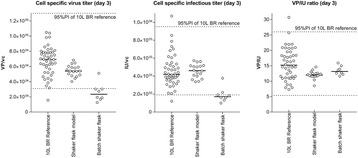
10L bioreactor manufacturing data (reference), optimized scale down model data and scale down model data from batch mode experiments. The 95% prediction intervals calculated on 10L bioreactor manufacturing data are the acceptance criteria

## P-099 Evaluation of signal peptides for enhanced production levels in CHO DG44 cells

### Nico Erb, Juliana Schubert, Caroline Hauser, Christoph Zehe

#### Sartorius Stedim Cellca GmbH, 88471 Laupheim, Baden-Württemberg, Germany

##### **Correspondence:** Nico Erb (nico.erb@sartorius-stedim.com)

**Background**

Until today, the market for therapeutic proteins, especially monoclonal antibodies, is gaining more and more importance in the pharmaceutical field. To meet the increasing demand for these products, the industry made tremendous efforts to generate highly efficient production systems. One of the pharmaceutical industry’s research focuses is the improvement of the secretion process in eukaryotic cells. In mammalian cells, the efficiency of protein transportation strongly depends on the translocation of a nascent protein into the ER, which is mostly conducted by the signal peptide (SP) coupled to the N-terminus. Through the interchangeability of signal peptides between products and even species, a large variety can be used to enhance protein expression in already existing production systems

**Materials and methods**

At first the influence of four different natural SPs (SP(7), (8), (9) and (10)) was compared on the secreted amount of an IgG4 model antibody (product A) in fed batches using a CHO DG44 host cell line. In the second part, one promising SP-candidate showing improved secretion (SP(9)) was identified and the influence of this SP on four additional antibody products, which varied in their expressability from good to mid/bad, was investigated. In both approaches, the standard SP was implemented for comparative reasons.

**Results**

In the first approach, four signal peptides SP(7), SP(8), SP(9) and SP(10) were screened for their potential to improve the product secretion of CHO DG44 cells expressing a model antibody (product A).

The results revealed a 2.4-fold increase in average final fed-batch antibody titer of SP(9) when compared to the standard SP approach (standard SP = 0.44 g/L; SP(9) = 1.50 g/L).

In the second approach, the enhancing capacity of SP(9) on secretion of four other IgG products (named product B to E, Table 1) was further evaluated. An improved performance was observed for all products when comparing SP(9) and the standard SP in a fed batch process (Fig. 1). With an increase in average final fed-batch titers ranging from 28 to 354 % and up to 290 % in cell-specific productivities.

Taken together, with a positive influence on the final concentrations of all tested products, the results obtained with SP(9) contribute to the optimization of Sartorius Stedim Cellca’s standard cell line development process.

**Conclusions**

- Signal peptide SP(9) was identified as a promising candidate with an average 2.4-fold titer increase during screening of four signal peptides.
- SP(9) was able to improve production titers up to 354 % compared to standard SP.
- SP(9) was able to improve cell-specific productivities up to 290 % compared to standard SP.
- Future usage of SP(9) contributes to the further optimization of Sartorius Stedim Cellca’s standard cell line development process.

**Fig. 1 (abstract P-099).**
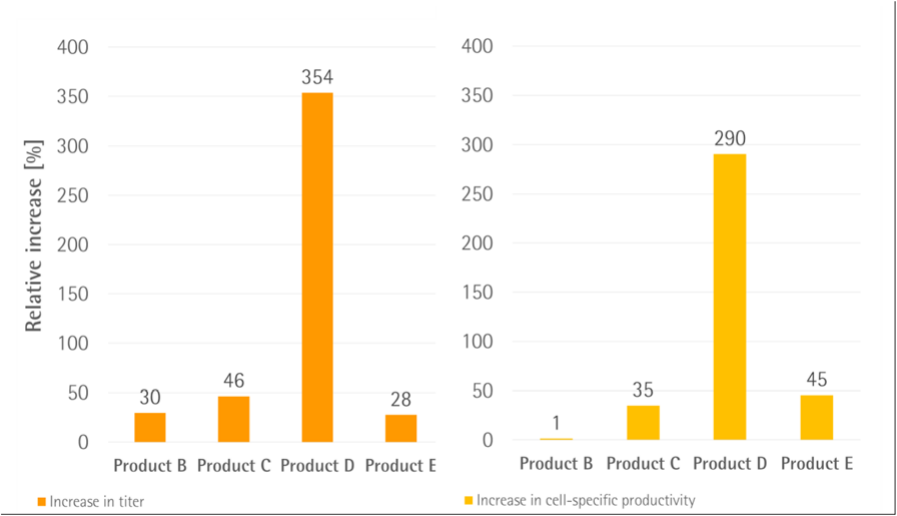
Percentaged increase in final fed batch titers and cell-specific productivity of CHO DG44 Mini Pools with SP(9) expressing product B to E. in comparison to the standard SP

**Table 1 (abstract P-099).** Information of antibody products implemented into signal peptide screening and evaluation (+ = good expressability, - = mid to bad expressability, 0 = bad expressability)

Product	Type of product	Expressability
A	IgG4 antibody	0/-
B	IgG4 antibody	+
C	IgG4 antibody	+
D	IgG2 antibody	-
E	IgG4 antibody	0/-

## P-103 New platform for the integrated analysis of bioreactor online and offline data

### Lukasz Gricman^1^, Milan Ganguly^2^, Amanda Fitzgerald^3^, Hans Peter Fischer^1^, Christoph Freiberg^1^

#### ^1^Genedata AG, Basel, Switzerland; ^2^Genedata Ltd., London, UK; ^3^Genedata Inc., Boston, MA, USA

##### **Correspondence:** Lukasz Gricman (bioprocess@genedata.com); Christoph Freiberg (bioprocess@genedata.com)

**Background**

More and more experiments are used to assess bioreactor suitability and stability of clones, to evaluate media composition and other process parameters, and to start upscaling campaigns. This has resulted in a major bottleneck due to the increase in data capturing, processing, aggregation, visualization, and statistical analysis. In addition, the association of the data with the experimental context (e.g., fermentation protocols, media recipes, bioreactor control parameters) is not easily accomplished in high throughput. The data generated in the process must not only be analyzed, but also managed and stored to enable easy tracking and relating to historical records. Furthermore, the processes are often developed by global teams interacting in complex enterprise IT ecosystems. Therefore, new and high performing systems for data capture, processing, and analysis need to be integrated in order to enable storage and correlation of experimental context information and various types of time course analytics data.

**Materials and methods**

We have developed Genedata Bioprocess™, a new enterprise platform for bioprocess development. The platform enables automatic capture and visualization of all online and offline data (e.g., pH, O2, metabolic data), auto-calculations and aggregations (e.g., IVCD, qP, consumption rates) and multi-parametric assessment of any type of time-series bioreactor data in the context of experimental protocol data (e.g., process parameters, feeds). Genedata Bioprocess comes with dedicated interfaces for integrating with relevant laboratory instruments, control systems, statistical analysis software packages and custom enterprise solutions. It enables the modeling and tracking of complex nonlinear workflows and supports decision making in bioprocess development. The data can be analyzed in the context of upstream process development, and also be correlated to other unit operations. Automation support assists the ever increasing throughput of bioprocess development operations, and the analysis of experimental data and process parameters across unit operations or even different projects. This overall integration enhances process development workflows.

**Results and conclusions**

Highlighted use cases describe the selection of the best producer clones (Fig. 1a), the identification of optimized media feeding strategies (Fig. 1b), and the comparison of clone performance across different fermentation scales (Fig. 1c). A special focus is on the analysis of data from micro- and bench-top bioreactors (such as the ambr15™ and DasGip™ systems) operated in parallel. These bioreactors allow for increased throughput of clone selection and process optimization studies, which in turn leads to an increase in data generation. Genedata Bioprocess supports integration with such systems and enables a comparison of data regardless of the instrument provider or scale. Automated bioreactor data analysis allows development groups to take advantage of even richer datasets and, as data management is built-in to the system, the data can be easily tracked and associated to historical records. Another focus is on cross-reactor scale comparisons. Data coming from different bioreactor scales can be easily imported into the platform and analyzed to establish the best conditions for upscaling. Genedata Bioprocess enables the correlation of process parameters (e.g., fermentation protocols, media recipes, bioreactor control parameters), with key performance indicators of the processes (e.g., Titer, qP) and the product quality attributes (e.g., aggregation, glycosylation profiles). Finally, bioreactor time course data can be tracked together with clone analytics and product quality parameters, which makes the platform uniquely able to support end-to-end biopharma development.

**Acknowledgements**

Allison Kurz and Gian Andrea Signorell, Genedata AG, Basel, Switzerland

**Fig. 1 (abstract P-103).**
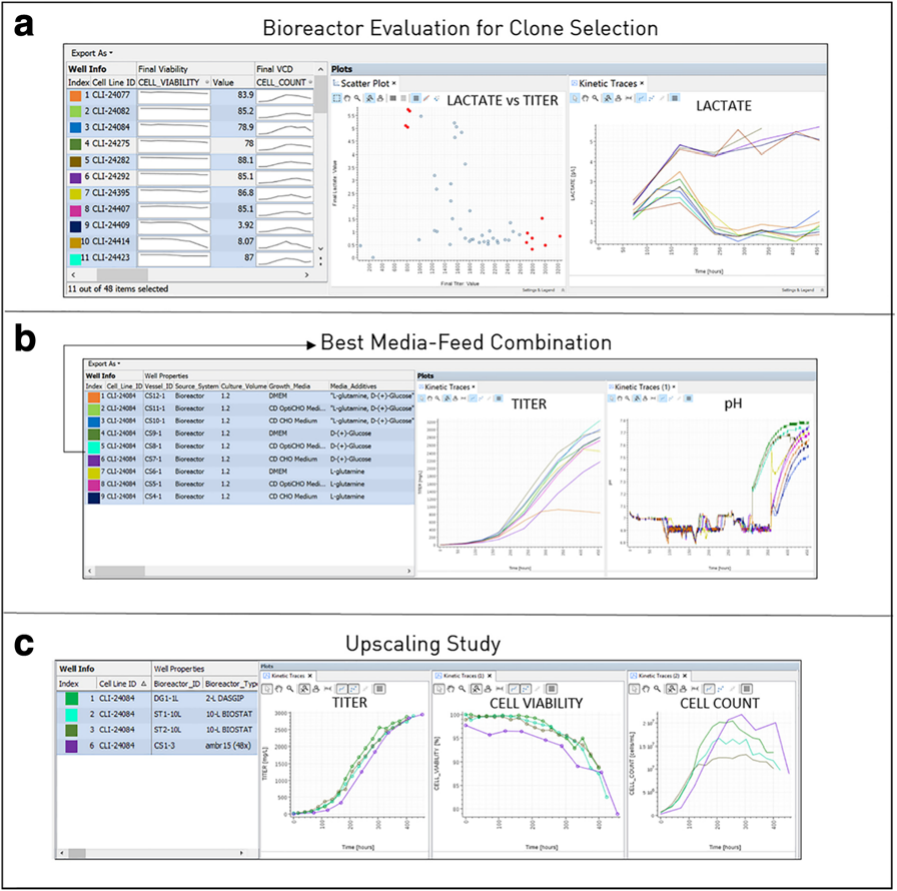
Example use cases for evaluating Bioreactor results with Genedata Bioprocess. **a** Identification of top performer cell line. The screenshot highlights interactive user interface, allowing to visualize and select cell lines based on custom criteria. **b** Tracking of process conditions together with online and offline performance analytics. The system allows to flexibly define tracked parameters and select optimal process conditions. **c** Comparison of process performance across different reactor scales. The open architecture makes Genedata Bioprocess a provider agnostic system which allows to aggregate and compare data regardless of provider

## P-108 Implementation of a virus barrier media filter into fed-batch bioprocesses

### Kimberly Mann, Michael McGlothlen, Joe Orlando, Jonathan Broe, Patricia Kumpey, Kristina Cunninham, Yuanchun Zeng, David Nhiem, Robert Smith, Christina Cabrello, Venkata Raman, Rong-Rong Zhu, Soleil Le, Nhung Nguyen, Danielle DeCesaro, Mary Priest, Jeremy Perreault, Kevin Rautio

#### EMD Millipore Corporation, Bedford, Massachusetts, 01730, USA

##### **Correspondence:** Kimberly Mann (kimberly.mann@emdmillipore.com)

**Background**

Upstream bioprocesses are at particular risk of contamination from adventitious agents. The typical 0.1 μm filters used at this step protect bioreactors from bacteria and mycoplasma but offer no protection from viral contaminations. A new polyethersulfone (PES) upstream virus filter, Viresolve® Barrier, has demonstrated high levels of microorganism retention - full retention for bacteria and mycoplasma (>8.0 LRV - Log Reduction Value) and ~ 5 LRV for small viruses, such as parvoviruses. It also has improved flow and capacity as compared to virus removal filters designed for monoclonal antibody purification.

Given the small pore size of virus retentive filters, implementing a virus filter upstream of the bioreactor raises the question of whether critical cell culture media components are removed. Therefore, it is important to evaluate the cell culture performance and protein quality attributes using virus-filtered media to ensure that filtration does not negatively impact the process.

**Materials and methods**

EX-CELL® CHO media and corresponding feeds were processed through either Viresolve® Barrier filters or 0.22 μm filters (control). Media composition post-filtration was evaluated by high performance liquid chromatography (HPLC), inductively coupled plasma/optical emission spectrometry (ICP-OES), and nuclear magnetic resonance (NMR).

Recombinant CHO cells were cultured in fed batch culture. Cell density and viability were measured by Vi-Cell^TM^ cell viability analyzer while metabolites were analyzed by BioProfile® FLEX analyzer. Shake flasks and bioreactors were utilized to verify that surfactants, such as poloxamer, (which are essential for shear protection in stirred tank bioreactors and can be difficult to filter) have not been removed during filtration.

Monoclonal antibody titer was quantitated by Protein A HPLC. Characterization of the antibody product quality was assessed via weak cation-exchange chromatography (charge heterogeneity), size exclusion chromatography (aggregate profile), and 2-AB fluorescent labeling with NP-UPLC (glycan species).

**Results**

Media and feed compositions were unaffected by filtration through the virus barrier filter. No significant differences in concentrations were observed with ICP-OES (trace metals) or HPLC (amino acids and water soluble vitamins). NMR showed no change in the organic composition of the media including poloxamer. The aromatic region with vitamin and amino acid signals is shown (Fig. 1a).

Cell cultures showed no differences in cell growth or titer, in either shake flasks or bioreactors (Fig. 1b). Cell viability was unaffected, metabolite levels were within limits, and titer was consistent. The protein quality of the secreted antibodies showed no differences in the glycosylation pattern (Fig. 1c), amount of aggregates or charge variants.

**Conclusions**

The risk of virus contamination in the bioreactor remains a concern for biotherapeutic manufacturers as there is no universal technology that provides a reliable, cost effective solution for virus removal that can be applied to all components of cell culture media. This study evaluated the Viresolve® Barrier filter that provides an efficient and easy way to protect bioprocesses from adventitious virus contamination.

Study results demonstrated that media and feed compositions, cell culture performance, and product quality were unaffected by filtration through the Viresolve® Barrier filter. Implementation of Viresolve® Barrier filters provides efficient filtration performance, high virus retention, and minimal cell culture impact and offers a viable option to improve the overall virus risk mitigation strategy for the manufacture of biotherapeutics.

**Reference**

Liu S, et al. **Development and qualification of a novel virus removal filter for cell culture applications.** Biotechnol Prog. 2000;16(3):425-434

**Fig. 1 (abstract P-108).**
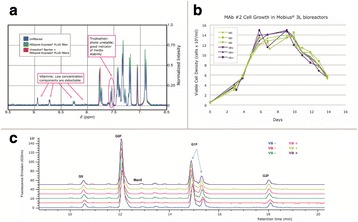
NMR did not detect any compositional change in the media (**a**). Micromolar levels of components can be detected (aromatic region shown). Cell growth in Mobius® 3 L single-use bioreactors (**b**) shows no difference with the use of Viresolve® Barrier filtered media and feeds (VB+) as compared to 0.22 μm filtered controls (VB-). Glycan profile (**c**) shows no change with the use of Viresolve® Barrier filtered media

## P-123 Design and evaluation of next-generation biologics for cancer immunotherapy

### Maria Wendt^1^, Guido Cappuccilli^1^, Carl Bruder^1^, Chris Smith^2^, Christoph Freiberg^1^, Yang-Chieh Chou^3^, Hans Peter Fischer^1^

#### ^1^Biologics, Genedata, Basel, Switzerland; ^2^Biologics, Genedata, Boston, MA, USA; ^3^Biologics, Genedata, San Francisco, CA, USA

##### **Correspondence:** Maria Wendt (biologics@genedata.com)

**Background**

Bi- and multi-specific antibodies, antibody-cytokine fusion proteins, non-immunoglobulin scaffolds, chimeric antigen receptors (CARs), engineered T-cell receptors (TCRs) and TCR-based bispecific constructs can provide significant advantages for use in cancer immunotherapy. However, as highly engineered molecules they pose new challenges in design, engineering, cloning, expression, purification, and analytics. We have thus implemented an infrastructure that addresses these challenges and enables the industrialization of these various novel therapeutic platforms.

**Material and method**

In close collaboration with leading biopharmaceutical companies, we implemented a workflow, data management and analysis support system, Genedata Biologics™, enabling the automated design, screening, and expression of large panels of therapeutic candidates using these novel technologies. We have also built tools for developability and manufacturability assessments of these complex molecules. We have ensured that there is a seamless integration of all data generated and that functionalities such as bulk protein and vector generation using our *in silico* cloning engine, configurable library of template vectors and cloning strategies, fully annotated *in silico* protein molecules and DNA constructs, and DNA synthesis verification support, can be used for the newest protein formats and molecule topologies.

**Results and discussion**

We implemented data structures and data handling systems, which mirror how these complex next-generation biologics molecules and cell lines are being designed, screened, and analyzed. The result successfully addresses workflows for TCR optimization and engineering. We exemplified this with the generation and evaluation of a panel of engineered TCRs with an alpha chain CDR3 randomization and successfully supported the analysis and selection of beneficial mutations. The system also successfully supported workflows for the design and generation of a panel of TCR-based bispecifics (TCR coupled with anti-CD3) using automated molecule registration and *in silico* cloning tools and subsequent capture of expression, purification, and functional and analytical characterization data. On the CAR-T cell front, the system is able to provide traceability of the work from antibody generation, optimization, CAR engineering (e.g., attachment to the scFv with CD3-zeta and co-stimulatory domains to mimic the natural TCR complex) to the engineering of the T-cell. The Genedata Biologics platform successfully enabled automation, increased data integrity and traceability during research and development work, and will contribute towards the industrialization of these very exciting novel approaches for cancer immunotherapy.

**Acknowledgements**

Allison Kurz, Gian Andrea Signorell, Genedata, Basel, Switzerland.

**Fig. 1 (abstract P-123).**
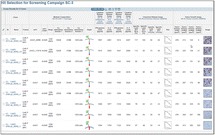
Next-generation biologics molecules are composed from a number of specific subdomains. Each type of molecule is composed of a specific set of domains, which must be mirrored in the registration and further research and development workflow. Molecule registration and hit-selection using data from a number of assays is shown here using the example of CAR-T cells. The image is a screenshot from the Genedata Biologics™ software

## P-130 Optimal selection of therapeutic antibodies and production cell lines by assessment of critical quality attributes and developability criteria

### Martin Moravec^1^, Christoph Freiberg^1^, Lukasz Gricman^1^, Amanda Fitzgerald^2^, Yang-Chieh Chou^3^, Hans-Peter Fischer^1^

#### ^1^Biologics, Genedata, Basel, Switzerland; ^2^Biologics, Genedata, Boston, MA, USA; ^3^Biologics, Genedata, San Francisco, CA, USA

##### **Correspondence:** Martin Moravec (bioprocess@genedata.com)

**Background**

The increasing cost of bringing a new drug to the market has put significant pressure on biopharma organizations. To increase efficiency in R&D processes and reduce costs, organizations need to evaluate potential drug candidates earlier in the R&D process, eliminate those with undesirable characteristics, and focus on the most promising candidates. After designing and thorough testing of successful candidates, efficient production of new biological entities in mammalian cell lines is necessary. The main goal here is to find a suitable cell line and optimal upstream and downstream processing conditions that not only lead to a satisfactory product yield, but also to a product with the desired biochemical properties. The evaluation of production cell lines, processes, and product quality attributes is performed earlier and in higher throughput for an increasing number of drug candidates. In addition, new methods in molecular and cell biology (e.g., novel genome engineering approaches such as CRISPR/Cas9), in analytics [e.g., Process Analytical Techniques (PATs)], in process miniaturization, and in automation promise to make process development more efficient.

However, the management and analysis of the increasing amount of experimental data during candidate selection and cell line and process development has become a bottleneck. In addition, quality-compromising steps in biopharma organizations can negatively impact the cost of goods and substantially prolong the drug candidate’s time to market. Therefore, systems for integrated management and analysis of well-structured and curated data that comprehensively integrate molecule and sample information, manufacturing process parameters, and process and product quality attributes are needed. Critical quality attribute (CQA) assessment should be enabled along the whole bioprocess development workflow, including cell line development, upstream and downstream process development, as well as analytical and formulation development.

**Materials and methods**

We have developed a comprehensive platform, Genedata Bioprocess™, which supports drug candidate developability and manufacturability assessment and bioprocess development. The platform captures and structures the cell line and process parameters together with analytical data for cell lines, processes, and protein products. The protein analytical data being tracked include biological data (such as bioactivity, immunogenicity), and physicochemical properties. These properties include glycosylation, chemical liabilities (such as deamidation and oxidation), aggregation, stability under different conditions (low pH, low and high temperature), solubility, and impurities. Genedata Bioprocess™ simplifies and streamlines laborious, manual process and supports tools for molecule, clone and process selection. Furthermore, the platform allows for seamless integration with laboratory instruments, statistical software packages, and custom solutions.

**Results and conclusions**

Here, we present use cases showing how to identify and annotate liability sites prone to chemical modifications (Fig. 1a) and how to monitor CQAs of molecules allowing to assess developability more efficiently. We show how the analytical data generated in the course of a developability assessment are compiled to select the best drug candidate (Fig. 1b). Implemented traffic-light systems indicate where molecules harbor issues such as in case of the antibody TPP-86, which is compromised by low temperature and repeated freeze-thaw operations. The same assessment views can also be applied on batches and cell lines. The underlying data can be visualized graphically. As an example, we show glycan types of products obtained from different cell line clones generated in a cell line development campaign for the molecule TPP-86 (Fig. 1c). Even though the selected clone CLI-35 meets the glycosylation criteria (e. g., <13% afucosylation, <40% galactosylation, <2% sialylation), the produced molecule harbors some stability issues as mentioned above. Therefore, more attempts would be needed either in formulation or in reengineering of the complimentarity determining regions (CDRs) in order to provide a developable TTP-86-like drug candidate.

**Acknowledgements**

Allison Kurz, Gian Andrea Signorell, Genedata AG

**Fig. 1 (abstract P-130).**
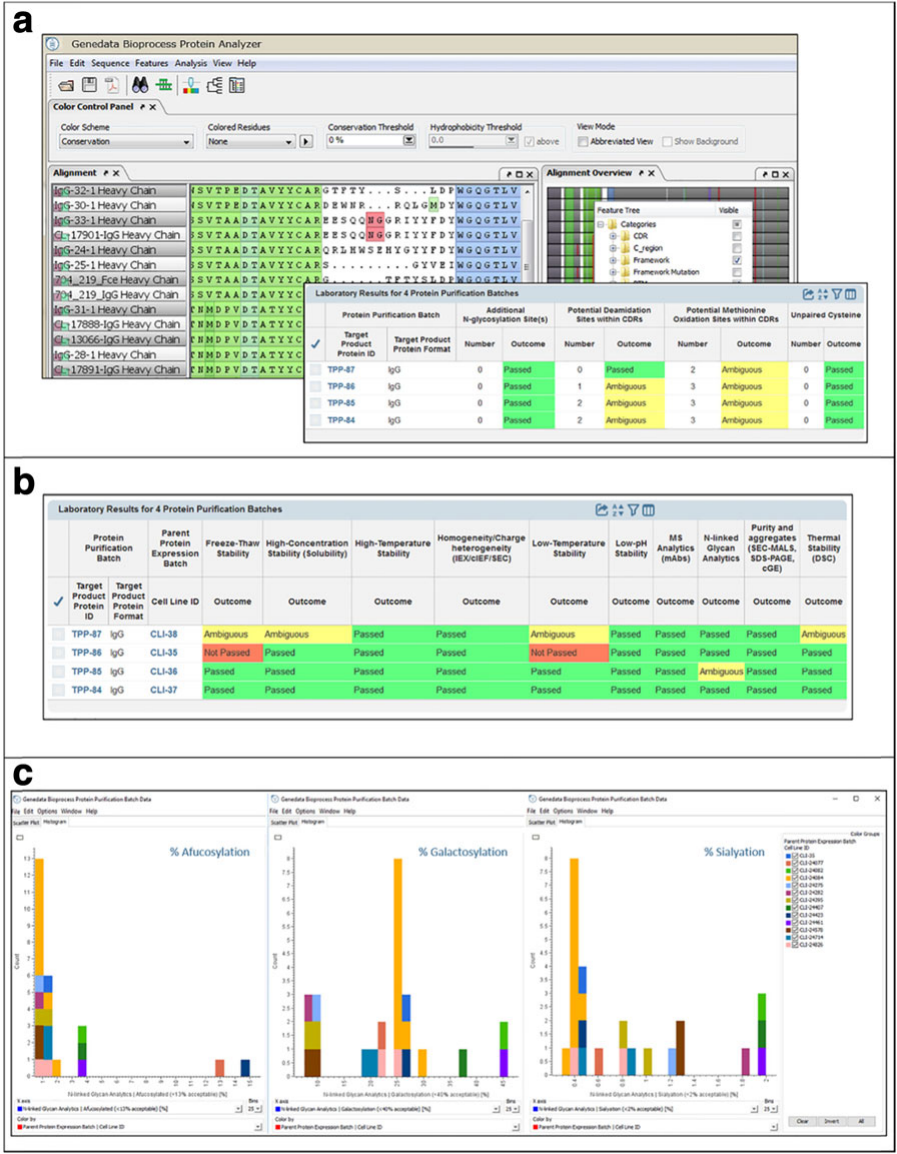
**a** Built-in sequence database and automated sequence-processing pipeline of Genedata Bioprocess™. In the upper panel the alignment of IgG1 sequences plus graphical representation of potential liability motifs such as deamidation, oxidation, glycosylation motifs in the CDR3 sequence of candidates are shown. Here, for four IgG1 drug candidates an overview of potential molecule liabilities for the developability assessment using a traffic-light system is depicted (lower panel). **b** Traffic-light system for developability assessment of drug candidates using distinct cell lines. The outcome of a broad panel of analytical tests is displayed, it is based on thresholds set for individual test parameters. Here, the drug candidate TPP-84 produced in the cell line CLI-37 fulfills all desired developability criteria. **c** Visualized data refer to N-linked glycan analysis. Here, we show the degrees of afucosylation, galactosylation and sialyation for drug candidate molecule TPP-86 expressed in different CHO-K1 cell lines generated during a cell line development campaign. The cell line CLI-35 fulfills the desired glycosylation requirements (refer to panel **b**)

## P-132 High glucose concentration and low specific cell growth rate improve specific r-tPA productivity in chemostat culture of CHO cells

### Mauricio Vergara^1,2^, Andrea Müller^2^, Verónica Avello^2^, Cristian Acevedo^3^, Julio Berrios^2^, Juan G Reyes^1^, Claudia Altamirano^2,4^

#### ^1^Chemistry Institute, Pontifical Catholic University of Valparaiso, Valparaiso, 2373223, Chile; ^2^Biochemical Engineering School, Valparaiso, 2340000, Chile; ^3^Institute of Physics, Universidad Técnica Federico Santa María, Valparaiso, 2340000, Chile; ^4^CREAS, Valparaiso, 2373223, Chile

##### **Correspondence:** Claudia Altamirano (claudia.altamirano@pucv.cl)

**Background**

Environmental process variables are often used as tools to optimize the performance of mammalian cell cultures to achieve higher cell densities and high productivities of r-proteins (q_P_). The manipulation of culture temperature in the range of mild hypothermia (MH) (35-30°C) [1,2], as well as different glucose availability scenarios [3,4], has been shown to improve productivity in different cell lines. However, the manipulation of these variables individually or together has a concomitant effect on the rate at which cells grow, masking the net response exhibited by the cells. In order to identify the effects of these variables, we have taken advantage of the use of the chemostat culture.

**Materials and methods**

Chemostat cultures were performed at two dilution rate (D)(0.010 or 0.018(h-1)), two temperatures (33 or 37°C) and three feed glucose concentrations (20, 30 or 40 mM). The response was analysed considering r-protein production, cell growth and key metabolites. r-tPA protein concentration was determined by immunoassay (TriniLIZE tPA KIT); cells were counted using a hemocytometer and cell viability was determined by the method of exclusion using trypan blue (T8154, Sigma, USA); glucose, lactate and glutamate were determined by enzymatic assay using a biochemical analyser YSI (Yellow Spring Instruments). Statistical analysis of the results was performed by ANOVA (Design-Expert 7 for Windows).

**Results**

A decrease in cell density was observed in response to an increase of glucose feeding concentration, regardless of temperature or specific growth rate (in this case μ=D) evaluated. The maximum cell densities were reached at 20mM, achieving 1.65 and 1.50 x10^6^ cells/ml at 37/33°C and 0.018(h^-1^); and 1.10 and 1.33 x 10^6^ cells/ml at 37/33°C and 0.010(h^-1^) respectively (Fig. 1a). The increase in glucose concentration from 20 to 40mM resulted in an q_P_ increase of 3 and 3.3 fold at 33°C/0.018(h^-1^) and 37°C/0.018(h^-1^) respectively. A lower increase of 2.4 and 1.8 fold was reached at 33°C/0.010(h^-1^) and 37°C/0.010(h^-1^) respectively (Fig. 1b). The highest q_P_s were reached at 37°C and 0.010(h^-1^). However, a positive effect of MH was not observed, in contrast to that observed in batch culture [1, 2, 3]. This behaviour suggests that low μ is a main factor on increased r-protein production in batch cultures exposed at MH condition.

The specific consumption rate of glucose was significantly increased by the glucose increase from 20 to 40mM and reduced by MH (Fig. 1c). At 0.010 (h^-1^) the specific production rate of lactate (q_Lac_) was increased by glucose increase, independent of the culture temperature used. While at 33°C/0.018(h^-1^) the q_Lac_ decreased with increasing glucose concentration and at 37°C/0.018(h^-1^) a maximum consumption was observed at 30 mM glucose (Fig. 1d). The lactate-glucose yield (Fig. 1e) not showed relevant changes at 0.010(h^-1^), while at 0.018(h^-1^) this yield showed a more efficient utilization of glucose, as glucose concentration was increased. However, this last behaviour was not reflected in an increase of r-protein production.

**Conclusions**

The concentration of glucose has the greatest impact on the behaviour of the culture, and its increase affects positively the protein productivity. The MH did not improve proteins productivity of CHO cells producing tPA under the different conditions evaluated; low dilution rate and at high glucose concentration impact positively the protein productivity and the metabolism exhibited by the cells.

**Acknowledgements**

This work was supported by FONDECYT 3150373 and FONDECYT 1161452.

**References**

1. Yoon SK, Choi SL, Song JY: **Effect of culture pH on erythropoietin production by Chinese hamster ovary cells grown in suspension at 32.5 and 37.0°C.** *Biotechnol Bioeng* 2005 **89**:345-56.

2. Lin ChY, Huang Z, Wen W: **Enhancing Protein Expression in HEK-293 Cells by Lowering Culture Temperature**. *PLoS One*. 2015 **10**:e0123562.

3. Berrios J, Díza-Barrera Á, Bazán C: **Relationship between tissue plasminogen activator production and specific growth rate in Chinese Hamster Ovary cells cultured in mannose at low temperature.** *Biotechnol Lett* 2009 **31**:1493-7.

4. Liu Z, Dai S, Bones J. **A quantitative proteomic analysis of cellular responses to high glucose media in Chinese hamster ovary cells.** *Biotechnol Prog*. 2015 **31**:1026-38.

**Fig. 1 (abstract P-130).**
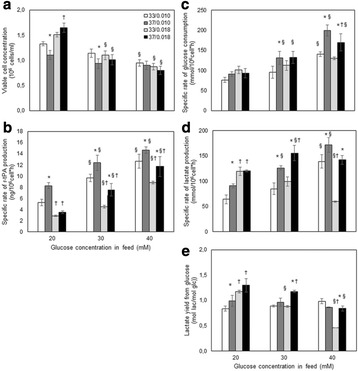
Impact of specific growth rate, culture temperature and glucose concentration in medium on viable cell concentration, specific hr-tPA productivity and metabolism in CHO cell cultures. (White) Low-D at 33°C; (Dark grey) Low-D at 37°C; (Light grey) High-D at 33°C; (Black) High-D at 37°C. * (p<0.05) Significant differences respect to counterpart culture at different T°, same glucose concentration and same D, Tukey’s test. ^§^ (p<0.05) Significant differences respect to counterpart culture at same T°, different glucose concentration and same D, Tukey’s test. ^†^ (p<0.05) Significant differences respect to counterpart culture at same T°, same glucose concentration and different D, Tukey’s test

## P-134 Online and real-time control of CHO cell specific growth rate throughout cultures in bioreactor

### Mengyao Li, Bruno Ebel, Frantz Fournier, Emmanuel Guedon, Annie Marc

#### Laboratoire Réactions et Génie des Procédés, UMR 7274, CNRS-Université de Lorraine, 2 avenue Forêt de Haye, TSA 40602, 54518 Vandœuvre-lès-Nancy, France

##### **Correspondence:** Bruno Ebel (bruno.ebel@univ-lorraine.fr)

**Background**

Mammalian cell cultures are the most commonly used bioprocess for the production of therapeutic recombinant proteins such as monoclonal antibodies (mAbs). Facing to the increasing demand of these biopharmaceuticals, the FDA has initiated the Process Analytical Technology (PAT) framework in order to encourage pharmaceutical industries to use innovative technologies to monitor in real time the critical process parameters (CPPs), and to ensure the final product quality [1].

One of the most important CPPs for cell culture bioprocesses is the specific growth rate (μ), which is a direct indicator of cellular physiological state. Indeed, μ is sensible to culture conditions and its value decreases when cells are in the unfavourable environment for growth [2], which may greatly influence mAb production and quality. However, until this day, the online monitoring of μ remains a great challenge for mammalian cell culture bioprocesses.

**Materials and methods**

IgG-producing CHO cells were cultured in 2 L stirred bioreactors equipped with an *in situ* dielectric spectroscopy (Hamilton). Operating conditions were fixed at 90 rpm, 50% of air saturation, pH 7.2 and 37°C. Permittivity of cell culture was measured every 12 min, which allowed to calculate in real time the VCD by using a previously established linear correlation. Then, a model of online estimation of μ was developed based on VCD prediction and cell mass balance equations. Several signal noise filters and various calculation methods were evaluated to reach better model stability. Cell cultures were performed in both batch and feed-harvest modes. Feed-harvest cultures consisted of sequential renewals of 2/3 volume of the culture medium by following different strategies.

**Results**

This study proposed an innovative methodology based on dielectric spectroscopy to monitor in real time the cellular physiological state, by online estimating the specific growth rate (μ) of cells. Model of online estimation of μ was developed from cultures in batch mode, and was validated by comparing online estimated μ with the experimental ones calculated at the end of the culture. With this model, the moment when μ started to decrease significantly, which indicated that cells were no longer in the exponential growth phase, was identified as the critical moment. To demonstrate the interest of online estimation of μ, the developed model was applied to a feed-harvest culture, where the medium renewals were performed at the critical moments indicated by the model. This culture was then compared with the traditional feed-harvest culture where medium renewals were performed by following offline measurements of glucose and glutamine. We found that the online strategy allowed to maintain the value of μ by renewing the medium at the right time, while the values of μ varied a lot when using offline strategy. Moreover, by using the online estimation of μ, the glycosylation of IgG was kept at a high level (about 95%) throughout the whole culture. However, for the culture using offline strategy, the glycosylation level decreased progressively and was only about 75% at the end of the culture (Fig. 1).

**Conclusions**

Model of online estimation of μ was developed by using dielectric spectroscopy, which allowed to monitor the physiological state of cells in cell culture bioprocesses. Implementation of this model in feed-harvest cell culture led to better mAb glycosylation, which demonstrates clearly the potential of this methodology in mAb production bioprocesses.

**References**

1. Hinz DC: **Process analytical technologies in the pharmaceutical industry: the FDA’s PAT initiative**. Anal. Bioanal. Chem. 2006, 384: 1036‑1042.

2. Lao MS, Toth D: **Effects of ammonium and lactate on growth and metabolism of a recombinant Chinese hamster ovary cell culture**. Biotechnol. Prog. 1997, 13: 688–691.

**Fig. 1 (abstract P-134).**
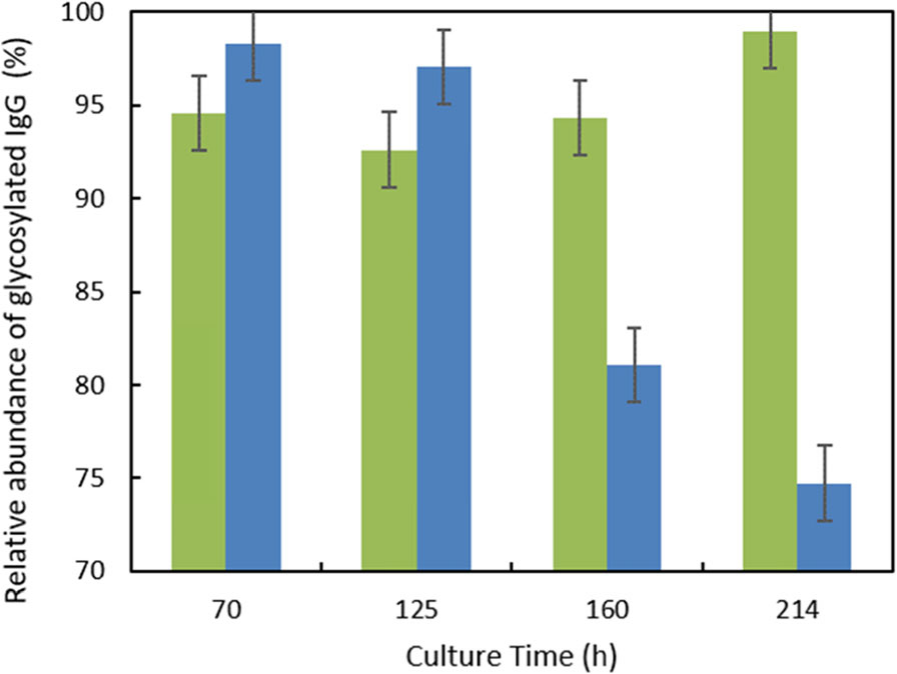
Relative abundance of glycosylated IgG produced in feed-harvest culture using online strategy (green) and in traditional feed-harvest culture using offline strategy (blue)

## P-135 Solid phase enzymatic re-modelling to produce single glycoform antibodies

### Michael Butler^1^, Venkata S. Tayi^2^

#### ^1^NIBRT, Dublin, Ireland; ^2^Glaxosmithkline, King of Prussia, PA, USA

##### **Correspondence:** Michael Butler (michael.butler@nibrt.ie)

**Background**

Monoclonal antibodies are normally synthesised from transfected mammalian cells as heterogeneous mixtures of glycoforms [1]. However, clinical efficacy may depend upon single glycoforms which have been difficult to isolate [2]. We have now developed an efficient method for generating single glycoforms by solid phase re-modelling which is superior to previous methods because it allows a sequential series of enzymatic changes without the need for intermediate purification of the antibody. Solid-phase binding exposes the antibody glycans to enable easier access of the transforming glycosylation enzyme.

**Materials and methods**

The antibodies subjected to modification were a chimeric human/camelid monoclonal antibody (EG2), a humanized monoclonal antibody (IL8), a full size chimeric antibody (Cetuximab) and polyclonal antibodies obtained from pooled human serum. The antibodies were bound to a Protein A column using conditions typical of Mab purification (Fig. 1). After washing out non-bound impurities by a neutral pH buffer, each antibody was subjected to enzymatic modification directed to a targeted glycan profile (Table 1). The antibodies were then eluted with a low pH buffer and neutralized. The glycan profiles were analysed following glycan removal with PNGase F, labelling with 2-aminobenzamide and separation on a HILIC-HPLC column [3].

**Results**

Prior to enzymatic modification glycan analysis of all 4 antibodies showed variable galactosylation and sialylation typical of human Abs. This included a distribution of FG0, FG1, FG2, FS1 and FS2 with galactosylation indices ranging from 0.22 for IL8 to 0.64 for EG2. There was minimal sialylation in IL8 but up to 11% in EG2.

Glycan modifications were made as each antibody was held on a Protein A column in accordance with procedures shown in Table 1. Agalactosylated glycans were enriched by treatment with the single addition of galactosidase and neuraminidase. This resulted in 83-95% of agalactosylated structures in the Mabs and 65% in the polyclonal antibody.

Galactosylated antibodies (>95% yield) were produced by a single stage reaction involving sialidase and by galactosyltransferase with UDP-Gal. Breakdown of the glycans to a trimannosyl core was accomplished by treatment of the agalactosylated structures with hexosaminidase. This produced a yield of 76-80% of the FM3 structure with a small remainder of FA1.

Sialylated antibodies (>95%) were produced by a 2 stage reaction involving sialidase, galactosyltransferase and finally treatment with 2,6 sialyltransferase in the presence of CMP-NANA. The latter reaction produced equimolar quantities of monosialylated and disialylated Cetuximab and polyclonal antibodies. The results suggest that for human antibodies (150 kDa) there may be a limitation for sialylation given the steric constraints between the two CH2 domains of the dimeric structure. The ability to sialylate the smaller camelid antibody (80 kDa) was greater resulting in a high (>90%) level of disialylated glycans. This suggests that the steric constraints for glycosylation may be lower. These sialylated antibodies have significant potential clinical importance for their ant-inflammatory activities.

**Conclusions**

We have modified the glycans of antibodies following immobilization on an affinity ligand column. This allows enzymatic transformation in a solid state that has a distinct advantage over the equivalent transformation in solution because the enzymes and buffers can be washed out on completion of the modification leaving the antibody still attached to the affinity ligand. This enables repeated rounds of an enzymatic reaction or sequential reaction steps without the need for intermediate antibody purification. The antibody can be removed eventually from the column by application of an elution buffer once all desired glycan modification have been made. Since affinity ligand purification of antibodies is performed routinely as an initial step of purification after cell culture, the glycan modification can easily be incorporated into this process. The enrichment of the resulting antibody for a targeted glycoform can enhance the potential therapeutic efficacy as it is known that specific glycoforms are required for certain biological effects.

**Acknowledgements**

Financial support is gratefully acknowledged from Natural Science and Engineering Research Council of Canada (NSERC) through a strategic network program (MabNet).

**References**

1. Spearman,M. and Butler,M.: **Glycosylation in Cell Culture.** Chapter 9 of Cell Engineering vol. 9, p237-258: Animal Cell Culture (ed. Al-Rubeai,M) publ. Springer 2015.

2. Lu, J., Sun, P. D., Structural mechanism of high affinity FcgammaRI recognition of immunoglobulin G. *Immunological reviews* 2015, *268*, 192-200.

3. Krahn,N., Spearman,M., Meier,M., Dorion-Thibaudeau, J., McDougall,M., Patel,T.R., De Crescenzo,G., Durocher,Y., Stetefeld,J, Butler**.**M**.: Inhibition of glycosylation on a camelid antibody uniquely affects its FcγRI binding activity.** European Journal of Pharmaceutical Sciences 96:428-439 2017.

**Fig. 1 (abstract P-135).**
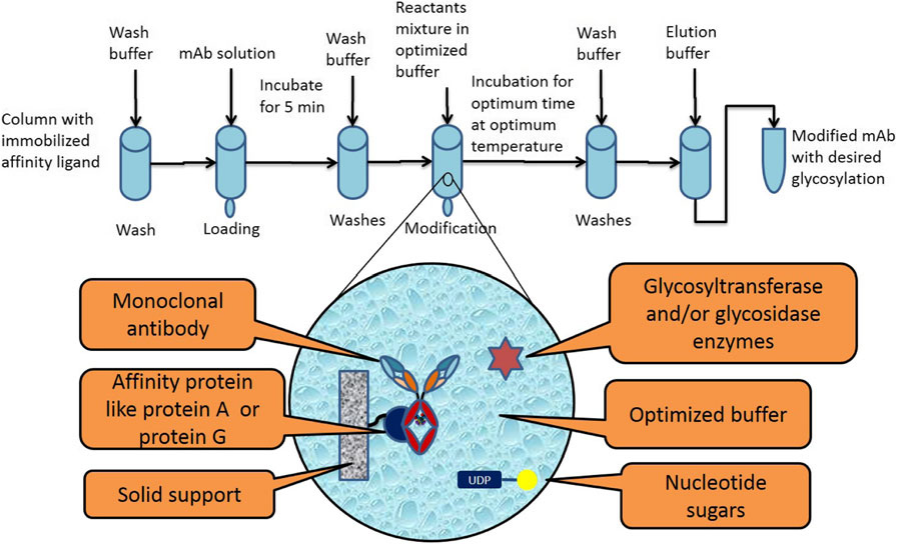
Schematic representation of the steps involved in the *in-vitro* modification process

**Table 1 (abstract P-135).** Enzymatic reactions were conducted in 25 mM Tris + 50 mM NaCl buffer at the specified pH for 48-72 h. The % yields of the targeted glycan structures are shown. FG0 = fucosylated agalactosylated, FG2 = fucosylated digalactosylated, FM3 = fucosylated trimannosyl core, FA1 = fucosylated mannosyl core plus one GlcNAc, FS1 = fucosylated monosialylated biantennary, FS2 = fucosylated disialylated biantennary

Targeted modification	Stages	Enzyme Reaction	Antibody	Final structure	Yield %
Agalactosylation	1	3 units/μL β(1,4) galactosidase, 3 units/μL neuraminidase at pH 6.6	EG2	FG0	85
			Cetux	FG0	94
			IL8	FG0	83
			Polyclonal	FG0	62
Galactosylation	1	2 milli-units/μL β(1,4) galactosyltransferase, 3 mM UDP-galactose, 10 mM MnCl_2_, 3 units/μL neuraminidase at pH7.5	EG2	FG2	96
			Cetux	FG2	95
			IL8	FG2	95
			serum	FG2	96
Trimannose (FM3 + FA1)	2	Stage 1: 3 units/μL β(1,4) galactosidase, 3 units/μL neuraminidase at pH6.6 Stage 2: 20 units of β-N-acetylglucosaminidase at pH6.6	EG2	FM3	80
				FA1	11
			Cetux	FM3	78
				FA1	15
			IL8	FM3	76
				FA1	18
Sialylation (FS1 + FS2)	2	Stage 1: 2 milli-units/μL β(1,4) galactosyltransferase, 3 mM UDP-galactose, 10 mM MnCl_2_ at pH7.5 Stage 2: 100 μg/mL α(2,6) sialyltransferase, 3 mM CMP-Neu5Ac at pH7.5	EG2	FS2	76
				FS1	16
			IL8	FS2	37
				FS1	38
			Polyclonal	FS2	43
				FS1	46

## P-141 Impact of microvesicles over cell growth and recombinant protein production from CHO cells

### Misba Majood, Sneha Arora, Shrikant Kumar, Chandresh Sharma, Shinjini Bhatnagar, Susmita Chaudhuri, Niraj Kumar

#### Center for Biodesign and Diagnostics, Translational Health Science and Technology Institute, Faridabad, India

##### **Correspondence:** Niraj Kumar (nkumar@thsti.res.in)

Misba Majood and Sneha Arora contributed equally

Shinjini Bhatnagar, Susmita Chaudhuri and Niraj Kumar contributed equally

**Background**

CHO cells are the main workhorse for production of recombinant proteins at commercial scale. Further improvements in production are of eminent importance to meet global demand at affordable cost. Cell drived microvesicles can induce/reduce cell proliferation and hence have capabilities to regulate bioprocess-related cellular phenotypes (like cell growth, cell death, recombinant protein production, and/or host cell proteins composition) [1,2]. This is mainly because microvesicles can be enriched/deprived for specific proteins, based on their functional purpose and their cellular origin. Recently, microvesicles purified from the supernatant of T24 bladder cancer cells were reported to be enriched for Bcl-2 and cyclin D1 (anti-apoptotic proteins), but deprived for Bax and caspase-3 proteins (pro-apoptotic proteins) contributing towards immunity against programmed cell-death [2]. However, impact of microvesicles on CHO-based bioprocess has not been evaluated yet. Therefore, in this investigation, we aimed to evaluate their impact on cell growth and recombinant protein production from CHO cells.

**Materials and methods**

CHO-K1 cells were grown in chemically-defined protein-free culture medium (Life Technologies-1835273) in shake flask (GX-00125P). The different fractions of spent-media (microvesicles and microvesicle-free spent media) were collected using ultracentrifugation method [1,3]. Quality of different fractions was ensured using Western blotting for exosomal marker, CD63 (SC-15363) and coomassie stained gel for loading control (Fig. 1a). To evaluate impact on cell growth, cells were seeded with microvesicles and microvesicle-free fraction collected from log-phase of culture and cell counts were performed by ViCell using trypan-blue dye exclusion method. For impact on productivity, cell-free supernatant, collected from microvesicle-treated human IgG secreting CHO culture from stationary-phase of culture with respective control, was evaluated using ELISA (ab100547). Microvesicles collected from 10% of media (by volume) from routine maintenance cultures compared to working volume for microvesicle-supplementation were used in each experiment.

**Results**

The growth of microvesicle-supplemented cultures had shorter lag-phase and achieved 1.2 fold higher maximum cell density (1.46x10^6^ viable cells/mL) compared to untreated standard culture (1.21 x10^6^ viable cells/mL) and maintained higher for the remaining period of batch culture (Fig. 1b). However, microvesicle-free fraction did not had significant impact on growth. The viability of microvesicle-supplemented cultures, similar to microvesicle-free media supplemented, was also higher compared to standard culture suggesting potential use of microvesicles for regulating CHO growth in production cultures. This could be possibily because microvesicles have already been reported to be enriched with cell growth/death-regulating proteins and hence facilitating cell growth [2,3]. We have also observed abundance of cell cycle regulators including cyclin D1 in microvesicle-fraction compared to microvesicle-free spent-media in our laboratory (data not shown); however, further investigation are required to prove the hypothesis. The overall productivity of human IgG secreting CHO cells was also observed to increase by ~4 fold following supplementation of microvesicles to the culture without significantly affecting per-cell productivity. Since microvesicle-supplementation facilitates cell growth, increased number of viable producer cells in the culture could be expected to be the basis of observed increase in the overall productivity of the culture [2,3]. The further work is ongoing to in-depth explore the potential of microvesicles for improving recombinant protein production from CHO cells.

**Conclusions**

The data indicate that microvesicles secreted from CHO cells can improve cell growth and hence recombinat protein production in culture. Therefore, strategies need to be developed for sterile isolation of CHO microvesicles from routine maintenance cultures and their supplementation into the production culture for improving the performance of CHO-based production process.

**Acknowledgements**

Translational Health Science and Technology Institute, India; Science and Engineering Research Board, India; Department of Biotechnology, India.

**References**

1. Chaudhuri S, Maurya P, Kaur M, Tiwari A, Borth N, Bhatnagar S, Kumar N: **Investigation of CHO Secretome: Potential Way to Improve Recombinant Protein Production from Bioprocess**. *J Bioprocess Biotech* 2015, **5**:240-247.

2. Yang L, Wu XH, Wang D, Luo CL, Chen LX: **Bladder cancer cell-derived exosomes inhibit tumor cell apoptosis and induce cell proliferation in vitro**. *Mol Med Rep* 2013, **8**:1272-1278.

3. Kumar N, Gupta DG, Kumar S, Maurya P, Tiwari A, Mathew B, Banerjee S, Haldar S, Pillai J, Bhatnagar S, Chaudhuri S: **Exploring Packaged Microvesicle Proteome Composition of Chinese Hamster Ovary Secretome**. *J Bioprocess Biotech* 2016, **6**:274-285.

**Fig. 1 (abstract P-141).**
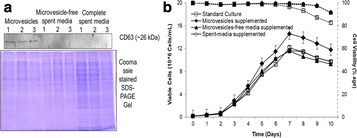
Evaluation of microvesicles and their impact on CHO cell growth over batch culture. **a** Evaluation of quality of different fractions (microvesicle and microvesicle-free fraction) using Western blotting for exosomal marker (CD63) and coomassie staining for equal protein loading. **b** Treatment of CHO cells with different fractions of spent-media to evaluate their impact on cell growth. Error bars represent standard deviation calculated using three biological replicate batch cultures

## P-151 Understanding the effects of utilizing a complete feeding supplement to modulate glycosylation profiles

### Ryan Boniface, Nicole DiNardo, Zofia Kozik, Jaime Goldfuss, Mark Stramaglia, Steve Gorfien

#### Thermo Fisher Scientific, 3175 Staley Rd., Grand Island, New York, USA, 14072

##### **Correspondence:** Ryan Boniface (ryan.boniface@thermofisher.com)

**Background**

The glycosylation profile of a recombinant protein is one of the most important attributes when defining product quality. Producing a protein with desired characteristics requires the ability to modify and target specific glycosylation profiles. Traditionally the approach to modify the glycosylation profile of a protein involves supplementing a culture with components that can improve galactosylation. Experimentation using this supplemental approach resulted in a dramatic increase in terminal galactosylation, but lacked the ability to easily and repeatedly target specific glycosylation profiles.

Using novel and proprietary technology, we have developed a feed (GlycanTune™) and a unique feeding process that will maximize growth and titer while being able to modulate glycan profiles. This new feed can be added as a standalone process that can result in a significant shift from G0F to G1F and G2F (maximum galactosylation). Using a unique fed-batch process, GlycanTune can also be used with a standard feed to dial in targeted glycosylation profiles. Through process development, we created a method where a transition point is used to switch from a standard feed to a glycan modulating feed. The timing of the transition point will determine the specificity of the glycan profile.

**Materials and methods**

(All materials were from Thermo Fisher Scientific unless otherwise indicated)

Cell culture: CHO DG44 derived recombinant cells expressing an IgG molecule were grown in Dynamis™ media supplemented with 4mM L-glutamine and 1:100 Anti-Clumping Agent. Culture conditions were maintained at 37°C, 8.0% CO_2_, 125 rpm. Cell densities and viabilities were measured using a Vi-CELL® counter (Beckman Coulter). Metabolites (glucose, ammonia, and lactate) and IgG were measured using a Cedex® BioHT Analyzer (Roche). 250mL flasks with 60mL starting volume inoculated at 0.3x10^5^ viable cells/mL in Dynamis™ medium. 2X EfficientFeed™ C+ AGT™ Supplement (EFC+) and/or 2X GlycanTune™ C+ Total Feed (GTC+) were supplemented at 1.7% on days 4 through 15 (20% total). Glycan modulation conditions involved transitioning from EFC+ to GTC+ on culture days 4, 5, 7, 9, 11, 13 and 15. Glucose was supplemented as required to maintain a concentration above 3g/L.

Glycan Analysis: IgG was purified from supernatant using POROS MabCapture A resin, then exchanged into 20mM phosphate buffer using Zeba™ Spin Desalting Plates (7K MWCO). N-linked glycans were digested with PNGase F and quantified using 100pmole maltohexose/maltopentose internal standards labeled with 8-aminopyrene-1,3,6-trisulfonic acid (ATPS) as described by Laroy et al [1] or the user guide for the Glycan Labeling and Analysis Kit (GlycanAssure™ user guide, Thermo Fisher Scientific). All CE separations were performed using the Applied Biosystems™ 3500xL.

**Results**

The timing of transition from EFC+ to GTC+ made it possible to target specific glycosylation profiles. Modulating G0F from 75% down to 32%, while increasing G1F and increasing G2F (Fig. 1). Transitioning to GTC+ early in culture resulted in a greater shift from G0F to G1F and G2F. Transitioning midway or late in culture resulted in a greater proportion of G0F compared to G1F and G2F.

**Conclusions**

Supplementation based approaches using glycosylation modulating media components to modify and target specific glycosylation profiles proved to be difficult. These approaches were able to increase terminal galactosylation (G1F and G2F), but lacked the ability to fine tune glycan profiles. This could result in numerous rounds of titration experiments to target specific glycan profiles that would likely remain inconsistent between cell lines, culture media and feeds, and process scale.

The development of a unique process made it possible to predictably target specific glycosylation profiles. Transition from standard feeding to GlycanTune allowed for precise targeting of glycan profiles. Transition to GlycanTune early in culture resulted in an increased shift from G0F to G1F and G2F. A transition late in culture resulted in increased G0F and decreased G1F and G2F.

**References**

1. Laroy W. Contreras R and Callewaert N.: **Glycome mapping on DNA sequencing equipment.** *Nature Protocols*, 206, 1(1), 397

**Fig. 1 (abstract P-151).**
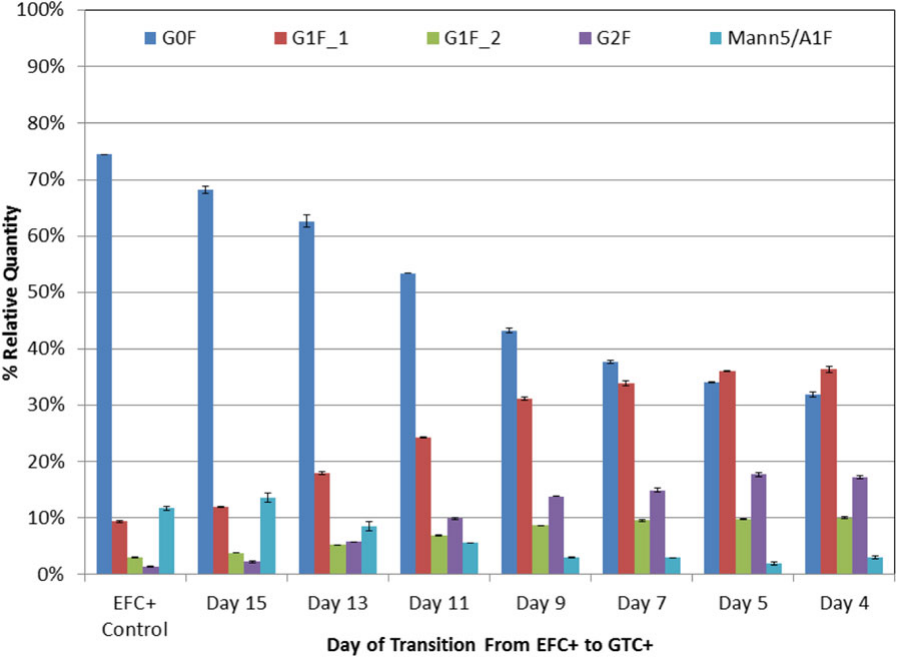
Glycan analysis from modulating glycosylation with EfficientFeed C+ and GlycanTune C+

## P-153 Surfactants in cell culture media: Impact on HEK and CHO cells in cultivation and transfection

### Sandra Klausing^1^, Ekaterina Rudiseva^2^, Falk Gronemeier^1^, Anica Schmidt^1^, Anja Träger^3^, Tanja Bus^3^, Anne-Kristin Trützschler^3^, Tim F. Beckmann^1^, Christoph Heinrich^1^

#### ^1^Xell AG, Bielefeld, Germany; ^2^Institute of Cell Culture Technology, Bielefeld University, Bielefeld, Germany; ^3^Laboratory of Organic and Macromolecular Chemistry (IOMC), Jena Center for Soft Matter (JCSM), Friedrich Schiller University Jena, Jena, Germany

##### **Correspondence:** Anica Schmidt (anica.schmidt@xell.ag)

**Background**

Surface active agents (surfactants) are commonly used cell culture medium components for reducing shear stress in non-static suspension culture. Despite the preferred application of poloxamer 188, there is an ongoing discussion within the cell culture community about surfactant-related process deviations. Furthermore, surfactants have been shown to interact with polyplexes as well as polymer nanoparticles within various applications (e.g. transfection, encapsulation). Better understanding the related mechanisms of action will facilitate finding alternative components for progressive cell culture media formulations.

**Materials and methods**

HEK 293‑F and CHO-K1 cell lines were cultivated in Xell’s chemically defined transfection media (HEK TF or CHO TF) in plain and baffled shake flasks to evaluate the impact of surfactants on shear stress. The surfactants were also evaluated regarding their impact on transient GFP expression via flow cytometry. The localisation of poloxamers in HEK 293-F culture was investigated using fluorescein-labeled Pluronic® F-68 and F-127, LysoTracker® Blue DND-22 (lysosomes) and CellMask™ Deep Red (cell membranes) in confocal microscopy. SEC and ^1^H-NMR measurements for structural analyses were performed at JCSM, Jena.

**Results**

Growth performance during precultures and batch curves in plain shaking flasks did not show any differences among tested surfactants or lots thereof, and cell densities reached 10-12·10^6^ cells/mL (Fig. 1a and b). Experiments with HEK 293-F cells at elevated power input in baffled shaking flasks revealed distinct differences between Pluronic® F-68, F-127 and Kolliphor® P188, with F-127 showing the best performance. Peak viable cell densities reached with lots A and B of Pluronic® F-68 and F-127 were comparable to those in plain shaking flasks, while those for Kolliphor® P188 and lots C and D of Pluronic® F-68 were significantly lower. Peak viale cell densities were of 2 – 12·10^6^ cells/mL (Fig. 1c). Similar transient transfection efficiency and mean fluorescence of transfected cells independent of applied surfactant and lot thereof indicated no major impact of respective poloxamer (Fig. 1d). Interestingly, experiments using fluorescein-labelled Pluronic® showed a time-dependent uptake into HEK cells. Visual tracking revealed an endocytic uptake of poloxamers by the cells (>10-fold increase in signal after 96 h) and its co-localisation with cell membrane and lysosomes. SEC (Fig. 1e) analyses showed differences between the tested poloxamers. Especially tested lots of Pluronic® F-68 revealed notable deviations in the low molecular weight fraction (peak 2, Fig. 1e), compared to the other poloxamers.

**Conclusion**

Cultures subjected to varying levels of shear stress showed distinct growth differences depending on used poloxamer. While experiments in plain shake flasks did not show any differences in growth, cultivations under elevated shear stress in baffled shake flasks resulted in lower peak viable cell densities with Kolliphor® P188 and some Pluronic® F-68 lots. It remaines unclear whether this can be explained by different membrane protective activities alone, or if other mechanisms, occuring during and after cellular uptake, contribute to this effect. Especially for the tested lots of Pluronic® F-68, SEC of surfactants showed differences in the low molecular weight fraction. This fraction mainly represents polyethylen oxide (PEO) (revealed by NMR), which is likely to be a remnant from synthesis. These observations indicate that the use of different poloxamers and lots thereof should be carefully evaluated, especially under elevated shear stress. Further experiments will focus on investigating distinct SEC fractions of poloxamers.

**Acknowledgements**

We would like to thank the German Federal Ministry of Education & Research (BMBF # 031A518B Vectura) for funding.

**Fig. 1 (abstract P-153).**
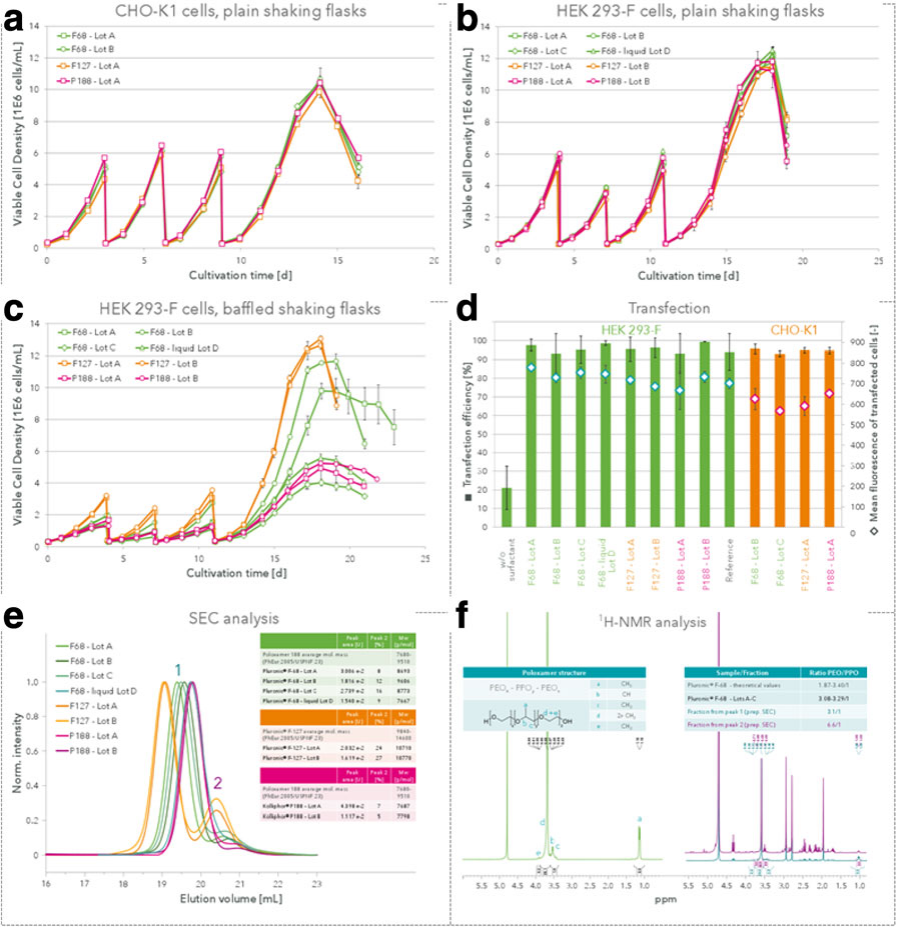
Impact of surfactants on growth (**a**-**c**) and transfection efficiency (**d**) in plain and baffled shake flasks, as well as structural analysis of surfactant lot-to-lot variation using SEC (**e**) and ^1^H-NMR (**f**) showing significant differences between poloxamer 188 and poloxamer 407 under elevated power input and in structural analyses. A commercially available medium suitable for transfection was used as a reference for the transfection experiments

## P-154 Overcoming the media design challenges in transient gene expression with CHO cell lines

### Sandra Klausing, Tim F. Beckmann, Christoph Heinrich

#### Xell AG, Bielefeld, Germany

##### **Correspondence:** Sandra Klausing (anica.schmidt@xell.ag)

**Background**

While transient gene expression (TGE) is routinely performed in HEK cell lines, high transfection of CHO (Chinese Hamster Ovary) cell lines has proven to be more challenging. For modern TGE procedures however, a scalable one-step solution is highly desired. Media components such as iron, which is essential for culture growth, chelators, and others can inhibit polyethylenimine (PEI)-mediated transfection [1], while polymers have been shown to enhance efficiency of electroporation. In this study, various media components were investigated to find a medium that allows efficient TGE with CHO cell lines.

**Materials and methods**

CHO-K1 and CHO-T suspension cell lines were investigated regarding growth and TGE in different medium variants. For determination of transfection efficiency, cells were analysed via flow cytometry 48 h after transfection with a GFP expression plasmid, using linear PEI as transfection reagent. Recombinant monoclonal antibody expression in batch processes was monitored by HPLC after cotransfection of cells with two plasmids carrying genes for IgG heavy and IgG light chain expression, respectively.

**Results**

While ammonium iron(III) citrate and iron(III) chloride have been well-known for their inhibiting effect [1], this study revealed a similar impact of iron(II) sulfate heptahydrate and ammonium iron(III) sulfate dodecahydrate on transfection. Iron(III) sulfate heptahydrate completely prevented transfection, whereas ammonium iron (II) sulfate dodecahydrate reduced efficiency by 40-60 % (Fig. 1). Aurintricarboxylic acid (endonuclease inhibitor; enhancer used in e.g. salivary gland transfection) and polyvinylpyrrolidone (polymer; beneficial in electroporation) were both found to negatively impact PEI-mediated transfection of CHO cells, while another tested polymer enhanced growth as well as transfection efficiency. The use of a strong chelator led to a high transfection efficiency, but impaired cell growth. Based on the results of the independent substance testings, the medium formulation was modified by the addition of a weak chelator and further components including vitamins. Different osmolalities between 280 mOsmol/kg and 340 mOsmol/kg were tested for the final formulation, but no major impact was seen neither on transfection efficiency nor on viability 2 days post-transfection.

The final CHO TF medium formulation supported high cell growth of finally tested CHO cell lines 2 and 3 with peak viable cell densities above 10⋅10^6^ cells/ml in batch cultivations with an overall cultivation time of 7-8 days (Fig. 1). Transfection efficiency was above 90 % at 2-4 d post-transfection during transient GFP expression. Growth performance during transient mAb expression was similar with final mAb titers ranging from 70 mg/L reached with CHO cell line 2 and 110 mg/L achieved with CHO cell line 3.

**Conclusions**

As components such as ammonium iron(III) citrate, iron(III) chloride, iron(II) sulfate heptahydrate and ammonium iron(III) sulfate dodecahydrate reduce or completely prevent transfection, iron composition requires careful optimisation to support high transfection efficiency and sufficient growth of CHO cell lines. Similarly, composition and concentration of polymers and chelators have to be tested carefully in order to balance out opposing effects on transfection and cell growth. Finally, it was possible to succesfully develop a high performing transfection medium, whereby it was possible to counterbalance some contrary effects of the presented substances.

Further improvements of the process might be achieved by adapting the protocol, as the results shown are based on a simple pre-complexing of DNA-PEI. Moreover, product yields could potentially be increased by using feeds, temperature shifts or commonly used enhancers (e.g. valproic acids).

**References**

1. Eberhardy SR, Radzniak L, Liu Zhong: **Iron (III) citrate inhibits polyethylenimine-mediated transient transfection of Chinese hamster ovary cells in serum-free medium.** *Cytotechnology* 2009, **60(1-3)**:1-9.

**Fig. 1 (abstract P-154).**
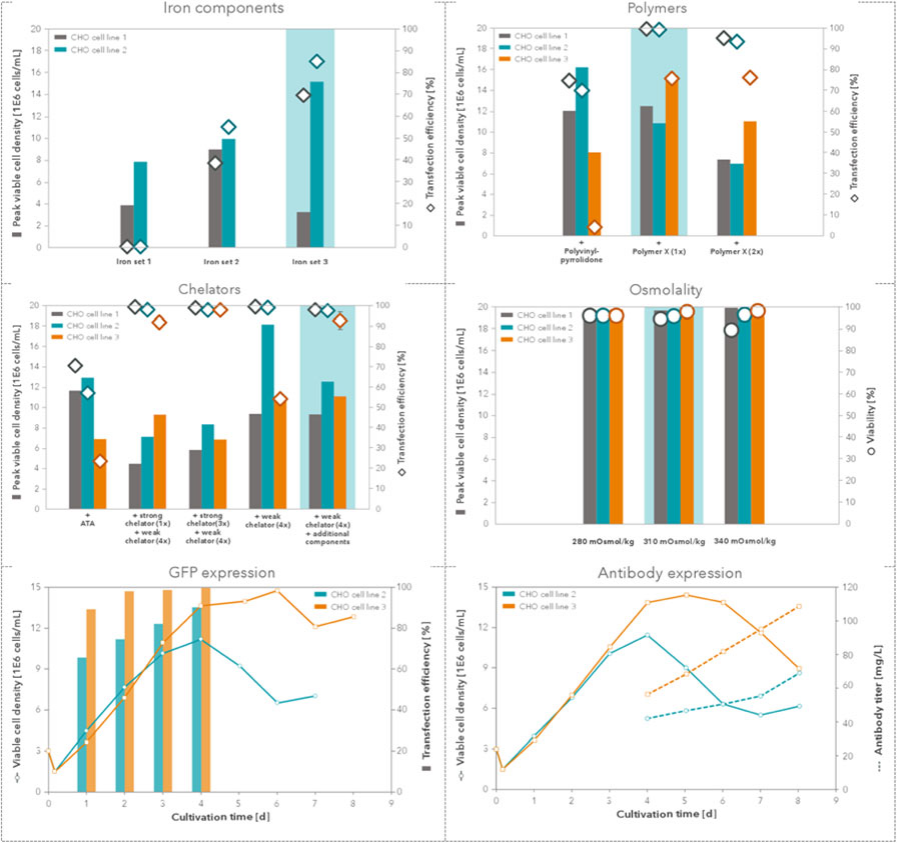
Impact of iron components, polymers, chelators and osmolality on transfection efficiency, growth and transient protein expression (GFP and monoclonal antibody) of different CHO cell lines in final CHO TF medium formulation. Blue highlighted substances, mixtures of substances or osmolality represent the best performing one chosen for final medium formulation

## P-161 Bioreactor scaling thought new – from 5 to 2000 L with utility functions

### Sebastian Ruhl^1^, Adrian Stacey^2^, Ellen Lam^3^, Cécile Villemant^2^, Anilkumar Rathod^4^, Anton Stefan^5^, Christian Zahnow^1^, Gregory Bremer^3^, Andrew Tait^2^, Jens Matuszczyk^1^, Ute Husemann^1^, Gerhard Greller^1^

#### ^1^Sartorius Stedim Biotech, Göttingen, Germany; ^2^Sartorius Stedim Biotech, Royston, UK; ^3^Sartorius Stedim North America, Bohemia, NY, USA; ^4^Sartorius Stedim India, Bangalore, India; ^5^Sartorius Stedim Cellca, Laupheim, Germany

##### **Correspondence:** Sebastian Ruhl (sebastian.ruhl@sartorius-stedim.com)

**Introduction**

Scaling of a cell culture process is an essential part in its development. In a typical approach scaling [1] is performed by keeping a (critical) process parameter constant throughout the complete bioreactor range. This can lead to non-beneficial results either on the high or the low end of the range. For instance, the specific power input [P/V] of 30 W/m^3^ might result in a good agitation in production scale whereas it leads to a non-turbulent mixing behavior in process development scale. To overcome this issue a new approach for an easy scaling procedure was developed. This “Utility Function” approach for agitation scaling is based on individual functions with a value-based mapping independent of bioreactor scale.

**Material and methods**

Process insight information (established either from DoE process investigation or existing experience with a process platform) is directly formalized into a set of mappings which transform bioprocess values into perceived benefits (0 to 1). At each bioreactor scale, parameters (e.g. stirring and gassing) are then chosen to maximize the product of resultant Utility Functions.

The model CHO fed-batch process in this trial comprised a CHO DG44 cell line that was transfected to produce a humanized antibody IgG1. A chemically defined media system was used. The process, including cell line, medium and feeding strategy was designed and developed by Sartorius Stedim Cellca.

The aim of the gassing scale-up was to achieve similar cell densities when the addition of pure oxygen starts. For all Flexsafe STR® bags oxygen was sparged via the micro sparger part of the combi sparger. All other systems used a ring sparger with holes face up.

The initial air flow rate was set to an oxygen transfer rate (k_L_a) of 8 1/h at the corresponding agitation rate and volume. All process engineering characterization parameters were determined according to DECHEMA guidelines [2].

With the use of the Utility Functions the discrete agitation rate was determined (Table 1).

**Results and Discussion**

The Utility Functions led to discrete agitation rates where not only homogeneous mixing but also a turbulent flow pattern and a suitable specific power input was guaranteed. The initial gassing rate of air supplied enough oxygen for 5 x 10^6^ cells/mL in all bioreactors.

Due to the used scaling methods the growth patterns in all bioreactor scales were comparable. Peak viable cell densities (VCD) of 20 – 26 x 10^6^ cells/mL were achieved and viability at the point of harvest was above 80 % in all scales.

The final product concentration was in an acceptable range of 2.9 – 3.6 g/L. Product quality attributes show comparability over the complete bioreactor range (Fig. 1).

The harvest criteria of 12 days gave a combination of viability and product concentration that made it easy to process the cell broth during cell removal and other downstream steps.

**Conclusions**

The process implementation of the CHO production System – Expressing mAb 2 was successfully performed with the use of Utility Functions. Cell growth, productivity and product quality is comparable over the complete bioreactor range.

**References**

1. Tai M., et al.: **Efficient high-throughput biological process characterization**. (2015) Biotechnology Progress DOI 10.1002/btpr.2142.

2. Zahnow C., et al.: **Scale-up of a stirred single-use bioreactor family.** (2017) Poster ESACT Meeting.

**Table 1 (abstract P-161).** Results of the scaling strategy

Ratio	UniVessel® 5 L Glass	STR® 50	STR® 200	STR® 2000
Agitation rate [rpm]	334	162	121	70
Initial gassing rate [Lpm]	0.15	2.75	7.0	73.2

**Fig. 1 (abstract P-161).**
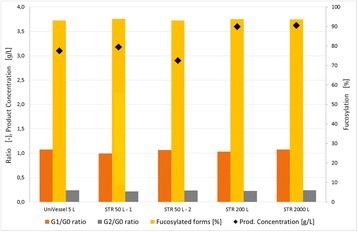
Product analysis from STR® and UniVessel® Glass (performed by BioOutsource)

## P-174 Use of an antioxidant to improve monoclonal antibody production and quality in CHO cells

### Tae Kwang Ha^1^, Anders Holmgaard Hansen^1^, Stefan Kol^1^, Helene Faustrup Kildegaard^1^, Gyun Min Lee^1,2^

#### ^1^The Novo Nordisk Foundation Center for Biosustainability, Technical University of Denmark, Kgs. Lyngby, Denmark; ^2^Department of Biological Sciences, KAIST, Daejeon, Republic of Korea

##### **Correspondence:** Helene Faustrup Kildegaard (hef@biosustain.dtu.dk); Gyun Min Lee (gyunminlee@kaist.ac.kr)

**Background**

Endoplasmic reticulum (ER), the central part of the secretory pathways in eukaryotic cells, is responsible for controlling the quality of secreted and resident proteins through the regulation of protein translocation, protein folding, and early post-translational modifications [1]. A number of physiological conditions such as oxidative stress, hypoglycemia, acidosis, and thermal instability can disturb the ER functions, which triggers ER stress [2]. Prolonged ER stress induces apoptotic cell death [3].

Oxidative stress that naturally accumulates in the ER as a result of mitochondrial energy metabolism and protein synthesis can disturb the ER function [4]. Because ER has a responsibility on the protein synthesis and quality control of the secreted proteins, ER homeostasis has to be well maintained. When H_2_O_2_, an oxidative stress inducer, was added to recombinant Chinese hamster ovary (rCHO) cell cultures, it reduced cell growth, monoclonal antibody (mAb) production, and galactosylated form of mAb in a dose-dependent manner. Antioxidants can reduce the oxidative stress level and suppress the apoptotic cell death by scavenging oxygen free radicals, inhibiting chain reaction of oxidation, and detoxifying peroxide [5]. However, despite the importance of mass production of mAbs, studies on the effect of antioxidants on the production and quality of mAbs in rCHO cell cultures have not been fully substantiated.

**Materials and methods**

To find a more effective antioxidant in rCHO cell cultures, six different antioxidants including baicalein, which have used widely in mammalian cell cultures, were evaluated as chemical supplements with two different rCHO cell lines producing the same mAb in 6-well plates. Then, batch and fed-batch cultures were performed in shake flasks with the supplementation of baicalein, which showed the best effect on culture performance among the 6 antioxidants. The reactive oxygen species (ROS) and ER stress levels were measured to study the effect of baicalein on mAb production and quality.

**Results**

Among these antioxidants, baicalein showed the best mAb production performance. Addition of baicalein significantly reduced the expression level of BiP and CHOP along with reduced ROS level, suggesting oxidative stress accumulated in the cells can be relieved using baicalein. As a result, addition of baicalein in batch cultures resulted in 1.7 - 1.8-fold increase in the maximum mAb concentration (MMC), while maintaining the galactosylation of mAb (Fig. 1 and Table 1). Likewise, addition of baicalein in fed-batch culture resulted in 1.6-fold increase in the MMC while maintaining the galactosylation of mAb.

**Conclusions**

Oxidative stress negatively affected the production and galactosylation of mAb in rCHO cell cultures. Among the various antioxidants tested in this study, baicalein showed the best mAb production performance in both batch and fed-batch cultures of rCHO cells. Baicalein addition significantly enhanced mAb production while maintaining galactosylated forms of mAb. Thus, baicalein is an effective antioxidant for use in rCHO cell cultures for improved mAb production.

**Acknowledgements**

This research was supported by Danish Council for Independent Research – Technology and Production Sciences (FTP), The Novo Nordisk Foundation, and the Ministry of Science, ICT and Future Planning for Basic Core Technology Development Program for the Oceans and the Polar Regions of the NRF (NRF-2016M1A5A1901813).

**References**

1. Ruggiano A, Foresti O, Carvalho P: Quality control: ER-associated degradation: protein quality control and beyond. J Cell Biol 2014, 204(6), 869-879.

2. Schönthal AH: Pharmacological targeting of endoplasmic reticulum stress signaling in cancer. Biochem Pharmacol 2012, 85(5), 653-666.

3. Urra H, Dufey E, Lisbona F, Rojas-Rivera D, Hetz C: When ER stress reaches a dead end. Biochim Biophys Acta 2013, 1833(12), 3507-3517.

4. Birben E, Sahiner UM, Sackesen C, Erzurum S, et al: Oxidative stress and antioxidant defense. World Allergy Organ J 2012, 5(1), 9-19.

5. Shimazaki H, Watanabe K, Veeraveedu PT, Harima M et al: The antioxidant edaravone attenuates ER-stress-mediated cardiac apoptosis and dysfunction in rats with autoimmune myocarditis. Free Radic Res 2010, 44(9), 1082-1090.

**Fig. 1 (abstract P-174).**
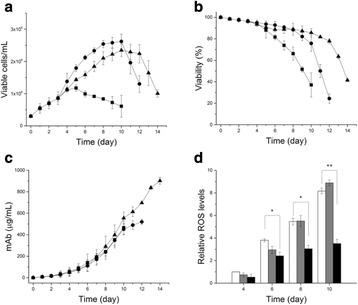
Profiles of **a** cell growth, **b** viability, **c** mAb concentration, and **d** ROS level of a CS13-1.00 cell line producing mAb during shake flask cultures with baicalein addition. (**a** – **c**) No baicalein (closed circle), DMSO (closed square), and 100 μM baicalein (closed triangle). **d** No baicalein (white), DMSO (gray), and 100 μM baicalein (black). The error bars represent the standard deviations calculated from three independent experiments. * *P* < 0.05, ** *P* < 0.01

**Table 1 (abstract P-174).** The μ, MVCC, qmAb, and MMC with or without baicalein addition during batch cultures. No baicalein (C), DMSO (C+), and 100 μM baicalein (T)

	μ (day^-1^)	MVCC (x 10^6^ cells/mL)	q_mAb_ (pg/cell/day)	MMC (μg/mL)
C	0.29 ± 0.01	2.62 ± 0.23	17.49 ± 1.37	520.1 ± 9.5
C+	0.18 ± 0.02	1.18 ± 0.19	25.05 ± 2.23	465.5 ± 93.9
T	0.21 ± 0.01	2.35 ± 0.06	22.13 ± 2.20	901.3 ± 112.9

## P-175 Model-assisted cell culture control – unstructured, unsegregated models as a key element for adaptive seed train and fed-batch optimization

### Tanja Hernández Rodríguez^1^, Susan Krull^1^, Volker C. Hass^3^, Johannes Möller^2^, Ralf Pörtner^2^, Björn Frahm^1^

#### ^1^Biotechnology & Bioprocess Engineering, Ostwestfalen-Lippe University of Applied Sciences, Lemgo, D-32657, Germany; ^2^Institute of Bioprocess and Biosystems Engineering, Hamburg University of Technology, Hamburg, D-21073, Germany; ^3^Hochschule Furtwangen University, Faculty of Medical and Life Sciences, Villingen-Schwenningen, D-78054, Germany

##### **Correspondence:** Björn Frahm (bjoern.frahm@hs-owl.de)

**Background**

The production of many biopharmaceuticals (e.g. antibodies & proteins for diagnostic and therapeutic purposes) requires the cultivation of mammalian cell lines, which is demanding with respect to various aspects such as complex cell metabolism, variabilities in cell behavior, scale dependencies, influences of changes in cultivation conditions, medium composition etc. Although an increasing number of measurement parameters is available, only a part of them is routinely utilized in industrial cell culture processes and their corresponding seed trains. Nevertheless, the data base grows, statistical investigation of data gains importance and process data are more easily accessible in the context of industry 4.0.

Cell cultivation has to consider these complex requirements, e.g. for fed-batch control and seed train design. Furthermore, cultivation strategies have to be adapted to new products, cell lines and clones as well as to different production plants when transferring processes.

One approach to encounter the variabilities and to include actual information from the process and from data analysis is adaptive model-assisted control [1].

**Methods**

Two software tools enabling adaptive model-assisted control applying unstructured, unsegregated models have been developed and implemented using MATLAB^©^, WinErs and Fortran, one tool for fed-batch control and another one for seed train simulation and optimization.

**Results**

One key element of adaptive model-assisted control is the underlying process model. In order to provide an adaptive character, model parameters should be easily identifiable from routine cultivation data, which is available during seed train and fed-batch without additional sophisticated measurements. Therefore, the usage of unstructured, unsegregated models is recommended.

a) Example of an unstructured, unsegregated cell culture model (for adaptive model-assisted control)

One example, describing cell growth, cell death, uptake of substrates and production of metabolites via a first order system of ordinary differential equations and Monod-type kinetics, is shown in Table 1. This mathematical model includes 13 cell specific model parameters [2].

b) Application examples

This contribution illustrates the usage of such a model for two applications: A controller for the calculation of fed-batch feed trajectories and a software tool for seed train design, analysis and optimization. The implementation is shown in each case concerning the structure and the open-loop control sequence in Fig. 1a fed-batch control and Fig. 1b seed train simulation & optimization. Both implementations are divided into two main parts, i) adaptation to cell line and ii) open-loop control sequence.

i) Adaptation to cell line: Based on a corresponding mathematical cell culture model, model parameters must be identified reflecting cell line and cell behaviour.

ii) a) Open-loop control sequence for fed-batch control: Based on model and a priori identified model parameters, the optimization cycle includes getting data from the running cultivation, automated adaption of model parameters, prediction of future process course, computation of optimal feed profiles and realization.

ii) b) Open-loop control sequence for seed train simulation and optimization [3]: Using model, a priori identified model parameters and starting concentration values, the temporal concentration courses can be predicted for the first scale. Subsequently, points in time for passaging and starting values for the next scale can be computed by adding a passaging strategy, seed train conditions and medium concentrations. Prediction for the following scales can be obtained iteratively. Integrating feedback from the process in terms of cultivation data enables increasing prediction accuracy and responding to possible changes in cell behaviour.

**Conclusions**

Process design and optimization, e.g. regarding seed train and fed-batch, is realized by adaptive model-assisted software tools using unstructured, unsegregated models. They enable feedback from the process via routine cultivation data and allow adaptation to diverse circumstances such as different cell lines, products, cultivation conditions, plant configurations etc.

**References**

1. Pörtner R et al.: **Advanced Process and Control Strategies for Bioreactors.** Book chapter, pp. 463-493, *Current Developments in Biotechnology and Bioengineering: Bioprocesses, Bioreactors and Controls*, Elsevier, ISBN 978-0-444-63663-8, 2016.

2. Kern S, Platas Barradas O, Pörtner R, Frahm B: **Model-based strategy for cell culture seed train layout verified at lab scale.** *Cytotechnology*, 2016 Aug;68(4):1019-32. doi: 10.1007/s10616-015-9858-9. Epub 2015 Mar 21.

3. Frahm B: **Seed train optimization for cell culture.** Chapter, *Animal Cell Biotechnology-Methods and Protocols*, 3^rd^ edition, edited by Pörtner R., Springer/Humana Press, ISBN 978-1-62703-732-7, ISBN 978-1-62703-733-3 (eBook), 2014.

**Table 1 (abstract P-175).** Equations of balances and kinetics of an employed process model including ***X***_***v***_ viable cell density, ***X***_***t***_ total cell density, ***μ*** cell-specific growth rate, ***μ***_***d***_ cell-specific death rate, ***t*** time, ***K***_***S***_ and ***k*** Monod kinetic constant and Monod constant for uptake, ***K***_***Lys***_ cell lysis constant, ***q*** cell-specific uptake rate or production rate, respectively, ***Y*** kinetic production constant, ***c*** concentration, ***Glc*** glucose, ***Gln*** glutamine, ***Lac*** lactate, ***Amm*** ammonia, ***F*** feed rate, ***V*** volume

	Balances with fed-batch terms		Kinetics
Biophase	$\frac{{\boldsymbol{d}\boldsymbol{X}}_{\boldsymbol{v}}}{\boldsymbol{d}\boldsymbol{t}}=\left(\boldsymbol{\mu} -{\boldsymbol{\mu}}_{\boldsymbol{d}}\right)\bullet {\boldsymbol{X}}_{\boldsymbol{v}}$ $-\frac{{\boldsymbol{F}}_{\boldsymbol{Glc}}+{\boldsymbol{F}}_{\boldsymbol{Gln}}}{\boldsymbol{V}}\bullet {\boldsymbol{X}}_{\boldsymbol{v}}$ $\frac{{\boldsymbol{dX}}_{\boldsymbol{t}}}{\boldsymbol{dt}}=\boldsymbol{\mu} \bullet {\boldsymbol{X}}_{\boldsymbol{v}}-{\boldsymbol{K}}_{\boldsymbol{Lys}}\bullet \left({\boldsymbol{X}}_{\boldsymbol{t}}-{\boldsymbol{X}}_{\boldsymbol{v}}\right)$ $-\frac{{\boldsymbol{F}}_{\boldsymbol{Glc}}+{\boldsymbol{F}}_{\boldsymbol{Gln}}}{\boldsymbol{V}}\bullet {\boldsymbol{X}}_{\boldsymbol{t}}$	Cell growth & death	$\boldsymbol{\mu} ={\boldsymbol{\mu}}_{\boldsymbol{max}}\bullet \frac{{\boldsymbol{c}}_{\boldsymbol{Glc}}}{{\boldsymbol{c}}_{\boldsymbol{Glc}}+{\boldsymbol{K}}_{\boldsymbol{S},\boldsymbol{Glc}}}\bullet \frac{{\boldsymbol{c}}_{\boldsymbol{Gln}}}{{\boldsymbol{c}}_{\boldsymbol{Gln}}+{\boldsymbol{K}}_{\boldsymbol{S},\boldsymbol{Gln}}}$ ${\boldsymbol{\mu}}_{\boldsymbol{d}}={\boldsymbol{\mu}}_{\boldsymbol{d},\boldsymbol{\min}}+{\boldsymbol{\mu}}_{\boldsymbol{d},\boldsymbol{\max}}\bullet \frac{{\boldsymbol{K}}_{\boldsymbol{S},\boldsymbol{Glc}}}{{\boldsymbol{K}}_{\boldsymbol{S},\boldsymbol{Glc}}+{\boldsymbol{c}}_{\boldsymbol{Glc}}}$
Liquid phase	$\frac{{\boldsymbol{dc}}_{\boldsymbol{Glc}}}{\boldsymbol{dt}}=-{\boldsymbol{q}}_{\boldsymbol{Glc}}\bullet {\boldsymbol{X}}_{\boldsymbol{v}}$ $+\frac{{\boldsymbol{F}}_{\boldsymbol{Glc}}}{\boldsymbol{V}}\bullet {\boldsymbol{c}}_{\boldsymbol{Glc},\boldsymbol{F}}-\frac{{\boldsymbol{F}}_{\boldsymbol{Glc}}+{\boldsymbol{F}}_{\boldsymbol{Gln}}}{\boldsymbol{V}}\bullet {\boldsymbol{c}}_{\boldsymbol{Glc}}$ $\frac{{\boldsymbol{dc}}_{\boldsymbol{Gln}}}{\boldsymbol{dt}}=-{\boldsymbol{q}}_{\boldsymbol{Gln}}\bullet {\boldsymbol{X}}_{\boldsymbol{v}}$ $+\frac{{\boldsymbol{F}}_{\boldsymbol{Gln}}}{\boldsymbol{V}}\bullet {\boldsymbol{c}}_{\boldsymbol{Gln},\boldsymbol{F}}-\frac{{\boldsymbol{F}}_{\boldsymbol{Glc}}+{\boldsymbol{F}}_{\boldsymbol{Gln}}}{\boldsymbol{V}}\bullet {\boldsymbol{c}}_{\boldsymbol{Gln}}$ $\frac{{\boldsymbol{dc}}_{\boldsymbol{Lac}}}{\boldsymbol{dt}}={\boldsymbol{q}}_{\boldsymbol{Lac}}\bullet {\boldsymbol{X}}_{\boldsymbol{v}}$ $-\frac{{\boldsymbol{F}}_{\boldsymbol{Glc}}+{\boldsymbol{F}}_{\boldsymbol{Gln}}}{\boldsymbol{V}}\bullet {\boldsymbol{c}}_{\boldsymbol{Lac}}$ $\frac{{\boldsymbol{dc}}_{\boldsymbol{Amm}}}{\boldsymbol{dt}}={\boldsymbol{q}}_{\boldsymbol{Amm}}\bullet {\boldsymbol{X}}_{\boldsymbol{v}}$ $-\frac{{\boldsymbol{F}}_{\boldsymbol{Glc}}+{\boldsymbol{F}}_{\boldsymbol{Gln}}}{\boldsymbol{V}}\bullet {\boldsymbol{c}}_{\boldsymbol{Amm}}$	Substrate uptake & metabolite production	${\boldsymbol{q}}_{\boldsymbol{Glc}}={\boldsymbol{q}}_{\boldsymbol{Glc},\boldsymbol{\max}}\bullet \frac{{\boldsymbol{c}}_{\boldsymbol{Glc}}}{{\boldsymbol{c}}_{\boldsymbol{Glc}}+{\boldsymbol{k}}_{\boldsymbol{Glc}}}\bullet \left(\frac{\boldsymbol{\mu}}{\boldsymbol{\mu} +{\boldsymbol{\mu}}_{\boldsymbol{max}}}+\mathbf{0.5}\right)$ ${\boldsymbol{q}}_{\boldsymbol{Gln}}={\boldsymbol{q}}_{\boldsymbol{Gln},\boldsymbol{\max}}\bullet \frac{{\boldsymbol{c}}_{\boldsymbol{Gln}}}{{\boldsymbol{c}}_{\boldsymbol{Gln}}+{\boldsymbol{k}}_{\boldsymbol{Gln}}}$ ${\boldsymbol{q}}_{\boldsymbol{Lac}}={\boldsymbol{Y}}_{\boldsymbol{Lac}/\boldsymbol{Glc}}\bullet \frac{{\boldsymbol{c}}_{\boldsymbol{Glc}}}{{\boldsymbol{c}}_{\boldsymbol{Lac}}}\bullet {\boldsymbol{q}}_{\boldsymbol{Glc}}-{\boldsymbol{q}}_{\boldsymbol{Lac},\boldsymbol{uptake}}$ ***if c***_***Glc***_ ≥ **0.5** ***mM : q***_***Lac,uptake***_ = **0** ***if c***_***Glc***_ < **0.5** ***mM : q***_***Lac,uptake***_ = ***q***_***Lac,uptake,max***_ ***q***_***Amm***_ = ***Y***_***Amm***/***Gln***_ ∙ ***q***_***Gln***_

**Fig. 1 (abstract P-175).**
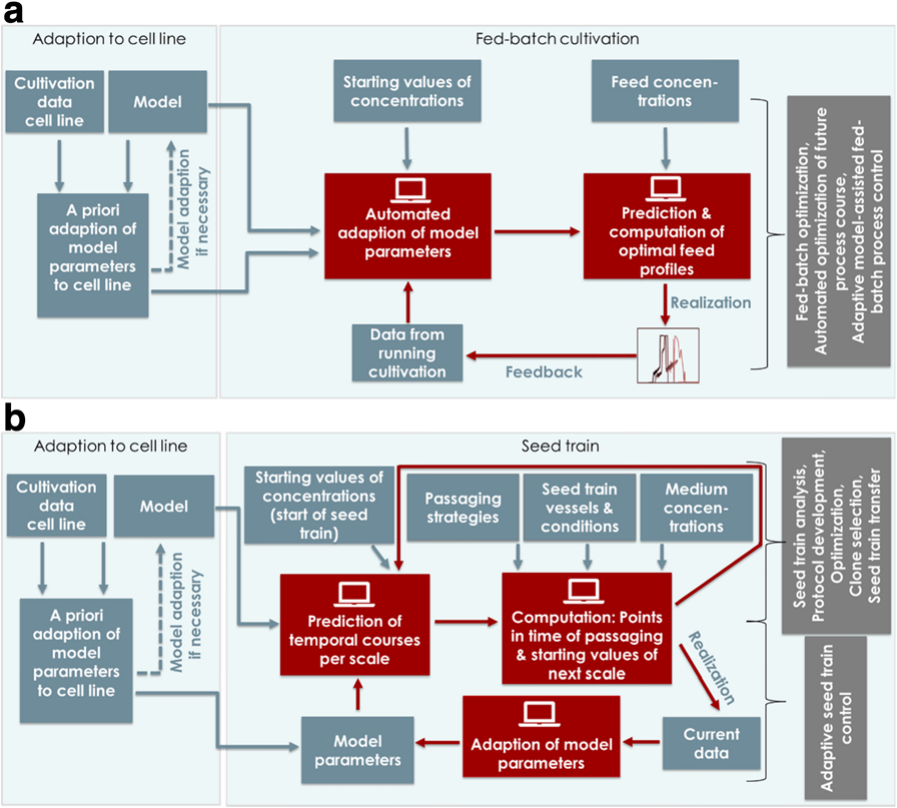
Scheme of adaptive model-assisted cell culture control. Example for **a** fed-batch control and **b** seed train simulation and optimization

## P-183 Creating a suitable microenvironment for growing human primary T cells to high cell densities

### Melanie Werner^1,2^, Patrick Kaiser^1^, Michael Hader^1^, Valérie Jérôme^1^, Ruth Freitag^1^

#### ^1^Chair for Process Biotechnology, University of Bayreuth, Bayreuth, 95447, Germany; ^2^Present address: GSK Vaccines GmbH, Marburg, Germany, 35041

##### **Correspondence:** Ruth Freitag (ruth.freitag@uni-bayreuth.de)

**Background**

*Ex vivo* expansion of human primary T cells is of considerable scientific and medical interest, e.g. as for T cell therapy. Currently, this is time consuming and requires the addition of massive amounts of stimuli. Recently, we showed that human leukemia T cells (Jurkat cells) can be expanded to tissue like cell densities (>10^8^ cells mL_capsule_^−1^) in polyelectrolyte capsules. Significant advantages, such as great mechanical stability, good biocompatibility and good mass transfer properties characterized these capsules based on sodium cellulose sulfate/poly(diallyldimethyl) ammonium chloride (SCS/PDADMAC) [1, 2]. Here, we present the possibility to cultivate human T cells, freshly isolated from blood, to high densities in similar semipermeable polyelectrolyte microcapsules within less than 10 days.

**Materials and methods**

Cells were encapsulated in semipermeable SCS/PDADMAC polyelectrolyte microcapsules or confined in 1.5% alginate/poly-l-lysine (PLL) beads, a standard approach for cell immobilization. The permeability of the microcapsules was estimated using dextran-based molecular weight standards (10 and 20 kDa) and vitamin B12 (1.6 kDa). Gentle digestion with endocellulase allows an easy release of the cells out of the capsules. Cell growth, cytokines production and phenotype were measured in non-encapsulated and encapsulated cells grown under standard culture conditions. Moreover, we analyzed the interplay between the secreted cytokines and the SCS within the capsules and its putative influence on cell growth.

**Results**

Cells mixed in the cellulose sulfate solution under physiological conditions can be safely trapped within a liquid core during capsule formation. Encapsulated cells can reached cell densities ≤ 40 x 10^6^ cells mL_capsule_^-1^, whereas cells confined in alginate/PLL beads and non-encapsulated ones reached 11.3 x 10^6^ cells mL_bead_^-1^ and 2.4 x 10^6^ cells mL, respectively. One major advantage of these polyelectrolyte microcapsules (<1 mm) is the low MWCO (<10 kDa) (Fig. 1a-b). This restricted permeability allows for a conditioning of the capsule core by autocrine factors, which in turn permits the use of basal cell culture medium instead of expensive T cell specialized media, hence does not necessitate high amounts of rhIL-2 and reduces the cultivation costs. Moreover, co-encapsulation of rhIL-2 had a beneficial effect on the growth kinetics in most cases (Fig. 1c). Some evidence is presented that the SCS used to form the polyelectrolyte microcapsules, specifically adsorbs IL-2 (Table 1) – A cytokine which provides an essential signal for T-cell proliferation and differentiation [3]. Therefore, we postulate that the SCS used for encapsulation has biomimetic properties, creating an artificial extracellular matrix mimicking heparin sulfate which in turn positively affect T cell proliferation via trans-presentation of IL-2 (Fig. 1d) [4].

**Conclusions**

Primary T lymphocytes can be expanded under appropriate conditions outside the body. In the latter, T cells grow/expand in specific environments where the cells are tightly packed, leading to multiple cell–cell contacts and manifold interactions with the extracellular matrix. *Ex vivo* suspension cultures of diluted cells cannot provide such a microenvironment. In the microcapsules-based cultivation system presented, the cells are suspended in a viscous SCS-solution. The low molecular weight cut off of the surrounding polyelectrolyte membrane assures that typical signaling molecules produced by the cells are retained thus facilitates the “conditioning” of the cellular microenvironment, while nutrients and metabolites can pass. Expensive additives, such as interleukin-2 (IL-2), can be co-encapsulated. Expansion then no longer requires specialized T-cell media. Moreover, the SCS seems to have biomimetic properties, representing an artificial extracellular matrix mimicking heparin sulfate. We consider that the described method may be an appropriate alternative to expand T cells while creating a local microenvironment mimicking *in vivo* conditions.

**Acknowledgements**

This research was supported by the DFG (Deutsche Forschungsgemeinschaft), grants BU 461/26-1 and FR 830/14-1.

**References**

1. Werner, M.; Schmoldt, D.; Hilbrig, F.; Jérôme, V.; Raup, A.; Zambrano, K.; Hübner, H.; Buchholz, R.; Freitag, R.: **High Cell Density Cultivation of Human Leukemia T Cells (Jurkat Cells) in Semipermeable Polyelectrolyte Microcapsules.** *Eng. Life Sci.* 2015, **15**:357-367.

2. Kaiser, P.; Werner, M.; Jérôme, V.; Hübner, H.; Buchholz, R.; Freitag, R., **Cell retention by encapsulation for the cultivation of Jurkat cells in fixed and fluidized bed reactors.** *Biotechnol. Bioeng*. 2014, **111**, 2571-2579.

3. Boyman, O.; Sprent, J., **The role of interleukin-2 during homeostasis and activation of the immune system**. *Nat. Rev. Immunol.* 2012, **12**, 180-190.

4. Jérôme, V.; Werner, M.; Kaiser, P.; Freitag, R.: **Creating a Biomimetic Microenvironment for the Ex Vivo Expansion of Primary Human.** *Macromol. Biosci.* 2017, DOI: 10.1002/mabi.201700091.

**Fig. 1 (abstract P-183).**
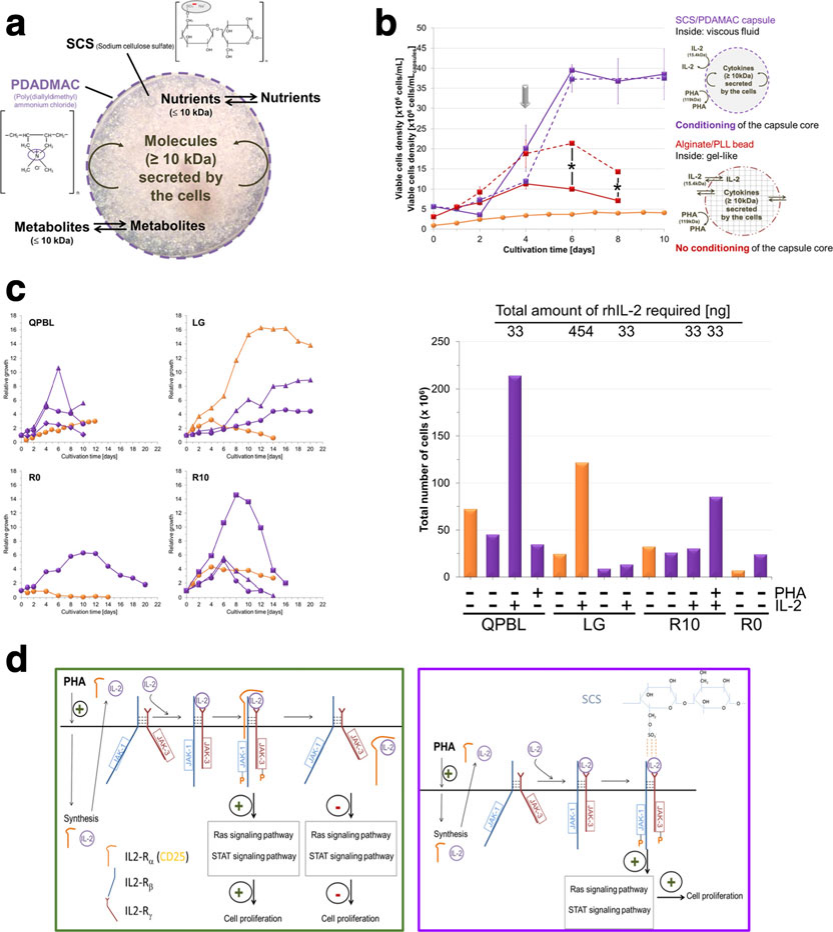
Influence of the capsule material on the expansion of human primary T lymphocytes. **a**: Polyelectrolyte capsule (SCS/PDAMAC). **b**: Growth of T lymphocytes encapsulated in SCS/PDADMAC versus alginate/PLL. Growth kinetics of activated peripheral blood T cells, non-encapsulated () and encapsulated in SCS/PDADMAC () or alginate/Poly-L-Lysine (). The cells were cultivated in fresh (solid lines) or conditioned (dotted lines) specialized T cells medium containing phytohemaglutinin (PHA) as activating substance. Starting on day 4, 35% of the growth medium was exchanged every second day. Data represent mean ± SD (n=3). D0: day of encapsulation. Statistical significance indicated by * (p < 0.05). **c**: Comparison of the relative expansion of T lymphocytes under various culture conditions. Left: IL-2 (rhIL-2, 11 ng mL^−1^) and PHA (2 μg mL^−1^) were added either alone or in combination to the cell culture medium of the non-encapsulated cells or co-encapsulated with the cells. No supplements (,), PHA (); IL-2 (,); IL-2/PHA ().QPBL, LG: activation media containing PHA. R10, R0: RPMI1640-based media supplemented or not 10 % FCS. Culture volume was 30 mL (QPBL) or 15 mL (LG, R10 and R0), total capsule volume was always 3 mL. D0: day of encapsulation. Right: Total number of cells produced in non-encapsulated (orange bars) and encapsulated (violet bars) systems and total amounts of additives (rhIL-2, PHA) required. **d**: Schematic representation of the putative influence of the capsule microenvironment on the T cells signalling after PHA activation. Left: Non-encapsulated T lymphocytes, right: T lymphocytes in SCS/PDADMAC capsules

**Table 1 (abstract P-183).** Analysis of rhIL-2, rhIFNγ, and sCD25 recovery after incubation with SCS

	Recovery [%]		
Time of incubation [h]	rhIL-2	rhIFNγ	sCD25
0	100 ± 5.3	117 ± 3.8	103.7 ± 1.3
3	74.0 ± 4	72.6 ± 1.0	N.D.
6	56.7 ± 0.3	106.5 ± 4.9	N.D.
21	10.9 ± 0.3	82.1 ± 2.1	93.6 ± 10.2

Prior to ELISA, the various proteins were incubated at 37°C in SCS prepared as for encapsulation. As control, the SCS was replaced by PBS. Shown are mean values ± SD, n = 3

## P-190 The new age of digital biomanufacturing

### William Whitford^1^, Daniel Horbelt^2^

#### ^1^GE Healthcare Bio-Sciences AB, Uppsala, Sweden, 75184; ^2^Insilico Biotechnology AG, Stuttgart, Germany, 70563

##### **Correspondence:** William Whitford (bill.whitford@ge.com); Daniel Horbelt (daniel.horbelt@insilico-biotechnology.com)

**Background**

Digital manufacturing (DM) is heightening the productivity and robustness of existing processes and facilities. It also enables the efficient development of previously unmanageable products or processes and provided the basis for a wave of innovations. DM is a resident and on-line source of continuous optimization of process performance. It relies upon the comprehensive, real-time interfacing of both human and machine sourced information through one centralized system. More than legacy Distributed Control System (DCS) and supervisory control and data acquisition (SCADA), it is an integral interconnection of real-time access to divergent sources of information. As such, it can promise deep analysis and predictions leading to shortened product cycle and advanced process control. This comprehensive analysis is extending beyond operations performance data from the production floor to data driving such activities as raw materials security of supply (SoS) and business continuity management systems (BCMS).

**Materials and methods**

Digital Biomanufacturing (DB) can be viewed as yet another, larger, embodiment of digital biotechnology. DB is similar to digital manufacturing in that it promotes innovations in the manufacturing of biologicals by using such things as computer aided design, manufacture, verification and deep process analysis using software sensors (Fig. 1). However, the fact that there are living components (cells) involved in the processes puts a distinctly different flavor to the systems employed. It is desirable to use a distinct term here to distinguish it because, as in the terms bioproduction and biopharmacology, DB addresses many unique aspects of biologically-based activities.

**Results**

The reasons why the biotech and biopharma industry lags behind other sectors such as the automotive regarding the transformation to digital manufacturing are (i) the complexity and dissipative nature of biological systems, (ii) distributed heterogeneous data and (iii) limited at-line or on-line data sources. However, the costs of genomic sequencing, omics data generation, and computing resources are decreasing rapidly, and at the same time process analytical technologies, computational power and predictive modeling as well as data management infrastructures are greatly improving (Table 1). By removing roadblocks that used to limit approaches, these changes have paved the way to transforming the bioeconomy into an industry that is based on digital knowledge.

Such new and optimized manufacturing technologies as continuous biomanufacturing and 3D bioprinting can actually demand the interfacing of many sources of information, deep data analysis including software sensors for metabolic fluxes, and model-based predictions of digital biomanufacturing. The application of predictive models for bioprocess optimization greatly improves established platforms and finally leads to a massively increased mechanistic process understanding.

**Conclusions**

Four essential benefits result from the increased bioprocess understanding, development, and control of DB. First, personnel are relieved of many manual and repetitive tasks. Second, strategic planning and operational efficiency are improved. Third, we see real-time optimization of end-to-end manufacturing based on such high-value criteria as projected product quality and profitability. Fourth, it enables previously unmanageable operations and creates innovative solutions.

**References**

Whitford W: **The Era of Digital Biomanufacturing.** *BioProcess International* 2017 **15(3)**:12–18

**Fig. 1 (abstract P-190).**
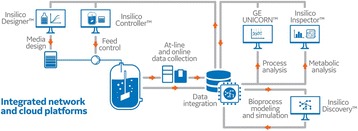
Intelligent software applications support digital biomanufacturing process development and control. • Databases using data collected online, at-line, and offline from bioprocesses operating worldwide. • Process data are used to generate metabolic network models that represent a specific host cell line in a bioprocess. • Model-based computational simulations improve process understanding and reduce experimental efforts for media design, clone selection, and metabolic engineering. • Automated data import and processing allow for a streamlined and standardized metabolic process analysis. • Identification of critical metabolic parameters is used for proactive steering and control of production processes

**Table 1 (abstract P-190).** Enablers of digital biomanufacturing

Information technology	Biotechnology
Advanced interfacing, process analytics, and control algorithms	Advanced process engineering, analytics equipment, at-line assay
Big Data: effective management of large and complex data sets	New in-line (SU) probes and automated at-line (cell-free) sampling
Adaptive fuzzy expert system now employ artificial intelligence	Rapid at-line 2D • fluorescence; near/mid-IR; Raman spectroscopy
Flexible and affordable data storage and cloud hosting	Near-real time concentration measurements using CFCA methods
Laboratory information management systems (LIMS)	At-line surface plasmon resonance specificity, kinetics, and affinity
Industrial internet of things (IIoT) and cloud computing	Intriguing microfluidic platform-based at-line cell-condition analytics
FDA-regulated suitable software providing traceability/backup	Near real-time product CQA assessment and data accession
Analytical QbD (AQbD) or QbD as applied to analytical methods	

## P-192 Monitoring between-batch behavior of real-time adjusted cell-culture parameters

### Xavier Lories, Jean-François Michiels

#### Arlenda, Mont-Saint-Guibert, 1435, Belgium

##### **Correspondence:** Xavier Lories (xavier.lories@arlenda.com)

**Background**

Cell-culture parameters (CCP), such as pH, may be continuously measured online and subject to real-time automated adjustment (e.g. automated addition of a base to prevent the pH to drop too low). This is an efficient method to maintain the parameter within specified limits. This type of control constraint the variability within the pre-defined limits and does not provide any information on the between-batch variability of the process.

Online measurements of CCP provide time-dependent curves presenting one or more transitions. Different types of transition can be observed:

- The process can shift from a state in which adjustment is needed to keep the CCP in range to a state in which it is not. Typically, the CCP drifts away from a limit.
- The process shifts from a state in which adjustment is not needed to one in which it is. For instance, a drifting CCP reaches the lower or upper limit of the accepted range.

The timepoints at which those transitions take place are here called changepoints. Those are aspects of the process and, as such, should be controlled.

In the multiple changepoints cases, the approach allows the early termination of runs showing very early or very late first changepoint.

**Material and methods**

The identification of the changepoints position is based on simple rules rather than complex statistical modeling to keep the identification methodology simple. Once the changepoint are identified, a multivariate Bayesian model is adjusted on the appropriately transformed data. Prediction regions are obtained and used as control limits [1].

**Results**

Results obtained for a 2-changepoint case are shown on Fig. 1. Points on the right-hand graph represent new batches. The red triangle represents a failed batch.

It appears that the control strategy fails to identify the failed batch. Two reasons can be considered:

- The limits of the prediction region have been established based on 9 points, such a small sample size is likely to be insufficient for the definition of such a control chart.
- The tested batches were produced out of set point. A control chart should be used on a stable process, ran in the same conditions, in order to be really relevant. This work was based on available historical data, which is never an ideal situation.

**Conclusions**

The suggested strategy offers a simple approach to the monitoring of between-batch behavior for cell-culture. Once the limits have been defined, the approach is quite straightforward and usable by non-statistician. However, such strategy, as any other of this type, must be based on a sufficient number of batches for the definition of the control limits in order to have a good estimation of the batch-to batch variability.

**References**

1. Krishnamoorthy K, Mathew T, **The Multivariate Normal Distribution**. In Statistical Tolerance Regions. Theory, Applications, and Computation, 1^st^ edition, Edited by Balding D, Cressie N, Fitzmaurice G, Johnstone I, Molenberghs G, Scott D. Hoboken: John Wiley & Sons, Inc; 2009 225-246.

**Fig. 1 (abstract P-192).**
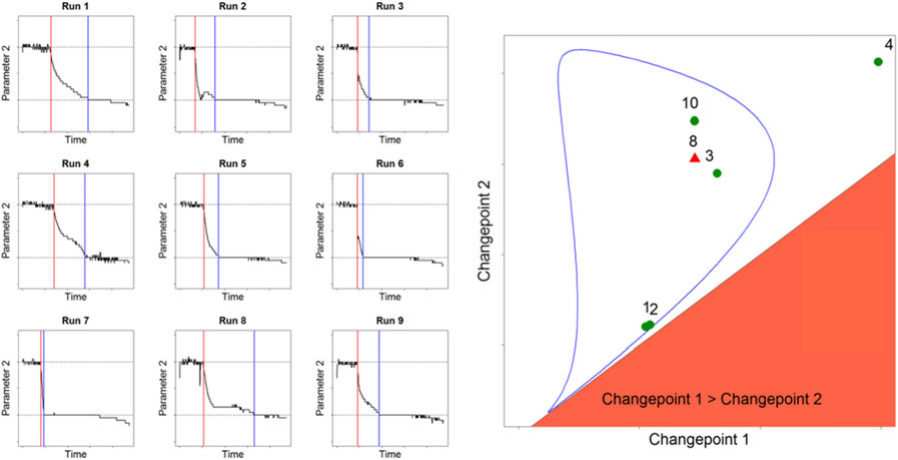
Results for a 2-changepoint situation. Left: identified changepoints (red: first changepoint, blue: second changepoint). Right: prediction region around new batches

## P-204 Optimization of the production process for a vlp-based rabies vaccine

### Diego Fontana^1,4^, Federico Marsili^2^, Sebastián Antuña^3^, Marina Etcheverrigaray^4^, Ricardo Kratje^4^, Claudio Prieto^2,3^

#### ^1^Biotechnological Development Laboratory, FBCB, Universidad Nacional Del Litoral, Conicet, Santa Fe, C.C. 242. (S3000ZAA), Argentina; ^2^Biotechnological Development Laboratory, FBCB, Universidad Nacional Del Litoral, Santa Fe, C.C. 242. (S3000ZAA), Argentina; ^3^Cell Culture Laboratory, FBCB, Universidad Nacional Del Litoral, Santa Fe, C.C. 242. (S3000ZAA), Argentina; ^4^Cell Culture Laboratory, FBCB, Universidad Nacional Del Litoral, Conicet, Santa Fe, C.C. 242. (S3000ZAA), Argentina.

##### **Correspondence:** Diego Fontana (dfontana@fbcb.unl.edu.ar)

**Background**

Rabies is a zoonotic viral disease with a mortality close to 100% [1]. As there is not an efficacious treatment available, post-exposure vaccination is recommended for individuals in contact with the virus. On the other hand, the most common source of virus transmission is saliva of infected animals, mostly dogs, whereby mass vaccination of pets is the most cost‑effective way to reduce human infections. In this context, availability of both human and veterinary vaccines is critical [2,3].

Our group had previously developed an effective VLP-based rabies vaccine candidate produced in high density HEK293 cell cultures with serum free medium (SFM) [4,5]. One of the aims in vaccine production process is the achievement of a good productivity with a low cost per dose, mainly in the case of vaccines for animal use in which case the SFM is one of the principal expenses. In this work, we show the adaptation of the producer clone to a non-expensive in-house developed culture medium, in order to reduce the global cost of the process and therefore the price per dose.

**Experimental approach**

First, we compared a direct and a sequential adaptation protocol of our HEK293 RV‑VLPs producer clone, from 100% of the commercial SFM (EX-CELL293, SAFC) to a new formulation with only 50% of the SFM and a minimum essential medium (P2G), developed in our laboratory specifically for RV-VLPs production. This new formulation was called RVPM (*Rabies Vaccine Production Medium*). The specific productivity of RV-VLPs in culture supernatants was measured by sandwich ELISA, using the 6^th^ International Standard for rabies vaccine that quantify the glycoprotein content (NIBSC, expressed in ELISA Units per ml). Further, we evaluated both media for the production of the rabies vaccine, using stirred tank bioreactors operated in continuous mode (BIOSTAT QPlus, Sartorius). The production of the RV‑VLPs was daily evaluated by ELISA and the obtained harvests analysed by the NIH potency test for rabies vaccine.

**Results and discussion**

After the adaptation process, suspension cultures without aggregates or clumps were obtained, with the same specific growth rate. A lower maximum cell density with the RVPM was reached, achieving 5x10^6^ cells.ml^-1^, compared with the SFM that reach cell densities between 8 and 9x10^6^cells.ml^-1^ in batch mode. The specific RV‑VLPs productivity per cell was maintained, obtaining values of 0.88 and 0.90 EU.10^6^cells^‑1^.day^-1^ for the clone being cultured in SFM and RVPM, respectively.

Taking into account that this producer clone can be changed directly from one medium to the other without *lag* phase or cell damage, and that in RVPM the maximum cell density reached was lower, this medium was proposed to be analysed in high cell density in perfusion mode for a continuous culture in bioreactor.

Therefore, we performed two cultures in parallel to compare the efficacy of each media formulation in perfusion. As shown in Fig. 1, we obtained very similar culture performances in both bioreactors; 14.4 EU.ml^-1^ and 16.1 EU.ml^-1^ of RV-VLPs for the commercial SFM and RVPM, respectively. After that, the harvests were evaluated by the NIH potency test obtaining a rabies vaccine potency of 1.2 IU.ml^-1^ for both cultures (being 1 IU.ml^-1^ the minimum potency required for animal vaccine).

Thus, the results obtained represent an interesting advance in the optimization of this vaccine production process since the use of this new medium formulation represents a reduction of 40% of the total cost which will be reflected in a considerable reduction of the price of the vaccine dose.

**References**

1. Jackson A: **Recovery from rabies: A call to arms**. *J. Neurol. Sci. 2014*, 339:6-7.

2. Briggs D: **The role of vaccination in rabies prevention**. *Curr. Opin. Virol.* 2012, 2:309–314.

3. Meslin F, Briggs D: **Eliminating canine rabies, the principal source of human infection: What will it take?.** *Antiviral Res.* 2013, 98:291–296.

4. D. Fontana D, Kratje R, Etcheverrigaray M, Prieto C: **Rabies virus-like particles expressed in HEK293 cells.** *Vaccine* 2014, 32:2799-2804.

5. D. Fontana D, Kratje R, Etcheverrigaray M, Prieto C: **Immunogenic virus-like particles continuously expressed in mammalian cells as a veterinary rabies vaccine candidate.** *Vaccine* 2015, 33:4238-4246.

**Fig. 1 (abstract P-204).**
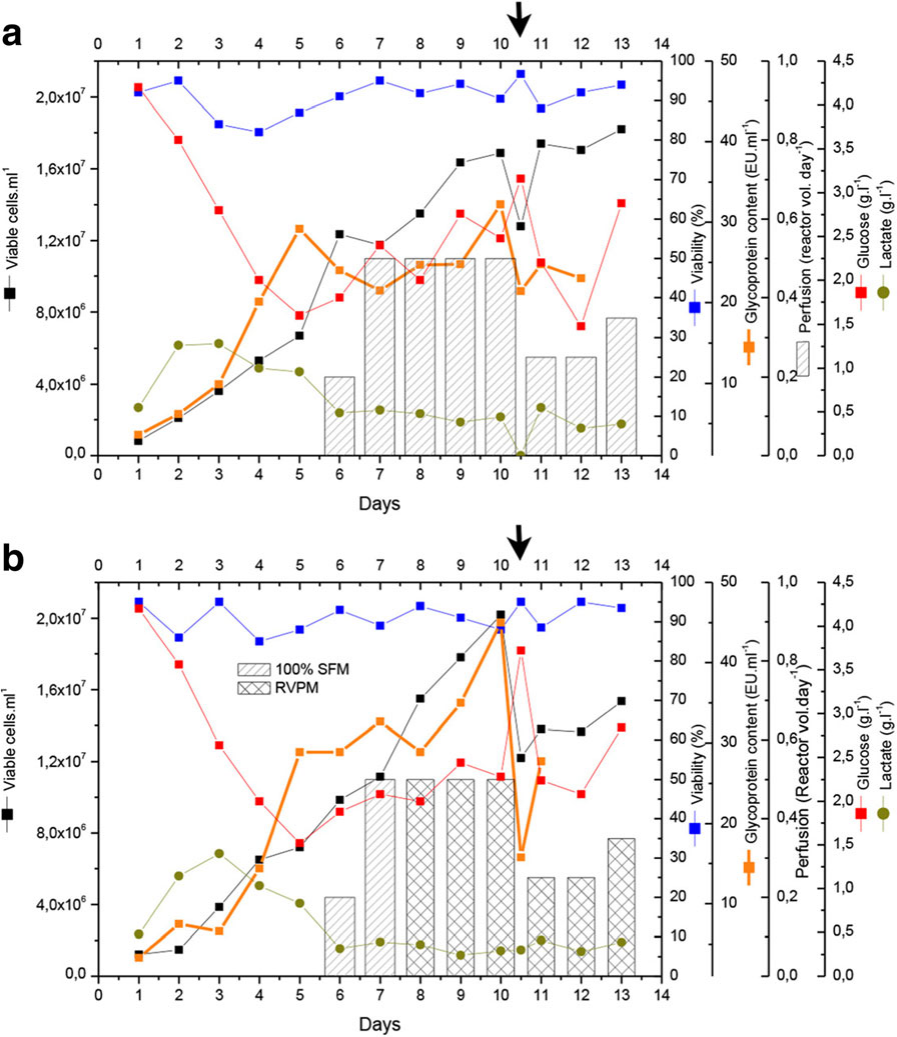
Comparison of media formulations for the vaccine production in 1 L stirred tank bioreactors operated in continuous mode. Both cultures were performed in parallel using the corresponding medium for the perfusion. **a** Feeding was performed with the commercial SFM. **b** The first two days of perfusion feeding was performed with SFM until the cell density reach 10^7^ cells.ml^-1^ and, after that, the bioreactor was fed using the RVPM formulation. (**↓**) On day number 10, 20% of the reactor volume was punctually bled maintaining the working volume

## P-213 Disruptive vaccine manufacturing platform aiming to reach extra low production cost

### J.-C. Drugmand, S. Dubois, A. Reniers, J. Forte, A. Deleau, D. Herbiniat, Y. Dohogne, J. Castillo

#### Univercells SA, Gosselies, Belgium

##### **Correspondence:** J.-C. Drugmand (jc.drugmand@univercells.com)

**Background**

Vaccines are one of the most powerful and effective health inventions ever developed – providing tremendous economic and societal value; yet several factors hinder comprehensive immunization coverage.

Traditional methods of biologics production, based on stainless steel bioreactors, allow pharmaceutical companies to achieve economy of scale, but are limited by high capital expenditures. Such approaches stifle manufacturing innovation and lack long-term cost-effectiveness and sustainability.

Current innovations can cut biologics’ production costs to revolutionize the mainstream use of biologic treatments, focusing on developing fast, potent and cost-effective vaccine production. Univercells’ mission to make biologics affordable to all initiated a paradigm shift, targeting an innovative single-use manufacturing platform incorporating bioprocess into continuous operations.

Univercells employs process intensification, using high volumetric productivity bioreactors; and unit steps integration, coupling USP and DSP into continuous operations. The objective is a down-scaled high-productivity process for a cost-effective manufacturing solution. The resulting micro-facilities are easily-deployable in developing countries, breaking entry barriers to biomanufacturing (Fig. 1).

Manufacturing and distribution advancements, from centralized to distributed, foresee affordable treatments’ obtainability via supplying local populations with local production units.

**Materials and methods**

- Bench-scale fixed-bed bioreactor;
- Carriers made of 100% pure non-woven hydrophilized PET fibers;
- Vero cells grown in serum-free and serum containing media;
- Attenuated polio strains;
- Cell nuclei on carriers counted by the crystal violet method;
- Polio virus production estimated by Elisa assay (D-antigen content).

**Results**

Cultivation of Vero cells in medium with serum and in serum-free medium, was carried out in bench-scale compact fixed-bed bioreactors, to determine which culture conditions result in the highest growth rate, the highest cell biomass by carriers and virus production.

Cells were inoculated at 0.05x10^6^ cells/cm^2^ and infected during the mid-exponential phase, following a complete media exchange. Viral infection took place in serum-free media. In-line clarification and purification is targeted to be performed in only a few steps (maximum one of two) without intermediary diafiltration. In such configuration, we measured that Vero cells can reach a cell density of 300-350x10^3^ cells/cm^2^ with PDL/day of 1.0-1.2 in serum-containing media.

This new facility is expected to manufacture any type of viral vaccine at a very low cost and could be deployed at the site of the manufacturer in emerging countries, killing the two birds of cost of manufacturing and distribution with one stone. The presentation will feature the description of the engineering development, but also the preliminary results of cell growth, infections, and product quality, as well as a description of the COGS calculation.

**Conclusion**

Univercells developed a disruptive polio vaccine manufacturing technology exceeding expectations when compared to traditional methods – achieving a superior result via its all-in-one solution of a simple, scalable, and fully-disposable vaccine production platform resulting in long-term cost-effectiveness, flexibility and sustainability:

- All upstream, downstream and inactivation steps take place within a closed system with all the equipment contained in a low footprint isolator – creating a confined area for polio virus handling that facilitates the deployment of micro-facilities.
- This leads to a dramatic reduction in capital investment, time required for development and increases production capacity.
- In conclusion, this is a simple and elegant solution for the industrial production of human vaccines at a low cost in micro-facilities, making polio vaccines available to all.

**Acknowledgements**

Univercells has granted by the Bill and Melinda Gate foundation to Develop Breakthrough Vaccine Manufacturing Platform.

**Fig. 1 (abstract P-213).**
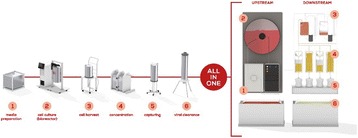
Schematic representation of Univercells’ all-in-one manufacturing process

## P-225 Extensive rearrangements but high genomic stability in a biotechnologically advantageous derivative of modified vaccinia virus Ankara

### Ingo Jordan^1,2^, Deborah Horn^1^, Kristin Höwing^1,3^, Lars Haag^4^, Volker Sandig^1^

#### ^1^ProBioGen AG, 13086 Berlin, Germany; ^2^CureVac AG, 72076 Tübingen, Germany; ^3^Sartorius Stedim Cellca GmbH, 88471 Laupheim, Germany; ^4^Vironova AB, 113 30 Stockholm, Sweden

##### **Correspondence:** Volker Sandig (volker.sandig@probiogen.de)

**Background**

Vectored vaccines based on modified vaccinia virus Ankara (MVA) are reported to stably maintain large transgenes, and to be safe, immunogenic and tolerant to pre-existing immunity. MVA is usually produced on primary chicken embryo fibroblasts but continuous cell lines are being investigated as more versatile substrates.

We have previously reported development of a continuous suspension cell line (CR.pIX) derived from the muscovy duck and efficient production process for MVA in chemically defined media [1,2]. This process allowed isolation of an hitherto undescribed genotype (MVA-CR19) that induced fewer syncytia in adherent cultures and replicated to higher infectious titers in the extracellular volume of suspension cultures [3]. Replication of MVA-CR19 remained restricted predominantely to avian cells, an important property of MVA vectors.

**Material and methods**

Homologous recombination in CR.pIX cells was used to generate viruses with various expression cassettes in deletion site III [4] and combinations of the differentiating point mutations of MVA-CR19 in a backbone of wildtype virus. All recombinant viruses were plaque-purified. Successful introduction of the mutations was confirmed by sequencing and specifically designed restriction fragment length polymorphisms (RFLPs). Viruses were analyzed by serial passaging, diagnostic PCRs accross deletion sites [4], replication kinetics, plaque phenotype and electron microscopy. The genome was further investigated by anchored PCR and long PCR.

**Results**

Efficiency of spread of recombinant viruses (Fig. 1a) could be mapped to a point mutation in one of the genes, A34R. However, although MVA-CR19 carries mutations in three structural proteins we detected no obvious differences to wildtype by electron microscopy (Fig. 1b).

The replacement of the left viral telomere by the right counterpart was the most surprising result of our new study (Fig. 1c). This extensive rearrangement affects 15 % of the viral genome and has also increased the area of complementarity between the two telomeres. The recombination site was precisely located and shown via analysis of earlier and subsequent passages to be a stable property of MVA-CR19.

Various viruses, including those with larger dual (DsRed1 and GFP) expression cassettes, were serially passaged at least 20-fold. Although the genotype of MVA-CR19 is advantageous for replication, all genomic and genetic markers of wildtype and MVA-CR19 were stably maintained in all passages of the recombinant viruses, independent of wildtype or MVA-CR19 backbone.

**Conclusions**

We confirmed our previous results that suggested that MVA-CR19 replicates efficiently in single-cell suspensions and were able to connect this property with the D86Y mutation in A34, a structural protein on the surface of the virions.

MVA-CR19 was also found to differ from wildtype MVA by a recombination between left and right viral telomere. Due to this event, several genes encoded at the left terminus have been deleted whereas the gene dosis of those originally encoded only at the right terminus may have increased. We do not currently know how much the various point mutations and changes in genomic structure combine to explain the improved replication of MVA-CR19. As several of the affected genes have been reported in the literature to impact interaction of MVA with the host we would expect that in vivo studies may reveal additional novel properties of MVA-CR19.

An extremely important distinction between our earlier study [3] and this one concerns the source of the viruses. Here, we investigated plaque-purified viruses and confirm the high genetic and genomic stability of MVA. Different expression cassettes inserted into deletion site III, all diagnostic RFLPs and PCRs over various sites of the genome and within the viral telomeres remained unchanged throughout at least 20 serial passages - independent of whether recombinant viruses with wildtype or CR19-derived backbones were characterized.

**References**

1. Jordan I, Vos A, Beilfuss S, Neubert A, Breul S, Sandig V: **An avian cell line designed for production of highly attenuated viruses**. *Vaccine*. 2009, **27**: 748–756.

2. Jordan I, Northoff S, Thiele M, Hartmann S, Horn D, Höwing K, Bernhardt H, Oehmke S, von Horsten H, Rebeski D, Hinrichsen L, Zelnik V, Mueller W, Sandig V: **A chemically defined production process for highly attenuated poxviruses**. *Biol J Int Assoc Biol Stand*. 2011, **39**: 50–58.

3. Jordan I, Horn D, John K, Sandig V: **A genotype of modified vaccinia Ankara (MVA) that facilitates replication in suspension cultures in chemically defined medium**. *Viruses*. 2013, **5**: 321–339.

4. Kremer M, Volz A, Kreijtz JHCM, Fux R, Lehmann MH, Sutter G. **Easy and efficient protocols for working with recombinant vaccinia virus MVA**. *Methods Mol Biol Clifton NJ*. 2012, **890**: 59–92.

**Fig. 1 (abstract P-225).**
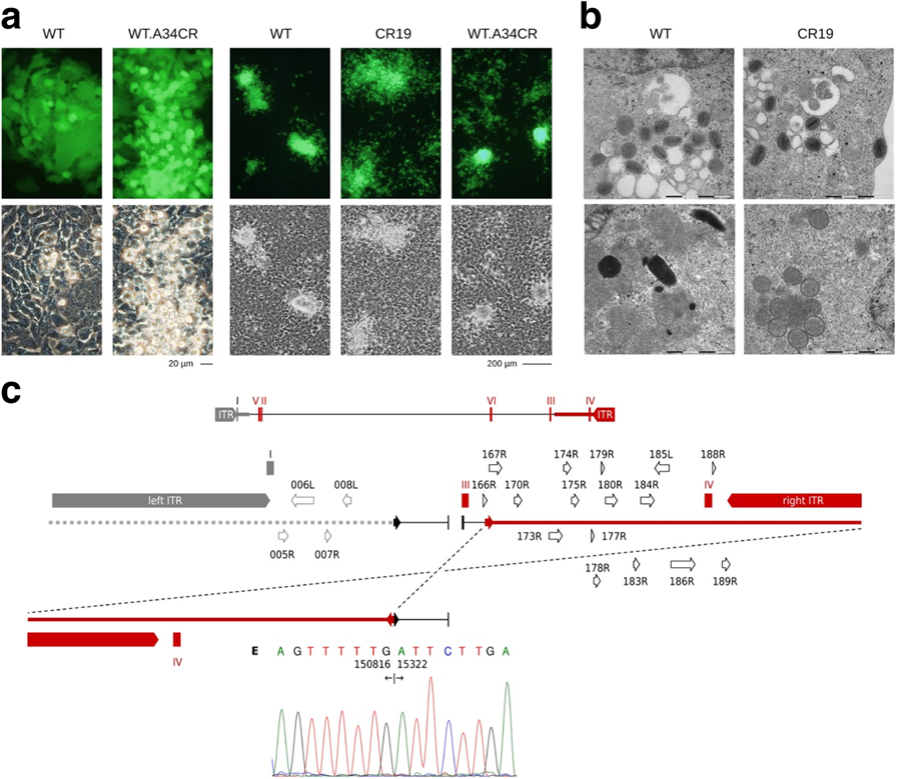
**a** One hallmark of MVA-CR19 is a significantly reduced tendency to induce syncytia and an increased dispersion of plaques in CR.pIX cell monolayers. This property appears to be supported by the mutation in A34R. **b** Electron microscopy reveals no obvious differences between novel genotype and wildtype. The top panels show intracellular infectious viruses, the bottom panels immature virions in the process of genome packaging. The scale bar in (**b**) is 1 μm wide, experiments in (**a**) and (**b**) werde done with recombinant viruses that express GFP. **c** The right telomere of the virus, starting with bp 150816, has been found to have recombined with the left telomere at bp 15322 (using numbering of GenBank sequence U94848). Top panel in (**c**) is a diagram of the full wildtype genome, center panel enlarges the telomeres of the wildtype, and bottom panel highlights the left telomere of MAV-CR19 together with the recombination site. ITR, inverted terminal repeat; roman numerals for amplicons that confirm deletion sites in MVA, open arrows for genes loacated in the telomeres

## P-227 Optimized transfection efficiency for CHO-K1 suspension cells through combination of transfection and culture media

### Abdalla A. Elshereef^1,2^, André Jochums^2^, Antonina Lavrentieva^2^, Janina Bahnemann^2^, Dörte Solle^2^, Thomas Scheper^2^

#### ^1^Institut für Technische Chemie, Leibniz Universität Hannover, Hannover, 30167, Germany; ^2^Pilot Plant Facility, Natural & Microbial Products, National Research Center, Cairo,12622, Egypt

##### **Correspondence:** Abdalla A. Elshereef (elshereef@iftc.uni-hannover.de)

**Background**

Transient gene expression systems using polyethylenimine (PEI) are considered to be fast, flexible and cost-efficient for recombinant protein production [1]. Transfection efficiency depends on different factors; one of them is the type of media. Production media support cell growth and protein production but not high transfection efficiency (TE) mediated by PEI [2]. Therefore, media were selected for transfection followed by feeding of production media [3] to improve TE and protein production. Two different transfection strategies are compared: conventional transfection by preparing polyplex of a plasmid (pDNA) and PEI interaction before transfection and *insitu* transfection by direct addition both of them to the cell suspension and the polyplex formed spontaneously [4].

**Materials and methods**

Cells were seeded 24 hr in CHOMACS CD media before transfection. At transfection time point an equal amount of cells were resuspended in each media type. Transfection was applied either *insitu* or conventional (polyplex prepared in 100 μL of 150 mM NaCl and incubated for 20 min.), media addition was performed 5 hours post-transfection (hpt). Media type and transfection condition were illustrated in Table 1.

**Results**

Media screen result exhibits the highest transfection efficiency of around 50% transfected cells by Opti-MEM medium coming along with low cell growth and viability. To improve the transfection efficiency, basic parameters including cell density, pDNA, and PEI concentrations were varied and higher transfection efficiency was reached by reducing media or accordingly increasing cell density, PEI and pDNA concentration for transfection. Further optimization results show that the transfection of CHO-K1 cells in Opti-MEM (transfection medium) for 5 hours followed by addition of CHOMACS CD (production medium) for further enhancing the transfection, cell count, and cell viability. The transfection efficiency (TE) increased up to 85 ± 2.6% coincide with increases in viable cell concentration (VCC) in comparsion to transfection and cultivation in Opti-MEM media alone Fig. 1a.

Both conventional and *insitu* methods are successfully transfected CHO-K1 to the same similar high TE as shown in fluorescence microscope images of Fig. 1b. *Insitu* transfection shows super-priority for suspension cell transfection concerning the reduction of handling steps (one step) compared to the conventional way (two steps). The *insitu* transfection avoiding the optimization step required for the incubation period to prepare transfection polyplex but require a higher amount of pDNA and PEI than conventional way as shown in Table 1.

**Acknowledgements**

I would like to thank DAAD for a Ph.D. grant and to thank (Institute of Technical Chemistry, Leibniz University of Hannover) for supporting me with all required facility.

**References**

1. Pham PL, Kamen A, Durocher Y. Large-scale transfection of mammalian cells for the fast production of recombinant protein. *Mol Biotechnol*. 2006;34(2):225-237. doi:10.1385/MB:34:2:225.

2. Meleady P. *Heterologous Protein Production in CHO Cells*. Vol 1603.; 2017. doi:10.1007/978-1-4939-6972-2.

3. Baldi L, Hacker DL, Adam M, Wurm FM. Recombinant protein production by large-scale transient gene expression in mammalian cells: state of the art and future perspectives. *Biotechnol Lett*. 2007;29(5):677-684. doi:10.1007/s10529-006-9297-y.

4. Backliwal G, Hildinger M, Hasija V, Wurm FM. High density transfection with HEK 293 cells allows doubling of transient titers and removes need for a priori DNA complex formation with PEI. *Biotechnol Bioeng*. 2008;99(3):721-727. doi:10.1002/bit.

**Table 1 (abstract P-227).** Summary of transfection conditions

Parameters	Media screen for GFP transfection	Optimize of GFP transfection	Comparison of transfection method	
Media type	ProCHO5, Opti-MEM, CHOMACS CD, ProCHO4, UltraCHO, DMEM/F12	Opti-MEM + CHOMACS CD	Opti-MEM + CHOMACS CD	
Transfection volume (w/o or w media addition at 5 hpt*)	5 mL	12.5 + 12.5 mL	2.5 + 2.5 mL	
Transfection protocol	*Insitu*	*Insitu*	Conventional	*Insitu*
Cells amount	10 x 10^6^	62 x 10^6^	10 x 10^6^	
pEGFP-N1 plasmid pDNA (μg)	6	37.5	5	6
Linear PEI 25 Kda (μg)	30	187.5	15	30
Scale	Spin tube®50 bioreactor	25 mL-Shake flask	Spin tube ®50 bioreactor	

*w/o= without and w= with

Transfection efficiency was monitored at 48 hpt by fluorescence microscope and flow cytometer. Cell count and viability were determined by Cedex analyzer

**Fig. 1 (abstract P-227).**
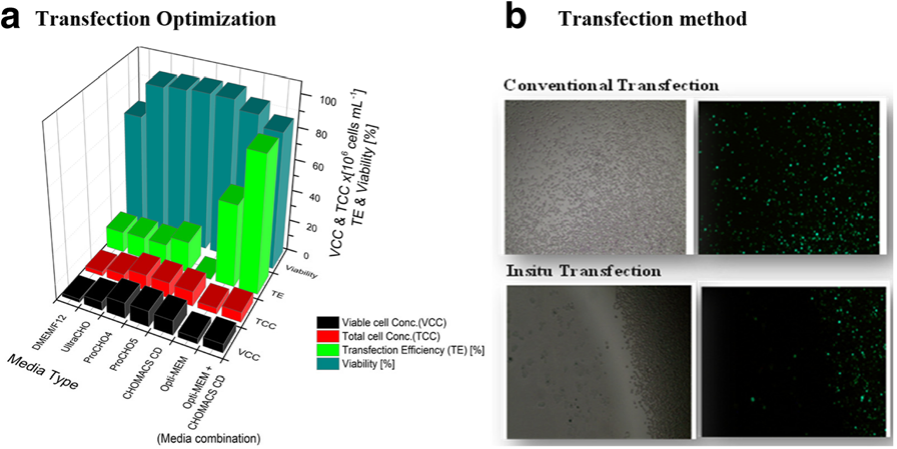
**a** Screening of six different media for CHO-K1 transfection and optimized transfection effeiciency by media combination. **b** Transfection efficiency byconventional and insitu transfection, images obtained48 hpt by flurescence micropscopy at 10x magnification

## P-228 Regulation of recombinant protein expression during CHOBRI/rcTA pool generation increases productivity and stability

### Adeline Poulain^1,2^, Alaka Mullick^1,2^, Yves Durocher^1,3^, Bernard Massie^1,2^

#### ^1^Human Health Therapeutics Portfolio, National Research Council, Montreal, QC, Canada; ^2^Department of microbiology, infectiology and immunology, Université de Montreal, Montreal, QC, Canada; ^3^Department of biochemistry, Université de Montreal, Montreal, QC, Canada

##### **Correspondence:** Bernard Massie (bernard.massie@cnrc-nrc.gc.ca)

**Background**

In order to deal with the growing demand of large quantities of therapeutic proteins in a timely fashion, expression systems are being optimized to reduce the time of generation of stable clones as well as to increase the levels of protein secretion. This can be achieved by a combination of expression cassette optimization, cell engineering and selection process.

**Materials and methods**

We have previously developed the cumate gene-switch, which is a very efficient expression system for protein production [1].

**Results**

We have shown that the cumate-inducible promoter (CR5) was the strongest promoter we had tested so far in Chinese hamster ovary (CHO) cells. With this promoter, we were able to generate stable CHO pools capable of producing high levels of a Fc fusion protein (900 mg/L), outperforming by 3 to 4 fold those generated with CMV5 and hybrid EF1α-HTLV constitutive promoters. Besides the strength of the CR5 promoter, we demonstrated that the ability to control both the time and the level of expression during pool generation and maintenance gave a real advantage to the inducible expression system. Indeed, we observed that keeping the expression OFF during selection enabled the generation of pools with superior productivity compared with the pools whose expression was maintained ON. Moreover, preliminary results suggest that keeping recombinant protein expression down increases the frequency of high producer clones [2].

**Conclusions**

Knowing that one of the main bottlenecks of the successful bioprocessing of recombinant proteins using CHO cells is the rapid isolation of a high producer, our data suggest that the cumate gene-switch system could be a valuable platform for the generation of stable clones.

**Acknowledgements**

This work was supported by the National Research Council Canada: Human Health Therapeutics portfolio.

**References**

1. Mullick, A., Y. Xu, R. Warren, M. Koutroumanis, C. Guilbault, S. Broussau, F. Malenfant, L. Bourget, L. Lamoureux and R. Lo: **The cumate gene-switch: a system for regulated expression in mammalian cells.** *BMC biotechnology* 2006, **6(1):** 43.

2. Poulain, A., S. Perret, F. Malenfant, A. Mullick, B. Massie and Y. Durocher: **Rapid protein production from stable CHO cell pools using plasmid vector and the cumate gene-switch.** *Journal of Biotechnology* 2017, **255:** 16-27.

## P-231 Generating monoclonal production cell lines with ≥ 99.9 % probability

### Albert J. Paul, Verena Fischer, Christoph Zehe

#### Sartorius Stedim Cellca GmbH, 88471 Laupheim, Baden-Württemberg, Germany

##### **Correspondence:** Albert J. Paul (albert.paul@sartorius-stedim.com)

**Background**

The main regulatory authorities and organizations demand proof of monoclonality for biotechnological producer cells. With increasing pressure to shorten timelines and to improve drug safety, technologically advanced methods have to be established to ensure that production cell lines are derived from a single progenitor cell.

Sartorius Stedim Cellca’s single cell cloning approach is based on one round of fluorescence-activated cell sorting (FACS) using Becton Dickinson (BD) FACSAriaTM Fusion cell sorter combined with photo-documentation by SynenTec Cellavista microscopic imaging system. For the approach, critical process parameters such as different cell lines, viability and cell aggregation levels were investigated separately to assess their contribution to the probability of monoclonality.

**Materials and Methods**

A variety of producer cell pools generated from Sartorius Stedim Cellca’s CHO DG44 host cell line were used for the evaluation of probability of monoclonality. Single cell cloning experiments were performed using FACSAria FusionTM (BD). Three to one day(s) prior to the experiments, cells were seeded at 3 x 10^5^ cells/mL. On day of the experiment, the cells were centrifuged and stained with DyLight 650 conjugated Protein A. Subsequently, cells were washed, resuspended in PBS, filtered through a FACS tube with cell strainer cap and analyzed by flow cytometry. The top 3–5 % population with regard to DyLight 650 fluorescence was selected and FACS droplets were sorted into 384-well flat bottom plates containing cloning medium. Doublet discrimination was done by using a FCS-W/FCS-H dot plot, and a SSC-W/SSC-H dot plot. Immediately after single cell cloning into 384-well plates (1 cell/well) the plates were centrifuged followed by imaging using the Cellavista (day 0). Further Cellavista images are taken on day 1, day 2 and on one day between day 5 and 7. Outgrowth was defined at day 14.

**Results**

8 cell lines expressing different recombinant products were investigated to calculate probability of having ≥ 2 cells/well after FACS sorting P(d), the apparent probability P(i) of having ghostcells (cells that are out-of-focus and, thus, are not visible during initial microscopic imaging), and the apparent probability P(k) of having ghostcells that outgrow the 384-well stage (Fig. 1).

Using these results, the probability of obtaining a monoclonal cell by using Sartorius Stedim Cellca’s single cell cloning approach was determined (Table 1) by

Conservative examination: P(monoclonal, conservative) = 1 – (P(d) x P(i))

Realistic examination: P(monoclonal, realistic) = 1 – (P(d) x P(k))

Cell pools with low viability can theoretically impact the probability of monoclonality by e.g. diminishing microscopic imaging quality (cell debris). Therefore, pool cell line 1 with very low viability (≥36 %) was used to demonstrate, that the probability of monoclonality is still 99.9 % in case of low viability on day of sorting:

P(monoclonal, conservative) = P(d) x P(i) = 99.9 %

P(monoclonal, realistic) = P(d) x P(k) = 99.9 %

Furthermore, cell pools with high aggregation levels can theoretically impact the probability of monoclonality by sticking together during FACS sorting and therefore increase the probability P(d) of having ≥ 2 cells/droplet. Therefore, pool cell line 8 with high aggregation levels (≥11.1 %) was used to demonstrate, that the probability of monoclonality is still ≥ 99.9 % in case of highly aggregated cell pools on day of sorting:

P(monoclonal, conservative) = P(d) x P(i) = > 99.9 %

P(monoclonal, realistic) = P(d) x P(k) = > 99.9 %

**Conclusions**

In summary, there is no obvious correlation between protein product type and the determined probabilities for monoclonality. Furthermore, pools with a viability as low as 36 % and pools with an aggregation level as high as 11.1 % can be used for SCC resulting in acceptable probabilities of monoclonality.

**Fig. 1 (abstract P-231).**
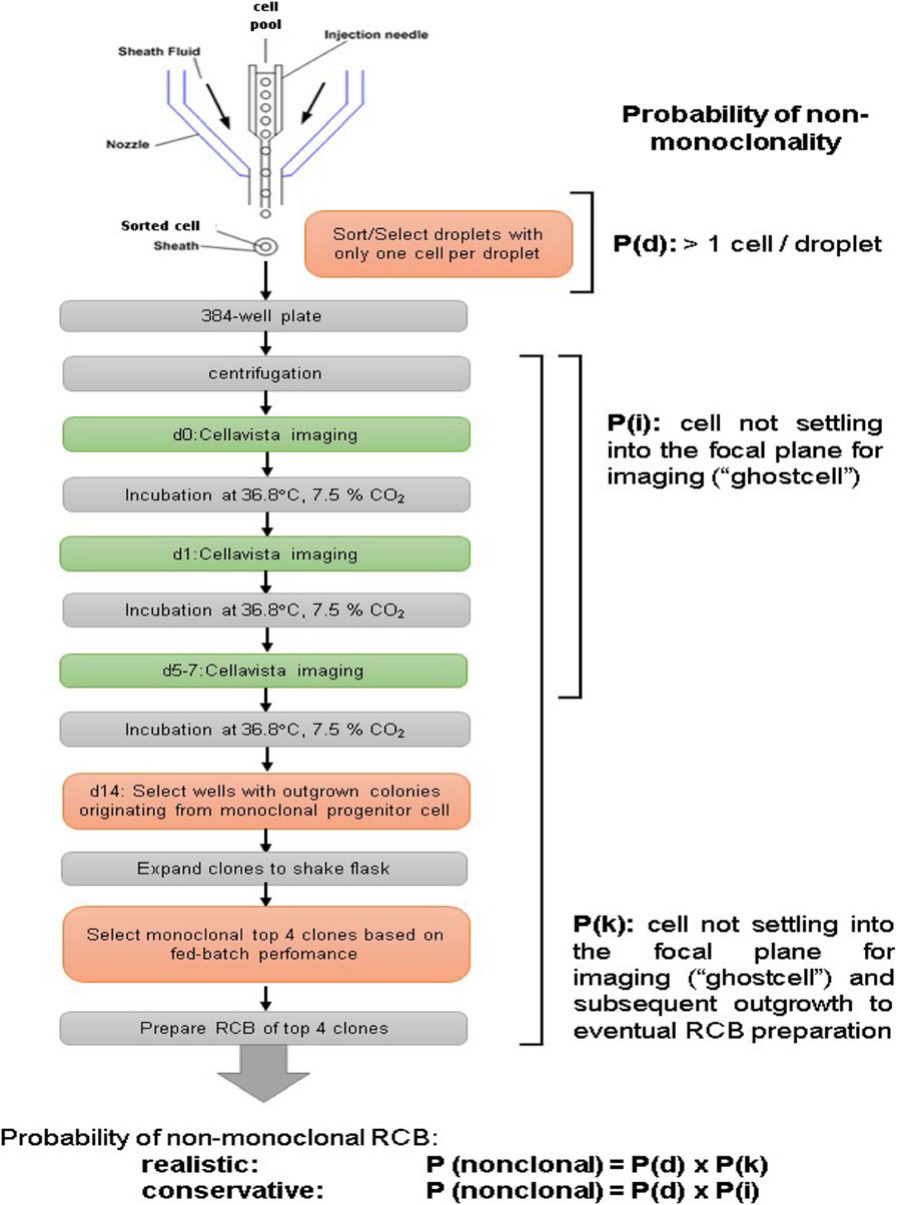
Schematic Overview of Sartorius Stedim Cellca’s single cell cloning procedure

**Table 1 (abstract P-231).** Probability of monoclonality of eight different cell lines, each expressing a distinct protein product

	Recombinant antibody product	P(monoclonal, conservative): confidence ≥ 95%	P(monoclonal, realistic): confidence ≥ 95%
Pool cell line 1	IgG4	99.9 %	99.9 %
Pool cell line 2	IgG4	99.8 %	>99.9 %
Pool cell line 3	Complex protein	>99.9 %	>99.9 %
Pool cell line 4	IgG1	>99.9 %	>99.9 %
Pool cell line 5	IgG1	>99.9 %	>99.9 %
Pool cell line 6	IgG4	>99.9 %	>99.9 %
Pool cell line 7	Complex protein	>99.9 %	>99.9 %
Pool cell line 8	IgG1	>99.9 %	>99.9 %

## P-232 Legacy cloning methods in the modern world part 1: reassessing the capillary aided cell cloning technique

### Alison Porter^1^, Ian Tedder^2^, John McGuire^3^, Andy Racher^4^

#### ^1^Process Development Sciences, Lonza Biologics plc, 228 Bath Road, Slough, SL1 4DX, UK; ^2^Cell Culture Development, Lonza Biologics plc, 228 Bath Road, Slough, SL1 4DX, UK; ^3^European Regulatory Affairs–Mammalian, Lonza Biologics plc, 228 Bath Road, Slough, SL1 4DX, UK; ^4^Future Technologies, Lonza Biologics plc, 228 Bath Road, Slough, SL1 4DX, UK

##### **Correspondence:** Alison Porter (alison.porter@lonza.com)

**Background**

ICH guidance [1] requires that any cell line used to produce biopharmaceuticals originates from a single progenitor cell. Recently, there has been increased scrutiny of the method(s) used to achieve this requirement. Here, we review the suitability of the legacy capillary aided cell cloning (CACC) method in light of this changing landscape of expectations.

The CACC method is based on the ‘spotting’ technique [2] and relies on independent visual conformation by two scientists of the presence of a single cell in a 1 μL droplet. This method achieves a high probability of monoclonality in one cloning round. Although the method has since been replaced by FACS single cell deposition for routine use, it remains a viable cloning method.

**Materials and methods**

- Performed by trained scientists
- Dilute culture to 1500 ± 500 cells/mL with ≤2% doublets
- Draw cell suspension into pipette tip by capillary action; tap tip against the centre of the base of each well of a 48 well plate.
  - Size of resulting droplet = ~1μL (Fig. 1a)
- Two scientists independently view all wells using a microscope (initially use 40x magnification with the entire rim of the droplet visible within the field-of-view. Next, examine particles using 100x or 200x magnification to confirm they are cells) and individually record the number of cells present in each well’s droplet (Fig. 1b to d).
- Exclude droplet from further analysis if full visualisation is hindered (Fig. 1e to h).
- Add growth medium, and incubate plates. Record all wells containing colonies; only progress colonies from wells that both scientists agree contains only one cell.
- Data analysis:
  - Each scientist’s observations categorised as: 0 cells, 1 cell or >1 cell
  - Observed outcome for each well: growth or no growth
  - Probability of monoclonality estimated from data using a statistical model

**Results**

Advantages of the CACC Method Compared to Limiting Dilution Cloning (LDC)

Increased accuracy of P(monoclonality) with CACC

- LDC weakness: no visualisation after seeding (to check both well seeding and subsequent growth of colonies is well described by the Poisson distribution), potentially overestimating P(monoclonality)
- Addressed by CACC: Visual examination with colonies arising from wells seeded with 1 cell distinguished from those seeded with >1 cell
- Visualisation step further strengthened by: Using controls for exclusion of wells; measuring errors based on the presence or absence of colonies in wells where two scientists independently reported 0 cells; and formally analysing the data using a suitable statistical model

Decreased time and resource requirements with CACC

- High P(monoclonality) possible in single round as each well examined individually with only those containing a single cell progressed, and because the error rate for incorrect scoring is considered to be low

Frequency of Errors With CACC Method

Possible visual observation errors considered:

- two scientists miss a cell
- one cell sitting on top of another and the two thus appearing as one

An experiment was performed to estimate error frequency [3].

Conclusion

- Scientists miss a cell infrequently (in the range 0.4% to 1.3%, [3])
- Error frequency does not invalidate use of direct observation methods for cell cloning
- Single cell seen by both scientists is highly likely to be monoclonal

Summary of Strategies in Place to Control Errors

- During method development, strategies established to control potential sources of error (Table 1)

**Conclusions**

Use of a contemporaneous visualisation approach, a strict control strategy, and a suitable statistical model (which takes into account potential errors) results in:

- The CACC method being at least as robust as the LDC method
- The CACC method being a reliable, single-step method for cloning to achieve a high P(monoclonality)

**Acknowledgements**

R. Boraston for photographs in Fig. 1.

**References**

1. International Committee on Harmonisation (ICH) Q5D (1997). www.ich.org.

2. Clarke JB & Spier RE (1980). Arch. Virol, 63:1-9.

3. Onadipe OA, Metcalfe KM, Freeman PR & James C (2001). E. Lindner-Olsson et al (eds), Animal Cell Technology: From Target to Market, 72-74.

**Fig. 1 (abstract P-232).**
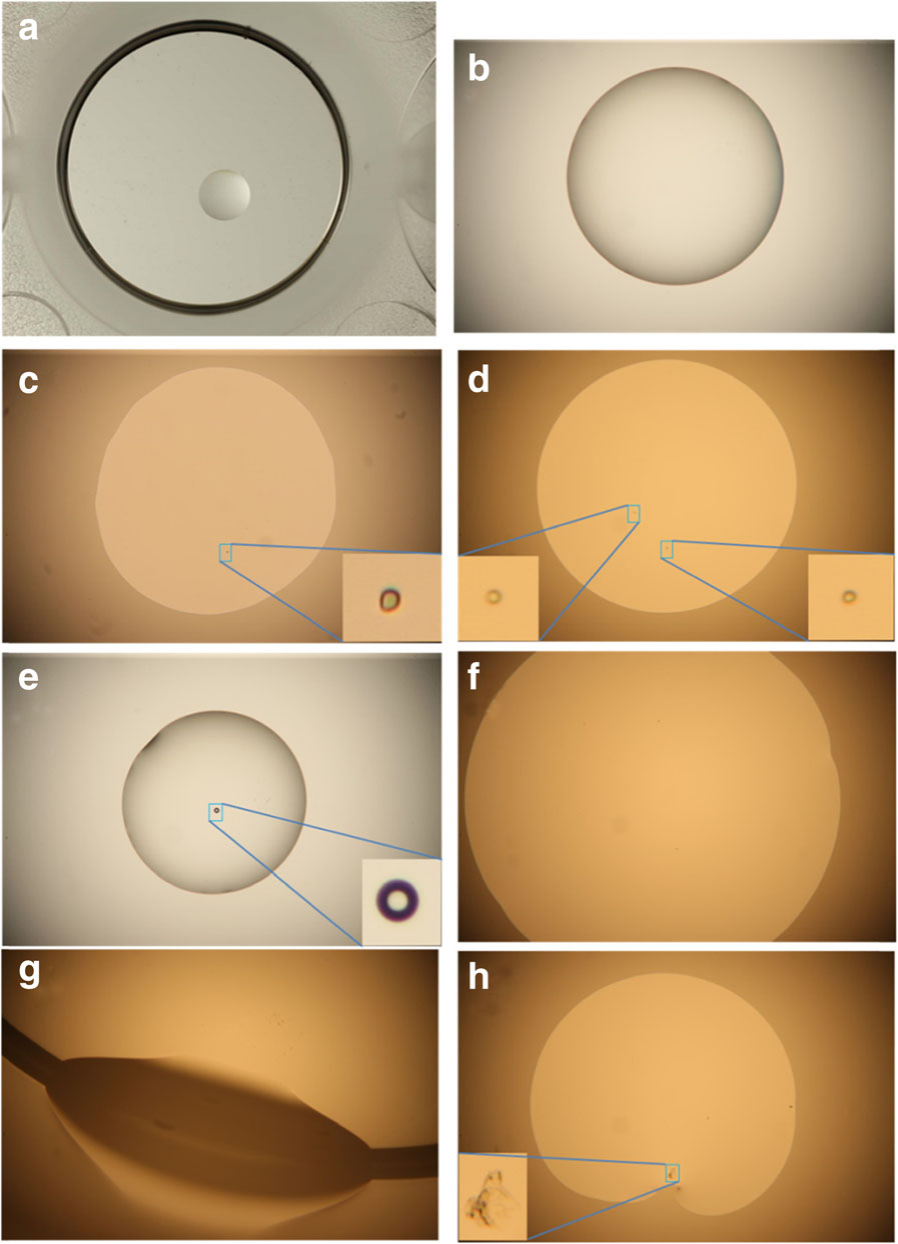
Images of droplets. **a** Image of a droplet in a well. Images of droplets containing (**b**) 0, **c** 1 and **d** 2 cells. Images of droplets which (**e**) contains an air bubble, **f** cannot be completely visualized in a single field-of-view, at 40x magnification, **g** has touched the edge of the well (an example situation where the boundary of the droplet is not clear), and **h** contains debris

**Table 1 (abstract P-232).** Potential sources or error and strategies to control them

Potential Error	Impact	Control strategy
Presence of doublets or clumps of cells	Doublets are unlikely to affect P(monoclonality) as wells with droplets containing these will not be recorded as containing a single cell and will therefore not be progressed. However, presence of a doublet could increase the chance of a cell being sat exactly on top of another and therefore being missed. It will also increase time and resource required to achieve a desired number of wells which both scientists have recorded as containing a single cell. As such, it is good practice to ensure proportion of doublets is low	Ensure cell sample to be cloned contains ≤2% doublets
Scientists hindered from visualising cells within a droplet	Scientists miss cells and P(monoclonality) is misleadingly high	Procedure in place to exclude wells with droplets harbouring certain characteristics from further analysis and progression
An error is made in determining the number of cells a droplet contains	P(monoclonality) is misleadingly high or low	(i) Two independent scientists view the droplets (ii) Scientist fatigue is controlled by limiting the amount of time a scientist can spend cloning on any given day (iii) Statistical model takes into account potential errors

## P-233 Improved vector design eases cell line development workflow in the CHOZN® GS^-/-^ expression system

### Amber N. Petersen, Trissa Borgschulte

#### MilliporeSigma, St. Louis, MO, USA

##### **Correspondence:** Amber N. Petersen (amber.petersen@sial.com)

**Background**

Vector design is a key step in cell line development for the expression of therapeutic biologics. It is essential that the vector design results in high, stable expression of the encoded protein. Other considerations include ease of cloning, stability for propagation in E. coli as well as in the mammalian host cell line, and ease of sequence amplification for verification of vector construction and for detection of insertion site and copy number in stably expressing cells. For these reasons, use of the same promoters and polyA tails in dual cassette vectors, as is common for expression of the heavy and light chains of monoclonal antibodies, can be problematic. In order to minimize sequence similarities between the two expression cassettes, we have modified the promoters, introns, and polyA tails of the light chain and heavy chain expression cassettes in the dual expression vector commonly used for the expression of therapeutic antibodies in the CHOZN® GS^-/-^ cell line development platform.

**Materials and methods**

Gene synthesis and vector construction of IgG1 and fluorophore-expressing vectors was done by Atum. Vectors were transfected into CHOZN® GS^-/-^ cells via electroporation. Analysis of GFP and RFP expression was achieved using a MACSQuant instrument. Selection and generation of stable pools and single cell clones from transfections with IgG1-encoding vectors was performed as described in the CHOZN® Platform Technical Bulletin. Titer analysis was performed in static (96 well plate), in a 7 day TPP assay and in a 14 day fed batch assay using a QK ForteBio.

**Results**

Initial screening experiments identified a lead vector, #39, and a vector, #37, which produced very low titers and relatively few minipools expressing detectable levels of IgG1. Analysis of GFP and RFP expression from the modified vectors indicated relatively high expression from the RFP/HC expression cassette of vector #37. A stronger promoter resulting in overabundance of HC, known to be toxic to cells, provides a possible explanation for the poor results with this vector. Interestingly, swapping the positions of the LC and HC in #37 resulted in a vector, #77, that out-performed the initially identified lead vector (Fig. 1). This same change was made to vector #39 without any resulting improvement in titers (vector #78, Fig. 1). Interestingly, vector #39 had a smaller difference in relative promoter strengths, based on mean channel fluorescence ratio of GFP to RFP, suggesting that overabundance of HC was not an impediment to IgG1 expression from #39. Poor titers were also seen with a modified version of vector #39 (vector #79, Fig. 1) in which the glutamine synthethase selection cassette was in the reverse orientation. This second screen identified vector #77 as the lead vector design (Fig. 1). A full comparative study of vector #77 and the control vector was performed, cumulating in the generation and comparison of single-cell clones from each. These studies have demonstrated the equivalence of these vectors in terms of IgG1 titer.

**Conclusions**

This work has resulted in the identification and characterization of a dual expression vector with minimized similarity between the two expression cassettes, easing the cloning, propagation and analysis of vector integration in stable cell lines while maintaining the high, stable expression of the encoded protein of the original vector design.

**Acknowledgements**

We acknowledge Atum for their contributions to vector design and construction.

**Fig. 1 (abstract P-233).**
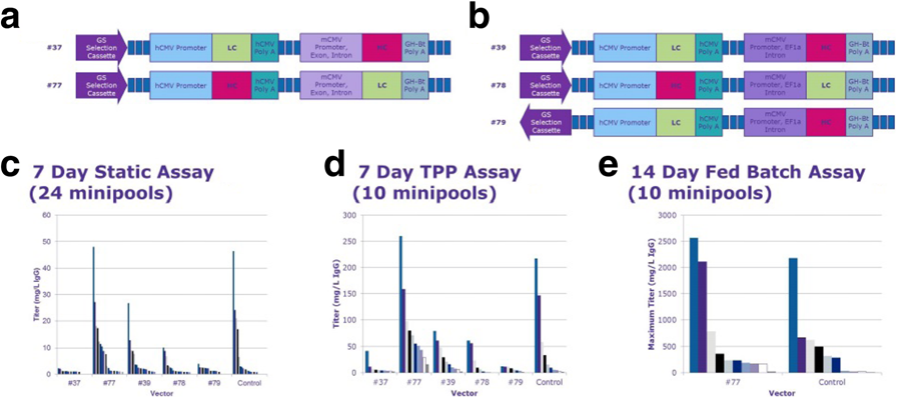
Top Performing Vector Design Created by Switching the Relative Positions of the Light and Heavy Chain of Vector #37. The mean channel fluorescence ratio of vector #37 expressing GFP and RFP in place of the LC and HC indicated high expression from the HC-expressing cassette, providing a potential explanation for the poor titers associated with this vector design. Therefore, modifications of vectors #37 (**a**) and #39 (**b**) were created in which the relative positions of the LC and HC were switched. In addition, the orientation of the GS cassette was reversed in vector #39 with the intent to improve titers through increased selection stringency (**b**). IgG1 titers of select minipools during the scale-up process are shown for each vector (**c**-**d**)

## P-236 A novel siRNA aided method for CHO cell line selection

### Andreas B. Diendorfer^1^, Vaibhav Jadhav^1^, Zach Wurz^2^, Frank Doyle^3^, Ted Eveleth^2^, Scott Tenenbaum^3^, Nicole Borth^1, 4^

#### ^1^Austrian Center of Industrial Biotechnology, Vienna, Austria; ^2^HocusLocus LLC, Albany, NY, USA; ^3^State University of New York Polytechnic Institute, Albany, NY, USA; ^4^University of Natural Resources and Life Sciences, Vienna, Austria

##### **Correspondence:** Andreas B. Diendorfer (andreas.diendorfer@boku.ac.at)

**Background**

Traditional cell line engineering strategies mainly include an antibiotics resistance selection. In this process, cells are transfected with the GoI (gene of interest) together with an antibiotics resistance gene and those cells are selected that survive treatment with the respective antibiotic [1]. Although the gene responsible for the survival of the cell is transfected together with the GoI, resistance is not necessarily linked to high GoI expression. Thus, a significant proportion of resistant cells may not express the GoI at all, necessitating the search for alternative, more closely linked selection systems.

siRNAs (silencing inducing RNAs) are short, non­coding RNAs that can bind to complementary mRNA and inhibit their translation. This function has been used in many approaches to silence the expression of certain genes [2]. With their short length, siRNAs can be hidden in introns (non-translating regions) of genes, making it possible to couple the expression of a siRNA to a gene. This way a cell produces a correlating amount of siRNA when transcribing the gene, without adding any further translational burden on the cell.

The co-expression of the siRNA can be used as a selective marker by one of the following methods: (1) Knock-down of a suicide gene to enable a cell’s survival after suicide gene mRNA transfection, (2) down-regulation of a surface marker which is used in MACS (magnetic cell separation) to filter out wanted or unwanted cells, and (3) inhibition of a fluorophore marker for selection using FACS without product specific antibodies.

For siRNA based cell selection systems, siRNAs replace the commonly used antibiotics resistance as a marker. Cells that produce GoI will also produce the siRNA that protects the cell from a suicide gene. The selection protein (suicide genes, fluorophores, surface markers, etc.) is transfected as mRNA and is only expressed during selection.

**Materials and methods**

The general process is outlined in Fig. 1. (A) The traditional antibiotics resistance marker is replaced by an siRNA, which is co­transcribed with the GoI. Unlike in antibiotic resistance, the marker here is not a protein, reducing the translational burden and providing more resources for GoI production [3]. (B) Transcription produces GoI mRNA together with either resistance gene or the spliced and processed siRNA. (C) Selection is done by the addition of antibiotics in the traditional approach or by transfecting a selective marker’s mRNA with a target site complementary to the product dependent siRNA. (D) Traditionally selected cells are selected based on the amount of antibiotic resistance they produce. siRNA knocks down a marker gene (suicide gene or a marker for MACS or FACS processing), but does not need an additional protein for selection. The genetic coupling of siRNA and GoI ensures selection based on GoI productivity.

**Results**

Transfection with the suicide gene proved to be 100% lethal within 2 days, with no outgrowth over two weeks. Protection by expression of the siRNA was shown to be efficient. Currently a comparison of stable cell line development programs based on siRNA selection and neomycin selection is ongoing.

**Conclusions**

The novel selection system should speed up cell line development, as the system kills rapidly and directly selects for cells transcribing the product gene on a high level. We expect to see more high producers earlier in the process, which will allow for an easier and faster selection in the following steps. siRNA based selection offers great opportunities. By directly selecting based on GoI transcription and not a proxy marker, we expect more relevant cells on a pool level. In addition, the elimination of an antibiotics resistance allows more cellular resources for GoI production. The system offers multiple ways of application, either by enriching wanted, or depleting unwanted cells.

**Acknowledgements**

Austrian BMWFW, BMVIT, SFG, Standortagentur Tirol, Government of Lower Austria and Business Agency Vienna through the Austrian FFG-COMET- K2.

**References**

1. Bandaranayake AD, Almo SC: **Recent advances in mammalian protein production.** *FEBS letters* 2014, 588.2:253-260.

2. Wilson RC, Doudna JA: **Molecular mechanisms of RNA interference.** *Annual review of biophysics* 2013, 42:217-239.

3. Kallehauge TB, Li S, Pedersen LE, Ha TK, Ley D, Andersen MR, Kildegaard HF, Lee GM, Lewis NE: **Ribosome profiling-guided depletion of an mRNA increases cell growth rate and protein secretion.** *Scientific reports* 2017, 7:40388.

**Fig. 1 (abstract P-236).**
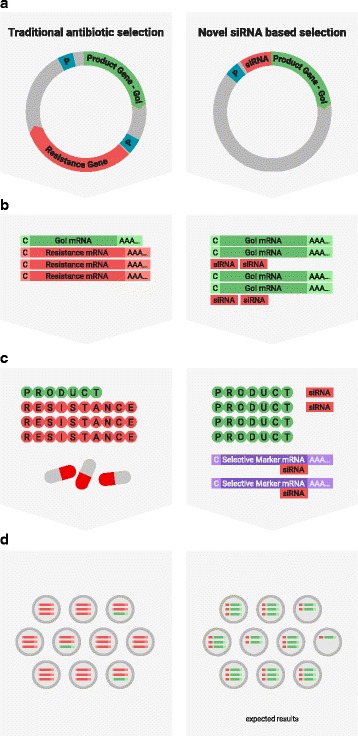
Comparison of cell line development strategies

## P-246 Developing alternatives to Chinese Hamster Ovary (CHO) media for single cell cloning

### Berta Capella Roca, Helena Joyce, Justine Meiller, Padraig Doolan, Martin Clynes

#### National Institute for Cellular Biotechnology, Dublin City University, Dublin, Dublin 9, Ireland

##### **Correspondence:** Berta Capella Roca (berta.capellaroca2@mail.dcu.ie)

**Background**

Single-cell cloning is an essential step used in the upstream development of transformed cell lines for therapeutic protein production. While single-cell clones are typically used to ensure product consistency, such low cell density cultures present a survival challenge; cells grow more slowly or may even not survive at low densities in protein-free media, costing the industry time and money and limiting the pool of candidate colonies for choice of production clones [1,2]. To address this problem, we aimed to develop a highly efficient serum-free medium suitable for optimising single-cell cloning efficiency by studying a range of conditioned media (CM) samples isolated from different Chinese Hamster Ovary (CHO) cell lines.

**Materials and methods**

CHO-S, DG44 and CHO-K1 were adapted to CHO-S SFM-II (Gibco) medium for a minimum of three passages. Conditioned media was then collected when the cultures reached a cell density of 1x10^6^cells/ml (typically day 2-day 3 depending on the growth profiles of each cell line and whether they grew in suspension or attached conditions). Samples were then centrifuged twice to remove cell pellet/debris and stored at -20°C. The ability of conditioned media to support CHO colony formation was then assayed using 96-well plates, seeding the cells at low cell density (1-10cells/well) by diluting down CHO cultures in media/conditioned media. After incubation at 37°C for 10 days, cloning efficiency was assayed using a standard XTT assay. Initial screening of the nine CM samples was performed using CHO-K1 cells due to their widespread use in industrial antibody production. Successful media candidates were subsequently screened using additional CHO cell lines.

**Results**

From the CHO-K1 tests, four conditioned media were identified as supporting improved cloning efficiency when compared to the unconditioned media results: SFM-II media (K1-SFMII-CM) conditioned by suspension CHO-K1; SFM-II media and Poly Vinyl Alcohol (PVA) (DG44-SFMIIPVA-CM) conditioned by suspension DG44; SFM-II media (CHOS-SFMII-CM) conditioned by suspension CHO-S and SFM-II media (ACHOS-SFMII-CM) conditioned by adherent CHO-S.

The highest CHO-K1 cloning efficiency was observed with the CHOS-SFMII-CM, displaying growth in 90% of the wells seeded. The DG44 SFM-II+PVA CM suspension cultures, also improved unconditioned media performance, displaying growth in 21 of the 96 wells seeded.

The K1-SFMII-CM and ACHOS-SFMII-CM are the most well characterized to date, having been tested on two other CHO cell lines: CHO-S and DG44. CM from CHO-S cells growing in SFM-II media adherent cultures (CHOS-SFM-II adherent CM) increased cell cloning efficiencies of several CHO cell lines when compared to SFM-II unconditioned media – (i) CHO-K1: 28-fold increase, (ii) CHO-S: 2-fold increase and (iii) DG44: 6% increase (Table 1).

The K1-SFMII-CM product improved cell cloning efficiency for DG44 cells (avg. increase>1.5-fold) and CHO-S cells (avg. increase>3-fold) (Fig. 1) and also the adherent CHO-K1 cell line growing in ATCC+5%FBS.

**Conclusions**

The ability of conditioned media to support CHO growth in limiting-dilution conditions (1, 6 and 10 cells/ml) was investigated. From a range of nine conditioned media samples; four compelling products have been identified which improve low-cell density growth of CHO-K1 cells, compared to SFM-II control media. We feel that these early-stage conditioned media products may increase cloning efficiencies during upstream CHO cell line development, resulting in financial savings for industry and increasing the possibilities of identifying particularly high-performing transformed clones.

**Acknowledgements**

This research is supported by SFI Grant 13/IA/1841.

**References**

1. Ming Lim U., Gek Sim Yap M., Pin Lim Y., Goh L., and Kong Ng S.: **Identification of autocrine growth factors secreted by CHO cells for applications in single-cell cloning media.** *J Proteome Res* 2013, **vol 12(7)**:3496-3510.

2. Zhu J, Wooh JW, Hou JJ, Hughes BS, Gray PP, Munro TP.: **Recombinant human albumin supports single cell cloning of CHO cells in chemically defined media.** *Biotechnol Prog* 2012, **vol 28(3)**: 887-891.

**Table 1 (abstract P-246).** Number of colonies observed in CHO-K1, DG44 and CHO-S limiting-dilution cultures in CHO-S SFM-II adherent conditioned media (ACHOS-SFMII-CM) compared to SFM-II unconditioned media (SFMII Ctl)

	SFMII Ctl	ACHOS-SFMII-CM	Fold change
CHO-K1	1.5+/-0.71	42+/-1.41	28
DG44	31+/-3.06	33+/-0.24	1.06
CHO-S	23+/-0.71	46+/-2.12	2

**Fig. 1 (abstract P-246).**
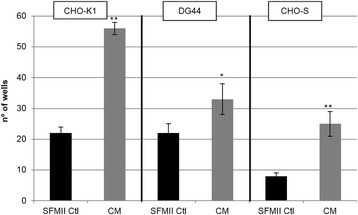
Number of colonies observed in CHO-K1, DG44 and CHO-S limiting-dilution cultures in CHO-K1 SFM-II suspension conditioned media (K1-SFMII-CM) compared to SFM-II unconditioned media (SFMII Ctl).* p≤0.05, ** p ≤0.01

## P-247 Expediting upstream stages of protein biomanufacture through the use of Ubiquitous Chromatin Opening Elements – UCOE®s

### Bethany McCloskey^1^, Joe Orlando^2^, Kimberley Mann^2^, Michael Antoniou^1^

#### ^1^Deapartment of Medical and Molecular genetics, Kings College London, London SE1 9RT, UK; ^2^MilliporeSigma, Process solutions, Bedford, MA, 01730, USA

##### **Correspondence:** Bethany McCloskey (bethany.mccloskey@kcl.ac.uk)

**Background**

The main rate-limiting step in the upstream stages of protein biomanufacture is the isolation of stable, high producing cell clones. Ubiquitous Chromatin Opening Elements (UCOE®s) consist of at least one promoter region with associated methylation-free CpG island from housekeeping genes; they possess a dominant chromatin opening capability and thus confer stable transgene expression. UCOE®-viral promoter (e.g. CMV) based plasmid vectors markedly reduce the time it takes to isolate high, stably producing cell clones. Although some UCOE®-viral promoter combinations have been tested, they have not been thoroughly evaluated in Chinese hamster ovary (CHO) cells.

**Material and method**

Plasmid vectors containing combinations of either the human HNRPA2B1-CBX3 UCOE® (A2UCOE®) or murine Rps3 UCOE® linked to different viral promoters (hCMV, gpCMV, SFFV) driving expression of an eGFP reporter gene were functionally analysed by stable transfection into CHO-K1 cells and expression analysed by flow cytometry and qPCR to determine vector copy number.

**Results and discussion**

The results at 21 days post-transfection and selection clearly indicate that the Rps3 UCOE®-gpCMV and -hCMV combinations give the highest transgene expression as shown in Fig. 1. The A2UCOE®-hCMV/gpCMV constructs were the next efficacious but 2-fold lower than the Rps3 UCOE® vectors. The SFFV promoter linked with either of the two UCOE®s was the least effective with expression levels 17-fold lower than the Rps3-CMV constructs. The Rps3 UCOE®-gpCMV/hCMV constructs are now being further modified to include elements that will provide optimal post-transcriptional pre-mRNA processing (splicing, polyadenylation, transcription termination, mRNA stability) thereby maximising stable cytoplasmic transgene mRNA levels and protein production.

**References**

1. Neville JJ, Orlando J, Mann K, McCloskey B, Antoniou MN. (2017) Ubiquitous Chromatin-opening Elements (UCOEs): Applications in biomanufacturing and gene therapy. *Biotechnol Adv*. **35**: 557-564.

**Fig. 1 (abstract P-247).**
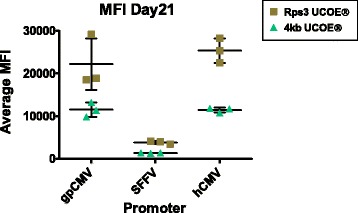
Medium fluorescence intensity data comparing UCOE®s and promoters

## P-248 New pool generation process CLD 2.0

### Kristin Thiele, Caroline Hauser, Juliana Schubert and Christoph Zehe

#### Sartorius Stedim Cellca GmbH, 88471 Laupheim, Baden-Württemberg, Germany

##### **Correspondence:** Kristin Thiele (kristin.thiele@sartorius-stedim.com)

**Background**

In the last 20 years, growing number of innovator biologics and biosimilars have formed a competitive environment, where speed and efficiency of generating robust and highly productive cell lines needs to be improved continuously.

Through various advances, especially in media development and process optimization, product titers as high as 10 g/L were achieved in the pharmaceutical industry (Kim et al., 2012) for standard products such as monoclonal antibodies. Nevertheless, other proteins e.g. bispecific antibodies, Fc-fusion proteins or Fab-related products are difficult-to-express (DTE) in Chinese Hamster Ovary (CHO) and may result in delays or even in termination of the cell line development process. We developed a new robust pool generation approach (CLD 2.0) addressing both, easy- and difficult-to-express molecules, while reducing timelines down to 5 months (CLD standard = 6 months), improving reliability of cell line development as well as clearly increasing obtained titers.

**Materials and methods**

In order to create stable cell lines, we transfected our CHO DG44 host cells by electroporation. Cells processed using the standard approach were cultivated in selective medium or medium containing additional 2.5 nM methotrexate (MTX) for three weeks. After an amplification step with 30 nM MTX for three weeks, stable individual cell pools were expanded and clones were generated by FACS-sorting. Clones were analyzed for growth performance and product concentration in fed-batch studies.

In our new CLD 2.0 approach, we increased MTX concentrations (2.5 nM, 5 nM and 10 nM MTX) during the first selection phase of three weeks. Afterwards we omitted the 30 nM MTX amplification step. Thereby, pool generation finished four weeks earlier than in the standard approach.

To evaluate the stability of cell clones derived from Mini Pools (MPs) generated according to the CLD 2.0 approach, stability studies were performed for eight weeks, including stability fed batches at t=2 weeks and t=8 weeks. Altogether three different proteins of interest with six cell clones each were tested.

**Results**

We adapted our cell line development process by increasing the initial selection during the first selection phase, thereby allowing the omission of the 30 nM MTX amplification step. We observed that the capacity of amplifiability varied for different products. Cell lines with a protein titer ranging from >1 g/L to 1.5 g/L (DTE) in shake flask fed-batch showed to be more susceptible to increased initial MTX levels and were thus not amplifiable with 30 nM MTX. In contrast, cell lines with high protein titer >1.5 g/L were observed to adapt to 30 nM MTX easily and were amplifiable.

Finale shake flask fed-batch data with CLD 2.0 clones of high-expressing products showed comparable titers to clones from the standard approach. CLD 2.0 clone titers for DTE proteins revealed in average a 2.0- fold increase compared to clones generated in the standard approach. Titers of top producing clones were in a range of 1.8 g/L to to 2.7 g/L (Fig. 1). Furthermore, stability data of CLD2.0 cell clones from different DTE products showed a stable specific productivity in a range of +/- 15 % over eight weeks cultivation. Fed-batch titer from t=2 weeks and t=8 weeks were in a normal range of +/-20% of the standard 30 nM projects.

**Conclusions**

Our results demonstrate that CLD 2.0 is a robust and reliable process for standard products (mAb) and DTE proteins. With our new process, we were able to increase titer of difficult-to-express proteins up to 200%. By omitting the amplification step (30 nM MTX) 96 % of generated clones were stable over eight weeks cultivation time. Additionally using the CLD2.0 approach, the time line from DNA to RCB was reduced to 5 months.

**Fig. 1 (abstract P-248).**
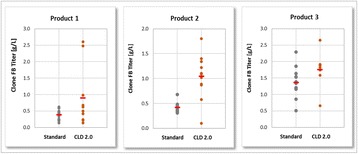
Final shake flask fed-batch titers [g/L] of top 10 clones of 3 difficult–to-express products

## P-256 Identification of integration sites of CHO Genome for the generation of high producer cells by CRISPR/Cas9 mediated target integration

### Dalton Chen^1^, Hsueh-Lin Lu^1^, Ching-Jen Yang^1^, Chi-Chen Hsu^1^, Sheng-Jie Huang^1^, Hsin-Lin Lu^1^, Chien-I Lin^1^, Wei-Kuang Chi^1^, Hsuan-pu Chen^2^

#### ^1^Bioengineering Group, Development Center for Biotechnology, New Taipei City, Taiwan, Republic of China; ^2^Fountain Biopharma Company, Taipei, Taiwan, Republic of China

##### **Correspondence:** Dalton Chen (dtchen@mail.dcb.org.tw); Wei-Kuang Chi (weikchi@dcb.org.tw)

**Background**

CHO cells have become the most popular platform for production of therapeutic proteins [1]. However, the generation of high-producer cells is a time-consuming and labor-intensive process that requires the screening of large amount of cells to get a clone of high titer and stability. Since the expression titer and stability of clone is highly dependent on the site of integration, we demonstrated a new cell line development strategy by using NGS to identify the integration site and using CRISPR/Cas9 to generate the target integrated high producing cell lines [1,2].

**Materials and Methods**

To identify the high expression sites in the CHO cells, we employed NGS to analyze the integration sites of a high producing cell line (titer > 3g/L). The pair-end reads with one read mapped to the vector and the other read mapped to the CHO reference genome are extracted to identify the integration sites. To test the expression activity of the integration sites, we employed CRISPR/Cas9 to specifically integrate the antibody gene into CHO genome for expression.

**Results**

Our data showed 4 integration sites are in the high producing cell line. Among the 4 integration site, IS1 integration site was tested by CRISPR/Cas9 for target integration of antibody gene for expression. The IS1-target integrated cell pool present higher expression titer than cell pool generated by target integration into other integration sites (Fig. 1a). The single cell clones derived from IS1-target integrated cell pool had low copy number of GOI (Fig. 1b). After normalization with copy numbers, the single cell clones derived from IS1-target integrated cell pool showed high titer per copy (123 ~583 mg/L/copy) (Fig. 1c).

**Conclusions**

This study demonstrated the generation of high-producing cell lines by CRISPR/Cas9 mediated target integration. This approach will cost less time and labor than traditional method. The active integration site will serve as a platform like a cassette player for therapeutic antibody production.

**Acknowledgements**

This study is funded by Ministry of Economic Affairs of ROC.

**References**

1. Fischer S, Handrick R, Otte K. **The art of CHO cell engineering: A comprehensive retrospect and future perspectives.** Biotechnol Adv. 2015 Dec;33(8):1878-96.

2. Inniss MC, Bandara K, Jusiak B, Lu TK, Weiss R, Wroblewska L, Zhang L. **A novel Bxb1 integrase RMCE system for high fidelity site-specific integration of mAb expression cassette in CHO Cells**. Biotechnol Bioeng. 2017 Aug;114(8):1837-1846.

**Table 1 (abstract P-256).** Integration sites of a high-producing cell line was determined by whole genome sequence with 15X coverage

Cell line	Titer	Integration sites	Location
3G7	2.5 g/L (fed batch) 175 mg/L (batch)	IS1	promoter
		IS2	upstream(~4k)
		IS3	intron
		IS4	promoter

**Fig. 1 (abstract P-256).**
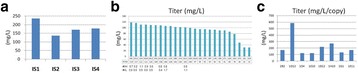
Productivity of target integrated cell pools (**a**), single cell clones (**b**), and titer per copy (**c**)

## P-260 Coupling lipidomics and transcriptomics for characterization of mammalian cell lines

### Yue Zhang^1^, Deniz Baycin^2^, Amit Kumar^1^, Joseph Priola^1^, Kristen Lekstrom^3^, Michael A. Bowen^4^**,** Michael J. Betenbaugh^1^

#### ^1^Chemical and Biomolecular Engineering Department, Johns Hopkins University, Baltimore, MD 21218, USA; ^2^Biotechnology Development Center, Turgut İlaclari, Istanbul, Turkey; ^3^Antibody Discovery and Protein Engineering Department, Medimmune, LLC, Gaithersburg, MD, USA; ^4^Juno Therapeutics, Seattle, WA, USA

##### **Correspondence:** Michael J. Betenbaugh (beten@jhu.edu)

**Background**

CHO, HEK and SP2/0 are the dominant host cells for biologics drug production. Achieving high level of recombinant protein production by these cell lines still remains a challenge. In order to understand the potential roles of lipids in protein production, secretion, vesicular transport and energy metabolism, we coupled high-throughput transcriptomics and lipidomics technologies. Quantitative lipidomics is an emerging ‘omics technology which can help us understand the physiological limitations of each cell line. The two types of major lipid groups in cells are non-polar and polar lipids. Polar lipids such as glycerophospholipids (PLs) include phosphatidylethanolamine (PE), phosphatidylcholine (PC), phosphatidylinositol (PI), phosphatidylserine (PS), phosphatidylglycerol (PG), and phosphatidic acid (PA). In this study; we integrated two dimensional high performance thin layer chromatography (2D-HPTLC) and mass spectrometry (MS) lipid analysis of SP2/0, CHO, and HEK cell lines to understand the major differences in the lipid content of these hosts.

**Materials and methods** 

Bligh-Dyer method was used to extract the lipids and extracts were analyzed by HP-TLC and MS. The polar lipids were separated into different categories by 2-D HP-TLC using a CHCl_3_-MeOH-H_2_O (71:25:2.5, v/v/v) solvent system in the first dimension and a CHCl_3_-MeOH-Acetic acid-H_2_O (76:9:12:2, v/v/v/v) solvent system in the second dimension. Non-polar lipids were separated by 1-D HPTLC using hexane-diethyl ether-acetic acid. 2,7-dichlorofluorescein dye was used to visualize both polar and non-polar lipids. Further detailed analysis was performed on a QqQ mass spectrometer (Thermo TSQ VANTAGE, San Jose, CA) using negative-ion and positive-ion ESI modes as well as negative-ion ESI mode in the presence of lithium hydroxide.

**Results**

In this study, quantitative lipidomics was coupled with transcriptomics to further understand the physiological pathways of HEK, CHO-M and SP2/0 cells. Initial HP-TLC analysis indicated that major lipids in these industrial cell lines were PE and PC. Other polar lipids such as PI, PS, PG, PA, and SM were lower compared to PC and PE in exponential and stationary phases of each cell line. Figure 1 represents 2D HP-TLC results of HEK with the relative quantitation of polar lipids.

In order to investigate the lipid subgroups, shotgun MS analysis was conducted for both exponential and stationary growth phases of the three cell lines. MS analysis indicated that lyso-phosphatidylethanolamine (LPE) and lyso-phosphatidylcholine (LPC) amounts were 4 - 10 fold and 2-4 fold higher in HEK cells compared to SP2/0 and CHO cell lines. Sphingomyelin (SM) was another lipid subgroup that was shown to have a major difference between SP2/0 and other mammalian cell lines. SM was 30-65 fold lower in SP2/0 cell line compared to CHO and HEK. To understand these metabolic differences, transcriptomics analysis using Illumina HighSeq and Gene Expression Omnibus was conducted on these mammalian cells. The Kyoto Encyclopedia of Genes and Genomes (KEGG) database was used to map the transcriptomics data to the lipid synthetic pathways. Transcriptomics data mapping to KEGG pathways demonstrated that differences in LPE and LPC pathways correlate with the expression profiles of secretory phospholipase A2 (sPLA2), lysophospholipid acyltransferase (LPEAT), lysophosphatidylcholine acyltransferase (LPCAT), and lysophospholipase (LYPLA) [1].

**Conclusions**

The HP-TLC and LC/MS findings demonstrated that high levels of LPE and LPC existed in the HEK cell line and low levels of SM were observed in the SP2/0 cell line. Coupling lipidomics with transcriptomics provides us with an improved understanding of the physiological differences across SP2/0, CHO, and HEK cell lines that could be used to guide cell engineering efforts with the goal of increasing the recombinant protein expression capabilities of these three cell lines.

**References**

1. Zhang Y., Baycin Hizal D., Kumar A., Priola J., Bahri M., Heffner K., Wang M., Han X., Bowen M.A., and Betenbaugh M. J.: High-troughput Lipidomic and Transcriptomic Analysis to Compare SP2/0, CHO, and HEK-293 Mammalian Cell Lines, *Anal. Chem.*, 2016, 89(3):1477-1485.

**Fig. 1 (abstract P-260).**
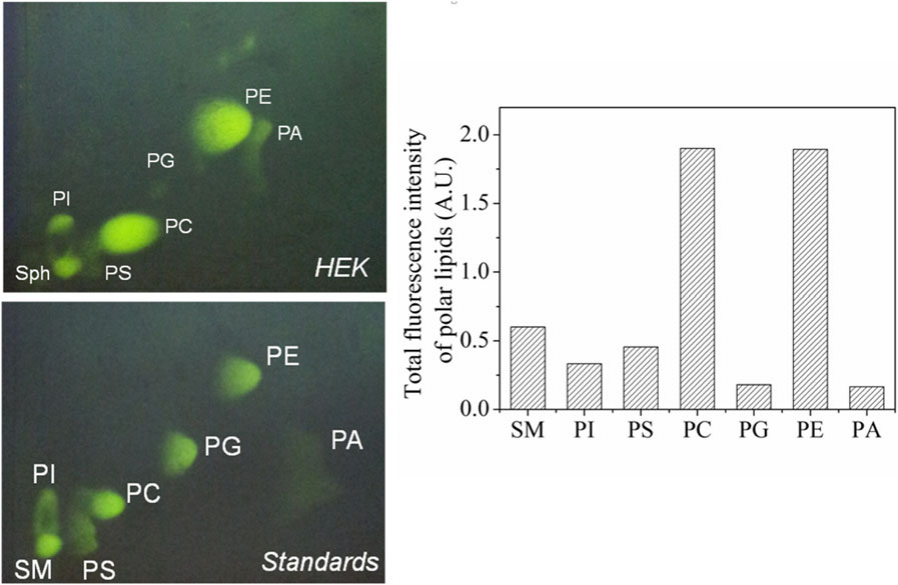
HP-TLC analysis of polar lipids from HEK-293F cell line with a representative fluorescence image (left top) and semi-quantitative results (right). The fluorescence image of standards is shown in the left bottom panel. The mass ratio of PE, PC, PS, PI, PG, PA, SM in standards is 1:1:1:1:1:1:1

## P-263 Single cell characterisation of Chinese hamster ovary (CHO) cells

### Eva Pekle^1,2^, Guglielmo Rosignoli^1^, Andrew Smith^1^, Mark Smales^2^, Christopher Sellick^1^, Claire Pearce^1^

#### ^1^MedImmune, Cambridge, Cambridgeshire, CB21 6GH, UK; ^2^School of Biosciences, University of Kent, Canterbury, Kent, CT2 7NJ, UK

##### **Correspondence:** Eva Pekle (peklee@medimmune.com)

**Background**

Biopharmaceuticals are a class of biological macromolecules that include antibodies and antibody derivatives, generally produced from cultured mammalian cell lines via secretion directly into the media. Manufacturing at MedImmune requires the generation of Chinese hamster ovary (CHO) clonal cell lines capable of producing the biopharmaceutical product at commercially relevant quantities with optimal product quality. The isolation of cell clones based on random single cell deposition via fluorescence activated cell sorting (FACS) provides a heterogeneous panel of expressers. We hypothesize that the application of FACS to provide an additional sorting step based on desirable cell attributes that correlate with productivity, product quality or cell growth attributes could lead to the isolation of higher producing cell lines with enhanced product quality attributes.

**Materials and methods**

A panel of 20 cell lines expressing a model recombinant monoclonal antibody were characterised in terms of growth, productivity, intracellular recombinant protein and mRNA amounts. Assays were also developed to investigate cell attributes using the commercially available ImageStream instrument, an imaging flow cytometer, which enables the investigation of cellular characteristics that correlate with cell productivity at the single cell level.

**Results**

Characterisation revealed the cell lines exhibited a range of values for productivity, growth, and intracellular (IC) antibody mRNA and protein expression, ideal for further ImageStream characterisation. Western blot and qRT-PCR analysis demonstrated that final titre correlated with both IC heavy chain (HC) protein and mRNA amounts (Pearson Correlation Coefficient (PCC) = 0.70 and PCC = 0.80, respectively).

To assess productivity at the single cell level, assays multiplexing IC HC protein and mRNA with cell attributes were therefore developed. Initial assay development focusing on HC mRNA and protein amounts has revealed interesting results; four cell lines displayed two distinct populations, one producing the antibody and another non-expressing population. The ratio of these populations varied amongst the cell lines. Images obtained from the ImageStream have shown the cellular localization and expression of HC and LC message and protein (Fig. 1). For both message and protein, HC and LC colocalize in the cell.

Whether there is any relationship between IC HC protein and cell attributes at the single cell level was then also investigated, as well as correlations with cell culture parameters at the population level. At the population level, correlations were found between titre and IC HC protein and mRNA (PCC = 0.84 and PCC = 0.79, respectively) confirming the data obtained by western blot and qRT-PCR analysis.

**Conclusions**

A panel of 20 cell lines has been characterised at the population level and show a wide range of antibody expression profiles at both the mRNA and protein level. In parallel, assays have been developed for the ImageStream to measure HC and LC message and protein amounts at the single cell level. Protein and message quantification with the ImageStream are consistent with more traditional approaches, such as western blots and qRT-PCR, that operate at the population level. The developed assays are now being used to investigate single cell productivity attributes and for the isolation of more productive clones.

**Acknowledgements**

The authors would like to thank Gary Pettman, Charlotte Godfrey, Sarah Dunn, Alison Mason, Lina Chakrabarti, Gareth Davies and Michele Garrett.

**Fig. 1 (abstract P-263).**
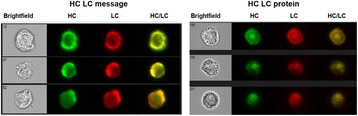
ImageStream HC and LC protein and mRNA localisation and expression. HC and LC message (left) and protein (right) sample images acquired with an ImageStream. An overlay of HC and LC is shown in each case

## P-268 Development of a transposon-mediated integration system to generate high yield producing cells with low copy number of integrated target antibody gene

### Hsin-Lin Lu^1^, Han-Yuan Liu^1^, Bor-Shiun Chen^1^, Ying-Ju Chen^1^, Chien-I Lin^1^, Hsuan-Pu Chen^2^, Wei-Kuang Chi^1^

#### ^1^Bioengineering Group, Institute of Biologics, Development Center for Biotechnology, New Taipei City, Taiwan; ^2^Fountain Biopharma Company, Taipei, Taiwan

##### **Correspondence:** Hsin-Lin Lu (hllu9999@dcb.org.tw); Wei-Kuang Chi (weikchi@dcb.org.tw)

**Background**

Productivity and stability are key factors for the selection of cell line in protein drugs production. Large amount of target gene integrated in cell genome could lead to the instability of production. Therefore, cells with low copies of target gene integrated in high yield sites could be an ideal production cells for manufacturing. It has been known that the transposon system can control the integrated copy number of target gene and can generate high yield producing cells, it could be a great approach to generate stable high yield producing cell lines carrying low copies of target gene through transposon system.

**Materials and methods**

We intended to develop a platform to generate high yield producing cell lines carrying 1-2 copy of the integrated target gene using transposon system. Two CHO cell lines, CHO-S cells and DXB11 cells, have been applied. Cells were co-transfected with transposon and target gene expression plasmids. After drug selection, the cell pool with highest productivity per target gene copy was applied to single cell cloning. The productivity and copy number of cell clones were determined, and the stability of cell clones was analysed after culture of about 60 generations.

**Results**

In the stable pools of CHO-S and DXB11 cells, the productivities per integrated target gene copy were about 11-13 mg/L/copy and 68-75 mg/L/copy in a batch culture, respectively. After single cell cloning, the integrated copy numbers in most cell clones were less than three copies per cell. In CHO-S and DXB11 cell clones, the productivities per integrated target gene copy were 20-60 mg/L/copy and 60-150 mg/L/copy in a batch culture, respectively. The productivity per integrated target gene in cell clones developed by the transposon system was much higher than that in cell clones developed by random integration (Fig. 1a and b). To evaluate the productivity stability of cell clones developed by the transposon system, ten cell clones at generation 0, 30, 60, and 100 were applied in the analysis. Of interest, about 80% of cell clones were stable at generation 60, but lost the productivity at generation 100 (Fig. 1c), implying the most cell clones could maintain the stability within 2 months.

**Conclusion**

Using the optimized conditions of the transposon system to develop the stable gene expression cells, the productivity per integrated target gene was higher than random integration. These results suggested that our platform is capable to develop high yield producing cells with 1-2 copy of integrated target antibody gene and can be applied to identify high yield integration sites.

**References**

1. Rajendra Y, Peery RB, Barnard GC: **Generation of stable Chinese hamster ovary pools yielding antibody titers of up to 7.6 g/L using the piggyBac transposon system.** *Biotechnol Prog.* 2016, **32**: 1301-1307.

2. Balasubramanian S, Rajendra Y, Baldi L, Hacker DL, Wurm FM: **Comparison of three transposons for the generation of highly productive recombinant CHO cell pools and cell lines.** *Biotechnol Bioeng*. 2016, **113**: 1234-1243.

**Fig. 1 (abstract P-268).**
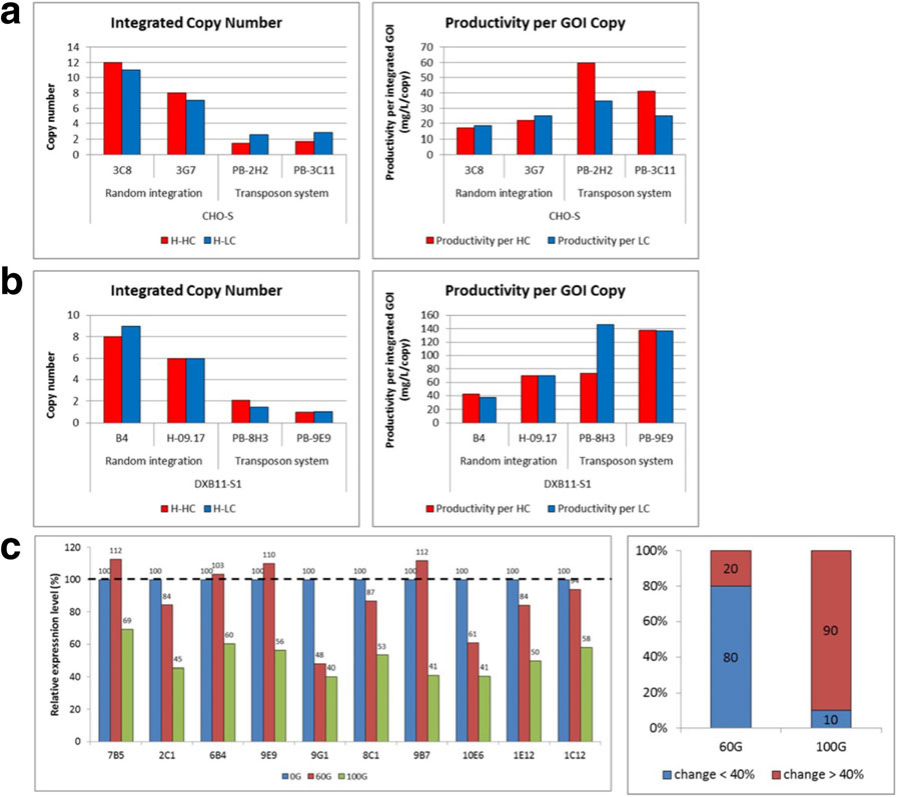
The productivity per integrated gene of interest (GOI) and the stability of cell clones developed by the transposon system. **a** Comparison of the integrated copy number of GOI and the productivity per integrated GOI copy between cell clones developed by random integration and transposon-mediated integration in CHO-S cells. **b** Comparison of the integrated copy number of GOI and the productivity per integrated GOI copy between cell clones developed by random integration and transposon-mediated integration in DXB11 cells. **c** The stability of antibody production in DXB-11 cell clones developed by the transposon system

## P-272 Comparison of glucose-lactate metabolism of three different mammalian cell lines using Flux Balance Analysis

### Iván Martínez-Monge^1^, Pere Comas^1^, Joan Triquell^1^, Joan Albiol^1^, Carles Solà^1^, Antoni Casablancas^1^, Martí Lecina^1,2^, Carles Paredes^1^ and Jordi Joan Cairó^1^

#### ^1^Department of Chemical, Biological and Environmental Engineering, Autonomous University of Barcelona, Bellaterra (Cerdanyola del Vallès), 08193, Spain; ^2^Bioengineering Department, IQS-Universitat Ramon Llull, Barcelona, 08017, Spain

##### **Correspondence:** Iván Martínez-Monge (ivan.martinez.monge@uab.cat)

**Background**

Mammalian cells show an inefficient metabolism characterized by high glucose uptake and the production of high amounts of lactate, a widely known growth inhibition by-product [1]. Recently, we have observed a different glucose-lactate metabolism in some cell lines. While some cell lines are unable to metabolize lactate, others can co-metabolize simultaneously glucose and lactate under certain culture conditions, even during the exponential growth phase [2]. These metabolic differences between different mammalian cell lines (CHO, HEK293 and hybridoma) have been studied by means of Flux Balance Analysis (FBA).

**Materials and methods**

Three different cell lines were cultured in a 2-liter bioreactor: CHO-S, HEK293SF and hybridoma KB-26.5. For the FBA, two adapted genome-scale metabolic models were used: a reconstruction of *Mus musculus* for CHO and hybridoma [3], and a reconstruction of Human metabolic model (Recon 2) for HEK293 [4].

**Results**

In cultures where pH was not controlled, two different metabolic phases were observed for CHO and HEK293 cells. During the first phase both cell lines produced large amounts of lactate as a consequence of the high glucose consumption rates. Interestingly, when pH dropped below 6.8, due to acid lactic secretion and accumulation, a second metabolic phase was identified, in which concomitant consumption of glucose and lactate was observed even during the exponential growth phase. Conversely, hybridoma cells were unable to co-consume lactate and glucose simultaneously even under non-controlled pH conditions. Therefore, the hybridoma physiological data used for the FBA corresponded to only phase 1 of pH-controlled cultures. A summary of the main cell growth and metabolic parameters obtained from the different experiments performed is presented in Table 1.

FBA shows (Fig. 1 for HEK293 cell culture) that lactate is produced in phase 1 because pyruvate has to be converted to lactate to fulfill the NADH regeneration in the cytoplasm and only a small amount of pyruvate can be transported into TCA through Acetyl-CoA. Cell metabolism in phase 1 is highly inefficient, as the majority of the carbon source is not used for the generation of energy nor biomass. In phase 2, in which mitochondrial LDH was considered, TCA fluxes could be maintained as in phase 1 at the maximal rate encountered; hence, the energy available for cells to grow was similar in both phases, obtaining similar growth rate.

**Conclusions**

Two different glucose and lactate metabolism behaviors have been observed in CHO and HEK293 cultures depending on the culture conditions: phase 1) glucose consumption and lactate production, and phase 2) glucose and lactate simultaneous consumption. In contrast, only phase 1 was observed in hybridoma cultures even when pH was non-controlled. FBA showed that TCA fluxes in phase 1 and phase 2 were similar, obtaining similar cell growth rate, but glucose uptake rate was much lower in phase 2 due to the lactate co-consumption. Some authors hypothesize that cells metabolize extracellular lactate as a strategy for pH detoxification [2]. Glucose and lactate co-metabolization resulted in a better-balanced cell metabolism, as can be seen from the metabolic fluxes calculated, with minor effects on cell growth. The observation of glucose and lactate co-consumption metabolic behavior and its deeper study and characterization could open the door of novel culturing strategies with the aim of increasing bioprocesses productivity.

**Acknowledgements**

The authors would like to mention that this research was supported by the FI-DGR (2017) from Spanish Government and the project was led by Prof. Jordi Joan Cairó Badillo.

**References**

1. Ozturk, S. S., Riley, M. R., & Palsson, B. O. (1991). Effects of ammonia and lactate on hybridoma growth, metabolism, and antibody production. *Biotechnology and bioengineering*, *39*(4), 418-431.

2. Liste-Calleja, L., Lecina, M., Lopez-Repullo, J., Albiol, J., Solà, C., & Cairó, J. J. (2015). Lactate and glucose concomitant consumption as a self-regulated pH detoxification mechanism in HEK293 cell cultures. *Applied microbiology and biotechnology*, *99*(23), 9951-9960.

3. Martínez, V. S., Dietmair, S., Quek, L. E., Hodson, M. P., Gray, P., & Nielsen, L. K. (2013). Flux balance analysis of CHO cells before and after a metabolic switch from lactate production to consumption. *Biotechnology and bioengineering*, *110*(2), 660-666.

4. Quek, L. E., Dietmair, S., Hanscho, M., Martínez, V. S., Borth, N., & Nielsen, L. K. (2014). Reducing Recon 2 for steady-state flux analysis of HEK cell culture. *Journal of biotechnology*, *184*, 172-178.

**Table 1 (abstract P-272).** Summary of the parameters related to cell physiology calculated for the different mammalian cell lines from experiments performed in a 2L-Bioreactor

	CHO-S		HEK293		Hybridoma	
	Phase 1	Phase 2	Phase 1	Phase 2	Phase 1	Phase 2
μ_max_ (h^-1^)	0.039	0.038	0.023	0.015	0.027	-
X_max_ (10^6^cells·mL^-1^)	4.1	8.5	3.0	14.0	2.0	-
pH range	7.5-6.8	6.8-7.3	7.2-6.8	6.8-6.9	7.0	-
q_glucose_ (nmols·mgDW^-1^·h^-1^)	-343.120	-77.573	-354.000	-79.000	-886.640	-
q_lactate_ (nmols·mgDW^-1^·h^-1^)	555.290	-87.608	600.000	-59.000	1639.560	-

**Fig. 1 (abstract P-272).**
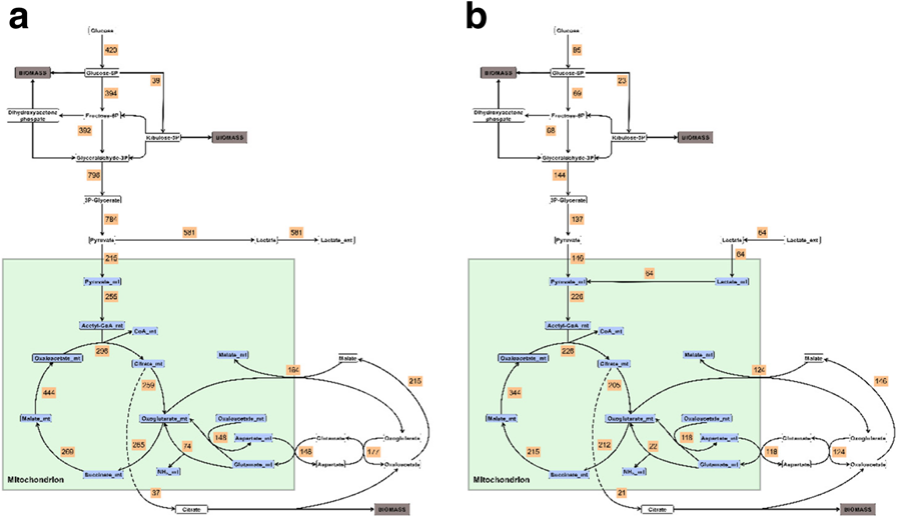
Flux Balance Analysis performed in phase 1 (glucose consumption and lactate secretion) (**a**) and phase 2 (glucose and lactate simultaneous consumption) (**b**) for HEK293 cells. Only the relevant fluxes for the discussion are depicted. Fluxes units are in nmols·mg_DW_^-1^·h^-1^. The ATP generation was maximized in order to calculate the fluxes through the metabolic network

## P-274 Robust and reliable transient protein production with PEIpro®, a well characterized polyethylenimine optimized for transfection

### Mathieu Porte, Valérie Moreau-Toussaint, Julien Depollier, Jonathan Havard, Valérie Kédinger, Géraldine Guérin-Peyrou, Cindy Croizier, Fabrice Stock, Alengo Nyama’antu, Patrick Erbacher

#### Polyplus-transfection, Bioparc,850 Boulevard S. Brant, 67400 Illkirch, France

##### **Correspondence:** Mathieu Porte (mporte@polyplus-transfection.com)

**Background**

Transient protein expression in mammalian cell lines has gained increasing relevance as it enables fast and flexible production of high-quality eukaryotic protein. Considerable efforts have thus been made to overcome existing limiting aspects of transient gene expression systems, in terms of cell lines, cell culture-based systems, and protein production in a cost-effective manner. Milligram amounts of protein per liter can be produced within several days, allowing a significant shortening of the bioproduction process in comparison to protein production from stable clones. To ensure the robustness of the process, it is essential to have a reliable and easy-to-use transfection method.

To palliate for the need of a reliable transfection reagent, we developed PEIpro®, the only commercially available PEI optimised for mid to large-scale transient protein production during process development. PEIpro® is a non-polydiperse and fully-characterised polymer that has become the gold PEI standard due to its reliability, reproducibility in high DNA delivery efficiency and in ensuing high protein production yields.

Here, we present experimental data showing the benefits of using PEIpro® for protein production in comparison to other PEIs. We further demonstrate compatibility of using PEIpro® for recombinant protein production in most commonly used chemically-defined media.

**Materiel and methods**

Suspension HEK-293 and CHO cells were cultured in shaker flasks in various synthetic media, as listed in Table 1. HEK-293 and CHO cells were resuspended at 1×10^6^ cells/ml of serum-free medium, on the day before transfection. Cells were transfected with 0.5-1 mg of plasmid DNA encoding for the Luciferase gene reporter using PEIpro®, PEI “Max” and L-PEI 25 kDa (Polysciences, Warrington, PA) resuspended at 1 mg/ml according to the manufacturer’s recommendation. Protein expression of the luciferase reporter gene was assayed 48 hours post-transfection by affinity chromatography using protein G (HPLC).

**Results**

Comparison of PEIpro® to other commercially available PEIs was achieved by transfecting suspension HEK-293 and CHO cells with plasmid DNA encoding for the luciferase gene reporter. Luciferase production yields obtained in HEK-293 and CHO cells were at least respectively 5-fold and 10-fold higher when using a similar amount of PEIpro in comparison to the other PEIs (Fig. 1).

Furthermore, PEIpro® was the only PEI that led to similar luciferase production yields when decreasing the amount of plasmid DNA per Liter of cell culture. Conversely, at least 1 mg of plasmid DNA and 4-fold more of PEI “Max” and L-PEI 25 kDa were needed to obtain a similar Luciferase expression range in both HEK-293 and CHO cells.

We further assessed the compatibility and versatility of PEIpro® by measuring protein production yields obtained in most commonly used animal-free synthetic media. As shown in Fig. 2, PEIpro® leads to high protein production yieds in several commercially avaialble media formulations for HEK-293 anc CHO cell lines.

**Conclusion**

PEIpro® is the only fully characterised PEI transfection reagent that is suitable for reliable and reproducible recombinant protein production, irrespective of the scale of production and of the type of adherent and suspension cell culture system.

**Fig. 1 (abstract P-274).**
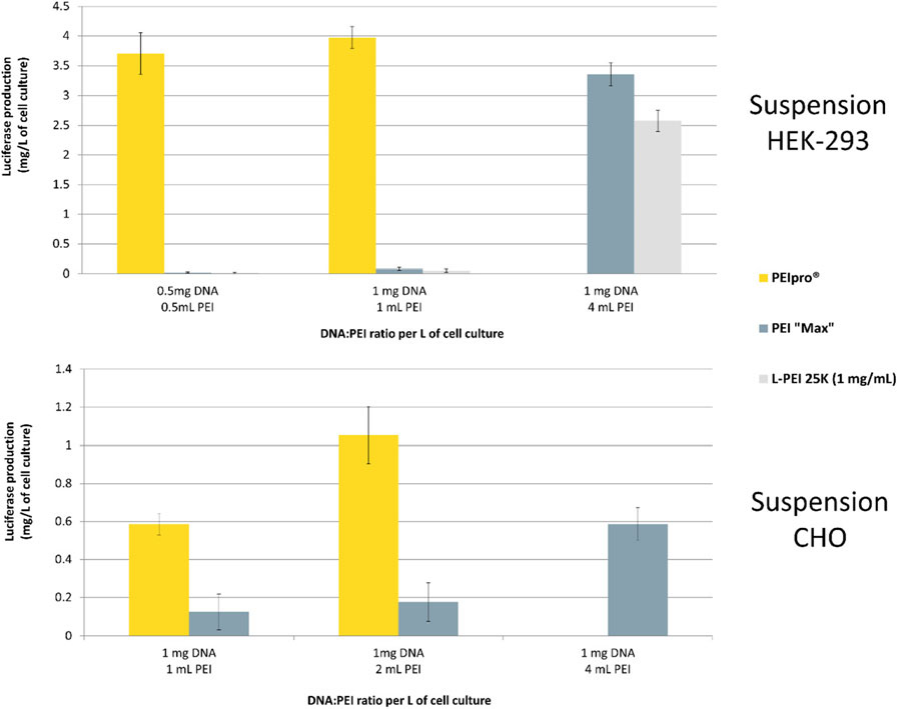
PEIpro® requires less reagent and similar to lower DNA amount compared to other PEIs. Suspension HEK-293 and CHO cells were seeded at 1×10^6^ cells/mL in serum free medium and transfected with PEIpro®, PEI “Max” and L-PEI 25 kDa (Polysciences, Warrington, PA) resuspended at 1 mg/ml. Luciferase expression was assayed 48 h after transfection using a conventional luciferase assay

**Fig. 2 (abstract P-274).**
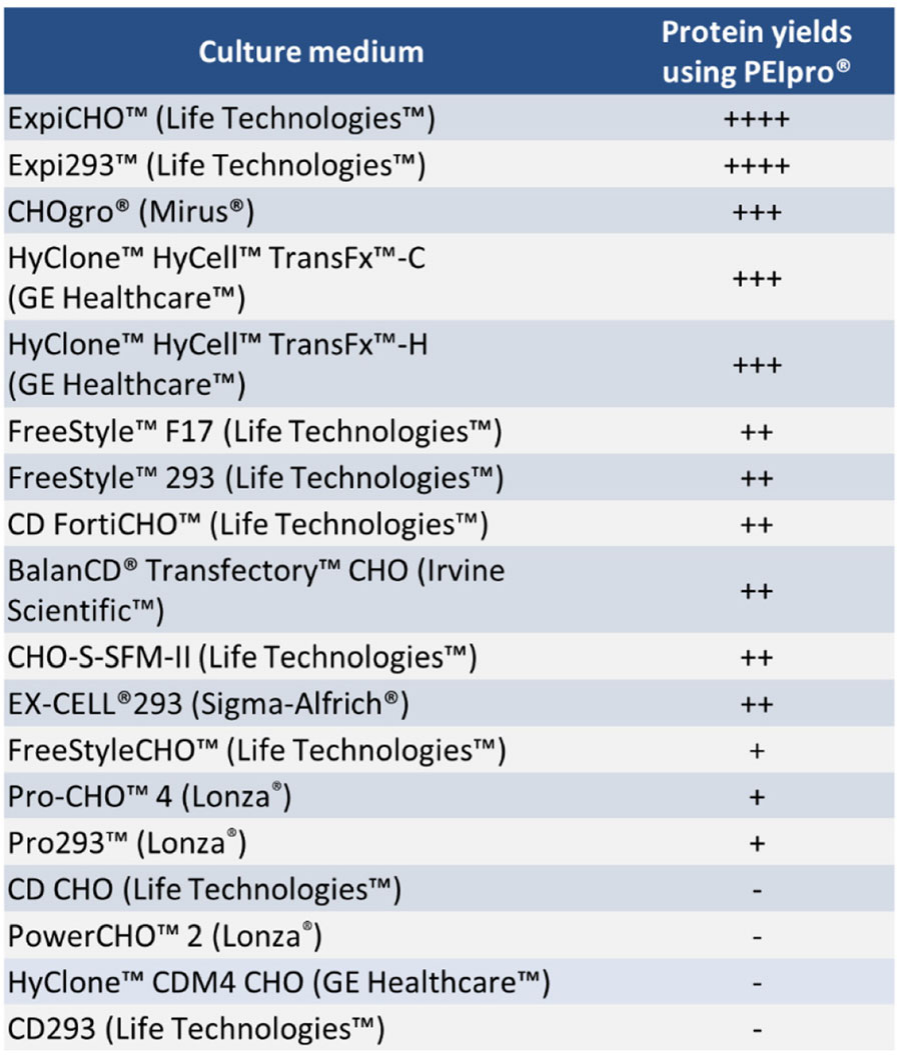
PEIpro® is optimized for transfection of HEK-293 and CHO cells in several specific synthetic culture media. Suspension HEK-293 and CHO cells were seeded following the recommended protocol in serum-free media and transfected with PEIpro® using the standard conditions. IgG3-Fc production was assayed 48 h after transfection using protein G affinity quantification (HPLC)

## P-276 Comparison of expression strategies based on bicistronic and tricistronic vectors for Trastuzumab and Trastuzumab-interferon-α2b production in CHO and HEK293 cells

### Joan Miret^1^, Ramón Román^1^, Aïda Roura^1^, Cristina Moreno^1^, Guillem Arboix^1^, Mercè Farràs^2^ Daniela Cancelliere^3^, Claudia Di Gesù^3^, Antoni Casablancas^1^, Martí Lecina^1,4^, Jordi Joan Cairó^1^

#### ^1^Department of Chemical, Biological and Environmental Engineering, Universitat Autònoma de Barcelona, Barcelona, 08193, Spain; ^2^Department of Biotechnology, Farmhispania SA, Montmeló, 08160, Spain; ^3^School of Medicine and Surgery, University of Palermo, Palermo, 90133, Italy; ^4^IQS School of Engineering, Universitat Ramon Llull, 08017, Barcelona, Spain

##### **Correspondence:** Joan Miret (joan.miret@uab.cat)

**Background**

Monoclonal antibodies (MAbs), which are widely used in anticancer therapies, are mainly produced by mammalian cell lines. MAb conjugation to biological molecules for enhancing their antitumor activity offers a new powerful tool for anticancer therapies. We have assessed the production of commercially approved anti-HER2 therapeutic antibody Trastuzumab (Tzmb) [1] and also its fusion with interferon-α2b (IFNα2b). Two cloning strategies consisting in transfecting CHO-S and HEK293 cell lines with two bicistronic or with a single tricistronic plasmids have been assessed. The *in vitro* efficacy of both antibodies has been tested and compared side by side.

**Matherials and methods**

Tzmb heavy and light chains were cloned in two bicistronic plasmids (pIRESpuro3 and pIRESneo3, Clontech) and in a tricistronic plasmid derived from pIRESpuro3. IFNα2b was spliced to Tzmb heavy chain by overlap extension PCR and the resulting Tzmb-IFNα2b fusion protein was also cloned in the expression vectors in the same way than non-modified Tzmb.

Selected cell pools were cultured in 125 ml shake flasks containing SFMTransfx supplemented with 10% v/v of Cell Boost 5 (HyClone), 4 mM of GlutaMAX (Gibco) and 2 μg/ml of puromycin and also with 700 μg/ml neomycin in the case of the cells transfected with pIRESneo3. Cells were cultivated in the same conditions as described elsewhere [2]. Purified products (using protein A chromatography (HiTrap MabSelect Sure, ÄKTA Avant 150)) were quantified by both ELISA and SDS-PAGE. Antigen binding test was performed in Sk-br-3 breast cancer cell line by means of flow cytometry analysis. The biological activity of the different candidates was tested with MTT assay.

**Results**

Both Tzmb and the fusion protein Tzmb-IFNα2b have been successfully expressed in CHO-S and HEK293, which use for heterologous protein expression have previously been optimized in prior works [3]. The tricistronic strategy resulted in the most efficient, showing a 3.5-fold increase in terms of productivity with respect to the bicistronic double-transfection for Tzmb in CHO-S cells and a 5-fold increase in HEK293 cells (Fig. 1a). In the case of Tzmb-IFNα2b, the tricistronic strategy also allowed to achieve higher productivities than the bicistronic one (Fig. 1b). Regarding the differences of specific productivity between both cell lines tested, HEK293 emerged as the best production host candidate, for the two tested strategies (tricistronic and bicistronic) and for the two produced proteins, showing a 1.5-fold increase in terms of productivity with respect to CHO-S cells for Tzmb using the tricistronic strategy.

Tzmb and Tzmb-IFNα2b were analysed in terms of their antigen binding capacity, and both were find to efficiently bind to HER2+ Sk-br-3 cells (Fig. 1c). Thus, the antibody affinity to HER2 antigen has not been affected when fused to INF-α2b. Finally, antiproliferative activity of Tzmb and Tzmb-IFNα2b were assessed on the same Sk-br-3 cells. At a concentration of 500 nM of Tzmb, and after a 72-hour incubation, Sk-br-3 cells presented a 83% growth with respect to the untreated control. However, no antiproliferative effect was observed for Tzmb-IFNα2b (Fig. 1d).

**Conclusions**

The tricistronic strategy provides higher productivity yields in HEK293 and CHO-S cell lines for both recombinant proteins (Trastuzumab and Tzmb-IFNα2b). Regarding which cell line is the best production host candidate, HEK293 achieved higher productivity than CHO-S cells for the two proteins tested. All constructions performed preserved the binding affinity to its antigen, Trastuzumab and Tzmb-IFNα2b bind efficiently to the HER2 antigen present in Sk-br-3 cells. Finally, Tzmb-IFNα2b does not present an improved antiproliferative effect with respect to Trastuzumab when compared by means of an *in vitro* assay.

**References**

1. Clifford A, Hudis MD: **Trastuzumab – Mechanism of action and use in clinical practice**. *N Engl J Med* 2007, 357: 39-51.

2. Liste-Calleja L, Lecina M, Cairó JJ: **HEK293 cell culture media study towards bioprocess optimization: Animal derived component free and animal derived component containing platforms**. *J Biosci Bioeng* 2014, 117(4): 471-477.

3. Román R, Miret J, Scalia F, Casablancas A, Lecina M, Cairó JJ: **Enhancing heterologous protein expression and secretion in HEK293 cells by means of combination of CMV promoter and IFNα2 signal peptide**. *J Biotechnol* 2016, 239: 57-60.

**Fig. 1 (abstract P-276).**
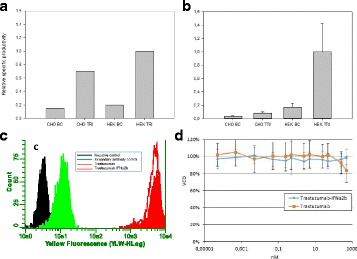
Expression of Trastuzumab (**a**) and Trastuzumab-IFNα2b (**b**) from bicistronic strategy (BC) and tricistronic strategy (TRI) with CHO-S and HEK293 cells. Relative specific productivity units are used for comparing the different strategies. **c** Antigen binding analysis of Trastuzumab and Trastuzumab-IFNα2b. **d** Antiproliferative activity of Trastuzumab and Trastuzumab-IFNα2b on Sk-br-3 cells

## P-279 Production of therapeutically relevant lentiviral vectors for clinical studies

### Johann-Christoph Dettmann^1^, Martin Meyer^1^, Thomas Noll^2^

#### ^1^Miltenyi Biotec GmbH, Bergisch Gladbach, Germany; ^2^Faculty of Technology/AG Zellkulturtechnik, University of Bielefeld, Bielefeld, Germany

##### **Correspondence:** Johann-Christoph Dettmann (johannd@miltenyibiotec.de)

**Background**

The genetic engineering of patient-specific T cells with lentiviral vectors (LVV) expressing chimeric antigen receptors (CAR) for late phase clinical trials requires the large- scale manufacture of high-titer vector stocks. The state-of-the-art production of LVV is based on 10- to 40-layer cell factories transiently transfected in the presence of serum. This manufacturing process is extremely limited by its labor intensity, open-system handling operations, its requirements for significant incubator space plus costs and patience risk due to presence of serum. To circumvent these limitations, this study aims to develop a stable and serum-free process to produce LVV with PEI- mediated transfection. In addition, this study also focuses on the development of a production system not only using a GFP marker but also a therapeutically relevant transgene (CD20-CAR) [1].

**Materials and methods**

Therefore, three different cell lines (HEK 293, 293T, 293FT) were investigated concerning their productivity of LVV and their growing behavior in the in-house serum-free medium TransMACS. As part of this, Design of Experiment was used to investigate the optimal conditions for PEI/DNA-transfection. Furthermore, this statistical approach was used focusing an ideal ratio between the 3rd generation plasmids (transfer plasmid CD20-CAR or GFP, envelope plasmid, packaging plasmids). In addition, different enhancers (sodium butyrate, lithium acetate, caffeine, trichostatin A, cholesterol, hydroxyurea, valproic acid) were investigated concerning their effects on productivity comparing HEK cultures producing LVV encoding for GFP-marker or CD20-CAR.

**Results**

Concerning productivity and growing behavior, HEK 293T was the favored cell line for our serum-free LV manufacturing process. In addition, an additive screen revealed that sodium butyrate alone had the most promising effect on both GFP-LVV and CD20-CAR-LVV production. After PEI/DNA titration, we finally could increase LVV productivity by lowering PEI/DNA amount at higher cell densities referred to our standard transfection protocol. Furthermore, the titration for the optimal plasmid ration revealed, that for large transfer constructs higher amounts of transfer plasmid are required than for smaller constructs to achieve a high productivity (Fig. 1).

**Conclusion**

The outcome of these experiments enabled the development of a robust HEK293T based process to produce clinical relevant LVV under serum-free conditions. Furthermore, it provides an insight how therapeutic genes and the expression of its transgene can influence cell productivity.

**References**

1. Merten OW, et al. Production of lentiviral vectors. Mol Ther Methods Clin Dev. 2016;3:16017. doi: 10.1038/mtm.2016.17

**Fig. 1 (abstract P-279).**
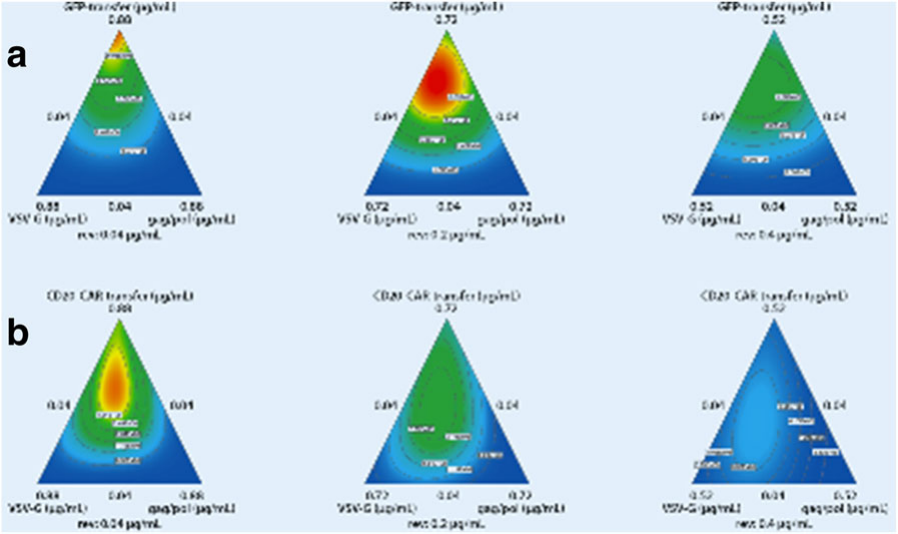
Figure shows response surface graphs for the GFP-transfer plasmid (**a**) and the CD20-CAR-transfer plasmid (**b**) at three different rev-plasmid concentrations (0.04, 0.2, and 0.4 μg/mL). Concentrations of the other plasmids decrease from the respective corner of the graphs towards the corresponding opposite line

## P-281 Molecular impact of the microRNA-23 cluster on bioprocess relevant attributes of Chinese hamster ovary cells

### Kevin Kellner, Nga T. Lao, Orla Coleman, Paula Meleady, Niall Barron

#### National institute for Cellular Biotechnology (NICB), Dublin City University, Dublin, Ireland

##### **Correspondence:** Kevin Kellner (kevin.kellner3@mail.dcu.ie)

**Background**

Chinese Hamster Ovary (CHO) cells are the prominent cell line used in biopharmaceutical production. Although optimisation efforts have led to a vast increase in productivity, CHO cells yield less than other expression systems like yeast or bacteria [1]. To improve yields and find beneficial bioprocess phenotypes, genetic engineering plays an essential role in recent research. The miR-23 cluster with its genomic paralogues (miR-23a and miR-23b) was first identified as differentially expressed during temperature shift, suggesting its role in proliferation and productivity [2]. The common approach to deplete miRNAs is the use of a sponge decoy which, requires the introduction of reporter genes. As an alternative this work aims to knockdown miRNA expression using the recently developed CRISPR/Cas9 system which does not require a reporter transcript. This system consists of two main components: the single guide RNA (sgRNA) and an endonuclease (Cas9) which induces double strand breaks (DSBs). These DSBs can result in insertion or deletion (indels) of base pairs which can disrupt miRNA function and processing [3].

**Materials and methods**

A CHO-K1 cell line stably expressing an IgG was used for knockdown experiments. SgRNAs were designed to target the seed region of each miRNA member and stable mixed populations were generated (Fig. 1a). Total RNA form each mixed population was reverse transcribed into cDNA using miRNA specific stemloop primers. The expression was quantified by RT-qPCR. To further analyse the range of indels the miR-23a and miR-23b clusters were amplified by a standard high-fidelity PCR. Amplicons were cloned into pCR^TM^-TOPO® vector and positive clones were analysed by Sanger sequencing. Cell growth was monitored using ViaCount^TM^ viability stain on a Guava^TM^ benchtop flow cytometer. Productivity was assessed by ELISA. Students t-test was used for statistical analysis.

**Results**

It was shown that miRNA expression was significantly reduced in mixed populations. A knockdown up to 95% was achieved for miR-23a, miR-23b and miR-24. The knockdown in miR-27a and miR-27b expression was considerably less - between 70-90% (n=3, * *P* ≤ 0.05, ** *P* ≤ 0.01, *** *P* ≤ 0.001) (Fig. 1b). Furthermore, it was shown that various sizes of indels were generated by targeting the seed region. Smaller indels (+1/+2/-1/-2 bps) seemed to be more common but larger deletions were detected as well (Fig. 1c). MiR-23a, miR-23b and miR-27b showed increased viability in late stages of the culture. Depletion of miR-27a reduced growth significantly whereas knockdown of miR-24 showed increased proliferation as well as boosting IgG titers (Table 1).

**Conclusion**

In this work, we have shown that CRISPR/Cas9 can be successfully applied as a tool to knockdown miRNA expression in CHO cells. The data was generated using mixed pools and it remains to be established if both alleles can be successfully targeted e.g. using next-generation sequencing of individual clones.

**Acknowledements**

This work is funded by Science Foundation Ireland (SFI: Grant No. 13/IA/1963).

**References**

1. Wurm F M: **Production of recombinant protein therapeutics in cultivated mamammalian cells.** Nature Biotechnology 2014 **22:** 1393-1398.

2. Gammell P, Barron N, Kumar N, Clynes M: **Initial identification of low temperature and culture stage induction of miRNA expression in suspension CHO-K1 cells.** Journal of Biotechnology 2007 **130**: 213-218.

3. Jiang Q, Meng X, Meng L, Chang N, Xiong J, Cao H, Liang Z: **Small indels induced by CRISPR/Cas9 in the 5' region of microRNA lead to its depletion and Drosha processing retardance.** RNA Biology 2014 **11**: 1243-1249.

**Table 1 (abstract P-281).** Phenotypic analysis of mixed populations

Targeted miRNA	Phenotype
MiR-23a	Prolonged culture
MiR-23b	Prolonged culture, decreased productivity
MiR-27a	Decrease in growth
MiR-27b	Prolonged culture
MiR-24	Boost in growth and productivity

**Fig. 1 (abstract P-281).**
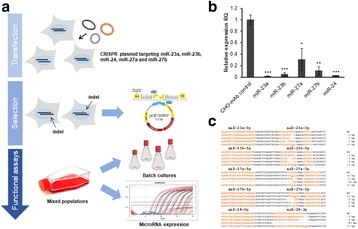
Graphical workflow of targeting miRNAs in CHO cells and analysis of miRNA expression as well as indels

## P-282 Intracellular secretion analysis of recombinant therapeutic antibodies in engineered CHO cells aiming to establish high producer

### Kohei Kaneyoshi^1^, Keiji Uchiyama^2^, Masayoshi Onitsuka^3, 4^, Noriko Yamano^1, 4^, Yuichi Koga^1^, Takeshi Omasa^1,4^

#### ^1^Graduate School of Engineering, Osaka University, Suita, Osaka 5650871, Japan; ^2^The Institute for Enzyme Research, Tokushima University, Tokushima, Tokushima 7708503, Japan; ^3^Graduate School of Technology, Industrial and Social Sciences, Tokushima University, Tokushima, Tokushima 7708513, Japan; ^4^Manufacturing Technology Association of Biologics, Kobe, Hyogo 6500047, Japan

##### **Correspondence:** Takeshi Omasa (omasa@bio.eng.osaka-u.ac.jp)

**Background**

Chinese hamster ovary (CHO) cells are the most widely used host cell line for the production of therapeutic antibodies. Pre- and post-translational modifications and optimization of culture methods contributed to increase the productivity, resulting in a very high titre [1, 2]. However, it has been pointed out that the intracellular secretion process is a bottleneck in the production of therapeutic antibodies [3]. In addition, the details of the process of secretion of humanized recombinant antibodies from CHO cells have not been well investigated. In this study, we thus analysed the detailed process of secretion of therapeutic antibodies using CHO cell lines, which have already been established as high producers, with the aim of obtaining information for the more rational and efficient establishment of high-producer cells.

**Materials and methods**

We performed 1) chase assay, 2) immunofluorescent microscopy observation, and 3) size exclusion chromatography (SEC) analysis to investigate the duration of secretion, bottleneck position, and formation of recombinant IgG, respectively. High-producer CHO cells expressing humanized IgG1 [4] and IgG3 were used. For the chase assay, cells were cultivated in shake flasks with serum-free medium containing 50 μg/ml cycloheximide (CHX) to stop nascent peptide synthesis. The amounts of IgG both remaining in the cell and secreted into the medium at each time point were measured by quantitative western blotting. For immuno-fluorescent microscopy observation, cells were cultivated on coverslips with CHX for 4 h. Immunofluorescent staining against the recombinant IgG, endoplasmic reticulum (ER), and Golgi apparatus was performed after chemical fixation. For SEC, cells cultured with CHX were re-suspended in a buffer containing tritonX-100 and injected into a column. The amount of IgG in each fraction was measured by quantitative western blotting.

**Results and discussion**

The amount of IgG3 in the supernatant increased until 4–6 h after the inhibition of protein synthesis by CHX; however, it hardly changed thereafter (Fig. 1, upper panel). At this point in time, however, around 40% of IgG still remained in the cells (Fig. 1, lower panel), meaning that all of the synthesized IgG could not be secreted into the medium and remained in the cells for several hours. This result was almost the same as that of studies using IgG1-expressing cells [5, 6]. The localization of IgG in the cells was checked before and after the addition of CHX, with the results showing that IgG1 remained in the ER and was hardly seen in the Golgi apparatus [5–7]; IgG did not seem to be efficiently transported to the Golgi apparatus. The SEC experiment showed that most of the IgG1 remaining in the cell seemed to form full-sized antibodies [5, 6], but it could not be secreted despite this.

**Conclusions**

The high-producer cells could not secrete all of the synthesized IgG, and around 40% of IgG remained in the cells for several hours. This incomplete secretion is a common phenomenon among CHO cells producing different types of recombinant IgG. The IgG could not be transported from the ER to the Golgi despite its formation of full-sized antibodies. Solving this bottleneck in the transportation of IgG from the ER to the Golgi and/or achieving more efficient glycosylation of IgG after the formation of full-sized antibodies might be the next target to improve productivity.

**Acknowledgements**

This work was financially supported by the Project Focused on Developing Key Technology of Discovering and Manufacturing Drugs for Next-generation Treatment and Diagnosis from the Ministry of Economy, Trade and Industry of Japan and by Grants-in-aid for Scientific Research from the Japan Society for the Promotion of Science (JSPS; JP26630433, JP26249125 and JP17H06157).

**References**

1. Wurm FM: **Production of recombinant protein therapeutics in cultivated mammalian cells.** *Nat Biotechnol* 2004, **22(11)**:1393-1398

2. Haredy AM, Nishizawa A, Honda K, Ohya T, Ohtake H, Omasa T: **Improved antibody production in Chinese hamster ovary cells by** ***ATF4*** **overexpression.** *Cytotechnology* 2013, **65(6)**:993-1002

3. Peng RW, Guetg C, Tigges M, Fussenegger M: **The vesicle-trafficking protein munc18b increases the secretory capacity of mammalian cells.** *Metab Eng* 2010, **12(1)**:18-25

4. Onitsuka M, Omasa T: **Rapid evaluation of N-glycosylation status of antibodies with chemiluminescent lectin-binding assay.** *J Biosci Bioeng* 2015, **120(1)**:107-110

5. Kaneyoshi K, Uchiyama K, Onitsuka M, Yamano N, Koga Y, Omasa T: **Intracellular secretion pathway analysis for constructing highly producible engineered CHO cells.** *16th annual peptalk*, San Diego, CA, USA, January 2017. Oral and Poster BC1101

6. Kaneyoshi K, Uchiyama K, Onitsuka M, Yamano N, Koga Y, Omasa T: **Intracellular secretion analysis of therapeutic antibodies in engineered high-producible CHO cells**. *Biochemical and Molecular Engineering XX*, Newport beach, CA, USA, July 2017. Poster 41

7. Kaneyoshi K, Uchiyama K, Onitsuka M, Yamano N, Koga Y, Omasa T: **Analysis of intracellular recombinant IgG secretion in engineered CHO cells.** *The 29th annual and international meeting of the Japanese Association for animal cell technology (JAACT2016 Kobe)*, Kobe, Hyogo, Japan, November 2016. Oral O-1

**Fig. 1 (abstract P-282).**
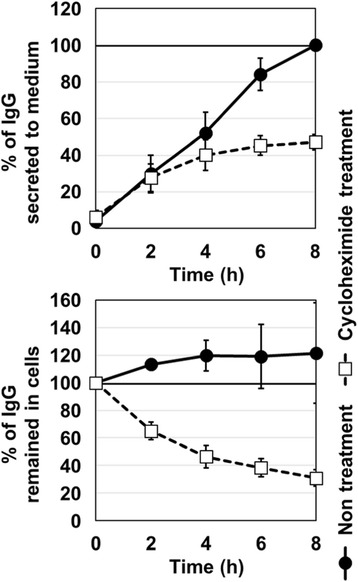
Chase assay of IgG3-producing cells. The amounts of IgG in the medium (upper panel) and the cells (lower panel) were measured every 2 h after the inhibition of protein synthesis by cycloheximide. The amount of IgG in the medium hardly changed between 6 and 8 h; however, 30%–40% of IgG still remained in the cells compared with that at the starting point (0 h)

## P-286 Rational antibody humanization assisted by molecular dynamics simulations

### Linda Schwaigerlehner^1^, Patrick Mayrhofer^1^, Maria Pechlaner^2^, Chris Oostenbrink^2^, Renate Kunert^1^

#### ^1^Department of Biotechnology, University of Natural Resources and Life Sciences, Vienna, Austria; ^2^Department of Material Sciences and Process Engineering, University of Natural Resources and Life Sciences, Vienna, Austria

##### **Correspondence:** Linda Schwaigerlehner (linda.schwaigerlehner@boku.ac.at)

**Background**

Humanized monoclonal antibodies (mAbs) are among the most promising drugs, but defined strategies for their modification are still not available. Our work deals with humanization of murine mAb2/3H6. The superhumanization approach leads to a loss of binding affinity which was partially restored by a single human-to-mouse backmutation (T98hR). [1] This residue was selected by synergistic combination of sequence analyses of antibody framework regions and structural information using novel *in silico* simulations. For structural stabilization, a conglomeration of tyrosine residues surrounding T98hR was identified, the so called “tyrosine cage”. [2] Analysis of the “tyrosine cage” was done by alanine scanning mutations with a double mutation variant T98hR + Y27hA (BM09) and a triple mutation variant T98hR + Y27hA + Y32hA (BM10). In a recent series of experiments we tried to enhance binding affinity by three new variants with backmutations in the variable light chain (VL). Originating from T98hR, residues in the VL were selected based on their spatial proximity to the CDR3 loop of the variable heavy chain. Affinity improvement of T98hR was evaluated by VL-double backmutation variants T98hR + F46lL (SU01) and T98hR + Q49lS (SU02) and a triple backmutation variant T98hR + F46lL + Q49lS (SU03).

**Materials and methods**

All five variants were expressed transiently in HEK293-6E cells and binding affinities were investigated in two individual settings with bio-layer interferometry. In the first approach concentrated cell culture supernatants were directly applied and mAbs were captured on protein A tips, blocked with 3D6scFv-Fc and the association and dissociation of 2F5 IgG was measured. For the second approach, the culture supernatants were purified and the affinity was determined with streptavidin biosensors. First, biotinylated 2F5 IgG was bound and then the association/dissociation of the purified 3H6 variants was measured.

**Results**

Affinity evaluation of concentrated culture supernatants with protein A sensor tips showed a decrease of binding affinity of BM09 and a loss of binding of BM10. The protein A measurement showed an increased binding strength of SU01, SU02 and SU03 compared to su3H6 and BM07. SU01 and SU03 result in a higher binding affinity compared to SU02. These results can be confirmed with purified variants by the streptavidin bio-assay (Fig. 1).

**Conclusions**

Alanine scanning of the tyrosine cage demonstrated a reduction of binding affinity (BM09) and a severe loss of binding (BM10), concluding that the tyrosine cage plays an important role for supporting a correct CDR loop conformation. Further affinity improvement of the single mutation variant T98hR could have been reached via mutations in the VL. It demonstrates the underestimated role of the VL for the interaction with its binding partner.

**Acknowledgements**

Financial support of the Austrian Science Fund (FWF; grant number P 25056) is gratefully acknowledged.

**References**

1. Mader A, Kunert R: **Humanization strategies for an anti-idiotypic antibody mimicking HIV-I gp41.** *Protein Eng. Des. Sel.* 2010, **vol.23**:947–954.

2. Margreitter C, Mayrhofer P, Kunert R, Oostenbrink C: **Antibody humanization by molecular dynamics simulations-*in-silico*** **guided selection of critical backmutations.** *J. Mol. Recognit*. 2016, **vol.29**:266–275.

**Fig. 1 (abstract P-286).**
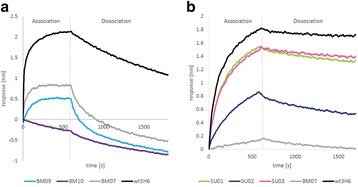
Binding evaluation of mAb2/3H6 variants: Real-time bio-layer interferometry sensorgram aligned to baseline **a** crude culture supernatant detected with protein A biosensors and **b** purified variants measured with streptavidin biosensors

## P-288 MS-SILAC approach for phosphoproteomics of IGF signaling in producer CHO cells

### Louise Brachtvogel^1^, Stefan Walter^2^, Thomas Noll^1^, Raimund Hoffrogge^1^

#### ^1^Institute of Cell Culture Technology, CeBiTec, Bielefeld University, Bielefeld, Germany; ^2^University of Osnabrück, Osnabrück, Germany

##### **Correspondence:** Louise Brachtvogel (l.brachtvogel@uni-bielefeld.de)

**Background**

Although CHO cells are a major expression system for production of recombinant biopharmaceuticals, the molecular and cellular background characterizing a high producer is largely unknown. It has been observed that in producer cell lines important signaling pathways like the Akt-signaling are altered in characteristical ways. Thus analyzing according signaling events should lead to identification of key elements characterizing high producer cells. To investigate this, our emphasis lies on the phosphorylation status of involved proteins as reversible switches in all signaling pathways.

We aimed to establish a workflow for CHO-specific phosphoproteomics and focused on IGF signaling, as cell culture media often are supplemented with this growth factor. Two producer cell lines and the according parental cells were cultivated in a stable isotope labeling with amino acids in cell culture (SILAC) experiment, followed by quantitative MS phosphoproteomic analysis including CHO-specific data evaluation.

**Materials and methods**

The chosen CHO cell lines were cultivated in triplicates in SILAC media containing isotopically-labeled lysine/arginine (hLys/hArg) and in parallel in identical standard media (lLys/lArg, TCX10D, Xell). Cell density, viability, metabolism and cell cycle distribution were monitored during 50 ml batch culture for 7-8 days. At day 3.25 IGF was added into hLys/hArg cultures. 5 min later a part of the cells was harvested. For MS analysis IGF-treated (hLys/hArg cultures) and control cultures (lLys/lArg cultures) were combined. The following MS sample preparation workflow included digestion of whole protein lysate and phosphopeptide enrichment via TIO_2_-beads. NanoLC-ESI-Orbitrap MS (Q Exactive Plus, Thermo Fisher Scientific) of phosphopeptides was excecuted with subsequent identification and quantification in MaxQuant [1]. In addition to SILAC quantification of H/L ratios for investigation of IGF effects, aquired data was also used to perform label-free quantification (LFQ) in MaxQuant [1] for comparison of cell lines. Statistical significance was calculated via t-test (*p*<0.05) or ANOVA (permutation-based FDR<0.05) in Perseus [2].

**Results**

IGF effects on growth and production

The IGF treatment resulted in a prolonged viability for all cell lines. However, an increased VCD was only observed for producer cell line 1, yielding in an enhanced integral of VCD (IVCD). For the parental cells growth was inhibited by IGF, although S-phase cells were enriched at least temporary (Fig. 1a).

Regarding antibody production IGF led to a decreased qP and product titer, concomitantly with an increase in S-phase cells (Fig. 1a). This inverse correlation of proliferation and cell specific productivity is known from different productivity enhancing molecules, like butyrate [3].

MS investigation of signaling events

The phosphoproteomic experiment resulted in the identification of 10.485 class I-phosphorylation sites. Statistical evaluation of phosphopeptide abundances in Perseus showed up 144 significant differences between the cell lines and led to producer vs. parental classifications (Fig. 1b).

The quantitative evaluation via SILAC yielded in about 2.408 quantifiable phosphosites in at least 6 biological replicates. Rapid phosphorylation changes after growth factor treatment indicated signaling towards protein synthesis, cell cycle and regulation of actin cytoskeleton amongst others. For 201 phosphosites significantly different H/L ratios were calculated between the two groups parental vs. producer, four of them are listed (Table 1).

**Conclusions**

The workflow to study phosphorylation states revealed differences in the related cell lines and gave insights into signal transduction as a response on IGF. On the one hand, IGF-treatment resulted in a fast and widespread upregulation of phosphorylation sites within Akt- and MAPK-signaling. On the other hand, a different phosphorylation status for producer compared to parental cell lines uncovered distinctions in biological processes like RNA- and DNA-binding and regulation of cytoskeleton.

In sum, our sucessfully established phosphoproteomic approach allows to detect important signaling key players in CHO cells that subsequently can be targeted through cell engineering or small molecule treatment.

**Acknowledgements**

We would like to thank the Australian Institute for Bioengineering and Nanotechnology, University of Queensland-Brisbane, Australia (AIBN) for providing the CHO clones.

**References**

1. Cox, Mann: **MaxQuant enables high peptide identification rates, individualized p.p.b.-range mass accuracies and proteome-wide protein quantification**. *Nat Biotechnol*. 2008, **26**:1367 – 1372.

2. Tyanova, Temu, Sinitcyn, Carlson, Hein, Geiger, Mann, Cox: **The Perseus computational platform for comprehensive analysis of (prote)omics data**. *Nat Methods*. 2016, **13**: 731–740.

3. Müller, Heinrich, Jabs, Kaspar-Schönefeld, Schmidt, Rodrigues de Carvalho, Albaum, Baessmann, Noll, Hoffrogge: **Label-free protein quantification of sodium butyrate treated CHO cells by ESI-UHR-TOF-MS**. *J Biotechnol.* 2017, **257**:87-98.

**Fig. 1 (abstract P-288).**
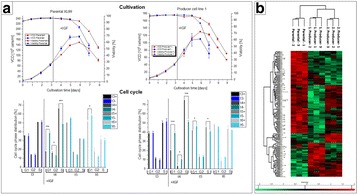
**a** Overview of growth, viability and cell cycle between parental (left) in comparison to one of the producer cell lines (right). **b** Hierarchical clustering of normalized, log-transformed and z-scored intensities as result of the LFQ-evaluation showing significant different phosphopeptide abundances between the cell lines

**Table 1 (abstract P-288).** Selected differentially regulated phosphosites between both producer and parental cells following IGF treatment and SILAC-quantification

Ratio H/L Producer 1+2	Ratio H/L Parental	Phosphosite	Protein Name (CHO)	UniProtAC
1.19	0.74	Ser5	DNA helicase	G3H7V9
1.67	1.01	Ser675 + Ser679	Lethal (2) giant larvae protein-like 1	G3GVH7
1.50	1.00	Ser756	Uncharacterized protein (Prune homology 2, human)	G3I7N4

## P-293 Construction of a system for rapid evaluation of production enhancer gene in CHO antibody production

### Masayoshi Onitsuka^1,2^, Yuuki Fujino^3^, Namiko Kawamura^3^,Takeshi Omasa^1,2,4^

#### ^1^Graduate School of Technology, Industrial and Social Sciences, Tokushima University, Tokushima, 770-8506, Japan; ^2^Manufacturing Technology Association of Biologics (MAB), Kobe, 650-0047, Japan; ^3^Graduate School of Advanced Technology and Science, Tokushima University, Tokushima, 770-8506, Japan; ^4^Graduate School of Engineering, Osaka University, Suita, 565-0871, Japan

##### **Correspondence:** Masayoshi Onitsuka (onitsuka@tokushima-u.ac.jp)

**Background**

To improve antibody production in the CHO cell expression system, it seems to be useful to up- or downregulate gene expression including antibody folding, secretion, and cell metabolism. Many cell engineering approaches, including gene introduction, knockout and knockdown, have been employed to enhance recombinant antibody production [1]. However, identifying production enhancer genes is the rate-limiting step for CHO cell engineering, because the conventional method requires a series of experiments including genomic integration of the tested genes, selection of stable cell clones and cell culture experiments of several clones. In this study, we propose an approach for rapid evaluation of production enhancer genes based on an episomal expression system.

**Materials and methods**

Plasmid vector carrying the Epstein–Barr virus (EBV) encoded nuclear antigen 1 (EBNA1) was transfected into CHO cell line producing IgG1 antibody. After G418 selection and single colony isolation, EBNA1 expression was checked with capillary electrophoresis system Wes (ProteinSimple). EBV EBNA1-antibody (1EB12) was used for detection as the primary antibody. The expression vector for the gene of interest was prepared by inserting 1508 bp of an oriP DNA sequence into a plasmid vector carrying CAG promoter, resulting in the pOTC vector. PEI max (Polysciences, Inc.) and BalanCD Transfectory CHO (Irvine Scientific) were used for the transfection. The number of viable cells and GFP-positive cells were counted using Countess II FL Automated Cell Counter (Thermo Fisher Scientific). The transfected cells were cultured in CELLSTAR CELLreactor tubes. The tubes were incubated in a Climo-shaker ISF1-X (Kuhner). Antibody production was measured using biolayer interferometry with an Octet QK system (Fortebio).

**Results**

We constructed four CHO cell lines stably expressing EBNA1, termed IgG1-EB01 to EB04. In capillary electrophoresis analysis, we observed a clear peak corresponding to the EBNA1 expression in all four cell lines. We tested the transfection efficiency by pOTC-GFP plasmids. In the best transfection condition, PEI/DNA ratio of 1/1, IgG1-EB01 cell showed the highest GFP-positive cell number (1.07×10^7^ cell/mL) and transfection efficiency (95%) among the four cell lines. Therefore, IgG1-EB01 cell lines were selected for further study. After the transfection, the number of GFP-positive cells continued to increase even after the passage (Fig. 1), suggesting that the pOTC-GFP plasmid was stably retained and replicated by EBNA1/oriP system in IgG1-EB01 cell lines. In preliminary experiments, we introduced three genes, MDH2, GSS and GCLM, into IgG1-EB01 cell lines. Cotransfection of these three genes led to an increase in IgG1 production from 287±18 mg/L (control) to 334±21 mg/L at day 8 (P<0.05, t-test, n=3). This result suggests that these three genes work as production enhancer genes. Conventional methods based on stable cells take up to 6 months to determine whether the gene of interest is beneficial for recombinant IgG1 production. In contrast, identification of production enhancer genes is achievable within 10 days by our proposed method based on EBNA1/oriP system.

**Conclusions**

The proposed method makes it possible to evaluate production enhancer genes in a rapid manner. The proposed method is a promising approach to identify genes enhancing recombinant antibody production.

**Acknowledgements**

This research is partially supported by the developing key technologies for discovering and manufacturing pharmaceuticals used for next-generation treatments and diagnoses both from METI and AMED, Japan.

**References**

1. Fischer S, Handrick R, Otte K: The art of CHO cell engineering: a comprehensive retrospect and future perspectives. Biotechnol Adv 2015, 33:1878-1896.

**Fig. 1 (abstract P-293).**
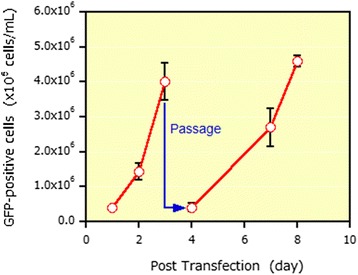
Number of GFP-positive cells in IgG1-EB01 cell lines after transfection

## P-294 GS system for increased expression of difficult-to-express proteins

### Bart Engels^1^, Annemarie de Jel^1^, Chantal Tilburgs^1^, Elise Krabbendam^1^, Maurice van der Heijden^1^

#### ProteoNic, Leiden, The Netherlands

##### **Correspondence:** Maurice van der Heijden (heijden@proteonic.nl)

**Background**

2G UNic™ (2GUN) technology comprises a set of protected genetic elements that improve protein production by acting on transcription as well as on translation. The elements can either be inserted into existing (platform) vectors or be provided as complete ready-to-use vectors. The technology can be used in stable and in transient transfection to boost protein production for product development and is being applied in CLD for pharmaceutical proteins. In combination with antibiotic selection or DHFR selection, 2GUN technology routinely results in 2-3 fold increase in expression of client antibodies or fusion proteins, both in pools and after clonal selection.

Previously, we have successfully combined 2GUN technology with glutamine synthetase (GS) selection and the CHO GS null cells of Horizon Discovery, resulting in clonal cell lines producing > 6 g/L of a biosimilar mAb in fed-batch assay.

Here we present data on the successful application of the 2GUN technology for the enhanced expression of a large (>300 kDa) human heterotrimeric glycoprotein, a renowned difficult-to-express (DTE) protein.

**Materials and methods**

All expression vectors comprised a hCMV promoter and BGH poly-adenylation sequence in the expression cassettes for the gene of interest, and a selection marker gene with SV40 promoter and SV40 polyadenylation sequence. 2G UNic™ Vectors also contained genetic elements (2G UNic™ technology, ProteoNic). CHO GS null cells (Horizon Discovery) were transfected in duplicate with Reference or 2GUN expression vectors and selected in media lacking glutamine and containing the appropriate antibiotics. The bulk pools were seeded at equal viable cell density after obtaining maximum viability and cultured for 9 days without feeding (batch). Expression of the target protein in cell culture supernatants of stable bulk pools was measured by ELISA.

**Results**

The three protein subunit genes were expressed from vectors with different selection markers. In the Reference constructs (without 2GUN), the α, β, and γ chains were expressed from vectors with marker genes for zeocin, blasticidin, and GS, respectively. A similar 3 vector combination was also generated with 2GUN elements integrated in each vector. In addition, 2 vectors with 2 subunits (γ-α and α-γ), each with a separate 2GUN element, promoter and poly-adenylation signal, were generated with a GS marker gene. CHO GS^-/-^ cells were transfected with the 4 appropriate vector combinations in equimolar ratios and selected in bulk in medium lacking glutamine and 1 or 2 antibiotics. The 2-vector transfected cell pools recovered first, due to the presence of only 1 antibiotic in the medium (Fig. 1a). The pools transfected with three 2GUN vectors recovered to maximum viability just a few days after the 2-vector 2GUN pools. Recovery of the Reference pools took up to a week longer than the 2GUN pools. Production of each pool was assessed in a batch production run in shaker flasks. All 2-vector 2GUN pools which recovered first produced titers around 0.1 g/L, which is almost 10-fold higher as compared to the production by Reference pools (Fig. 1b). The highest titers of 0.5 g/L were obtained in the 3-vector 2GUN pools. These data show that the 2G UNic™ genetic elements can be successfully used to obtain a significant increase in the titer of difficult-to-express proteins. Similar results have been obtained with other DTE proteins, including fc-fusion proteins and bi-specific antibodies (not shown).

**Conclusions**

The expression of a large, glycosylated multimeric difficult to express protein can be increased more than ten-fold in CHO GS pools by application of 2G UNic™ genetic elements. The highest expression of is obtained using a separate vector for each subunit.

**Fig. 1 (abstract P-294).**
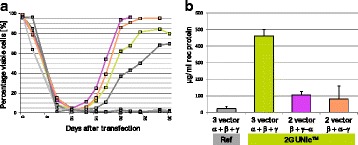
CHO GS^-/-^ cells were transfected with 4 different sets of expression plasmids: Reference (without 2GUN; grey), 3-vector 2GUN (green), 2-vector 2GUN with γ-α (pink), and 2-vector 2GUN with α-γ (orange). Panel **a**: recovery of bulk pools on selective medium. Each line is the average of 4 bulk pools: 2 replicate transfections, each split into two flasks after transfection. Mock and GFP transfected pools didn’t recover (light grey lines). Panel **b**: After recovery, each pool was seeded in fresh medium in shaker flasks and cultured for 9 days. The bars show the average expression of 4 bulk pools

## P-302 Characterization of antibody-producing CHO cells with chromosome aneuploidy

### Noriko Yamano^1,2^, Sho Tanaka^1^, Norichika Ogata^2, 3^, Masayoshi Onitsuka^2, 4^, Yuichi Koga^1^, Takeshi Omasa^1, 2^

#### ^1^Graduate School of Engineering, Osaka University, 2-1 Yamadaoka, Suita, Osaka 565-0871, Japan; ^2^Manufacturing Technology Association of Biologics, 7-1-49 Minatojima-Minamimachi, Chuo-ku, Kobe, Hyogo 650-0047, Japan; ^3^Nihon BioData Corporation, 3-2-1 Sakado, Takatsu-ku, Kawasaki, Kanagawa 213-0012, Japan; ^4^Graduate School of Technology, Industrial and Social Sciences, Tokushima University, 2-1 Minamijosanjima-cho, Tokushima, Tokushima 770-8513, Japan

##### **Correspondence:** Noriko Yamano (yamanori@bio.mls.eng.osaka-u.ac.jp)

**Background**

Chinese hamster ovary (CHO) cells are commonly used as host cells to produce biopharmaceuticals. However, the number of chromosomes in CHO cells varies. Previously, DG44-SC20 and DG44-SC39 cell lines with modal chromosome numbers of 20 and 39 were isolated from parental CHO-DG44 cells, from which IgG3-expressing cell lines named IgG3-SC20 and IgG3-SC39 were established, respectively. The IgG3-SC39 cell pool showed a higher specific IgG3 production rate than the IgG3-SC20 cell pool [1]. Even though all of the IgG3-SC20 clones and half of the IgG3-SC39 clones contained the same number of vector integration sites (single integration site), IgG-SC39 cell clones produced more IgG3 following the culture of single-cell clones than any of the IgG3-SC20 clones [1]. In this study, we performed transcriptome analysis to investigate the characteristics of high-producer cells with chromosome aneuploidy.

**Materials and methods**

Transcriptome analyses using amplified fragment length polymorphism (AFLP)-based high-coverage expression profiling (HiCEP) and *de novo* mRNA-seq were performed on DG44-SC20, DG44-SC39, IgG3-SC20 and IgG3-SC39. To compare cell lines with different numbers of chromosomes, transcriptome data from mRNA-seq were adjusted for cell number using RNA reference materials (NMIJ CRM 6204-a; National Institute of Advanced Industrial Science and Technology) mixed at equal amounts per cell. Pathways related to differentially expressed genes were searched using KeyMolnet (KM Data).

**Results and discussion**

High-chromosome-number CHO cells showed larger cell diameters, as determined by Vi-cell (Beckman Coulter) measurement. The predicted volume ratios, based on these diameters, are 2.24 (DG44-SC39:DG44-SC20) and 1.59 (IgG3-SC39:IgG3-SC20). The levels of β-actin and the products of most other genes that were detected by mRNA-seq differed by approximately 20% in the comparison between SC39 and SC20 (SC39 > SC20). Based on the analysis of gene expression levels per cell volume, approximately 90% of detected genes showed lower expression in both DG44-SC39 and IgG3-SC39 compared with the levels in DG44-SC20 and IgG3-SC20, respectively. In addition, the number of genes whose expression level was decreased in IgG3-SC39 compared with that in DG44-SC39 was larger than those showing the opposite pattern. The results of the comparisons between IgG3-SC20 and IgG3-SC39 indicate that differentially expressed genes were mainly related to cell growth (e.g. Myc, SMAD), apoptosis (e.g. Caspase), lipid metabolism (e.g. SREBP, PPARγ) and epigenetic histone modification (e.g. BRCA, HAT) pathways. The mRNA levels of Myc, SMAD, Caspase, BRCA and HAT related genes were lower in IgG3-SC39, while those of SREBP and PPARγ related genes were higher in IgG3-SC39. The effects of these pathways on antibody production should be examined in future.

**Conclusions**

In this study, we found that high-chromosome-number CHO cells have lower amounts of mRNA relative to their volume. A reduction per unit volume in the expression of genes that are required for survival might generate additional energy for recombinant protein production in high-chromosome-number cells. From an evolutionary perspective, an increased set of chromosomes underlies rapid evolutionary adaptation. Although there are issues to be considered, such as stability, there may also be advantages to using high-chromosome-number aneuploid CHO cells as a production host cells of recombinant proteins.

**Acknowledgements**

This work was funded partly by the Ministry of Economy, Trade and Industry of Japan (METI) and the Japan Agency for Medical Research and Development (AMED) for the Project Focused on Developing Key Technology of Discovering and Manufacturing Drugs for Next-generation Treatment and Diagnosis and partly by KAKENHI grants from the Japan Society for the Promotion of Science (JSPS; JP26630433, JP26249125 and JP17H06157).

**References**

1. Yamano N., Takahashi M., Haghparast S. M. A., Onitsuka M.*,* Kumamoto T. Frank J. Omasa T.: **Increased recombinant protein production owing to expanded opportunities for vector integration in high chromosome number Chinese hamster ovary cells.** *Journal of Bioscience and Bioengineering* 2016, **122**: 226-231.

## P-317 Investigation of factors influencing recombinant human BMP2 expression in mammalian cells

### Valérie Jérôme^1^, Lena Thoring^2^, Alexander Raup^1^, Denise Salzig^1, 3^, Sören Blum^1^, Florian Gruber^1^, Jennifer Nack^1^, Stefan Kubick^2^, Ruth Freitag^1^

#### ^1^Chair for Process Biotechnology, University of Bayreuth, Bayreuth, 95447, Germany; ^2^Department of Cell-free and Cell-based Bioproduction, Fraunhofer Institute for Cell Therapy and Immunology (IZI), Branch Bioanalytics and Bioprocesses Potsdam-Golm (IZI-BB), Leipzig, Germany; ^3^Present address: University of Applied Sciences Mittelhessen, Institute of Bioprocess Engineering and Pharmaceutical Technology, Wiesenstrasse 14, 35390 Giessen, Germany

##### **Correspondence:** Ruth Freitag (ruth.freitag@uni-bayreuth.de)

**Background**

Human growth factors have an enormous therapeutic potential. Among them, the bone morphogenetic protein-2 (BMP-2) can induce *de novo* bone formation endowing the protein a high therapeutic potential. However, finding a suitable recombinant production system for such a protein still remains a challenge.

**Materials and methods**

Recombinant expression of hBMP2 was investigated in transiently transfected HEK-293 cells and in stable clones established in CHO-K1 cells cultivated in ExCell and Pro-CHO5 medium, respectively. Protein stability and interaction of the hBMP2 with the producer cells were investigated *in vitro* using commercially available rhBMP2. In addition, we investigated a cell-free protein synthesis system harboring translocationally active microsomal structures, hence having the potential to perform post-translational modifications, as an alternative production method.

**Results**

We showed that growth rates and viabilities of the rhBMP2-producing cells were similar to those of the parent cell line, while entry into the death phase was delayed in case of the recombinant cells. The maximum rhBMP2 concentration detected in the culture supernatant was low for stable clones but can be greatly improved combining the HEK-293 cells transient expression system and batch reactor cultivation which reflects a better compatibility of the codon usage in the human cells (Table 1).

hBMP2 protein is sensitive to slightly acidic pH and to a lesser extend to proteases (Fig. 1a) and binds to both producers cell lines (Fig. 1b) – All this could incidentally contribute to the low product titers.

Cell-free protein synthesis has been proposed as alternative for “difficult-to-express” proteins. Since native hBMP2 is glycosylated, a cell-free system based on eukaryotic cell lysates is required for its production. CHO cell lysates were chosen, since they had previously been established as the most productive eukaryotic system in our hands [1], while concomitantly enabling a direct comparison to the production of hBMP2 in stable clones established in CHO-K1. The ability to perform post-translational modifications is a major advantage of eukaryotic systems. The CHO lysates prepared by the protocol used here have previously been shown to contain significant amounts of endogenous microsomes derived from the endoplasmatic reticulum during lysis [2]. To enforce translocation of the target protein into the microsomal structures, a melittin signal peptide was fused to the hBMP2 cDNA. The glycosylation of the protein was assessed by enzymatic treatment (PNGase, EndoH) and confirmed using ^14^C-mannose for the *de novo* protein synthesis. Upon cell-free protein synthesis, the hBMP2 yield was 100-fold higher than the best one in the HEK-293 cells. The difference becomes even more dramatic, when productivities are considered (Table 1), i.e. the fact that maximum product titers are reached within 3 h in the cell-free system compared to 120 h in the cell-based ones. This demonstrates that the cell-free expression system is most suitable compared to mammalian cell expression method for the production of glycosylated human BMP2 (Table 1) [3].

**Conclusion**

Human growth factors are complex molecules, which make their production in mammalian cells desirable. However, low product titers caused by a variety of both cell and process related effects may hinder the development of highly productive processes. In such cases, cell-free protein production using CHO cell lysates containing endogenous microsomes for post-translational processing, may eventually present an attractive alternative. In particular since these lysates can be used under tightly controlled conditions assuring a higher degree of reproducibility, than, e.g. transient transfection systems. Cell-free systems are known to circumvent typical bottlenecks of cell-based ones, e.g. metabolic regulation and cell maintenance mechanisms. In consequence, the production of a recombinant protein is neither inhibited by its accumulation nor by any interaction with the cells, e.g. through the activation of inhibitory signaling pathways.

**References**

1. Brödel, A. K.; Sonnabend, A.; Roberts, L. O.; Stech, M.; Wüstenhagen, D. A.; Kubick, S., **IRES-mediated translation of membrane proteins and glycoproteins in eukaryotic cell-free systems**. PLoS ONE 2013, 8, (12), e82234.

2. Brödel, A. K.; Sonnabend, A.; Kubick, S., **Cell-free protein expression based on extracts from CHO cells**. Biotechnol Bioeng 2014, 111, (1), 25–36.

3. Jérôme, V.; Thoring, L.; Salzig, D.; Kubick, S.; Freitag, R.: **Comparison of cell-based vs. cell-free mammalian systems for the production of a recombinant human bone morphogenic growth factor.** *Eng. Life Sci.* 2017, DOI: 10.1002/elsc.201700005.

**Table 1 (abstract P-317).** Comparative analysis of the three production systems in term of yield and specific productivity

Production system	Total amount produced [μg]	Time to peak hBMP2 titer [h]	Peak hBMP2 titer [ng/mL]	Specific productivity [ng/(mL h)]
Recombinant cell line (CHO)	0.015	120	0.15	0.0013
Transient production (HEK-293)	25	120	274.9	2.3
Cell-free system (CHO)	0.75	3	37000	12333.3

**Fig. 1 (abstract P-317).**
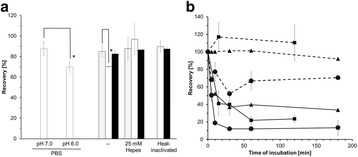
*In vitro* stability and interaction of rhBMP2 with the producer cells. **a** Stability of recombinant hBMP2: rhBMP2 produced in *E.coli* (white bars) and in CHO cells (black bars), duration: 120h. Fresh (dotted bars) and end-of-culture (solid bars) ExCell medium. **b** Binding of hBMP2 to CHO and HEK-293 cells: rhBMP2 produced in *E.coli* (■,▲) and in CHO cells (●). ■ : 3 x 10^6^ CHO cells mL^-1^ in ProCHO5 medium (solid line). ▲, ● : 4 x 10^6^ HEK-293 cells mL^-1^ in ExCell medium (solid lines). rhBMP2 incubated in the respective medium without cells (dashed lines). rhBMP2: 5 ng mL^-1^, incubation temperature 37°C

## P-318 Systematic investigation of PDMAEMA-functionalized magnetic nanoparticles for their utilization in biotechnology

### Ullrich Stahlschmidt^1^, Alexander P. Majewski^2^, Valérie Jérôme^1^, Axel H.E. Müller^3^, Ruth Freitag^1^

#### ^1^Chair for Process Biotechnology, University of Bayreuth, Bayreuth, 95447, Germany; ^2^Evonik Resource Efficiency GmbH, Marl, Germany, 45772; ^3^Institute for Organic Chemistry, Johannes-Gutenberg-University of Mainz, Mainz, 55128, Germany

##### **Correspondence:** Ruth Freitag (ruth.freitag@uni-bayreuth.de)

**Background**

In the recent years, the development of novel non-viral vectors for gene delivery into mammalian cells has increasingly become on focus. Recently, we reported on novel PDMAEMA-based nano-stars based on a superparamagnetic maghemite nanoparticles (γ-Fe_2_O_3_) core. Preliminary studies showed that the corresponding polyplexes, but also some of the cells that came into contact with them, became magnetic and were manageable by magnetic fields [1-3]. Here, we present a characterization of the influence of structure and composition on the function of these polymers using a library of highly homogeneous, paramagnetic nano-stars with varied arm lengths and densities [4].

**Materials and methods**

The paramagnetic nano-stars library was synthesized by coating maghemite nanoparticles (γ-Fe_2_O_3_) with a thin silica-shell functionalized with an atomic transfer radical polymerization (ATRP) initiator. PDMAEMA arms were grown from the core particles via ATRP. In one case, the PDMAEMA arms was end-capped with PDEGMA blocks produced during a second ATRP step. All nano-stars were characterized by size exclusion chromatography and thermogravimetry to calculate number and length of the PDMAEMA arms. The core diameter was determined by transmission electron microscopy and dynamic light scattering (DLS). The different variants (Table 1) were analyzed for their ability to complex pDNA (pEGFP-N1) using various physicochemical methods (DLS, Zeta sizer). Transfection efficiency/cytotoxicity in CHO-K1 cells were determined by flow cytometry. Transfected cells were placed in a magnetic field and the influence of the polymer architecture on the magnetic separation was investigated. Non-parametric Spearman analysis was used to correlate between arm length/arm densities, magnetic properties of the cells and transfection efficiency.

**Results**

Based on the hydrodynamic radii of the polyplexes, the investigated nano-stars could be divided into three subgroups (Table 1). Middle, but also high arm density nano-stars formed smaller polyplexes with hydrodynamic radii ≤ 300 nm, a size that is considered suitable for endocytosis and transfection.

Transfection efficiencies and cytotoxicities varied systematically with the nano-stars architecture, with viability showing a more pronounced dependency on the characteristics of the transfection agent than the transfection efficiency itself. The arm density was particularly important, with values of approximately 0.06 arms/nm^2^ yielding the best results (Fig. 1a). The end-capping the polycation arms with PDEGMA significantly improved the serum compatibility (Fig. 1b).

The gene delivery potential of a given nano-star and its ability to render the cells magnetic did not correlate. Although, compared to the non-separated cells, EGFP-expressing cells were consistently more frequent in the magnetic cell fraction, while the non-magnetic fraction was slightly depleted. When the EGFP-expressing cells were further divided into low, middle and high producers, a statistically significant shift towards the high producers was observed in the magnetic cell fraction (Fig. 1c).

A nonparametric Spearman correlation analysis was used to statistically evaluate possible links between the molecular characteristics of the nano-stars, the physicochemical properties of the corresponding polyplexes, the transfection conditions, and the cellular reactions. The resulting correlogram is shown in Fig. 1d.

**Conclusions**

Transfection agents with magnetic properties enlarge the toolbox for studying non-viral gene delivery, since cellular magnetism is added as a new parameter. This allows, *inter alia*, a distinction between mere cellular interaction and actual uptake, which is otherwise difficult. Viability showed a much more pronounced dependency on the characteristics of the transfection agent/polyplex than the transfection efficiency itself, which should be taken into account during method optimization. End-capping the polycationic PDMAEMA-arms with PDEGMA-blocks improved the compatibility of the polycationic nano-stars with serum components. In future optimized, blood-compatible, nano-stars, which can be retained/directed by magnetic fields, could become options for non-viral gene delivery *in vivo*.

**Acknowledgements**

This research was supported by the Upper Franconian Trust, grant P-Nr.: 03847.

**References**

1. Majewski, A. P.; Schallon, A.; Jérôme, V.; Freitag, R.; Müller, A. H.; Schmalz, H., **Dual-responsive magnetic core-shell nanoparticles for nonviral gene delivery and cell separation.** *Biomacromolecules* 2012, **13**:857-866.

2. Majewski, A. P.; Stahlschmidt, U.; Jérôme, V.; Freitag, R.; Müller, A. H. E.; Schmalz, H.: **PDMAEMA-grafted core-shell-corona particles for nonviral gene delivery and magnetic cell separation.** *Biomacromolecules* 2013, **14**: 3081-3090.

3. Raup, A.; Stahlschmidt, U.; Jérôme, V.; Synatschke, C. V.; Müller, A. H. E.; Freitag, R., **Influence of polyplex formation on the performance of star-shaped polycationic transfection agents for mammalian cells**. *Polymers* 2016, **8**: 224-240.

4. Stahlschmidt, U.; Jérôme, V.; Majewski, A.; Müller, A.; Freitag, R.: **Systematic Study of a Library of PDMAEMA-Based, Superparamagnetic Nano-Stars for the Transfection of CHO-K1 Cells.** *Polymers* 2017, **9**: 156-175.

**Table 1 (abstract P-318).** Molecular characteristics of the nano-stars used in this study (sorted in ascending order of arm grafting density)

Designation	Name^a^	Grafting density (arms/nm^2^)	Mn Polymer/Particle (kg/mol)	PDI	Subgroup^b^
P300_5	NP@(PDMAEMA_300_)_5_	0.006	235	1.2	I
P242_9	NP@(PDMAEMA_242_)_9_	0.011	342	1.2	I
P528_20	NP@(PDMAEMA_528_)_20_	0.024	1660	1.3	I
P1100-4350_98	NP@(PDMAEMA_1100_-b-PDEGMA_4350_)_98_	0.038	975000	1.6	n.a.
P1037_46	NP@(PDMAEMA_1037_)_46_	0.054	7498	1.6	II
P1470_54	NP@(PDMAEMA_1470_)_54_	0.064	12470	1.4	II
P312_337	NP@(PDMAEMA_312_)_337_	0.149	16513	1.4	III
P477_411	NP@(PDMAEMA_477_)_411_	0.182	30825	1.5	III
P1240_653	NP@(PDMAEMA_1240_)_653_	0.289	127335	1.5	III
P439_657	NP@(PDMAEMA_439_)_657_	0.291	45333	1.4	III
P1616_679	NP@(PDMAEMA_1616_)_679_	0.300	172466	1.3	III

PDMAEMA: poly(dimethylaminoethyl methacrylate). PDEGMA: poly(diethylene glycol) methyl ether methacrylate). PDI: polydispersity index (Mw/Mn). a: NP@(PDMAEMA_Block length_)_Arm number_. b: The classification of the variants in the subgroups is based on the arm density and on the hydrodynamic radii (R_h_) of the corresponding polyplexes covering a wide range of N/P-ratios and analyzed for size by DLS after a 30 min incubation (I: low arm density, 300 < R_h_ (nm) ≤ 1000; II: middle arm density, 50 < R_h_ (nm) ≤ 200; III: high arm density, 50 < R_h_ (nm) ≤ 300). n.a.: not available

**Fig. 1 (abstract P-318).**
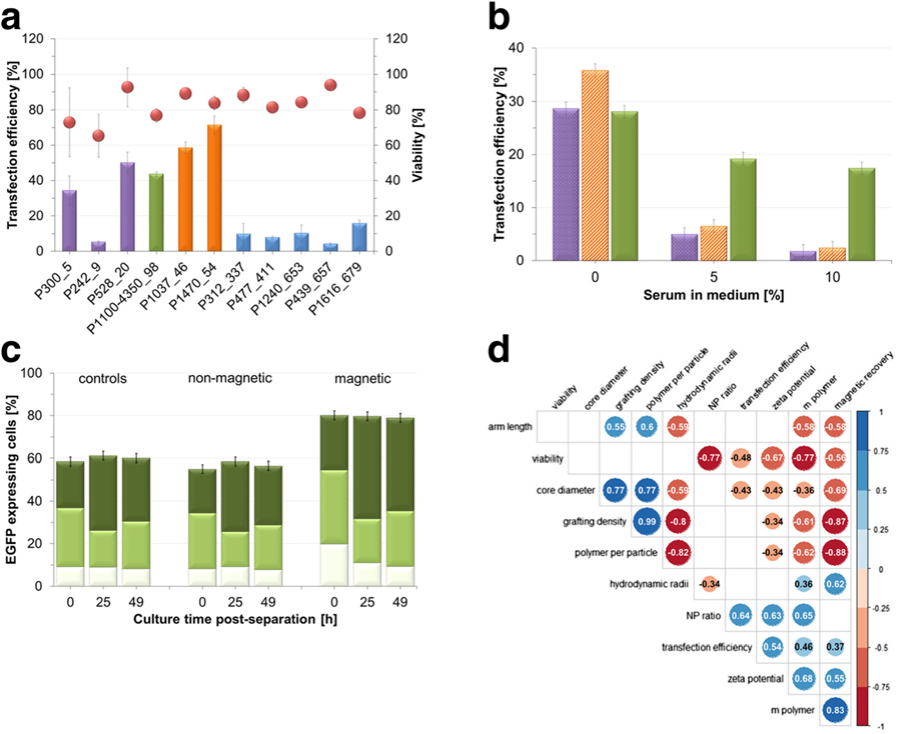
Transfection efficiency and magnetic property of the cells after transfection. **a** Transfection in serum-free medium at optimal N/P ratios for each polycation. Transfection efficiencies (EGFP^+^-cells, bars) and viabilities (circle) determined 26h post transfection. Subgroups according to the grafting density/hydrodynamic radii of the corresponding polyplexes: I (violet), II (orange), III (blue). Data represent mean ± S.E.M., n ≥ 3. **b** Effect of serum on transfection efficiencies of NP@(PDMAEMA_528_)_20_ (N/P 20) (violet, dotted bars), NP@(PDMAEMA_1037_)_46_ (N/P 15) (orange, stripped bars), and NP@(PDMAEMA_1100_-b-PDEGMA_4350_)_98_ (N/P 20) (green, solid bars). Data represent one experiment carried out in duplicate, with random experimental error shown. **c** EGFP expression in magnetic and non-magnetic cell fractions after magnetic separation compared to the controls (non-separated cells). Cells were transfected with NP@(PDMAEMA_1037_)_46_ (N/P 15), separated 24 h post transfection (t = 0) by magnetically-assisted cell sorting and placed into separated cultures. The bars represent the overall transfection efficiency. Distribution of low (light green), middle (green), and high (dark green) producers within the EGFP-expressing cell fraction. Data represent one experiment carried out in duplicate, with random experimental error shown. **d** Correlogram between the molecular characteristics of the nano-stars (core diameter, arm density, arm length, number of monomeric units per nano-star), the physicochemical properties of the corresponding polyplexes (hydrodynamic radius, zeta potential), the transfection conditions (N/P ratio, amount of polymer), and the cellular reactions (transfection efficiency, magnetism, viability). Positive correlations are indicated in blue, negative ones in red. Correlation coefficients were not calculated for parameters, which showed no statistically relevant differences (p > 0.01)

## P-323 UPR-mediated increase in IgG productivity in rCHO cells adapted under mild tunicamycin stress

### Vikas Chandrawanshi, Sarika Mehra

#### Chemical Engineering, Indian Institute of technology Bombay, Mumbai, India

##### **Correspondence:** Sarika Mehra (sarika@che.iitb.ac.in)

**Background**

The increasing demand for monoclonal antibodies has necessitated the need to increase the productivity of current industrial cell lines. In our earlier study [1], we had shown that treatment with ER-stress inducer, Tunicamycin significantly increased the titers and productivity in recombinant CHO cell lines with a simultaneous upregulation of many genes from the unfolded protein response pathway (UPR). However the loss in cell viability prevented a sustained increase in titers. In the current study we explore the effect of varying concentrations of tunicamycin and treatment times, such as to modulate the increase in protein folding capacity while preventing induction of apoptosis.

**Materials and methods**

Anti-rhesus IgG-secreting CHO cells [2] were cultured in SF-CDM in 125 mL shake flasks. The cells were treated with varying concentrations (30-500 ng/mL) of tunicamycin in a batch culture. Further, the effect of treatment with tunicamycin for short periods of time (24 hrs) was also evaluated. IgG titers and mRNA expression levels were quantified using ELISA and qRT-PCR (Illumina), respectively.

**Results**

CHO cells were treated with different concentrations of tunicamycin and cultured in a batch for 8 days (referred as Continuous treatment/CTE). Figure 1a presents the maximum VCD and % drop in viability under treatment. A dose-dependent inhibitory effect is observed on growth and viability of cells in CTE-cultures, with minimal inhibition as lower concentrations. Contrastingly, IgG titers (Fig. 1b) were higher in treated cultures w.r.t. control in initial phase of the cultures at all the concentrations of tunicamycin. The per-cell productivity (Fig. 1c) also showed a significant increase w.r.t control at all the concentrations of tunicamycin. However, the increased productivity due to tunicamycin was not sustained and levels become similar to control after day 3 (data not shown).

To prevent loss of viability due to tunicamycin, the effect of short-term treatment (STE) with tunicamycin was explored. Cells treated with tunicamycin for 24 hours were harvested (corresponding to day 2 of CTE cultures) and inoculated in fresh media. The STE-cultures showed improved viability and higher maximum VCD as compared to CTE-cultures (Fig. 1a). The fold increase in IgG titers was not sustained beyond day 1-2 in STE-cultures (Fig. 1d) but significant increase in productivity was seen in the initial phase (Fig. 1e). Further, the cells were adapted over 25 continuous generations under 30ng/mL tunicamycin. The adapted cells had overall 1.3-fold higher productivity, as compared to control (Fig. 1f), in a batch culture.

To understand the molecular basis of increase in productivity, mRNA expression level of key genes was determined. XBP1s is a transcription factor involved in activation of chaperones (like Grp78, Calreticulin) and apoptotic genes (such as CHOP). Significant increase in the levels of calreticulin was seen on treatment with tunicamycin (Fig. 1g). Both XBP1s and Grp78 were marginally induced when treated with 30ng/mL of tunicamycin in both CTE-and STE-cultures (Fig. 1h), and significantly up-regulated when treated with 500ng/mL of tunicamycin. The CHOP mRNA levels also increase with increasing tunicamycin concentrations, with levels in STE-cultures lower than CTE-cultures (Fig. 1h). The results suggest that UPR induction may be important to increase productivity in these CTE/STE-cultures. Note that, tunicamycin had no effect on the expression levels of IgG heavy-chain, thus eliminating the involvement of IgGHC-mRNA in increasing productivity (Fig. 1i).

**Conclusions**

Tunicamycin induced ER-stress increased productivity in the initial phase of the culture and enhanced UPR-mediated folding capacity can be attributed as one of the reasons for it. At lower concentrations of tunicamycin, a fine balance between optimum UPR induction and apoptosis can be achieved, as seen in 30ng/mL tunicamycin STE-cultures. In summary, this study demonstrates an alternate approach to enhance productivity of current industrial cell lines.

**Acknowledgements**

This work was partially funded by DBT, Govt. of India. VC acknowledges UGC for his fellowship.

**References**

1. Segar, K.P., V. Chandrawanshi, and S. Mehra, Systems biology of unfolded protein response in recombinant CHO cells. BMC Proceedings, 2013. 7(Suppl 6): p. P67-P67.

2. Prashad, K. and S. Mehra, Dynamics of unfolded protein response in recombinant CHO cells. Cytotechnology, 2015. 67(2): p. 237-254.

**Fig. 1 (abstract P-323).**
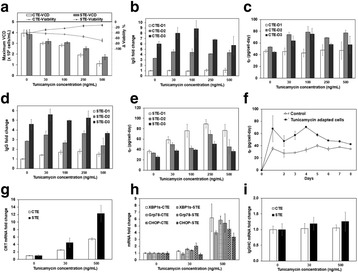
**a** Growth characteristics of CTE-and STE-cultures: comparison of maximum viable cell density achieved when rCHO cells are treated with varying concentrations of tunicamycin. Note, the maximum VCD was obtained on day 6. The % drop in viability at day 6 from time point of inoculation is also plotted. White bars denote VCD of CTE-cultures, Grey bars denote VCD of STE-cultures, White solid circle with dotted lines denote Viability of CTE-cultures, Grey solid circle with dotted lines denote Viability of STE-cultures. **b** Fold change in CTE IgG titers: fold change of IgG titers in CTE cultures. The IgG titers of day 2 (grey bars) and day 3 (black bars) were normalized w.r.t control day 1 levels. White bars denote day 1 levels. **c** IgG productivity profile of CTE-cultures: comparison of per-cell productivity in pg/cell-day of CTE-cultures treated with varying concentrations of tunicamycin (30 – 500 ng/mL). White bars denote day 1, grey bars denote day 2 and black bars denote day 3 q_P_ levels. **d** Fold change in STE IgG titers: fold change of IgG titers in STE cultures. Ratios are calculated w.r.t control day 1 levels. White bars denote day 1, Grey bars denote day 2, Black bars denote day 3 fold-change. **e** IgG productivity profile of STE-culture: comparison of per-cell productivity in pg/cell-day of STE-cultures when treated with tunicamycin (30 ng/mL – 500 ng/mL). White bars denote q_P_ at day 1, grey bars at day 2 and black bars at day 3. **f** Productivity profile of Long term adapted cultures: Comparison of productivity profile of control and long term tunicamycin adapted cultures in a batch mode. These long term adapted cells were obtained by culturing CHO cells in presence of 30 ng/mL tunicamycin for over 25 passages. Solid white circle with black line denote control productivity of control cultures and solid black squares with black line denote q_P_ of tunicamycin adapted cells. **g** Calreticulin/CRT mRNA fold change: comparison of Calreticulin mRNA fold change w.r.t respective control for CTE-and STE-cultures when treated with 30 and 500 ng/mL of tunicamycin.White bars denote CTE-cultures harvested post 24 hrs of tunicamycin treatment. Black bars denote STE-cultures harvested post 24 hrs culturing in fresh media. **h** UPR mRNA (XBP1s, Grp78, CHOP) fold change: Comparison of XBP1s. Grp78 and CHOP mRNA fold change w.r.t respective control for CTE-and STE-cultures at 30 and 500 ng/mL of tunicamcyin. White solid bars denote XBP1s levels of CTE-cultures, Grey solid bars denote XBP1s levels of STE-cultures, White solid bars with black horizontal lines denote Grp78 levels of CTE-cultures, Grey solid bars with black horizontal lines denote Grp78 levels of STE-cultures, White solid bars with black bubble pattern denote CHOP levels of CTE-cultures, Grey solid bars with black bubble pattern denote CHOP levels of STE-cultures. **i** IgG Heavy Chain mRNA fold change: comparison of IgG Heavy chain mRNA fold change wrt respective control for CTE-and STE-cultures at 30 and 500 ng/mL. White bars denote CTE-cultures harvested post 24 hrs of tunicamycin treatment. Black bars denote STE-cultures harvested post 24 hrs culturing in fresh media

## P-325 Enhancement of antibody productivity in recombinant CHO cells constructed by targeting the IgG1 gene to the stable chromosome

### Wataru Tanaka^1^, Kota Yoshitomi^1^, Noriko Yamano^1,2^, Masayoshi Onitsuka^2,3^, Yuichi Koga^1^, Takeshi Omasa^1,2^

#### ^1^Graduate School of Engineering, Osaka University, Suita, Osaka 5650871, Japan; ^2^Manufacturing Technology Association of Biologics, Kobe, Hyogo 6500047, Japan; ^3^Graduate School of Technology, Industrial and Social Sciences, Tokushima University, Tokushima, Tokushima 7708513, Japan

##### **Correspondence:** Takeshi Omasa (omasa@bio.eng.osaka-u.ac.jp)

**Background**

Chinese hamster ovary (CHO) cells have been widely used for the large-scale production of biopharmaceuticals [1]. To construct antibody-producing CHO cells, exogenous genes encoding antibodies are usually integrated into unspecified regions of chromosomes (random integration). However, the chromatin structure differs depending on the location of the chromosomal region, which affects the expression level of the gene of interest [2]. Recently, gene-targeting methods that enable site-specific integration of expression vectors have been developed. However, the regions that are most efficient for exogenous gene expression have not been clarified. We previously constructed a CHO genomic bacterial artificial chromosome (BAC) library generated from the recombinant CHO-DG44 cell line. It was expected to cover the entire CHO genome five times. The 20 chromosomes in CHO-DG44 cells were aligned in decreasing order of size and assigned letters from A to T [3]. Three hundred and four BAC clones were mapped to every chromosome of CHO-DG44. Among the karyotypes of CHO-DG44, CHO-K1 and primary Chinese hamster cells, chromosomes A and B are considered as the sole paired chromosomes corresponding to chromosome 1 in primary Chinese hamster cells. Hence, chromosomes A and B are considered to be stable [4]. In this study, we constructed antibody-producing cells by using a gene-targeting method, which focused on the stable chromosomes.

**Materials and method**

A gene map of chromosome 1 was constructed by combining the BAC-fluorescence *in situ* hybridization (FISH)-based chromosome physical map and sequence data of mapped BAC clones. The sequences of BAC clones were searched by BLAST with NCBI and CHOgenome.org databases. Three different regions on chromosomes A and B were selected based on CHO genomic BAC library sequences as target sites. CHO-K1 cells were stably transfected by lipofection. The target sequences were broken using the clustered regularly interspaced short palindromic repeats (CRISPR)/CRISPR-associated protein 9 (Cas9) system and humanized IgG1 genes were integrated by non-homologous end joining recombination. Transfection without using the CRISPR/Cas9 system was also performed. These cell pools were cultivated for six days with serum-supplemented medium, and their levels of antibody productivity were evaluated by ELISA. Copy number analysis was also performed using real-time PCR.

**Results and discussion**

**Construction of gene map of chromosome 1:** Eighty-three BAC clones were mapped onto chromosomes A and B (each clone contained 100–150 kb of the CHO genome sequence). As a result of annotations of 83 BAC clone sequences, 91 genes were mapped on chromosome 1.

**Investigation of the differences of productivity among antibody-producing cells that were constructed by chromosome 1 targeting and/or random integration:** Cell growth was not affected by the gene targeting site. The specific production rates of antibody-producing cell pools constructed by gene targeting of chromosome 1 were higher than those of the cell pool constructed by random integration. All cell pools constructed by gene targeting showed lower copy numbers of heavy chain and light chain in genomic DNA than those in the cell pool constructed by random integration, despite showing high productivity.

**Conclusion**

Our results indicate that high productivity of the cells constructed by gene targeting of chromosome 1 does not depend on the increase of the antibody copy number, and that the environments around these target regions are suitable for exogenous gene expression. The approach of using gene targeting to chromosome 1 may be promising for constructing antibody-producing cells.

**Acknowledgements**

This work was partially supported by the Project Focused on Developing Key Technology of Discovering and Manufacturing Drugs for Next-generation Treatment and Diagnosis from the Ministry of Economy, Trade and Industry, Japan (METI), and by Grants-in-aid for Scientific Research from the Japan Society for the Promotion of Science (JSPS; JP26630433, JP26249125 and JP17H06157).

**References**

1. Wurm FM: **Production of recombinant protein therapeutics in cultivated mammalian cells.** *Nat Biotechnol* 2004, **22 (11)**: 1393-1398.

2. Wilson C, Bellen HJ, Gehring WJ: **Position effects on eukaryotic gene expression.** *Annu Rev Cell Biol* 1990, **6**: 679–714.

3. Omasa T, Cao Y, Park JY, Takagi Y, Kimura S, Yano H, Honda K, Asakawa S, Shimizu N, Ohtake H: **Bacterial artificial chromosome library for genome-wide analysis of Chinese hamster ovary cells.** *Biotechnol Bioeng* 2009, **104 (5)**: 986-994.

4. Cao Y, Kimura S, Itoi T, Honda K, Ohtake H, Omasa T: **Construction of BAC-based physical map and analysis of chromosome rearrangement in Chinese hamster ovary cell lines.** *Biotechnol Bioeng* 2012, **109 (6)**: 1357-1367.

## P-327 Development of retroviral vectors capable of site-specific gene insertion together with protein delivery

### Yoshinori Kawabe^1^, Takuya Shimomura^1^, Shuohao Huang^2^, Suguru Imanishi^1^, Akira Ito^1^, Masamichi Kamihira^1,2^

#### ^1^Department of Chemical Engineering, Faculty of Engineering, Kyushu University, Fukuoka, 819-0395, Japan; ^2^Graduate School of Systems Life Sciences, Kyushu University, Fukuoka, 819-0395, Japan

##### **Correspondence:** Masamichi Kamihira (kamihira@chem-eng.kyushu-u.ac.jp)

**Background**

Retroviral vectors have been widely used as gene delivery tools in various biotechnology fields. However, the random integration feature of retroviral vectors seems to cause problems such as insertional mutagenesis and gene silencing. We previously demonstrated Cre-mediated retroviral transgene insertion into a pre-determined site of the founder cells using integrase-defective retroviral vectors (IDRVs), where a Cre expression plasmid was transfected into the cells prior to retroviral transduction [1]. Recently, we reported novel hybrid IDRVs (Cre-IDRVs) incorporating bioactive Cre recombinase protein, and validated site-specific gene integration of an scFv-Fc antibody expression unit into the Chinese hamster ovary (CHO) cell genome [2]. We also developed an accumulative site-specific gene integration system, which enables repeated integration of multiple transgenes into a pre-determined locus of the cell genome [3]. Here, we attempted repeated integration of transgenes using Cre-IDRVs.

**Materials and methods**

A viral vector plasmid (pQMSCV/HD[scFv-Fc]) encoding reporter genes and an scFv-Fc expression unit flanked with wild-type and mutant *loxP*s was constructed for the production of IDRVs. Cre-IDRVs were produced described previously [2]. CHO cells (CHO/NE[scFv-Fc]×1 #2) introducing reporter genes (*ATG-deleted-Neo*^*r*^*/IRES/EGFP*) and an scFv-Fc expression unit flanked with a compatible pair of *loxP*s were used as founder cells for second round of site-specific integration. Viral solution (8.0 × 10^10^ copies/well, MSCV/HD[scFv-Fc]) was infected to CHO/NE[scFv-Fc]×1 cells (2.5 × 10^4^ cells/well). The cells were screened for 7–10 days in the presence of 200 μg/mL hygromycin B. Formed colonies were observed under a fluorescence microscope. Clones were isolated by the colony picking method. Genomic DNA extracted from cells was subjected to genomic PCR analysis.

**Results and discussion**

Figure 1a shows a schematic drawing of each round of targeted transgene integration using Cre-IDRVs harboring an scFv-Fc expression unit. We previously established recombinant CHO cells (CHO/NE[scFv-Fc]×1) expressing reporter (Neo^r^ and EGFP) and scFv-Fc proteins using Cre-IDRV produced by pQMSCV/NE[scFv-Fc] [2]. For the second Cre-RMCE reaction, we constructed a viral vector plasmid (pQMSCV/HD[scFv-Fc]) encoding marker genes (*ATG-deleted Hyg*^*r*^*/IRES/DsRed*) and an scFv-Fc expression unit flanked with corresponding *loxP* sites. After the second round of Cre-mediated integration between circular retroviral DNA derived from IDRV and CHO/NE[scFv-Fc]×1 cell genome, *Neo*^*r*^ expression was replaced with *Hyg*^*r*^ expression, and red fluorescent protein was expressed in the cells. The expected structures of the transgene after Cre-RMCE reaction between IDRV and cell genome are also shown in Fig. 1a. After CHO/NE[scFv-Fc]×1 cells were infected with Cre-IDRV produced using pQMSCV/HD[scFv-Fc], two cell colonies were isolated using hygromycin screening (CHO/HD[scFv-Fc]×2). The cells expressed DsRed (Fig. 1b). Genomic DNA extracted from the cells were subjected to PCR using specific primer pairs α and β, and γ and δ to confirm site-specific integration. DNA fragments with expected sizes were amplified in each cell clone (Fig. 1c). These results indicate that site-specific repeated integration was achieved using Cre-IDRVs. In contrast, scFv-Fc productivity in CHO/HD[scFv-Fc]×2 cells was slightly decreased compared with that of CHO/NE[scFv-Fc]×1 (data not shown). Although the reason remains unclear, repeat-induced gene silencing might occur due to tandem repeat structure of expression units. We reported improved recombinant antibody production using a production enhancer element [4]. Such a *cis*-regulatory element might be a feasible approach to enhance the productivity.

**Conclusions**

We demonstrated site-specific repeated transgene integration into a pre-determined chromosomal locus using Cre-IDRVs for the production of an scFv-Fc antibody.

**Acknowledgements**

This work was supported in part by grants for developing key technologies for discovering and manufacturing pharmaceuticals used for next-generation treatment and diagnoses, both from the Ministry of Economy, Trade and Industry (METI), Japan and from the Japan Agency for Medical Research and Developments (AMED).

**References**

1. Huang S, Kawabe Y, Ito A, Kamihira M: **Cre recombinase-mediated site-specific modification of a cellular genome using an integrase-defective retroviral vector.** *Biotechnol Bioeng* 2010, **107:**717–-729.

2. Kawabe Y, Shimomura T, Huang S, Imanishi S, Ito A, Kamihira M: **Targeted transgene insertion into the CHO cell genome using Cre recombinase-incorporating integrase-defective retroviral vectors.** *Biotechnol Bioeng* 2016, **113:**1600–1610.

3. Kameyama Y, Kawabe Y, Ito A, Kamihira M: **An accumulative site-specific gene integration system using Cre recombinase-mediated cassette exchange.** *Biotechnol Bioeng* 2010, **105:**1106–1114.

4. Kawabe Y, Inao T, Komatsu S, Huang S, Ito A, Omasa T, Kamihira M: **Improved recombinant antibody production by CHO cells using a production enhancer DNA element with repeated transgene integration at a predetermined chromosomal site.** *J Biosci Bioeng* 2017, **123:**390–397.

**Fig. 1 (abstract P-327).**
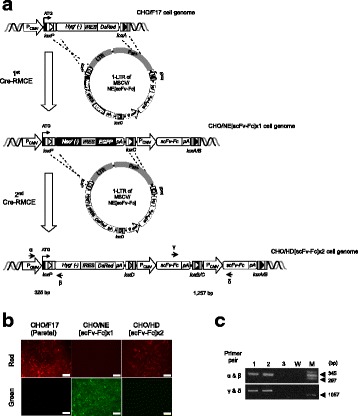
Site-specific repeated transgene integration for CHO cells using Cre-IDRVs. **a** Schematic drawing of Cre-RMCE using Cre-IDRVs. **b** Fluorescent microscope images. Scale bars; 100 μm. **c** Genomic PCR analysis. Lanes 1 and 2, established clones; Lane 3, CHO/NE[scFv-Fc]×1; Lane W, H2O; Lane M, molecular weight standard markers (mix of λ-HindIII and ΦX174-HindII digests)

## P-330-B Lipidomics: challenges, techniques, and futures possibilities for mammalian cell culture

### Andréa McCann, Gregory Mathy, Laetitia Malphettes

#### UCB pharma, Braine l’Alleud, Belgium

##### **Correspondence:** Andréa McCann (andrea.mccann2@ucb.com)

**Background**

If lipids role in the cell have been reduced for a long time to cell membrane formation, it is now understood that lipids plays also a role into energy metabolism, vesicular transport, membrane structure, dynamics and signaling. However, the exact mechanism of how compositional complexity affects cell homeostasis remains unclear. Thanks to recent advances in mass spectrometry, it is now possible to study a wide range of lipids, providing a better understanding of lipid homeostasis in high performance cell culture processes.

**Materials and methods**

The purpose of this work was to develop a robust lipidomics method applied to mammalian cell cultures in a three step method: extraction, separation and detection (Fig. 1). Both Matyash [1] and Folch [2] extraction method were performed on our cells to reach the highest yield. Two separation techniques were also tested: hydrophilic interaction liquid chromatography (HILIC) and reverse phase chromatography. Finally lipid classes’ identification was achieved by tandem mass spectrometry analysis thanks to structure-specific fragmentation ions.

**Results**

The yield obtained with Matyash extraction method was higher than with Folch method for each lipid class tested. Besides, Matyash method presents also the advantage to be less toxic and suitable for high throughput analysis since the organic layer is above the aqueous layer.

Lipids separation by HILIC is based on their polar head. Since lipid classes are defined by polar head, the lipids are eluted class by class, making their identification easier. The separation of lipids by reverse phase was correct but the method is longer and we observed a massive carryover of triglycerides on the column.

Finally each lipid class was screened in MS/MS parent ion mode. Target daughter ion was set according to the lipid class structure and fragmentation pattern. This detection technique enabled the identification of 50 different lipids. To ensure the absolute quantification of the detected lipids and to guarantee comparable results between batches labeled internal standard were added prior to extraction.

**Conclusion**

This method was optimized in a stepwise process to ensure a sensitive and selective measurement of the lipids. Lipids were extracted by Matyash method, separated by HILIC and detected by tandem mass spectrometry. This method is suitable for both in process sample lipid analysis providing information on the cell lipid content, and for harvest samples, enabling to follow the lipid release during the different harvest steps.

This non-targeted lipidomic quantitation method will enable us to better control lipid synthesis during biopharmaceutical fed batch production through clone selection, metabolomics studies and harvest development.

**Acknowledgments**

Many thanks Stefanos Grammatikos for his support and to the whole Upstream Process Sciences team.

**References**

1. Matyash V, Liebisch G, Kurzchalia T V, Shevchenko A, Schwudke D: **Lipid extraction by methyl-tert-butyl ether for high-throughput lipidomics** J Lipid Res. 2008, **49**(5):1137-46.

2. Folch, J, Lees M, Sloane Stanley G H: **A simple method for the isolation and purification of total lipids from animal tissues.** J Biol Chem. 1957, **226**(1): 497-509.

**Fig. 1 (abstract P-330-B).**
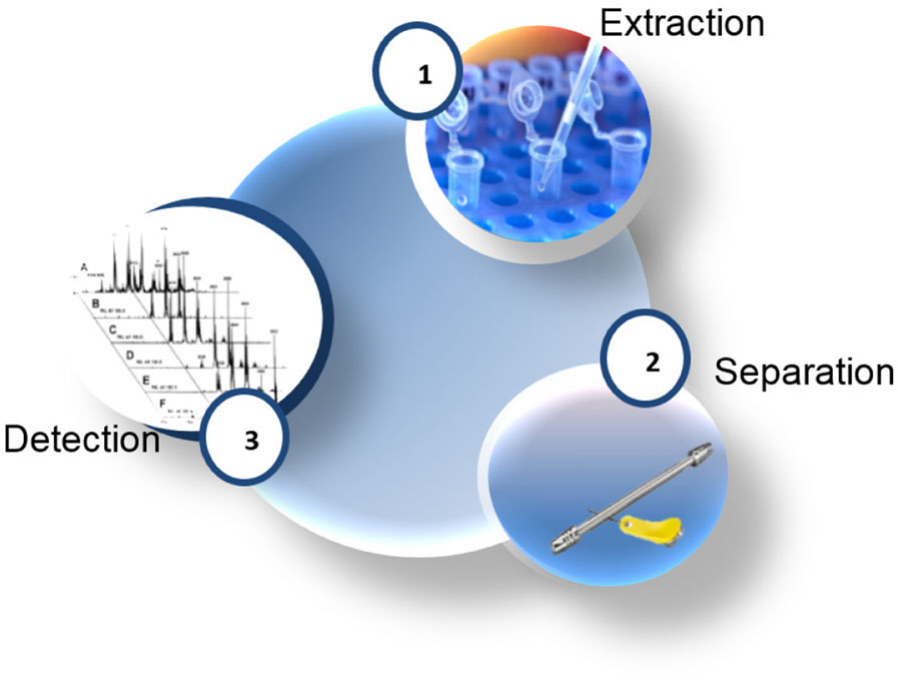
Lipid detection method workflow

## P-339 Using Baculovirus as a gene shuttle in hMSC: optimization of transduction efficacy

### Gundula Sprick^1^, Tobias Weidner^2^, Peter Czermak^1,2,3,4^

#### ^1^Institute of Bioprocess Engineering and Pharmaceutical Technology, University of Applied Sciences Mittelhessen, Wiesenstr. 14, 35390 Giessen, Germany; ^2^Fraunhofer Institute for Molecular Biology and Applied Ecology (IME), Project group Bioresources, Winchester Str. 2, 35394 Giessen, Germany; ^3^Faculty of Biology and Chemistry, Justus Liebig University, Ludwigstr. 23, 35390 Giessen, Germany; ^4^Department of Chemical Engineering, Kansas State University, 1005 Durland Hall, Manhattan, KS 66506, USA

##### **Correspondence:** Gundula Sprick

**Background**

Human mesenchymal stem/stromal cells (hMSC) can easily be isolated from e.g. bone marrow, fat tissue or umbilical cord blood and are therefore a central player in regenerative medicine, gene therapy and cell therapy [1–3]. The necessary gene shuttle is mainly provided by viruses associated with diseases, like retrovirus or adenovirus [4–7]. These possible pathogen viruses demand for high safety standards. Also, they are prone to genomic alterations and there is the possibility of virus inactivation, triggered due to pre-existing immunity in the patient [8–10].

In this context, the *Autographa californica* multicapsid nucleopolyhedrovirus (*Ac*MNPV) is a safe alternative. The virus replication is host-specific for insects [11], but it is known since the mid-90s, that a temporary transduction of mammalian cells is possible [12]. Some modifications of the virus increased the applicability in stem cells. Pseudotyping the virus with the vesicular stomatitis glycoprotein (VSV-G) led to an expansion of the transducable cell [13,14] and the integration of the woodchuck hepatitis virus post-transcriptional regulatory element (WPRE) prolonged the recombinant protein expression [15,16].

For achieving a baculovirus-induced differentiation of hMSCs, the promotor and the expression strength of the recombinant protein are crucial factors. Still, there are still few comparative promotor studies [17,18]. However, a successful virus uptake is the prerequisite for a successful protein expression. We therefore investigated factors significantly influencing the transduction process by applying design of experiments (s. Fig. 1a).

**Materials and methods**

The experimental design comprises a two level factorial screening, set-up using Design Expert V9.For the transduction 60,000 c/cm^2^ were seeded in 24-well plates with DMEM + 10% FCS and incubated overnight at 37°C, 8% CO_2_ and humidified atmosphere. The recombinant baculovirus using an integrated EF1α promoter to control GFP expression, described elsewhere [18], was diluted to the respective concentrations in the different surrounding fluids. After discarding the cultivation medium of the hMSC-TERT, 1 mL of virus containing solution was added to the cells. The following incubation was varied in duration before replacing the virus solution with growth medium and an incubation overnight. 24 h post transduction (hpt) the cells were washed with PBS, trypsinized with 100 μL Trypsin/EDTA and incubated for 5 min at 37°C. Trypsination was then stopped applying 100 μL soybean trypsin inhibitor and the cells were analyzed using flow cytometry.

**Results**

As shown in Fig. 1a, the virus concentration and incubation time exert the highest influence on the transduction efficiency. Obviously, a higher concentration of viral particles and longer incubation of cells with virus increases the probability for hits between cells and virus particles. Additionally, the surrounding fluid can have a negative impact on the transduction. This is due to the interaction of medium components with the baculovirus. Therefore, PBS containing Ca^2+^ & Mg^2+^ is recommended as surrounding fluid for transduction experiments. In Fig. 1B, the transduction conditions resulting in the highest percentage of GFP+ cells are displayed: 150 virus particles per cell (PPC) and an incubation time of 5 h with hMSC-TERT.

**Conclusion**

The experiments show, that especially the virus concentration and the incubation time of cells with virus influence the transduction efficiency. Based on the results of the screening, further optimization of the transduction conditions will be done using a face centered central composite design with PBS containing Ca^2+^ & Mg^2+^ as surrounding fluid and at an incubation temperature of 37°C.

**Acknowledgements**

The authors thank the Hessen State Ministry of Higher Education, Research and the Arts within the Hessen initiative for scientific and economic excellence (LOEWE Program) for the financial support.

**References**

1. Kim N, Cho S-G. Clinical applications of mesenchymal stem cells. Korean J Intern Med 2013;28:387–402. doi:10.3904/kjim.2013.28.4.387.

2. Volarevic V, Arsenijevic N, Lukic ML, Stojkovic M. Concise review: Mesenchymal stem cell treatment of the complications of diabetes mellitus. Stem Cells 2011;29:5–10. doi:10.1002/stem.556.

3. McGuirk JP, Smith JR, Divine CL, Zuniga M, Weiss ML. Wharton’s Jelly-Derived Mesenchymal Stromal Cells as a Promising Cellular Therapeutic Strategy for the Management of Graft-versus-Host Disease. Pharmaceuticals (Basel) 2015;8:196–220. doi:10.3390/ph8020196.

4. Verma IM, Weitzman MD. Gene therapy: twenty-first century medicine. Annu Rev Biochem 2005;74:711–38. doi:10.1146/annurev.biochem.74.050304.091637.

5. Kay MA. State-of-the-art gene-based therapies: the road ahead. Nat Rev Genet 2011;12:316–28. doi:10.1038/nrg2971.

6. Vannucci L, Lai M, Chiuppesi F, Ceccherini-Nelli L, Pistello M. Viral vectors: a look back and ahead on gene transfer technology. New Microbiol 2013;36:1–22.

7. Balakrishnan B, Jayandharan G. Basic Biology of Adeno-Associated Virus (AAV) Vectors Used in Gene Therapy. Curr Gene Ther 2014;14:86–100. doi:10.2174/1566523214666140302193709.

8. Rothe M, Modlich U, Schambach A. Biosafety challenges for use of lentiviral vectors in gene therapy. Curr Gene Ther 2013;13:453–68. doi:10.2174/15665232113136660006.

9. Castro MG, Candolfi M, Wilson TJ, Calinescu A, Paran C, Kamran N, et al. Adenoviral vector-mediated gene therapy for gliomas: coming of age. Expert Opin Biol Ther 2014;14:1241–57. doi:10.1517/14712598.2014.915307.

10. Merten O-W, Schweizer M, Chahal P, Kamen AA. Manufacturing of viral vectors for gene therapy: part I. Upstream processing. Pharm Bioprocess 2014;2:183–203. doi:10.4155/pbp.14.16.

11. Ayres MD, Howard SC, Kuzio J, Lopez-Ferber M, Possee RD. The complete DNA sequence of Autographa californica nuclear polyhedrosis virus. Virology 1994;202:586–605. doi:10.1006/viro.1994.1380.

12. Hofmann C, Sandig V, Jennings G, Rudolph M, Schlag P, Strauss M. Efficient gene transfer into human hepatocytes by baculovirus vectors. Proc Natl Acad Sci U S A 1995;92:10099–103.

13. Barsoum J, Brown R, McKee M, Boyce FM. Efficient Transduction of Mammalian Cells by a Recombinant Baculovirus Having the Vesicular Stomatitis Virus G Glycoprotein. Hum Gene Ther 1997;8:2011–8. doi:10.1089/hum.1997.8.17-2011.

14. Kost T a, Condreay JP. Recombinant baculoviruses as mammalian cell gene-delivery vectors. Trends Biotechnol 2002;20:173–80.

15. Mähönen AJ, Airenne KJ, Purola S, Peltomaa E, Kaikkonen MU, Riekkinen MS, et al. Post-transcriptional regulatory element boosts baculovirus-mediated gene expression in vertebrate cells. J Biotechnol 2007;131:1–8. doi:10.1016/j.jbiotec.2007.05.022.

16. Zeng J, Du J, Zhao Y, Palanisamy N, Wang S. Baculoviral Vector-Mediated Transient and Stable Transgene Expression in Human Embryonic Stem Cells. Stem Cells 2007;25:1055–61. doi:10.1634/stemcells.2006-0616.

17. Qin JY, Zhang L, Clift KL, Hulur I, Xiang AP, Ren B-Z, et al. Systematic comparison of constitutive promoters and the doxycycline-inducible promoter. PLoS One 2010;5:e10611. doi:10.1371/journal.pone.0010611.

18. Sprick G, Weidner T, Salzig D, Czermak P. Baculovirus-induced recombinant protein expression in human mesenchymal stromal stem cells: A promoter study. N Biotechnol 2017. doi:10.1016/j.nbt.2017.08.006.

**Table 1 (abstract P-339).** Factors, their type and the range used to identify factors significantly influencing the transduction of hMSC-TERT with pseudotyped Baculoviruses

Factor	Type	Range
Virus concentration	Numeric	10 – 150 PPC
Incubation time	Numeric	1 – 5 h
Incubation temperature	Categoric	27°C, 37°C
Surrounding fluid	Categoric	PBS with Ca^2+^ & Mg^2+^, DMEM

**Fig. 1 (abstract P-339).**
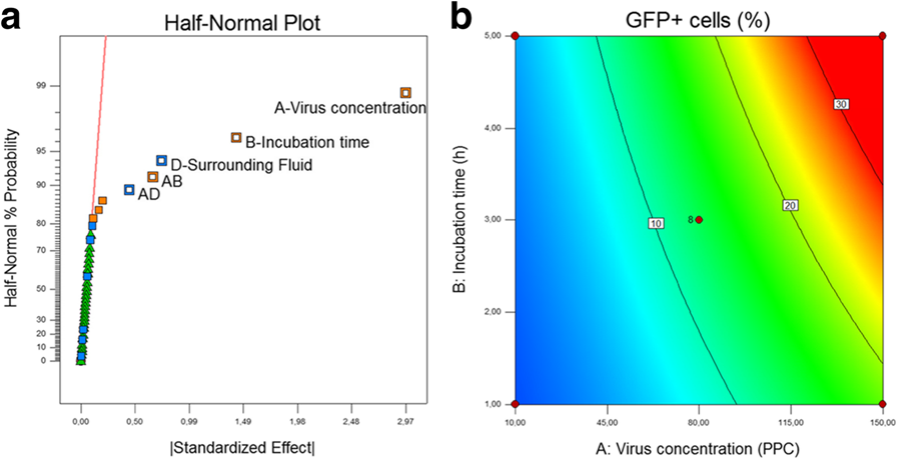
**a** The half-normal plot shows factors significantly influencing the transduction of hMSC-TERT with pseudotyped baculovirus. **b** Optimal parameters for transduction of hMSC-TERT are 150 virus particles per cell (PPC), incubated with hMSC-TERT for 5 h at 37°C with PBS containing Ca^2+^ & Mg^2+^ as surrounding fluid

## P-340 Generation of a function blocking antibody against Notch Delta-like-1 with therapeutic efficacy against breast cancer

### Joana Sales Dias^1^, Márcia Lamy^1^, Andreia Ferreira^1^, Gabriela Silva^1^, Tiago Bandeiras^1,2^, Ana Barbas^1,3^

#### ^1^IBET, Oeiras, Portugal; ^2^ITQB, Oeiras, Portugal; ^3^Bayer, Lisboa, Portugal

##### **Correspondence:** Joana Sales Dias (ab@ibet.pt)

**Background**

Breast cancer is the second main cause of cancer related deaths for women worldwide and among them the triple negative subtype (TNBC) represents a clinical challenge by being associated with high mortality and having no effective therapies against it [1], [2]. Accordingly, there is an urgent need to design new and more effective drugs to treat breast cancer. Notch signaling is an evolutionary conserved cell-to-cell communication pathway crucial during embryonic and breast development and tissue homeostasis. This pathway is often hyper-activated by overexpression of Notch receptors and/or its ligands in several types of cancers, such as breast cancer (TNBC included), where it contributes to its development, progression and drug resistance [3], [4], [5]. Our aim is to generate a function blocking antibody against the Notch Delta-like-1 (DLL1) ligand with therapeutic efficacy against breast cancer.

**Materials and methods**

DNA of human DLL1 full length extracellular domain (DLL1-ECD) and a truncated version, containing the minimal binding region to the Notch receptor (DLL1-EGF3), were cloned into pFUSE-Fc1-IgG1, and expressed in HEK293E6cells. Recombinant proteins were purified from culture media by Protein-A affinity and size exclusion chromatography. The human scFv phage display Tomlinson I+J library was used to select specific scFv against peptides targeting DLL1 binding regions to Notch. The binding ability and specificity of the selected scFv clones was evaluated by *scFv-on-Phage* ELISA.

**Results**

Our strategy allowed us to obtain 20 mg of pure (>95%) and stable DLL1-ECD-FC as confirmed by SDS PAGE and thermofluor assay. DLL1-EGF3-FC yield was very low and buffer screenings are ongoing to optimize protein stability. Functional studies performed in human breast cancer MCF7 cells showed that both ligands are biologically active as they increased the expression of the Notch-dependent genes HES-1, HEY-L and HEY-1. Recombinant DLL1 and peptides were used to select for monoclonal antibodies by Phage Display. After three rounds of panning with DLL1 peptides we identified 13 scFv positive clones, 2 of which presented high affinity to DLL1-ECD-FC. Currently we are performing more phage display selections to increase the number of positive clones. scFv with higher affinities will be reformatted into IgGs and their ability to inhibit the Notch pathway will be evaluated. The anti-oncogenic effects of anti-DLL1 IgGs will be assessed in breast cancer cells in viability/apoptosis, proliferation, migration, and invasion assays.

**Conclusions**

An anti-DLL1 IgG with therapeutic efficacy against breast cancer will demonstrate that targeting DLL1 could be one of the key factors for successfully targeting breast cancer.

**Acknowledgements**

iBET; iNOVA4Health (LISBOA-01-0145-FEDER-007344); FCT: PTDC/SAUONC/121670/2010, PTDC/BBB-BMD/4497/2014, PD/BD/113987/2015

**References**

1. Hudis CA and Gianni L. Triple-Negative Breast Cancer: An Unmet Medical Need. Oncologist. 2011. 16(Suppl 1): p. 1-11.

2. Ecker DM, Jones SD, Levine HL. The therapeutic monoclonal antibody market. mabs, 2015. 7(1): p. 9-14.

3. Sharma A, Paranjape AN, Rangarajan A, Dighe RR. A monoclonal Antibody against Human Notch1 Ligand-Binding Domain Depletes Subpopulation of Putative Breast Cancer Stem-like Cells. Mol Cancer Ther. 2011. 11(1): p. 77-86.

4. Bolós V, Mira E, Martínez-Poveda B, Luxán G, Cañamero M, Martínez-A C, Mañes S, de la Pompa JL. Notch activation stimulates migration of breast cancer cells and promotes tumor growth. Breast Cancer Res. 2013. 15(4): p. R54.

5. Lamy M, Ferreira A, Dias JS, Braga S, Silva G, Barbas A. Notch-out for breast cancer therapies. N Biotechnol. 2017 Epub ahead of print.

## P-349 AAV production in suspension: evaluation of different cell culture media and scale-up potential

### Rebecca C. Feiner, Kathrin Teschner, Irina Schierbaum, Julian Teschner, Kristian M. Müller

#### Cellular and Molecular Biotechnology, Bielefeld University, 33602 Bielefeld, Germany

##### **Correspondence:** Kristian M. Müller (kristian@syntbio.net)

**Background**

Recombinant adeno-associated virus (rAAV) approaches have an outstanding reputation in gene therapy and are evaluated for cancer therapy [1]. Advantages include long-term gene expression, targeting of dividing and non-dividing cells, and low immunogenicity. Established rAAV production utilizes triple transfection of adherent HEK 293 cells, which hardly meets product yield requirements for clinical applications. We transferred the AAV production system to HEK 293-F suspension cells. This process is scalable and uses serum-free media streamlining downstream procedures. After optimization of transfection efficiencies and shaker cultivations, we produced titers of 1×10^5^ viral genomes per cell in a 2 l bioreactor.

**Materials and methods**

The suspension adapted HEK-FreeStyle 293-F cell line was used for the experiments in chemically defined animal component free media (HEK-TF, HEK-GM (Xell AG), Freestyle F17 (Thermo Fisher Scientific)). Samples for viable cell density and viabilities were taken daily and analyzed using an automated cell counting system (CEDEX, Roche Diagnostics). Transient transfection of 3×10^6^ cells/ml was carried out with polyethylenimine Max in a 1:4 DNA-PEI ratio (w/w) with 2 μg DNA. Three plasmids (pGOI, pRepCap, pHelper) were applied in a molar 1:1:1 ratio (Fig. 1a). Pretests were performed in orbital shaking tube spin bioreactors. For scale-up, batch processes were carried out in 125 ml shake flasks as well as in 2 l stirred bioreactors at 30% air saturation and pH 7.1. Transfection efficiencies and rAAV production were quantified by flow cytometry using a GOI coding for a fluorescent protein and qPCR of genomic copies, respectively.

**Results**

By optimizing the DNA amount for transfection of 293-F cells more than 90 % of the cells were reproducibly transfected. Batch cultivations in shaker flasks revealed that rAAV were produced in the first 24-96 h after transfection. Figure 1b shows viable cell densities and viabilities in relation to the genomic titer. Genomic titers were determined from raw cell extracts and up to 10^9^ copies/ml were repetitively achievable. A decrease in viability marked the decline in genomic copies per ml showing that a prolongation of the process e.g. by addition of a feed would probably not increase yield. In a first scale-up, the rAAV production was transferred to a 2 l bioreactor (Fig. 1c). Transfection efficiencies in bioreactors of up to 55% were comparable to that obtained in a simultaneous shaker flask experiment. Transfection efficiencies were lower compared to prior experiments due to controlled conditions in the bioreactor. Nonetheless the titer with up to 1×10^5^ genomic copies per cell was elevated compared to that of shaker flasks.

**Conclusions**

First experiments with 293-F cells in HEK TF medium showed promising results of transferring rAAV production from the adherent system to suspension. After improvement of transfections by the adjustment of DNA amounts in small scale experiments, AAV production was analyzed in shaker flasks. The batch process showed an expected increase in cell density with low variability between biological replicates (Fig. 1b). The genomic titer increased according to the viable cell density until day four where a sudden drop started. This observation was made for AAV productions in HEK-TF, HEK-GM and Freestyle F17 medium. For optimal yields, we assume that a slight decrease in viability marks the point in time for harvest. From optimized protocols, a batch process in a 2 l bioreactor was carried out. Interestingly the bioreactor cultivation resulted in lower overall viable cell densities but in higher genomic copies per cell compared to shaker flasks (Fig. 1c). These results are comparable to already published data for suspension cells [2]. Subsequent optimization of the bioreactor protocol will lead to further increase in rAAV yield.

**Acknowledgements**

The authors thank Xell AG, Bielefeld, for providing HEK serum-free media (HEK GM and HEK TF) and for fruitful discussions.

**References**

1. Hagen S, Baumann T, Wagner HJ, Morath V, Kaufmann B, Fischer A, et al. **Modular adeno-associated virus (rAAV) vectors used for cellular virus-directed enzyme prodrug therapy.** *Sci Rep*. 2014;**4**:3759.

2. Grieger JC, Soltys SM, Samulski RJ. **Production of Recombinant Adeno-associated Virus Vectors Using Suspension HEK293 Cells and Continuous Harvest of Vector From the Culture Media for GMP FIX and FLT1 Clinical Vector.** *Mol Ther*. 2016;**24**:287–97.

**Fig. 1 (abstract P-349).**
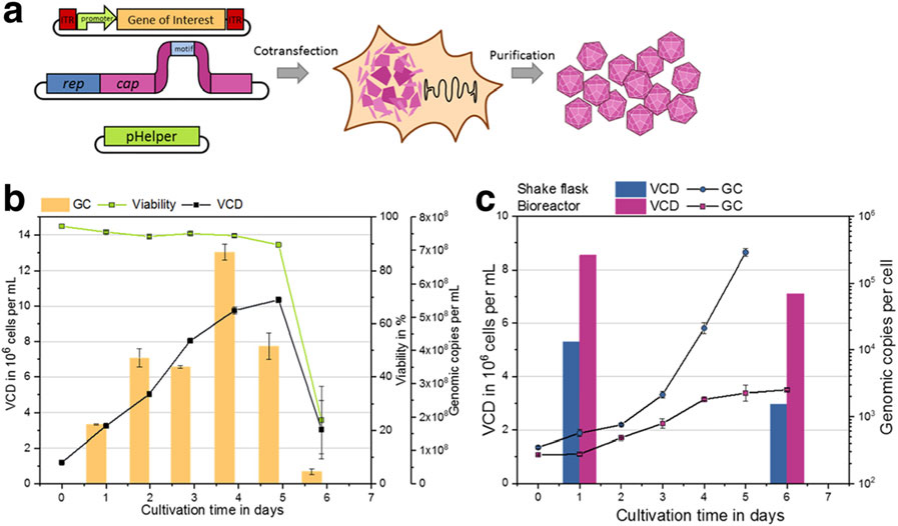
**a** Schematic overview of rAAV production in HEK293 cells with triple-transfection system. **b** Viable cell densities (VCD), viabilities and genomic copies per ml (GC) of a rAAV production with 293-F batch cultivations in shaker flasks. Genomic copies per ml refer to the titer determined in 1 ml culture volume. Error bars represent biological and technical duplicate measurements of samples. **c** Viable cell densities and genomic copies per cell of a rAAV production with 293-F batch cultivation in a 2 l bioreactor. For reasons of comparability between shaker and bioreactor data genomic copies are given per cell. Error bars represent technical duplicate measurements of samples

## P-350 Development of a cost-efficient scalable production process for RAAV-8 based gene therapy by transfection of Hek-293 cells

### Pascal Lefèbvre^1^, Christophe Van Huffel^1^, Otto-Wilhelm Merten^2^, Matthias Hebben^2^, Cédrick Rousseau^2^, Simon Arias^2^, Mustapha Hohoud^1^, Roel Lievrouw^1^, Fabien Moncaubeig^1^, Paule Nowicki^3^, Catherine Cancian^2^

#### ^1^Pall Artelis, Rue de Ransbeek 310, B-1120 Brussels, Belgium; ^2^Généthon, Rue de l’Internationale 1, BP 60, 91002 Evry, France; ^3^Aktehom, Avenue du Maréchal JOFFRE - 92000 Nanterre, France

##### **Correspondence:** Pascal Lefèbvre (pascal_lefebvre@europe.pall.com)

**Background**

Genethon and Pall have collaborated to assess Pall’s single-use iCELLis fixed-bed bioreactor for viral vector production. Clinical use of gene therapies to treat formerly incurable genetic diseases is advancing rapidly. Viral vectors are an important tool for introducing genes into target cells. Many gene therapies have been developed using adherent cells in 2-dimensional flatware or roller bottles but using these technologies to reach commercial-scale production represents a significant challenge.

The iCELLis bioreactor enables large-scale viral vector production by providing a 3-dimensional matrix for cell growth in a compact configuration (Fig. 1). Up to 500 m^2^ of surface area is available in a compact bioreactor measuring 88 mm in diameter in a total volume of 75 L with pH, DO and temperature control. A key feature of the iCELLis bioreactor is that it scales by increasing the diameter of the fixed-bed while keeping the height constant with no change in aspect ratios. The height of the fixed-bed can be varied (2, 4 and 10 cm) as well as density of carrier packing (96 gm/L or 144 gm/L). The iCELLis system comes in two formats, the iCELLis Nano bioreactor (0.53-4.0 m^2^) and the iCELLis 500 bioreactor (66-500 m^2^). Processes developed in the bench top iCELLis Nano bioreactor can be directly transferred to the corresponding iCELLis 500 system. The iCELLis Nano bioreactor enables an efficient platform for process optimization.

**Materials and methods**

Materials

- HEK-293 cells (Généthon).
- iCELLis Nano bioreactors 0.8 m^2^ (Pall, Part 810040NS) and 4 m^2^ (Pall, Part 810042NS).
- Growth medium: FreeStyle F17 Expression medium (Thermo Fisher, Part A13835-02) supplemented with 4 mM GlutaMAX^♦^ Supplement (Thermo Fisher, Part 35050-083).
- Transfection reagents: PeiPRO^♦^ transfection reagent (PolyPlus, Part 115-100) and mix of proprietary plasmid constructions: pGFP, pRep2Cap8 and pHelper (Genethon).
- Production medium: Dulbecco’s Modified Eagle Medium (DMEM, Thermo Fisher, Part 31053-028 supplemented with 4 mM GlutaMAX Supplement, Thermo Fisher, Part 35050-083).
- Lysis buffer: Triton X-100 solution (Merck Millipore, Part 1086432500) and NaCl solution (Sigma Aldrich, Part S9888). pH adjusted with NaOH 0.5 M (Merck Millipore 1.09137.2500).

Methods

The Genethon rAAV-8 process was transferred to an iCELLis Nano bioreactor 0.8 m^2^ (2 cm bed height, 144 gm/L density) bioreactor using FreeStyle media. The initial iCELLis Nano process was established as (1) seed on Day 1, (2) transfect at Day 5, (3) harvest at Day 8 and yielded <1x 10^9^ vp/cm^2^(n=3). Media exchange, cell density at transfection, pDNA/cell ratio, and lysis method were then changed to determine the effect on productivity. The modified process was then scaled from 0.8 m^2^ to 4.0 m^2^ (10 cm bed height, 144 gm/L density) iCELLis Nano bioreactor.

**Results**

- Media: A media exchange at 5 hours post transfection with DMEM substituted for FreeStyle medium resulted in an 8x increase in specific productivity.
- Cell density at transfection: Cells were seeded at 6,000 cells/cm^2^ and reached 200,000 cells/cm^2^ at Day 5 which was determined to be the optimal cell density for transfection.
- pDNA/cell ratio: Reducing pDNA by 50% had no significant effect on productivity.
- Lysis: Use of Trion x-100 at 0.5% with 100 mM NaCl at pH 8 resulted in >100% virus recovery compared to sampled carriers.
- Scaling: Specific productivity was maintained as the system was scaled from 0.8 m^2^ to 4.0 m^2^.
- Overall, an average yield of 4x10^13^ VG/m^2^ was achieved.

**Conclusions**

The iCELLis technology is being adopted widely for viral vector production. Transferring a process to the iCELLis Nano bioreactor can be easily achieved and once in place can be optimized to provide significant productivity increases and cost savings such as reduced pDNA. The iCELLis Nano bioreactor is an efficient bench-top system the results of which can be readily scaled to the iCELLis 500 system.

**Fig. 1 (abstract P-350).**
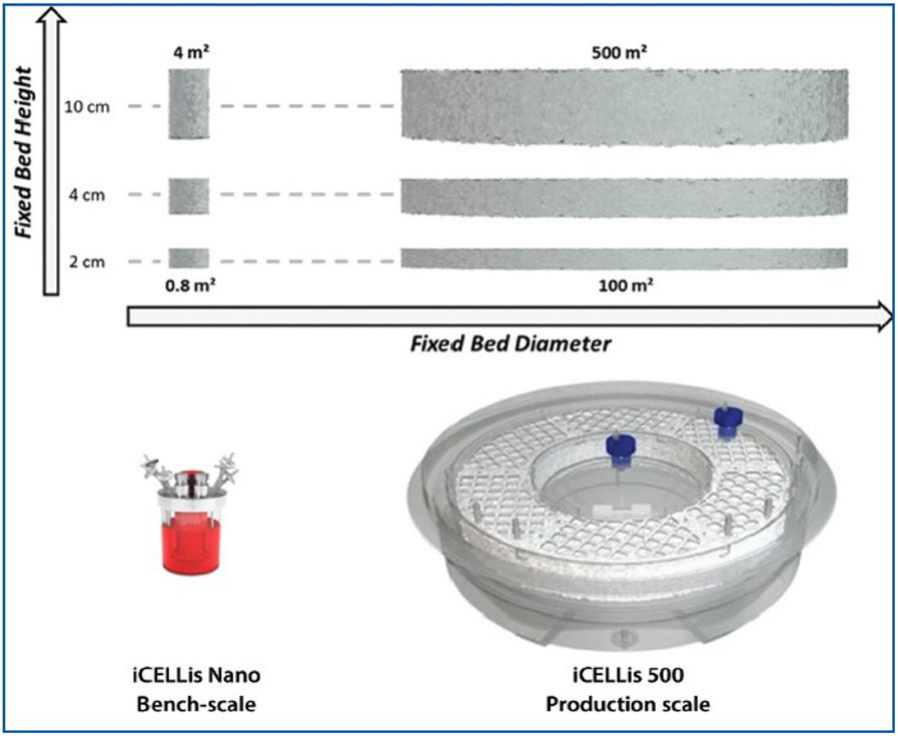
Linear scalability from iCELLis Nano bioreactor to iCELLis 500 bioreactor

## P-353 iPSC derived cardiomyocytes development for Multi-Organ-Chip cultivation

### Anja Ramme^1^, Eva Dehne^1^, Anna Krebs^1^, Roland Lauster^2^, Uwe Marx^1^

#### ^1^TissUse GmbH, Oudenarder Str. 16, 13347 Berlin, Germany; ^2^Technische Universität Berlin, Medizinische Biotechnologie, Gustav-Meyer-Allee 25, 13355 Berlin, Germany

##### **Correspondence:** Anja Ramme (anja.ramme@tissuse.com)

**Background**

TissUse Multi-Organ-Chip (MOC) platform contributes to the ongoing advancement in systemic substance testing in vitro. Current in vitro and animal tests for drug development are failing to emulate the systemic organ complexity of the human body and, therefore, often do not accurately predict drug toxicity. Especially, cardiotoxicity is one of the main reasons why new compounds are failing in clinical trials. Therefore, we aimed to establish an autologous dynamic multi-organ-device integrating cardiomyocytes for substance testing.

**Results**

Generic 2D monolayer and 3D suspension iPSC derived cardiomyocytes differentiation protocols were established. Beating cardiomyocytes were first seen on day 8 in monolayer as well as in spheroid culture. Cardiomyocytes show up to 64% cardiac troponin T positive cells and 44% myosin heavy chain positive cells by flow cytometry (Fig. 1g, h). Myosin II heavy chain, α-actinin, myosin 9/10, myosin 11 and caldesmon expression was shown by immunohistochemistry (Fig. 1a-d). Due to the exclusion of a lactate enrichment of cardiomyocytes, cardiac fibroblasts are also expressed in the spheroids shown by vimentin staining. Those cardiac fibroblasts lead to a physiological heterologous cell population similar to the human heart. Beating spheroids were cultivated for 7 days under dynamic culture conditions in the Multi-Organ-Chip. The integrated on-chip micropump provides physiological-like pulsatile circulation at a microliter scale and leads to better nutrition and oxygen supply.

**Conclusions**

The next significant step is to combine multiple autologous 3D organ equivalents in our Multi‑Organ‑Chip using iPSC differentiation technology. Differentiating all cell types from one iPSC donor is crucial to overcome source and rejection problems. Combining our Multi-Organ-Chip platform with iPSC differentiation technology will eventually lead to a personalized system for drug and substance testing.

**Fig. 1 (abstract P-353).**
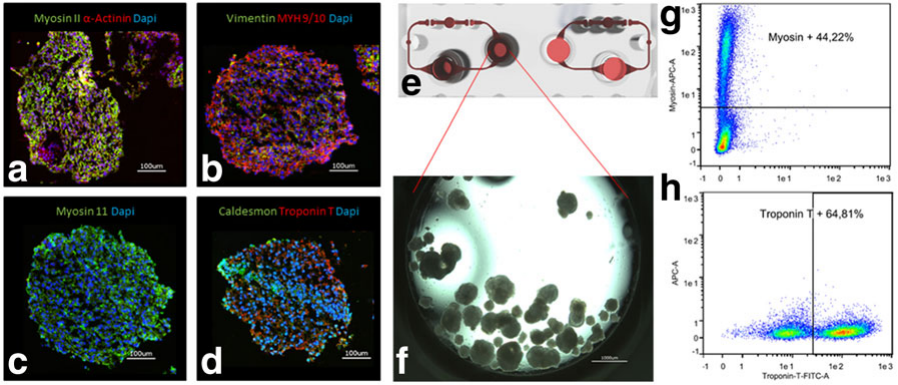
**a**-**d** Immunostaining of representative cardiomyocyte spheroids. **e** Channel structure of 2-Organ-Chip. **f** Cardiomyocyte spheroids cultivated under dynamic conditions in 2-Organ-Chip over 7 days. G-H: Characterization of cardiomyocyte spheroids by FACS analysis for **g** myosin heavy chain and **h** cardiac troponin T

## P-365 Lab as a service - automated cell-based assays

### Lena Schober, Moriz Walter, Andrea Traube

#### Laboratory Automation and Biomanufacturing Engineering, Fraunhofer IPA, Stuttgart, Germany

##### **Correspondence:** Lena Schober (lena.schober@ipa.fraunhofer.de)

**Background**

The use of cell-based assays in pharmaceutical industry and academic research is a growing trend that is a driving force to reduce costs for drug development. Academic research is gaining information about intracellular targets or functional mechanisms through the variety of different assays. These benefits can be used in preclinical studies and furthermore costly late-stage drug failures may be reduced by the use of cell-based assays. The use of automated systems is also in great demand and will change the testing of substances and research activities. Nevertheless, there are a lot of barriers at the moment limiting the successful application of automated systems in this field. By the lack of flexibility and the demand for skilled computer scientists & engineers just the two main aspects stated by experts shall be mentioned.

Our strong background on automated cell culture technologies and expertise, gained in several projects, let us rethink the overall process chain and overcome established principles. A new service orientated platform for the execution of cell-based assays that are commonly used will be introduced. The main idea is to give access to automated infrastructure for academic research or spin-offs which cannot afford the special infrastructure.

**Material and methods**

The infrastructure which was modularly built up, consists of automated liquid handling robots, plate and tube handling robots as well as incubators, refrigerator and analysis systems as for example an imaging system. The aim is to address the need on reproducibility and reliability of results and to offer access to a maximal controlled and automated environment. With the help of a web-based configurator assay selection as well as parameterization of the assays can be done in an easy way. After the order process, test items can be shipped to the lab. Assays will be executed on the fully automated platform. By capturing in process data as well as environmental conditions, a real complete data set is leading to comprehensively results. As soon as results are available during the process, the view and analysing can be done in a secure cloud.

**Results and conclusion**

The service can be used for single experiments in low throughput applications and is therefore a benefit for labs which cannot afford automated infrastructure or the staff for the maintenance for such platforms. Extensive monitoring and data capturing during the run leads to a gapless data trail and the possibility of detailed result analysis. Due to automated processing the reproducibility is increased associated with direct reduction of costs and time. The centralized service paired with specific know-how allows up-scaling of processes at any time. The web-based interface provides a flexible guidance for the user and the online order gives 24/7 access on the infrastructure, leading to a fast reliable result generation. Furthermore the secure interaction with additional services e.g. other specific data analysis tool is possible. This dynamic access to automation offers high flexibility for low throughput experiments and will push high quality research and drug development in early stage.

## P-366 Development of alternative animal cell technology platforms: CHO based cell-free protein synthesis systems for the production of “difficult-to-express” proteins

### Lena Thoring^1,2^, Marlitt Stech^1^, Srujan K Dondapati^1^, Doreen A Wuestenhagen^1^, Stefan Kubick^1^

#### ^1^Department of Cell-free and Cell-based Bioproduction, Fraunhofer Institute for Cell Therapy and Immunology (IZI), Branch Bioanalytics and Bioprocesses Potsdam, Potsdam, 14476, Germany; ^2^Department of Biotechnology, Technical University of Berlin, Berlin, 10623, Germany

##### **Correspondence:** Stefan Kubick (stefan.kubick@izi-bb.fraunhofer.de)

**Background**

Nowadays, animal cell technologies are commonly used for a broad range of medical and pharmaceutical applications. One main topic of these technologies is the production of proteins used for therapeutical purposes. These *in vivo* production processes are often time consuming and limited in production of so called “difficult-to-express” proteins including the pharmaceutical relevant class of membrane proteins. To overcome these issues, novel cell-free protein synthesis platforms were developed based on the industrial working horse CHO cells [1]. Cell lysates provide a basis for this technology by including all components of the translational machinery and enabling protein production within a few hours. Microsomal structures present in CHO cell lysates enable posttranslational modification of target proteins and insertion of membrane proteins into lipid bilayer.

**Materials and methods**

In this study a cell-free protein synthesis platform was developed based on a combination of CHO cell lysates and a continuous exchange reaction format. The continuous exchange reactor consists of a two-chamber system, a reaction and a feeding chamber, separated by a semipermeable membrane. Due to concentration gradients, energy components can diffuse to the reaction chamber, while inhibitory byproducts are continuously removed. Different classes of proteins were selected to evaluate the quality of the CHO CECF system including a transmembrane receptor, a single chain variable fragment and an ion channel. Cell-free protein synthesis was performed in the presence of ^14^C leucine for radio labeling of synthesized proteins. Protein yield was quantified by TCA precipitation of radio labeled proteins followed by scintillation measurement and molecular mass was detected by autoradiography. Posttranslational modifications and activities of proteins were estimated by kinase assays, ELISA, endoglycosidase treatment and electrophysiological measurements.

**Results**

The demonstrated results showed a protein production of up to around 1 g/l while detecting correct molecular weights by autoradiography. Analysis of the productivity using different lysate batches by the production of the membrane protein EGFR revealed only minimal batch-to-batch variations (Fig. 1a). Posttranslational modifications of proteins, including phosphorylation and glycosylation, were detected using western blot and autoradiography (Fig. 1b). Evaluation of localization of membrane embedded eYFP fusion proteins by confocal laser scanning microscopy resulted in the detection of proteins in the microsomal fraction of CHO cell lysate. Produced single chain variable fragments showed binding specificity in ELISA experiments. The activity of synthesized ion channels was underlined by electrophysiological measurements and detected single channel activities. A cell-free system based on CHO cell lysates for high yield production of proteins was developed that provides a platform for efficient production of “difficult-to-express” proteins.

**Conclusions**

The combination of a CHO lysate based cell-free system and a continuous exchange cell-free system leads to be a highly efficient production system for various classes of “difficult-to-express” proteins. This approach opens up a fast and cost-effective process pipeline for the production of “difficult-to-express” proteins and shows a high potential for industrial applications including screening technologies, protein structure determination and just-in-time protein production processes.

**Acknowledgements**

The authors would like to thank Dana Wenzel for CHO lysate preparation (Fraunhofer IZI, Potsdam-Golm, Germany). This work is supported by the European Regional Development Fund (EFRE) and the German Ministry of Education and Research (BMBF, No. 031B0078A).

**References**

1. Thoring L, Wüstenhagen D A, Borowiak M, Stech M, Sonnabend A, Kubick S: Cell-Free Systems Based on CHO Cell Lysates: Optimization Strategies, Synthesis of “Difficult-to-Express” Proteins and Future Perspectives. PLoS ONE, 11(9).

2. Brödel A K, Sonnabend A, Kubick S: Cell-free protein expression based on extracts from CHO cells. Biotechnol. Bioeng. (Biotechnology and Bioengineering), 1: 25–3.

3. Jerome V, Thoring L, Salzig D, Kubick S, Freitag R: Comparison of cell-based vs. cell-free mammalian systems for the production of a recombinant human bone morphogenic growth factor. Engineering in Life Sciences.

4. Brödel A K, Sonnabend A, Robert L O, Stech M, Wüstenhagen D A, Kubick S: IRES-Mediated Translation of Membrane Proteins and Glycoproteins in Eukaryotic Cell-Free Systems PLoS ONE, 8(12).

**Fig. 1 (abstract P-366).**
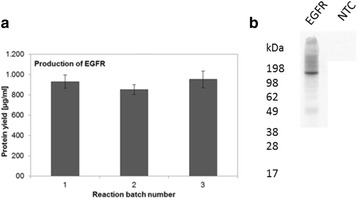
**a** Determination of protein yield of EGFR (Epidermal growth factor receptor) synthesized in a CHO CECF system. Analysis of EGFR protein yield obtained in a various batches of CECF formatted reaction. CECF synthesis was performed in the presence of ^14^C leucine for radio labeling of target proteins. Radio labeled proteins were precipitated using TCA followed by scintillation measurement. **b** Detection of radio labeled EGFR by autoradiography. A no template control (NTC) was prepared containing no EGFR DNA template

## P-368 Production of recombinant factor VII in Sk-Hep-1 human cell line

### Marcela C. C. Freitas^1,2^, Aline S. Bomfim^1, 3^, Vladmir Granovski^1,2^, Virgínia Picanço-Castro^1,2^, Dimas T Covas^1,2^

#### ^1^Center for Cell-based Therapy and Regional Blood Center of Ribeirão Preto, Laboratory of Biotechnology, University of São Paulo, Ribeirão Preto, São Paulo, ZIP 14051-140,Brazil; ^2^Department of Medical Clinic, Medical School of Ribeirão Preto, University of São Paulo, Ribeirão Preto, São Paulo, ZIP 14049-900, Brazil; ^3^Department of Clinical, Toxicological and Food Science Analysis, Faculty of Pharmaceutical Sciences of Ribeirão Preto, University of São Paulo, Ribeirão Preto, São Paulo, ZIP 14040-903, Brazil

##### **Correspondence:** Marcela C. C. Freitas (marcelafreitas@usp.br)

**Background**

Nowadays it is known that the development of inhibitory antibodies by hemophiliac patients is closely related with immunogenic epitopes present in the coagulation factors. These proteins are produced in hamsters cells [1 – 4] which insert a different post-translational modification profile when compared with the human profile. Patients with high-titer/high-responding inhibitors must be treated with bypassing agents that can achieve hemostasis. Activated factor VII (FVIIa) is an attractive candidate for hemostasis, independent of FVIII/FIX, making this coagulation factor an alternative for hemophilia patients with inhibitory antibodies. However recombinant factor VII is produced in BHK-21 cells (Baby hamster kidney cells) and as well as the others coagulation factors, it may contain immunogenic epitopes [5 - 7]. In this context, becomes extremely important to produce recombinant proteins with complex posttranslational modifications in a cell line not yet used [8 – 10].

**Materials and methods**

We have been using the Sk-Hep-1 human cell line for the production of recombinant FVII. To generate the recombinant cell line we have used a bicistronic lentiviral vector, 1054-GFP, containing a FVII gene and the GFP selection marker gene. A master cell bank and a work cell bank were generated in GMP conditions. The rFVII analyses were made by ELISA assay, western blot, gene expression quantification and biological activity using the Prothrombin Time (PT) assay. rFVII purification by affinity chromatography using VIISelect (GE) column. After purification the rFVII was formulated and dry froze to be used in in vivo experiments.

**Results**

In static conditions Sk-Hep-1 cells showed, for a period of 6 months, a stable FVII production with an average of 8,03 IU/mL of FVII, 83% of cell viability and 77% of cells expressing the GFP gene. After purification with VIISelect column it was possible observe a recover of 65% of the purified protein with 95% degree of purity (Fig. 1). This recombinant purified FVII is being used in in vivo experiments to determine the pharmacokinetics parameters and to evaluate the post-translation modifications profile.

**Conclusion**

In conclusion, this study reports the use of Sk-Hep-1 cell line for high-level production of recombinant factor VII. These cells have proven to be effective in the production of recombinant protein and can be used as a new platform for the production of recombinant proteins.

**Acknowledgements**

The authors acknowledge São Paulo Research Foundation – FAPESP (2015/19017-6), Centro de Pesquisa, Inovação e Difusão (CEPID), and National Institute of Science and Technology in Stem Cell and Cell Therapy – INCTC for financial support.

**References**

1. Fliedl L, Grillari J, Grillari-Voglauer R: **Human cell lines for the production of recombinant proteins: on the horizon.** *N Biotechnol*. 2015, 32(6):673-9.

2. Wurm FM: **Production of recombinant protein therapeutics in cultivated mammalian cells**. *Nat Biotechnol.* 2004, 22(11):1393-8.

3. Jayapal KP, Wlaschin KF, Hu W-S, Yap MGS: **Recombinant protein therapeutics from cho cells – 20 years and counting**. *Chem Eng Prog* 103: 40-47.

4. Halabian R, Roudkenar MH, Esmaeili NS, Masroori N, Roushandeh AM, Najafabadi AJ: **Establishment of a cell line expressing recombinant factor VII and its subsequent conversion to active form FVIIa through hepsin by genetic engineering method**. *Vox Sang*. 2009. 96(4):309-15.

5. Xiao W, Li CQ, Xiao XP, Lin FZ: **Expression and fast preparation of biologically active recombinant human coagulation factor VII in CHO-K1 cells**. *Genet Mol Res*. 2013. 12(4):6813-24.

6. Ghaderi D, Taylor RE, Padler-Karavani V, Diaz S, Varki A: **Implications of the presence of N-glycolylneuraminic acid in recombinant therapeutic glycoproteins**. *Nat Biotechnol*. 2010. 28(8):863-7.

7. Varki A: **Uniquely human evolution of sialic acid genetics and biology**. *Proc Natl Acad Sci.* 2010.107(Suppl.):8939–46.

8. Ghaderi D, Zhang M, Hurtado-Ziola N, Varki A: **Production platforms for biotherapeutic glycoproteins. Occurrence, impact, and challenges of non-human sialylation**. *Biotechnol Genet Eng Ver*. 2012. 28:147–76.

9. Swiech K, Picanço-Castro V, Covas DT: **Human cells: new platform for recombinant therapeutic protein production**. *Protein Expr Purif*. 2012. 84(1):147-53.

10. Lalonde ME, Durocher Y: **Therapeutic glycoprotein production in mammalian cells**. *J Biotechnol*. 2017. 251:128-140.

**Fig. 1 (abstract P-368).**
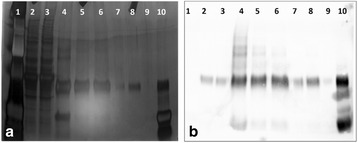
Recombinant Factor VII purification process. In **a**, silver nitrate stained SDS-Page gel showing the decreasing of total protein amount and the increasing of FVII purity degree. In **b**, Western Blot showing the presence of rFVII during the several steps of protein purification. 1 – Molecular weigh marker; 2 – Cell supernatant; 3 - Flow through; 4, 5 and 6 – VIISelect column elution; 7 and 8 - Q Sepharose FF column elution; 9 – no sample; 10 – NovoSeven (10 μg)

## P-376 Soft biocompatible microcarriers for adipose-derived mesenchymal stem cells expansion

### Dominique Gallo^1^, Elisabeth Bodo^1^, Catherine Saint-Hubert^1^, Alain Durieux^1^, Ruben Werquin^2^, John Werenne^3^, Serge Lowagie^4^

#### ^1^Institut de recherches microbiologiques Wiame, Brussels, Belgium; ^2^Meurice R&D, Brussels, Belgium; ^3^Biocelan, Université Libre de Bruxelles, Brussels, Belgium; ^4^Oh-Cell, Beloeil, Belgium, 7970

##### **Correspondence:** Serge Lowagie (serge.lowagie@skynet.be)

**Background**

Emergence of stem cell-based regenerative medicine recently leaded to the necessity to reach a sustained production of such cells [1]. Hence, new bioreactors and carriers were designed for cell expansion. However, to meet this increasing demand, improvement of both quality and quantity of stem cells remains necessary. Soft biocompatible microcarriers mimicking extracellular matrix in term of structure and stiffness should be of valuable utility as substrate stiffness strongly influence *in vitro* stem cell fate and differentiation [2,3].

**Material and methods**

Our expertise in the field of microbeads design using jetcutting technology [4] enabled us to engineer +/- 200 μm alginate beads of various G/M monomer ratio. We used jetcutter (geniaLab Gmbh) with 100 μm nozzle at max speed 12000 rpm. Alginate solutions with concentrations 2% to 4% were gelifyed in 2% CaCl2 EtOH 50% solution. Alginates with estimated viscosity (@1%) from 30 to 720 mPa were tested. A further surface treatment with gelatine (0,1%, 1%) and poly-L-lysine (0,1%) was carried out to reach an optimal cell anchoring of human adipose-derived mesenchymal stem cells (ATCC-PSC-500-011) in MesemPro RS medium (Gibco).

**Results and conclusions**

Jetcutter technology allowed us to obtain alginate microcarriers with a good homogeneity in size around 200 μm and sphericity comparable to commercial carriers (Table 1). Best adhesion of human adipose-derived mesenchymal stem cells was obtained on 0,1% gelatine coated alginate carriers (Fig. 1). We observed limited apoptosis and human adipose-derived mesenchymal cells stemness was conserved after 14 days in culture (data not shown).

**References**

1. Jean-Paul Prieels et al. **Masthering industrialization of cell therapy products.** *Bioprocess International* 2012 **10(3).**

2. Dennis E. Discher et al. **Tissue cells feel and respond to the stiffness of their substrate.** *Science* 2005 **310.**

3. Adam J. Engler et al. **Matrix elasticity directs stem cell Lineage specification.** *Cell* 2006 **126** : 677-689.

4. Nedovic VA et al. **Continuous cider fermentation with co-immobilized yeast and Leuconostoc oenos cells.** *Enzyme Microb. Technology* 2000 **26 (9-10)**: 834-839.

**Table 1 (abstract P-376).** Alginate microcarriers characterization

	Mean size (μm)	Distribution factor (μm)	Sphericity	Surface (mm^2^)
A2^a^ (3%)	193	19	0,96	0,117
Cytodex3	227	22	0,94	0,164

^a^Alginate with estimated viscosity 50-150 mPa %G/M ratio 35-65

**Fig. 1 (abstract P-376).**
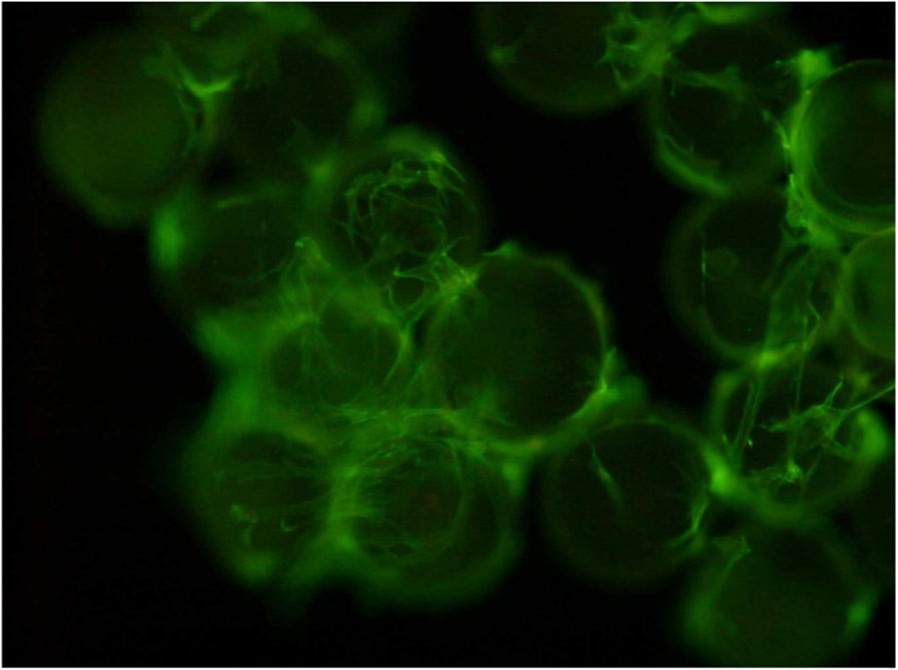
Human adipose-derived mesenchymal stem cells grown on alginate microcarrier 0,1% gelatine coated (green – phalloidine-FITC marking of F-actin filaments)

## P-381 Cellular bioassays developed with functionally immortalized cell lines

### Aileen Bleisch^1^, Aleksandra Velkova^3^, Tom Wahlicht^2^, Dagmar Wirth^2^, Tobias May^1^

#### ^1^InSCREENeX GmbH, Braunschweig, Germany; ^2^MSYS, Helmholtz Centre for Infection Research, Braunschweig, Germany; ^3^Greiner Bio-One GmbH, Frickenhausen, Germany

**Background**

A major challenge of current research is the limited availability of physiologically relevant cells [1]. Thus the development of relevant cellular bioassays that are robust, reproducible and scalable is hindered. To overcome current limitations we developed an immortalization strategy allowing the efficient and reproducible establishment of novel cell lines showing an *in vivo*-like phenotype.

The main feature of our CI-SCREEN technology is the ability to combine the advantage of cell lines – the unlimited cell supply – with the advantage of primary cells – the physiological relevance. Using this technology we have immortalized, amongst others, a human osteoblast cell line (CI-huOB) [2]. In the present study, the in vivo-like phenotype and functionality of the novel CI-huOB was examined. Therefore, CI-huOB cells were used to develop a 3D cell culture model by using the magnetic 3D bioprinting technology (Nano3D Biosciences, Houston, TX, USA) [3].

**Materials and methods**

The CI-huOB cell line was recently described and cultivated in huOB Maintenance Medium (InSCREENeX, Germany). For spheroid creation CI-huOBs were grown in a monolayer, magnetized by adding a magnetic nanoparticle assembly (NanoShuttle, NS, Nano3D Biosciences, Houston, TX, USA) at a concentration of 4μl NS/cm^2^ growth area. After an overnight incubation magnetized CI-huOB were detached and seeded into Cellstar® Cell-Repellent 96-well plates (Greiner Bio-One, Frickenhausen, Germany). With the help of mild magnetic forces cells were printed into spheroids within 2h. These consist of 1.000-50.000 cells and were cultured for a period of up to 50 days. The cell viability was analyzed by a Propidium iodide (PI) and Calcein AM staining. To improve spheroid functionality spheroids were cultivated with huOB Differentiation Medium (InSCREENeX, Germany). “Mini bone” tissue functionality and thus mineralization was analyzed by an alkaline phosphatase (alkaline phosphatase activity) and an alizarin red S Staining (Ca^2+^deposits).

**Results**

The combination of CI-huOB cells with the magnetic 3D bioprinting technology enabled the establishment of reproducible and consistent 3D spheroids. Single spheroids per well were formed independent of the amount of cells (1.000-50.000 cells) (Fig. 1a). Formed spheroids were stable for a culture period of up to 50 days (Fig. 1b). Neither cell death nor cell proliferation were observed in the bioprinted spheroids which is indicated by the stable size of the spheroids throughout the cultivation (Fig. 1c). After treatment with a differentiation stimulus the 3D bioprinted spheroids became fully functional “mini bones”. This was highlighted by the alkaline phosphatase activity and the Ca^2+^ deposits within the 3D bioprinted spheroids (Fig. 1d,e).

**Conclusion**

Taken together, these results demonstrated that the functional immortalization technology provides physiologically relevant cells in sufficient numbers and that the magnetic 3D Bioprinting technology enabled a fast, consistent cell aggregation and the formation of stable uniform spheroids. Importantly, these immortalized cells are capable to differentiate when a suitable stimulus is provided. For differentiation into mini bones, 3D spheroid cultivation and additional stimulation by small molecules are required. The combination of physiologically relevant cell systems with three dimensional culturing will help to generate in vitro test systems which closely resemble the in vivo physiology and thereby supporting future drug discovery approaches.

**Acknowledgements**

This work was supported by grants from the Niedersächsisches Ministerium für Wissenschaft und Kultur (80029155) and the German Ministry for Economic Affairs and Energy (IGF 16153 N).

**References**

1. Lipps C, May T, Hauser H, Wirth D. Eternity and functionality - rational access to physiologically relevant cell lines. Biol Chem. Dec (2013);394 (12):1637-48.

2. Pérez-Campo FM, May T, Zauers J, Sañudo C, Delgado-Calle J, Arozamena J, Berciano MT, Lafarga M, Riancho JA. Generation and characterization of two immortalized human osteoblastic cell lines useful for epigenetic studies. J Bone Miner Metab. 2017 Mar;35(2):150-160. doi: 10.1007/s00774-016-0753-z.

3. n3D Biosciences and Greiner Bio-One, Biocompatibility of NanoShuttleTM and the magnetic field in magnetic 3D bioprinting, 2014, (Online), Available: http://www.n3dbio.com/wp-content/uploads/2014/05/Nanoshuttle-Biocompatibility-App-Note.pdf. [Accessed: 28.08.2017]

**Fig. 1 (abstract P-381).**
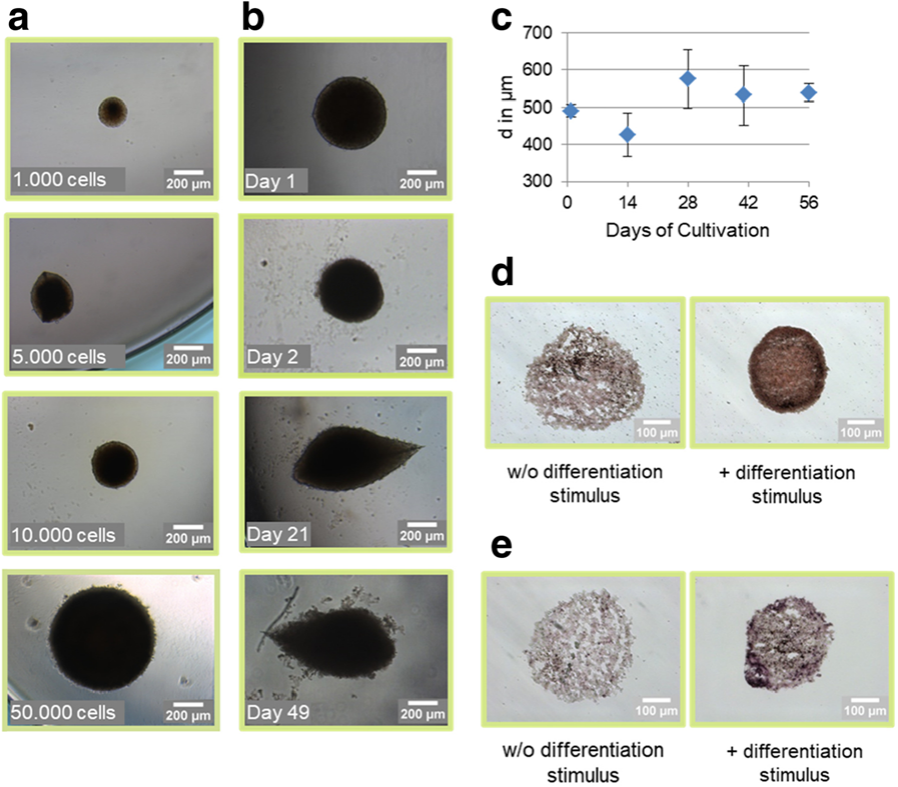
Characterization of spheroid “mini bones”. **a** Different number (1.000-50.000 cells) of CI-huOB cells were printed into spheroids. **b** 20.000 CI-huOBs were printed into spheroids and cultivated for indicated time points. **c** For analyzing spheroid sizes, pictures were taken and quantified by ImageJ. (D/E) 20.000 CI-huOB cells were printed into spheroids and cultivated with (huOB Differentiation Medium) or without a differentiation stimulus for two weeks. Afterwards, bioprinted spheroids were sectioned by a cryo microtome and **d** stained for Ca^2+^ deposits (Alizarin Red S) or **e** stained for alkaline phosphatase activity

